# Catalytic, Enantioselective Cycloaddition of Pyrrole-2*-*methides with Aldehydes toward a Synthesis of 2,3-Dihydro-1*H*-pyrrolizin-3-ols

**DOI:** 10.1021/acs.orglett.4c03081

**Published:** 2024-09-25

**Authors:** Philipp Stehr, Johannes Zyrus, Christoph Schneider

**Affiliations:** Institut für Organische Chemie, Universität Leipzig, 04103 Leipzig, Germany

## Abstract

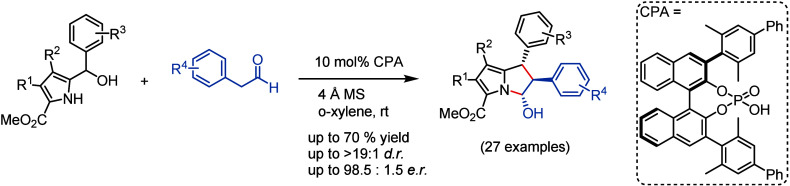

An organocatalytic, highly enantioselective [6 + 2]-cycloaddition of 2-methide-2*H*-pyrroles with aryl acetaldehydes represents a novel and straightforward route toward densely substituted 2,3-dihydro-1*H*-pyrrolizin-3-ols, which were generated with good yields and high enantio- and diastereoselectivity. This one-step process involves a BINOL-phosphoric acid catalyzed reaction of 1*H*-pyrrole-2-carbinols with aryl acetaldehydes via the corresponding hydrogen-bonded, chiral 2-methide-2*H*-pyrroles.

The pyrrolizine motif is present in a variety of natural products displaying interesting pharmacological and biological activities.^1−3^ However, little effort has been made toward their asymmetric synthesis.^4−8^ The group of Zecchi employed a chiral amino acid as a stochiometric chiral auxiliary in an intramolecular diastereoselective cycloaddition of nitrones.^9−11^ In 2010, Cho et al. utilized an organocatalytic approach employing the Hayashi–Jørgensen and cinchona alkaloid based catalysts to facilitate a domino Michael-aldol cyclization of 2-pyrrole carbaldehydes and ketones and enals to access densely substituted 2,3-dihydro-1*H*-pyrrolizines.^12−14^ Finally, the Smith group employed an isothiourea catalyst in a Michael addition-lactonization/ring opening protocol to furnish pyrrolizine carboxylates with outstanding diastereo- and enantioselectivity.^15^

Indole-2-carbinols undergo facile dehydration in the presence of chiral Brønsted acid catalysts to generate hydrogen-bonded 2-methide-2*H*-indoles, which can be trapped by a wide variety of nucleophiles, furnishing acyclic and cyclic indole-based heterocycles with excellent enantioselectivity.^16^ Especially, the seminal work of Han,^17,18^ Shi,^19−30^ Sun,^31−35^ and ourselves^36−40^ has demonstrated the utility and application of indole-2-carbinols in the synthesis of various highly enantiomerically enriched indole derivatives.

In recent years, we have developed several chiral BINOL phosphoric acid catalyzed formal [6 + 2]-cycloadditions of pyrrole-3-carbinols with cyclic enamides^41^ and 2-vinylindoles^42^ and of pyrrole-2-carbinols with 2-vinylindoles^43^ to afford cyclopenta[*b*]pyrroles and dihydropyrrolizines with excellent yields and enantioselectivities, respectively (Scheme 1A). Similar to indole-2-carbinols, pyrrole-2-carbinols undergo facile dehydration to form reactive 2-methide-2*H*-pyrroles (or azafulvenes). These are important intermediates in the biosynthesis of porphyrines^44^ or other natural products such as *nonylprodigiosin*.^45^ But unlike biosynthetic processes, the application in synthetic organic chemistry is problematic due to the exceptional reactivity of the pyrrole core toward electrophilic attack, resulting in facile oligomerization reactions. This, however, was intentionally avoided by placing an electron-withdrawing ester group at the pyrrole C5-position to attenuate the electron density of the heterocycle.

**Scheme 1:**
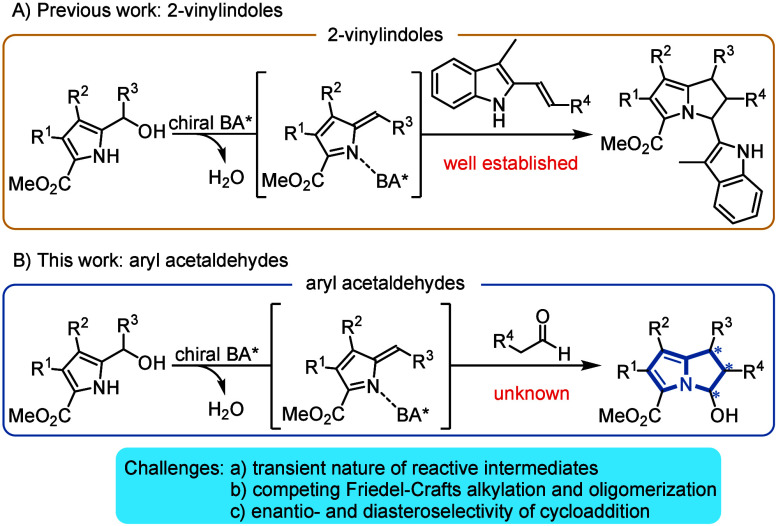
Conceptualization of This Work

In earlier work, we had used *o*-hydroxybenzhydryl alcohols en route to transient *o*-quinone methides and employed them in a formal [4 + 2]-cycloaddition with aldehyde enols to form 3,4-diaryl dihydrocoumarins with good enantioselectivity.^46^ In analogy to that concept, we envisioned that the same strategy might be applied to pyrrole-2-carbinols to allow for the first enantioselective synthesis of 2,3-dihydro-1*H*-pyrrolizin-3-ols **4** (Scheme 1B). An additional bonus of the latter approach presented in this work is that the hemiaminal moiety can be further manipulated and a variety of different substituents can be introduced into the 2,3-dihydropyrrolizine backbone subsequent to the actual cycloaddition.

We initiated our investigations with reactions of 5-ethoxycarbonyl-1*H*-pyrrol-2-yl carbinol (**1a**), which was readily available by Grignard addition from the corresponding pyrrolyl carbaldehyde.^47^ Treatment of carbinol **1a** with phenylacetaldehyde (**2a**) in the presence of (*R*)-BINOL-derived phosphoric acid **3a** in toluene at rt led to the formation of all-*trans* cycloaddition product **4a** in moderate yield and poor enantioselectivity (Table 1, entry 1). Fortunately, the major diastereomer could be fully separated from the other diastereomers by column chromatography. Subsequently, various other phosphoric acids **3a**–**e** with varying 3,3′-aryl substituents on the BINOL backbone were also tested under the same reaction conditions (for further details and studies with more catalysts, see the Supporting Information). Both the steric size of the 2,6-position and the 4-position within the 3,3′-aryl groups proved important for a highly stereoselective process. Eventually, it turned out that catalysts carrying 4-*tert*-butyl-2,6-dimethyl and 4-phenyl-2,6-dimethyl phenyl groups (as in **3c, d**) in the 3,3′-BINOL positions gave rise to optimal results (entries 3 and 4). In particular, **3d** furnished product **4a** with good enantio- and diastereoselectivity, albeit in a moderate yield (entry 4). The yield was further improved by running the reaction in *o*-xylene as the solvent and in the presence of 4 Å molecular sieves, which also improved the enantioselectivity. Thus, product **4a** was isolated as a pure diastereomer with 62% yield and 94:6 er (entry 8). Other solvents gave rise to inferior results (see Supporting Information). A recurring side reaction which could never be completely suppressed was the self-dimerization of the 1*H*-pyrrol-2-yl carbinol **1a** via the transient 2-methide-2*H*-pyrrole (see the Supporting Information).^48^

**Table 1:**
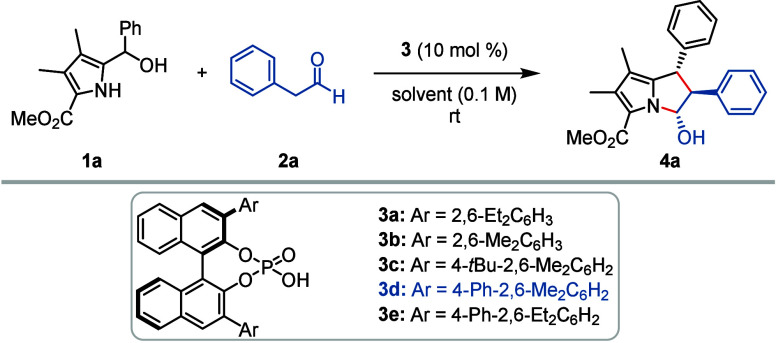

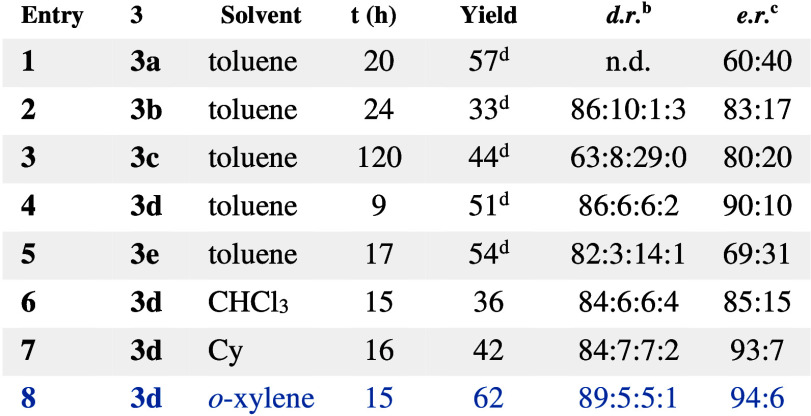
Reaction Optimization^a^

a Reaction conditions: 0.10 mmol **1a**, 0.30 mmol **2a**, catalyst **3** (10 mol %) and 40 mg 4 Å molecular sieves in 1 mL of solvent at rt.

b The diastereomeric ratio (*d.r*.) was determined by ^1^H NMR.

c The enantiomeric ratio (*e.r*.) was determined by HPLC analysis on a chiral stationary phase.

d Yields were determined by ^1^H NMR with 2,4-dinitrobenzoic acid methyl ester as internal standard.

Having optimized catalyst and reaction conditions, we set out to further explore the scope and limitations of this reaction. The aldehyde scope was investigated first by reacting differently substituted aryl acetaldehydes **2** with 1*H*-pyrrol-2-yl carbinol **1a** (Scheme 2). Methyl substitution at different positions in the aryl moiety was readily tolerated, furnishing the products **4b**–**d** in good yields and good diastereo- and enantioselectivity. *ortho*-Substitution as in **4b** led to a slight decrease in enantioselectivity, likely due to steric congestion in the transition state. Introducing halogen atoms at different positions of aryl acetaldehyde **2** was readily possible. Electron-rich aryl groups gave rise to pyrrolizines **4h**–**i** in particularly good yields with an excellent enantioselectivity of 95:5 *e.r*. for *para*-methoxy substitution. On the contrary, electron-poor aryl acetaldehydes such as *para*-CF_3_ resulted in significantly lower yields and enantioselectivities in product **4j** (85:15 *e.r*.). Aliphatic aldehydes were not competent reaction partners, likely due to their lower enol content.

**Scheme 2:**
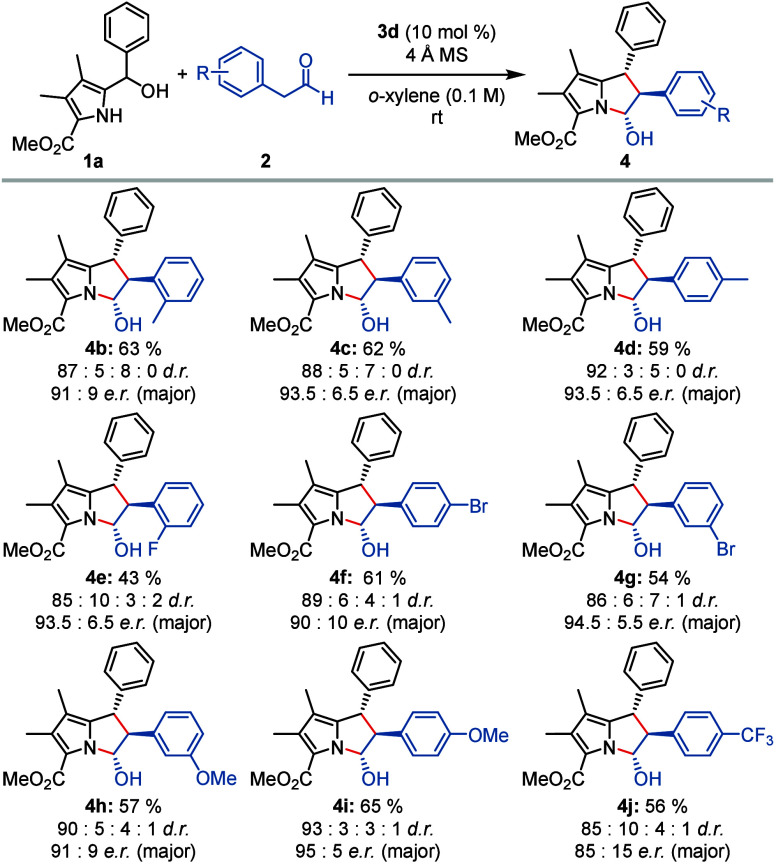
Scope of the Cycloaddition on the Aryl Acetaldehyde Side Reaction conditions: 0.20 mmol of 1*H*-pyrrol-2-yl carbinol **1a**, 0.60 mmol of aryl acetaldehyde **2**, catalyst **3d** (10 mol %), 80 mg of 4 Å molecular sieves, *o*-xylene (2 mL), rt; yields are given for the pure all-*trans* diastereomer; diastereomeric ratio was determined via ^1^H NMR; enantiomeric ratio was determined by HPLC on a chiral stationary phase (see the Supporting Information).

We next aimed to study the scope of the cycloaddition on the pyrrole carbinol side by subjecting different 1*H*-pyrrolyl-2-carbinols **1** to reactions with phenylacetaldehyde (**2a**) and *para-*methoxy phenylacetaldehyde (**2i**) (Scheme 3). In general, fully substituted pyrroles must be employed for complete conversions. Any alkyl substitution in the pyrrole-3- and 4-positions was well tolerated and gave rise to the desired cycloaddition products in good yields and enantioselectivities. A 4-phenyl-substituted pyrrole furnished the desired product **4x** only with the more reactive *para-*methoxy phenylacetaldehyde (**2i**), albeit in a poor yield of 26% and only moderate enantioselectivity. Furthermore, the structural diversity could be increased by incorporating a butyl bridge between the 3- and 4-position of the pyrrole core. Thus, the hexahydro-1*H*-pyrrolo[2,1-*a*]isoindole (**4l**) was obtained in good yield and with good enantioselectivity.

**Scheme 3:**
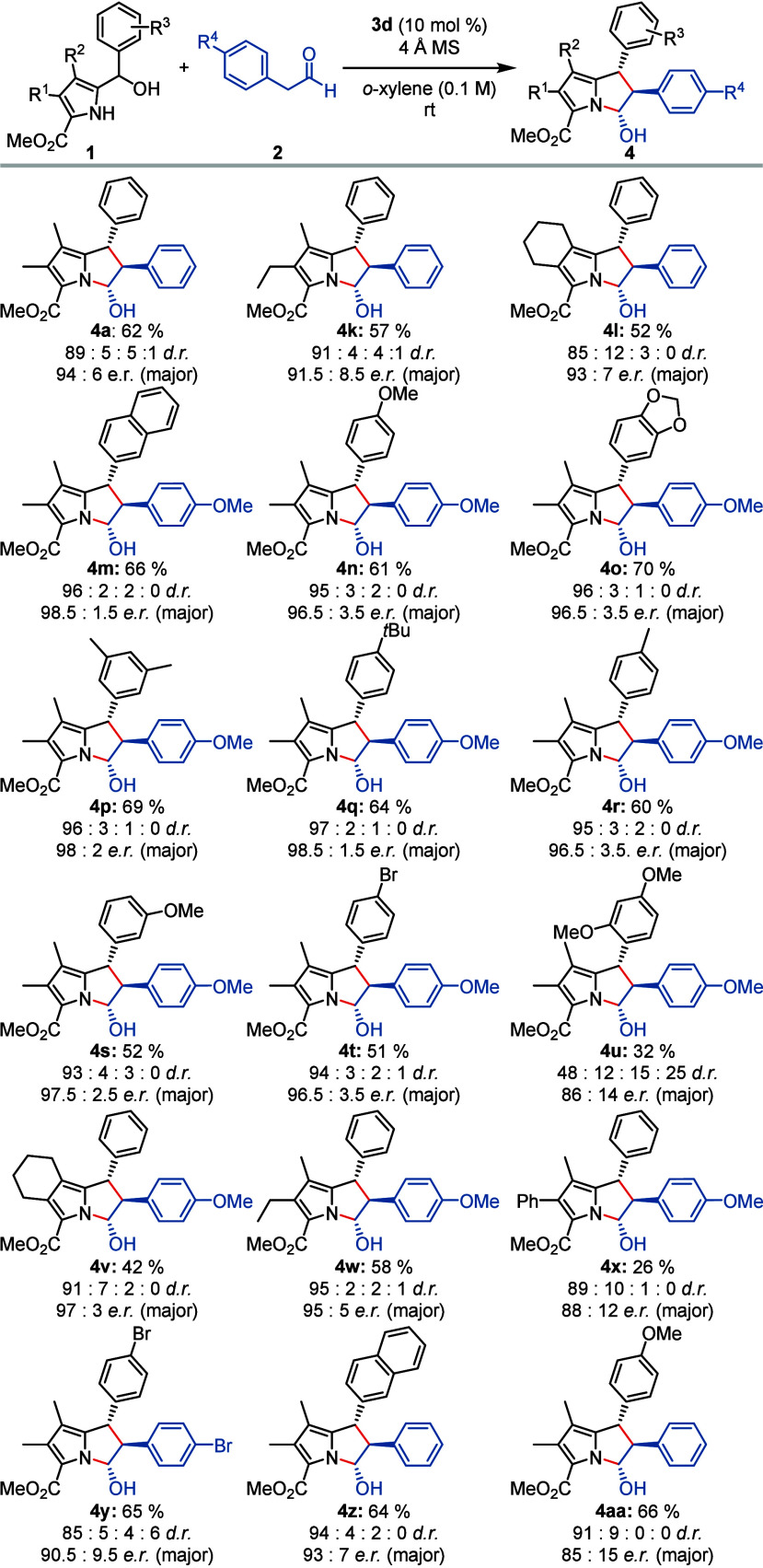
Scope of the Cycloaddition on the Pyrrole-2-carbinol Side Reaction conditions: 0.20 mmol of 1*H*-pyrrol-2-yl carbinol **1**, 0.60 mmol of aryl acetaldehyde **2**, catalyst **3d** (10 mol %), 80 mg of 4 Å molecular sieves, *o*-xylene (2 mL), rt; yields are given for the pure all-*trans* diastereomer; diastereomeric ratio was determined via ^1^H NMR; enantiomeric ratio was determined by HPLC on a chiral stationary phase (see the Supporting Information).

Next, the aryl carbinol group was investigated. Electron-donating substituents gave rise to the most reactive substrates, likely because the dehydration proceeded more rapidly. The cycloaddition products **4m**–**s** were obtained in good yields of up to 70% and enantioselectivities of up to 98.5:1.5 *e.r*. Halogenated aryl groups as in brominated pyrrole-2-carbinol **1t** furnished the desired product **4t** with a slightly reduced yield of 51% but with excellent enantioselectivity. However, aryl residues with an *ortho*-substitution, such as **4u**, gave significantly reduced yields and enantioselectivities. Sterically more demanding substituents such as 2,6-dimethylphenyl gave no conversion.

In a comparison between reactions of phenylacetaldehyde (**2a**) and *para-*methoxy phenylacetaldehyde (**2i**) with various pyrrole-2-carbinols **1k**–**m** a significant increase in enantioselectivity was noted for the more electron-rich aldehyde **2i**. We attribute this observation to the higher enol content of **2i** leading to an enhanced reaction rate and more efficient hydrogen bonding to the chiral bifunctional phosphoric acid catalyst.

The relative and absolute configuration of 2,3-dihydro-1*H*-pyrrolizin-3-ols **4f** was determined by single crystal X-ray diffraction analysis and was adopted for all other products as well (see the Supporting Information and CCDC 2376720).

Next, we demonstrated the practicality of this process by carrying out a large-scale experiment between pyrrole-2-carbinol **1m** and *para-*methoxyphenylacetaldehyde (**2i**) furnishing cycloadduct **4m** with a 66% yield and 98:2 *e.r*. (Scheme 4A).

**Scheme 4:**
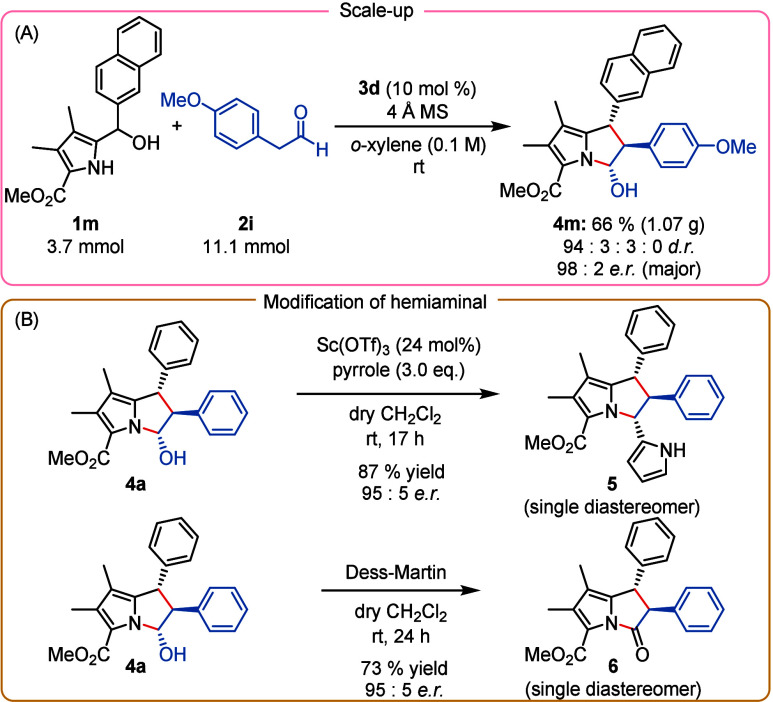
(A) Scale-up of the Enantioselective [6 + 2]-Cycloaddition and (B) Postmodifications of Hemiaminal 4a

Simple postmodifications of the hemiaminal moiety were conducted in two experiments to reveal the synthetic potential of the products (Scheme 4B). First, we transformed pyrrolizine **4a** with catalytic amounts of Sc(OTf)_3_ into the corresponding iminium ion which was trapped with pyrrole to furnish 3-(1*H*-pyrrol-2-yl)-pyrrolizine **5** in 87% yield with fully retained enantioselectivity as a single diastereomer. This diastereoselectivity is assumed to be governed by the nucleophilic attack of the pyrrole at the sterically more accessible face of the bicyclic iminium ion. Additionally, we could convert the hemiaminal moiety of **4a** into the corresponding lactam **6** by a mild oxidation with Dess-Martin periodinane which proceeded with 73% yield and fully retained relative and absolute configuration.

Based on earlier work of our group^41,43^ we propose the following transition state model to account for the observed diastereo- and enantioselectivity of the [6 + 2]-cycloaddition (Scheme 5). The BINOL-derived phosphoric acid connects and activates the transient pyrrole-2-methide and the enol tautomer of the aryl acetaldehyde through double hydrogen bonding, while the lower 3-aryl group within the BINOL backbone effectively shields the bottom side of the 2-methide pyrrole, directing the incoming enol to the top face.

**Scheme 5:**
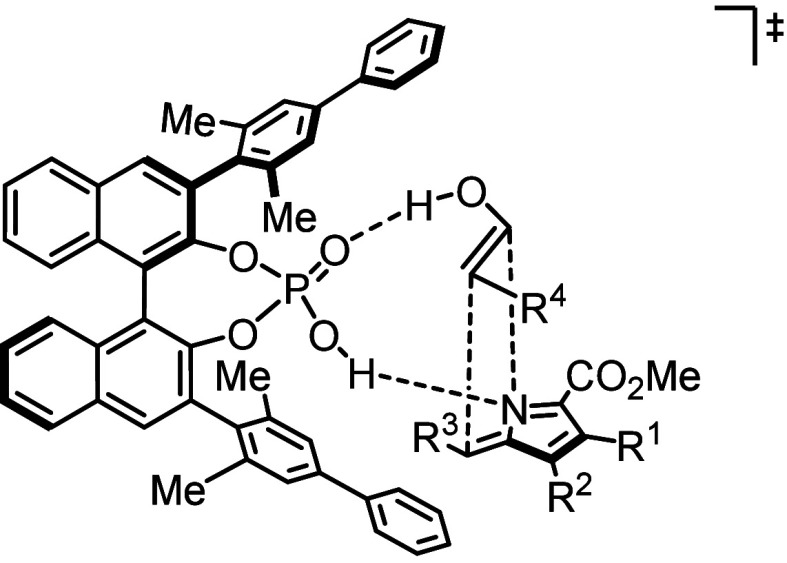
Plausible Transition State Assembly

In conclusion, we have developed the first catalytic, enantioselective [6 + 2]-cycloaddition of hydrogen-bonded 2-methide-2*H*-pyrroles with *in situ* generated aryl enols. This one-step procedure is catalyzed by a chiral BINOL-derived phosphoric acid and allows for direct access to highly substituted 2,3-dihydro-1*H*-pyrrolizin-3-ols with three contiguous stereocenters. The products **4** were typically obtained with good yields and diastereoselectivity, as well as high enantioselectivity.

## Data Availability

The data underlying this study are available in the published article and its online Supporting Information.

## Supporting Information Available

Experimental procedures and analytical data (^1^H and ^13^C NMR spectra and HPLC traces) for all new compounds and X-ray data for **4f** (PDF)
