# Supplementary material for: Catalytic, Enantioselective Cycloaddition of Pyrrole-2-methides with Aldehydes toward a Synthesis of 2,3-Dihydro-1H-pyrrolizin-3-ols
Source: Org Lett. 2024 Sep 25;26(39):8345–9. doi: 10.1021/acs.orglett.4c03081 (PMC11459514; doi:10.1021/acs.orglett.4c03081)
Supplement: Supplementary file 1 — ol4c03081_si_001.pdf [file ol4c03081_si_001.pdf]

Supporting Information

Catalytic, Enantioselective Cycloaddition of Pyrrole-2-Methides with Aldehydes towards a Synthesis of 2,3-Dihydro-1*H*-pyrrolizin-3-ols

Philipp Stehr, Johannes Zyrus, and Christoph Schneider*

Institut für Organische Chemie, Universität Leipzig, 04103 Leipzig, Germany

Corresponding author: Christoph Schneider, e-mail: schneider@chemie.uni-leipzig.de

Table of Contents

1. Methods ... 2
  1.1 General Information ... 2
  1.2 Catalyst and reaction conditions optimization for the cycloaddition ... 3
  1.3 General procedure for the preparation of racemic products (*rac*-**4**) ... 7
  1.4 Typical procedure for the catalytic enantioselective [6+2]-cycloaddition of reactive 2*H*-pyrrol-2-methides with *in situ* formed 2-phenylethen-1-ols ... 7
  1.5 Large scale synthesis of **4m** ... 26
  1.6 Procedure for functionalization of hemiacetal **4a** ... 26
  1.7 Isolation of Dimer ... 27
2. NMR Spectra of Products ... 29
3. HPLC Chromatograms ... 61
4. Crystallographic Data ... 90
5. References ... 97

1. Methods

1.1 General Information

All reactions in dry solvents were conducted under nitrogen or argon atmosphere. Liquid reagents and solvents were transferred via syringe using standard Schlenk techniques. Dry toluene, tetrahydrofuran, diethyl ether and dichloromethane were dried by a solvent purification system (MB SPSP-800 Braun). Solvents for extraction, column chromatography, preparative and analytical TLC were distilled from appropriate drying agents (hexane, diethyl ether: KOH, dichloromethane: CaH_2, ethyl acetate: $CaCl_2$). Chloroform (HPLC grade) and methyl-*tert*-butyl ether were used without further purification. Flash chromatography was performed using Merck silica gel 60 230-400 mesh (0.040-0.063 mm). Preparative and analytical TLC was performed using silica gel pre coated plates ALUGRAM Xtra SIL G/UV_{254} by Macherey Nagel. Spots were visualized by UV light (λ = 254 nm) and were treated with a vanillin/sulfuric acid solution in MeOH (HPLC grade). NMR yields were determined by using 2,4-dinitrobenzoic acid methyl ester as an internal standard. BINOL-phosphoric acids[1], Pyrrole-2-carbinols[2] and aryl acetaldehydes[3,4] were known and prepared by following representative literature procedures.

^{1}H, ^{13}C, ^{19}F, ^{31}P NMR spectra were recorded in $CDCl_3$ or DMSO-d^6 using a Bruker Avance III HD (^{1}H: 400 MHz; ^{13}C: 100 MHz, ^{19}F: 376 MHz, ^{31}P: 162 MHz), a Varian MERCURYplus 400 (^{1}H: 400 MHz; ^{13}C: 100 MHz, ^{19}F: 376 MHz, ^{31}P: 162 MHz) and a Varian MERCURYplus 300 (^{1}H: 300 MHz; ^{13}C: 75 MHz, ^{31}P: 121 MHz) spectrometer. The signals were referenced to residual chloroform (δ = 7.26 ppm for ^{1}H NMR, δ = 77.16 ppm for ^{13}C NMR) or dimethyl sulfoxide (δ = 2.50 ppm for ^{1}H NMR, δ = 39.52 ppm for ^{13}C NMR). Chemical shifts are reported in ppm, multiplicities are indicated by s (singlet), d (doublet), t (triplet), q (quartet), dd (doublet of doublet), dt (doublet of triplet), td (triplet of doublet), qd (quartet of doublet), qt (quartet of triplet), ddd (doublet of doublet of doublets), ddt (doublet of doublet of triplets), dtd (doublet of triplet of doublets), dddd (doublet of doublet of doublet of doublets), m (multiplet) and the prefix br (broad). Melting points were determined with a Büchi M-560 melting point apparatus and are uncorrected. IR spectra were obtained with a FTIR spectrometer (JASCO FT/IR-4100) and are reported in frequency of absorption (cm^{-1}). Optical rotations were measured using a Polarotronic polarimeter (Schmidt & Haensch). All ESI mass spectra were recorded on a Bruker ESI-TOF microTOF. Enantiomeric ratios (*e.r.*) were determined via HPLC on a JASCO MD-4015 instrument with a chiral stationary phase (Daicel Chiralpak IA, IB, IE, column). Diastereomeric ratios were determined by HPLC or ^{1}H NMR analysis of the crude reaction mixture.

1.2 Catalyst and reaction conditions optimization for the cycloaddition

Table S1: Catalyst Screening[a)]

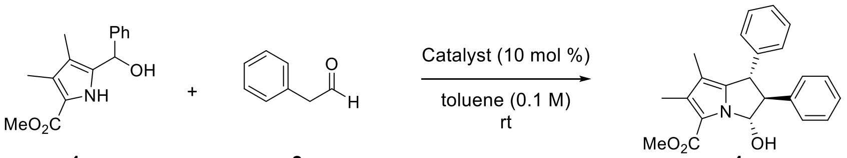

1a **2a** **4a**

Catalyst:

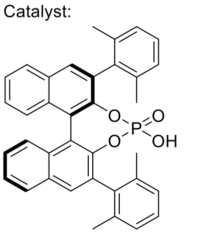	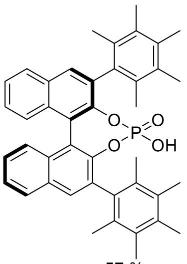	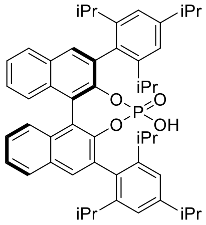	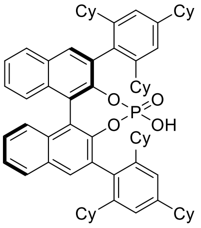
33 %<br>86 : 10 : 1 : 3 *d.r.*<br>83 : 17 *e.r.*	57 %<br>78 : 7 : 8 : 7 *d.r.*<br>76 : 24 *e.r.*	43 %<br>67 : 5 : 24 : 5 *d.r.*<br>92 : 8 *e.r.*	58 %<br>48 : 5 : 42 : 5 *d.r.*<br>47 : 53 *e.r.*
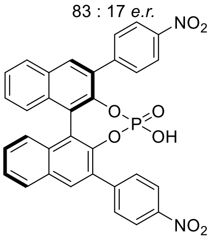	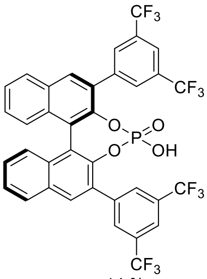	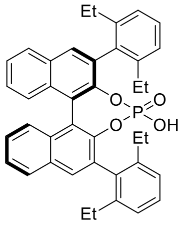	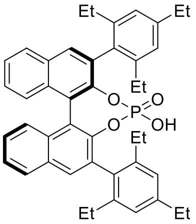
34 %<br>64 : 16 : 18 : 2 *d.r.*<br>60 : 40 *e.r.*	14 %<br>69 : 19 : 8 : 4 *d.r.*<br>68 : 32 *e.r.*	40 %<br>87 : 3 : 8 : 2 *d.r.*<br>73 : 27 *e.r.*	46 %<br>79 : 3 : 14 : 4 *d.r.*<br>85 : 15 *e.r.*
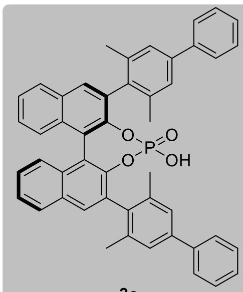	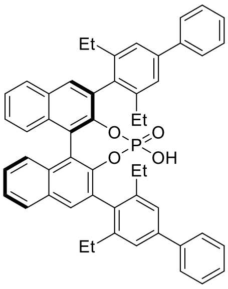	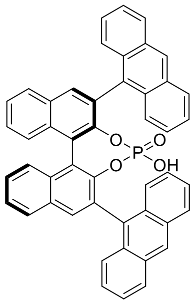	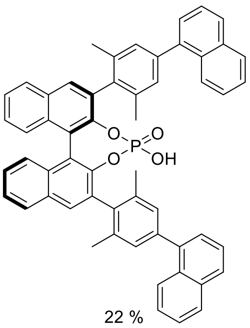
**3e**<br>51 %<br>86 : 6 : 7 : 1 *d.r.*<br>90 : 10 *e.r.*	54 %<br>82 : 3 : 14 : 1 *d.r.*<br>69 : 31 *e.r.*	41 %<br>80 : 7 : 12 : 1 *d.r.*<br>42 : 58 *e.r.*	22 %<br>81 : 14 : 4 : 1 *d.r.*<br>82 : 18 *e.r.*
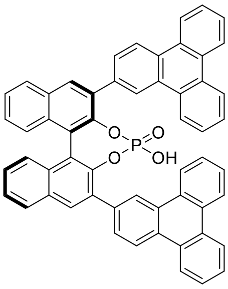	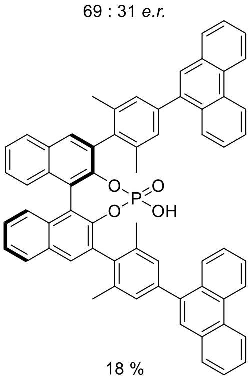	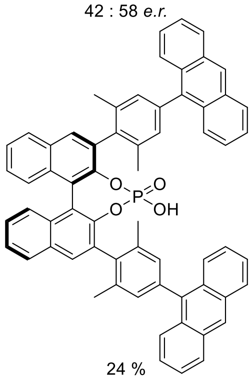	
14 %<br>83 : 13 : 3 : 1 *d.r.*<br>63 : 37 *e.r.*	18 %<br>84 : 10 : 3 : 3 *d.r.*<br>87 : 13 *e.r.*	24 %<br>74 : 9 : 5 : 12 *d.r.*<br>47 : 53 *e.r.*	

a) Reactions conditions: 0.10 mmol of **1a**, 0.30 mmol of aryl acetaldehyde **2a**, 10 mol% of catalyst **3** and 35 mg of 4 Å molecular sieves in 1 mL toluene. Diastereomeric ratio (*d.r.*) was determined by ^{1}H-NMR. Yields were determined by ^{1}H-NMR spectroscopy with 2,4-dinitrobenzoic acid methyl ester as internal

standard and are given for the all-*trans* diastereomer. Enantiomeric ratio (*e.r.*) was determined by HPLC analysis on a chiral stationary phase.

Table S2: Optimization of solvent[a)]

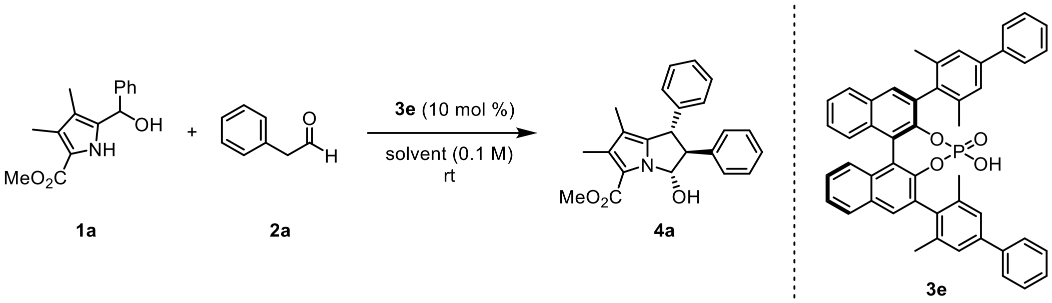

entry	solvent	time [h]	yield [%]	*d.r.*	*e.r.*
**1**	CH_2Cl_2	17	31	79 : 12 : 4 : 5	84 : 16
**2**	THF	no conversion			
**3**	$CHCl_3$	17	36	84 : 6 : 6 : 4	85 : 15
**4**	toluene	17	60	91 : 4 : 5 : 0	90 : 10
**5**	mesitylene	17	13	90 : 4 : 6 : 0	91 : 9
**6**	chlorobenzene	17	60	91 : 3 : 6 : 0	91 : 9
**7**	ethylbenzene	17	60	91 : 4 : 5 : 0	92 : 8
**8**	cyclohexane	16	42	85 : 7 : 6 : 2	93 : 7
**9**	*o*-xylene	21	62	89 : 5 : 5 : 1	94 : 6
**10**	hexane	18	36	84 : 7 : 9 : 0	87 : 13
**11**	methylcyclohexane	18	55	86 : 7 : 6 : 1	92 : 8
**12**	toluene/cyclohexane[b)]	21	61	90 : 4 : 6 : 0	93 : 7

a) Reactions conditions: 0.10 mmol of **1a**, 0.30 mmol of aryl acetaldehyde **2a**, 10 mol% of catalyst **3e** and 35 mg of 4 Å molecular sieves in 1 mL solvent. Diastereomeric ratio (*d.r.*) was determined by ^{1}H-NMR. Isolated yields for the pure all-*trans* diastereomer are given. Enantiomeric ratio (*e.r.*) was determined by HPLC analysis on a chiral stationary phase. b) 1:1 v/v

Table S3: Optimization of additives[a)]

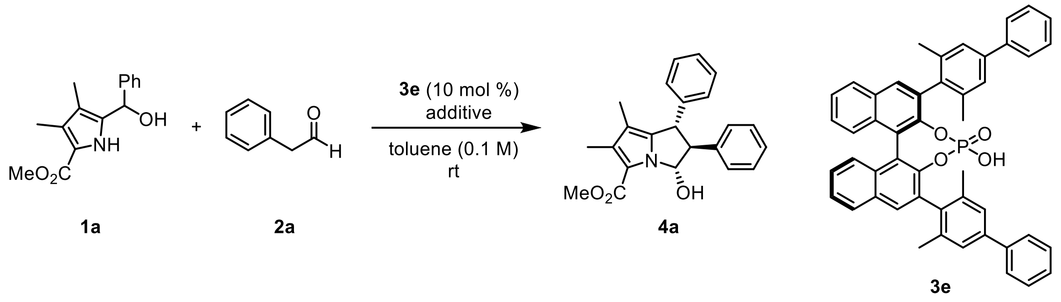

entry	additive	time [h]	yield [%]	*d.r.*	*e.r.*
**1**	none	15	31	72 : 24 : 4 : 0	80 : 20
**2**	1 equiv. water	15	37	71 : 24 : 4 : 1	80 : 20
**3**	4 Å MS	17	60	91 : 4 : 5 : 0	92 : 8
**4**	3 Å MS	22	51	91 : 3 : 5 : 1	92 : 8
**5**	$MgSO_4$[b)]	17	44	85 : 9 : 6 : 0	88 : 12
**6**	Na_2SO_4[b)]	17	40	75 : 19 : 6 : 0	80 : 20

a) Reactions conditions: 0.10 mmol of **1a**, 0.30 mmol of aryl acetaldehyde **2a**, 10 mol% of catalyst **3e** and 35 mg of 4 Å molecular sieves in 1 mL toluene. Diastereomeric ratio (*d.r.*) was determined by ^{1}H-NMR. Isolated yields for the pure all-*trans* diastereomer are given. Enantiomeric ratio (*e.r.*) was determined by HPLC analysis on a chiral stationary phase. b) 60 mg of drying agent were used instead of molecular sieves.

Table S4: Optimization of stoichiometry[a)]

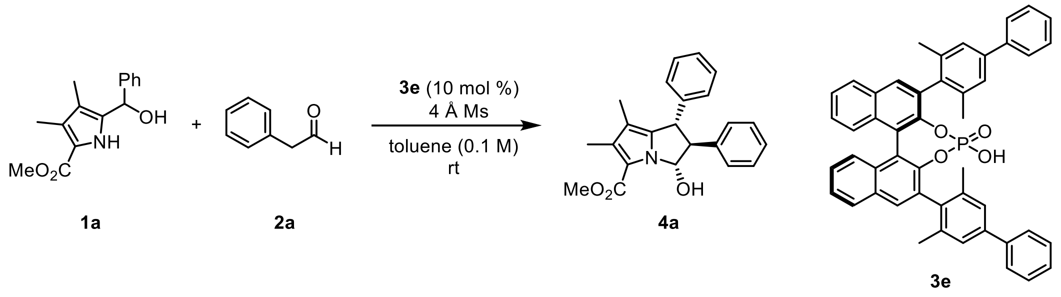

entry	carbinol	aldehyde	time [h]	yield [%]	*d.r.*	*e.r.*
**1**	1.00	1.00	22	41	91 : 3 : 5 : 1	89 : 11
**2**	1.00	2.00	22	58	91 : 3 : 6 : 0	n.d.
**3**	1.00	3.00	17	60	91 : 4 : 5 : 0	92 : 8
**4**	1.00	4.00	22	53	89 : 4 : 6 : 1	90 : 10
**5**	1.00	5.00	22	51	91 : 3 : 5 : 1	91 : 9
**6**	1.50	1.00	23	65	80 : 4 : 15 : 1	90 : 10

a) Reactions conditions: 0.10 mmol of limiting reaction partner was used, X mmol of excess reaction partner, 10 mol% of catalyst **3e** and 35 mg of 4 Å molecular sieves in 1 mL toluene. Diastereomeric ratio (*d.r.*) was determined by ^{1}H-NMR. Isolated yields for the pure all-*trans* diastereomer are given. Enantiomeric ratio (*e.r.*) was determined by HPLC analysis on a chiral stationary phase. n.d. : not determined.

Table S5: Optimization of catalyst loading and concentration[a)]

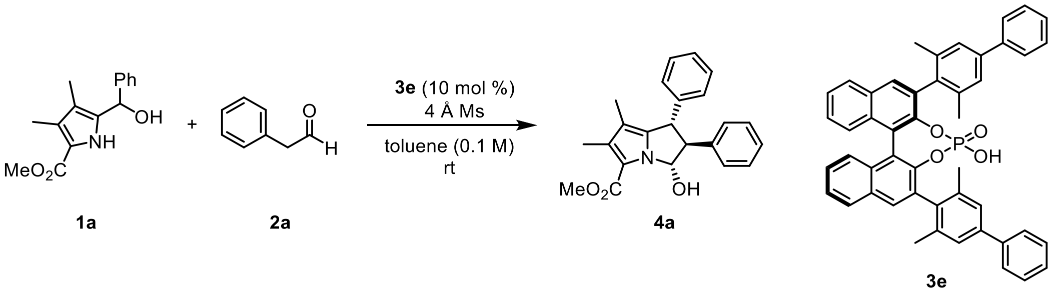

entry	concentration	catalyst loading	time [h]	yield [%][a)]	*d.r.*	*e.r.*
**1**	0.1 M	10 mol %	17	60	91 : 4 : 5 : 0	92 : 8
**2**	0.2 M	10 mol %	23	65	88 : 5 : 6 : 1	90 : 10
**3**	0.05 M	10 mol %	23	54	91 : 3 : 6 : 0	91 : 9
**4**	0.01 M[b)]	10 mol %	15	32	92 : 2 : 5 : 1	86 : 14
**5**	0.1 M	20 mol %	17	62	89 : 4 : 7 : 0	89 : 11
**6**	0.1 M	5 mol %	27	51	n.d.	87 : 13

a) Reactions conditions: 0.10 mmol of **1a**, 0.30 mmol of aryl acetaldehyde **2a**, X mol% of catalyst **3e** and 35 mg of 4 Å molecular sieves in Y mL toluene. Diastereomeric ratio (*d.r.*) was determined by ^{1}H-NMR. Isolated yields for the pure all-*trans* diastereomer are given. Enantiomeric ratio (*e.r.*) was determined by HPLC analysis on a chiral stationary phase. b) addition via syringe driver over 8 h. n.d. : not determined.

Table S6: Optimization of reaction temperature[a)]

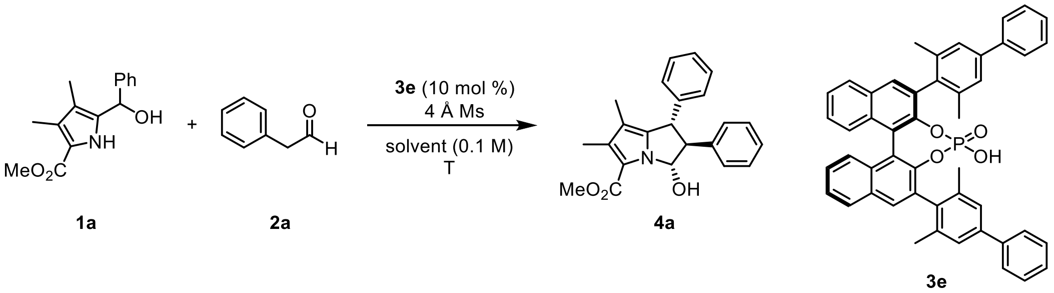

entry	solvent	temperature [°C]	time	yield [%]	*d.r.*	*e.r.*
**1**	toluene	-25	12 days	low conversion		
**2**	toluene	-10	4 days	37	94 : 4 : 2 : 0	92 : 8
**3**	toluene	5	1 day	46	91 : 4 : 4 :1	93 : 7
**4**	toluene	60	2 hours	55	87 : 4 : 9 : 0	91 : 9
**5**	*o*-xylene	5	1 day	54	91 : 5 : 4 : 0	92 : 8

a) Reactions conditions: 0.10 mmol of **1a**, 0.30 mmol of aryl acetaldehyde **2a**, 10 mol% of catalyst **3e** and 35 mg of 4 Å molecular sieves in 1 mL solvent. Diastereomeric ratio (*d.r.*) was determined by ^{1}H-NMR. Isolated yields for the pure all-*trans* diastereomer are given. Enantiomeric ratio (*e.r.*) was determined by HPLC analysis on a chiral stationary phase.

1.3 General procedure for the preparation of racemic products (*rac*-**4**)

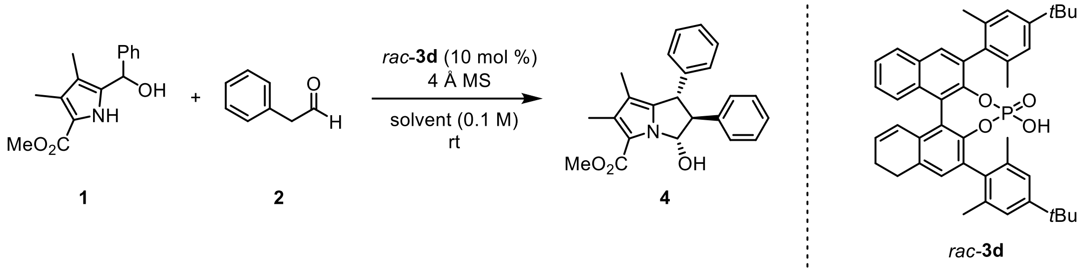

In a glass vial, (1*H*-pyrrol-2-yl)(aryl)methanol **1** (0.05 mmol, 1.0 equiv) and pulverized 4 Å MS (15 mg, 300 mg/mmol) were suspended in 0.5 mL *o*-xylol. Freshly distilled aryl acetaldehyde **2** (0.15 mmol, 3.0 equiv) was added, followed by 3 mg catalyst *rac*-**3d** (0.005 mmoL, 10 mol%) and stirred until TLC (25 % EtOAc in hexanes) showed complete conversion of **1**. The crude mixture was purified by preparative TLC to obtain the racemic product (*rac*-**4**) samples for HPLC analysis.

1.4 Typical procedure for the catalytic enantioselective [6+2]-cycloaddition of reactive 2*H*-pyrrol-2-methides with *in situ* formed 2-phenylethen-1-ols

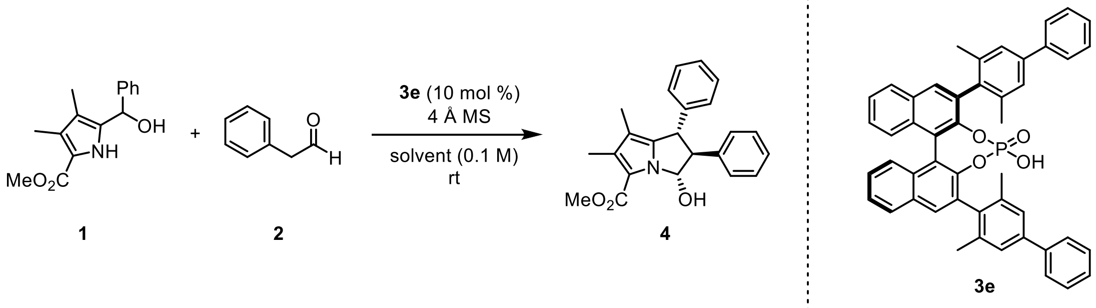

General procedure A: In a screw-cap vial, (1*H*-pyrrol-2-yl)(aryl)methanol **1** (0.20 mmol, 1.0 equiv) and pulverized 4 Å MS (60 mg, 300 mg/mmol) were suspended in 2 mL *o*-xylol. Freshly distilled aryl acetaldehyde **2** (0.60 mmol, 3.0 equiv) was added, followed by catalyst **3e** (0.02 mmoL, 10 mol%) and stirred until TLC (25 % EtOAc in hexanes) showed complete conversion of **1**. Reaction mixture was filtered over cellite and solvents were removed under reduced pressure to obtain a yellowish-brown oil. This residue was purified by silica-gel flash column chromatography (3-7 % EtOAc in hexanes) to obtain **4**. The diastereomeric ratio was determined by ^{1}H-NMR. Enantiomeric excess was determined by HPLC on a chiral stationary phase. [Note: Purification and removal of solvents should be done in the dark and at 30 °C or below to avoid degradation of the products.]

Characterization of Products

Methyl (1*S*,2*S*,3*S*)-3-hydroxy-6,7-dimethyl-1,2-diphenyl-2,3-dihydro-1*H*-pyrrolizine-5-carboxylate (4a)

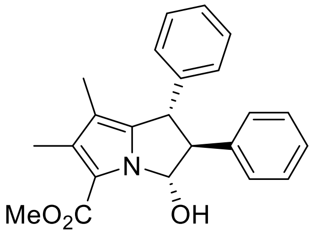

According to general procedure A: 26.0 mg (0.100 mmol) **1a**, 35.0 mg (0.300 mmol) **2a**, 21 h; Purified by silica-gel flash column chromatography (6 % EtOAc in hexanes);

Yield: 22.6 mg, 63 µmol, 62 % as an off white solid, **e.r.:** 94 : 6, **[a]$^{25}_{D}$:** = +164° (c = 1.00, $CHCl_3$).

R_f = 0.75 (Hex/EE 3:1).

m.p. = 122 – 124 °C.

^{1}H-NMR (400 MHz, $CDCl_3$): δ = 7.36 – 7.23 (m, 6H), 7.19 (tt, *J* = 7.9, 1.6 Hz, 4H), 6.06 (d, *J* = 4.9 Hz, 1H), 5.85 (s, 1H), 4.24 (d, *J* = 6.8 Hz, 1H), 3.89 (s, 3H), 3.74 (dd, *J* = 6.8, 4.9 Hz, 1H), 2.29 (s, 3H), 1.63 (s, 3H).

^{13}C-NMR (100 MHz, $CDCl_3$): δ = 164.2, 141.4, 141.1, 140.3, 133.9, 128.9, 128.8, 127.9, 127.8, 127.4, 127.1, 114.3, 112.2, 88.6, 65.7, 51.5, 51.5, 11.6, 8.6.

FT-IR (KBr): ṽ [cm^{-1}] = 3432, 2920, 1648, 1470, 1449, 1290, 1142.

UV-Vis (CH_2Cl_2): λ [nm] = 228, 257, 287.

HRMS (ESI-TOF) m/z: [M - H_2O + H]$^+$ Calcd. for $C_{23}H_{22}NO_2$ 344.1651; Found 344.1667.

HPLC IA column (90 % hexane, 10 % *iso*-propanol, 1 mL/min, 284 nm) τ_{major} = 6.7 min, τ_{minor} = 7.8 min.

Methyl (1*S*,2*S*,3*S*)-3-hydroxy-6,7-dimethyl-1-phenyl-2-(*o*-tolyl)-2,3-dihydro-1*H*-pyrrolizine-5-carboxylate (4b)

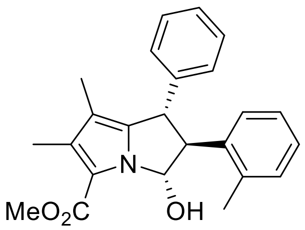

According to general procedure A: 51.8 mg (0.200 mmol) **1a**, 80.9 mg (0.603 mmol) **2b**, 21 h; Purified by silica-gel flash column chromatography (6 % EtOAc in hexanes);

Yield: 47.1 mg, 0.125 mmol, 63 % as a colorless oil, **e.r.:** 91 : 9, **[a]$^{25}_{D}$:** = +102° (c = 1.00, $CHCl_3$).

R_f = 0.80 (Hex/EE 3:1).

^{1}H-NMR (400 MHz, $CDCl_3$): δ = 7.30-7.27 (m, 1H), 7.25-7.22 (m, 2H), 7.22-7.18 (m, 1H), 7.17 (d, *J* = 1.9 Hz, 2H), 7.16 (d, *J* = 1.8 Hz, 2H), 7.15-7.13 (m, 1H), 6.05 (d, *J* = 4.5 Hz, 1H), 5.66 (s, 1H), 4.18 (d, *J* = 6.4 Hz, 1H), 4.06 (dd, *J* = 6.4, 4.5 Hz, 1H), 3.89 (s, 3H), 2.28 (s, 3H), 2.05 (s, 3H), 1.64 (s, 3H).

^{13}C-NMR (100 MHz, $CDCl_3$): δ = 164.1, 141.7, 141.5, 139.3, 136.8, 133.9, 130.6, 128.8, 127.7, 127.1, 127.1, 126.7, 126.6, 114.3, 112.2, 89.2, 61.2, 52.2, 51.5, 20.0, 11.6, 8.7.

FT-IR (thin film): ṽ [cm^{-1}] = 3433, 2952, 2924, 1656, 1448, 1281, 1139.

UV-Vis (CH_2Cl_2): λ [nm] = 228, 287.

HRMS (ESI-TOF) m/z: [M - H_2O + H]$^+$ Calcd. for $C_{24}H_{24}NO_2$ 358.1807; Found 358.1802.

HPLC IA column (90 % hexane, 10 % *iso*-propanol, 1 mL/min, 286 nm) τ_{major} = 4.9 min, τ_{minor} = 6.3 min.

Methyl (1*S*,2*S*,3*S*)-3-hydroxy-6,7-dimethyl-1-phenyl-2-(*m*-tolyl)-2,3-dihydro-1*H*-pyrrolizine-5-carboxylate (4c)

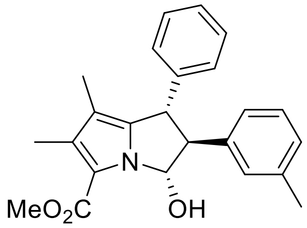

According to general procedure A: 52.0 mg (0.200 mmol) **1a**, 80.8 mg (0.602 mmol) **2c**, 27 h; Purified by silica-gel flash column chromatography (5 % EtOAc in hexanes);

Yield: 46.3 mg, 0.123 mmol, 62 % as a colorless oil, **e.r.:** 93.5 : 6.5, **$[a]^{25}_{D}$:** = +168° (c = 1.00, $CHCl_3$).

R_f = 0.79 (Hex/EE 3:1).

^{1}H-NMR (400 MHz, $CDCl_3$): δ = 7.32-7.26 (m, 2H), 7.24 (d, *J* = 1.2 Hz, 1H), 7.21 (d, *J* = 7.6 Hz, 1H), 7.19-7.15 (m, 2H), 7.09 (ddt, *J* = 7.5, 1.8, 1.0 Hz, 1H), 7.03-6.97 (m, 2H), 6.03 (dd, *J* = 4.9, 1.8 Hz, 1H), 5.81 (s, 1H), 4.23 (d, *J* = 6.8 Hz, 1H), 3.89 (s, 3H), 3.68 (dd, *J* = 6.8, 4.9 Hz, 1H), 2.33 (s, 3H), 2.28 (s, 3H), 1.62 (s, 3H).

^{13}C-NMR (100 MHz, $CDCl_3$): δ = 164.2, 141.5, 141.3, 140.3, 138.6, 133.9, 128.9, 128.8, 128.6, 128.2, 127.9, 127.1, 124.8, 114.3, 112.2, 88.8, 65.8, 51.5, 51.5, 21.6, 11.6, 8.7.

FT-IR (KBr): ṽ [cm^{-1}] = 3376, 2953, 2923, 1646, 1447, 1329, 1282, 1187, 1143.

UV-Vis (CH_2Cl_2): λ [nm] = 228, 288.

HRMS (ESI-TOF) m/z: [M - H_2O + H]$^+$ Calcd. for $C_{24}H_{24}NO_2$ 358.1813; Found 358.1814.

HPLC IA column (98 % hexane, 2 % *iso*-propanol, 1 mL/min, 284 nm) τ_{minor} = 8.2 min, τ_{major} = 9.2 min.

Methyl (1*S*,2*S*,3*S*)-3-hydroxy-6,7-dimethyl-1-phenyl-2-(*p*-tolyl)-2,3-dihydro-1*H*-pyrrolizine-5-carboxylate (4d)

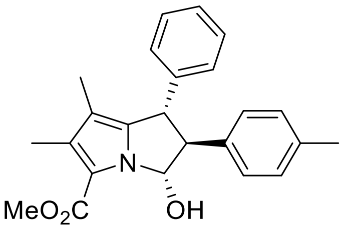

According to general procedure A: 52.3 mg (0.202 mmol) **1a**, 80.3 mg (0.599 mmol) **2d**, 27 h; Purified by silica-gel flash column chromatography (5 % EtOAc in hexanes);

Yield: 44.4 mg, 0.118 mmol, 59 % as a colorless oil, **e.r.:** 93.5 : 6.5, **$[a]^{25}_{D}$:** +196° (c = 1.00, $CHCl_3$).

R_f = 0.82 (Hex/EE 3:1).

^{1}H-NMR (400 MHz, $CDCl_3$): δ = 7.31-7.26 (m, 2H), 7.26-7.23 (m, 1H), 7.18 (d, *J* = 1.7 Hz, 1H), 7.16-7.12 (m, 3H), 7.10-7.07 (m, 2H), 6.01 (dd, *J* = 5.0, 2.0 Hz, 1H), 5.81 (s, 1H), 4.21 (d, *J* = 6.9 Hz, 1H), 3.89 (s, 3H), 3.69 (dd, *J* = 6.9, 4.9 Hz, 1H), 2.34 (s, 3H), 2.28 (s, 3H), 1.62 (s, 3H).

^{13}C-NMR (100 MHz, $CDCl_3$): δ = 164.3, 141.6, 141.3, 137.4, 137.2, 133.9, 129.7, 128.9, 128.0, 127.8, 127.2, 114.4, 112.3, 88.9, 65.5, 51.6, 51.6, 21.3, 11.7, 8.8.

FT-IR (KBr): ṽ [cm^{-1}] = 3445, 3265, 2952, 2922, 1654, 1452, 1327, 1288, 1145.

UV-Vis (CH_2Cl_2): λ [nm] = 228, 287.

HRMS (ESI-TOF) m/z: $[M - H_2O + H]^+$ Calcd. for $C_{24}H_{24}NO_2$ 358.1813; Found 358.1807.

HPLC IA column (90 % hexane, 10 % *iso*-propanol, 1 mL/min, 284 nm) τ_{major} = 7.9 min, τ_{minor} = 9.2 min.

Methyl (1*S*,2*S*,3*S*)-2-(2-fluorophenyl)-3-hydroxy-6,7-dimethyl-1-phenyl-2,3-dihydro-1*H*-pyrrolizine-5-carboxylate (4e)

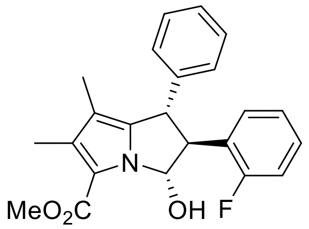

According to general procedure A: 52.2 mg (0.201 mmol) **1a**, 80.7 mg (0.584 mmol) **2e**, 5 h; Purified by silica-gel flash column chromatography (5 % EtOAc in hexanes);

Yield: 32.8 mg, 0.086 mmol, 43 % as a colorless oil, **e.r.:** 93.5 : 6.5, **$[a]^{25}_{D}$:** = +162° (c = 1.00, $CHCl_3$).

R_f = 0.80 (Hex/EE 3:1).

^{1}H-NMR (400 MHz, $CDCl_3$): δ = 7.31-7.26 (m, 3H), 7.26-7.21 (m, 1H), 7.15-7.12 (m, 2H), 7.11-7.04 (m, 3H), 6.15 (d, *J* = 5.1 Hz, 1H), 5.86 (s, 1H), 4.30 (d, *J* = 7.1 Hz, 1H), 3.89 (s, 1H), 3.82 (dd, *J* = 7.2, 5.1 Hz, 1H), 2.28 (s, 3H), 1.62 (s, 3H).

^{19}F-NMR (376 MHz, $CDCl_3$): δ = 115.7.

^{13}C-NMR (100 MHz, $CDCl_3$): δ = 164.1, 161.0 (d, *J* = 246.5 Hz), 141.1, 140.8, 133.7, 130.6 (d, *J* = 4.6 Hz), 129.2 (d, *J* = 8.4 Hz), 128.7, 127.7, 127.1, 126.8 (d, *J* = 13.4 Hz), 124.5 (d, *J* = 3.5 Hz), 116.0 (d, *J* = 21.8 Hz), 114.3, 112.2, 87.2 (d, *J* = 3.5 Hz), 61.3, 51.4, 50.1 (d, *J* = 2.5 Hz), 11.5, 8.6.

FT-IR (KBr): ṽ [cm^{-1}] = 3420, 2952, 2921, 1659, 1493, 1450, 1283, 1139.

UV-Vis (CH_2Cl_2): λ [nm] = 228, 287.

HR-MS (ESI): calc. for: $C_{23}H_{22}FNO_3^+$ ($[M\text{-}H_2O+H]^+$): 362.1562, found: 362.1574.

HPLC IA column (90 % hexane, 10 % *iso*-propanol, 1 mL/min, 284 nm) τ_{major} = 6.4 min, τ_{minor} = 8.0 min.

Methyl (1*S*,2*S*,3*S*)-2-(4-bromophenyl)-3-hydroxy-6,7-dimethyl-1-phenyl-2,3-dihydro-1*H*-pyrrolizine-5-carboxylate (4f)

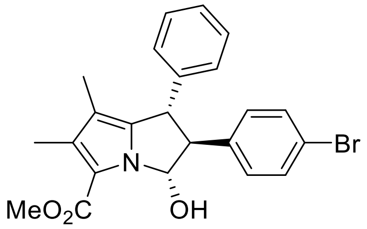

According to general procedure A: 51.6 mg (0.199 mmol) **1a**, 119.5 mg (0.601 mmol) **2g**, 7 h; Purified by silica-gel flash column chromatography (4 % EtOAc in hexanes);

Yield: 53.7 mg, 0.122 mmol, 61 % as a colorless oil, **e.r.:** 90 : 10, **$[a]^{25}_{D}$** = +150° (c = 0.98, $CHCl_3$).

R_f = 0.81 (Hex/EE 7:1).

^{1}H-NMR (400 MHz, $CDCl_3$): δ = 7.45 (dt, *J* = 8.5, 1.8 Hz, 2H), 7.32 – 7.28 (m, 1H), 7.28 – 7.24 (m, 2H), 7.15 (dt, *J* = 6.5, 1.7 Hz, 2H), 7.08 (dt, *J* = 8.4, 1.8 Hz, 2H), 5.99 (d, *J* = 5.1 Hz, 1H), 5.91 (s, 1H), 4.17 (d, *J* = 7.1 Hz, 1H), 3.89 (s, 3H), 3.68 (dd, *J* = 7.2, 5.1 Hz, 1H), 2.27 (s, 3H), 1.61 (s, 3H).

^{13}C-NMR (100 MHz, $CDCl_3$): δ = 164.2, 140.9, 140.7, 139.1, 134.0, 132.1, 129.6, 128.9, 127.9, 127.3, 121.4, 114.5, 112.4, 88.3, 65.3, 51.6, 51.4, 11.6, 8.6.

FT-IR (KBr): ṽ [cm^{-1}] = 3436, 2951, 2925, 1656, 1471, 1450, 1284, 1143.

UV-Vis (CH_2Cl_2): λ [nm] = 231, 287.

HRMS (ESI-TOF) m/z: $[M - H_2O + H]^+$ Calcd. for $C_{23}H_{21}BrNO_2$ 422.0761; Found 422.0734.

HPLC IE column (90 % hexane, 10 % *iso*-propanol, 1 mL/min, 284 nm) τ_{major} = 8.9 min, τ_{minor} = 15.1 min.

Methyl (1*S*,2*S*,3*S*)-2-(3-bromophenyl)-3-hydroxy-6,7-dimethyl-1-phenyl-2,3-dihydro-1*H*-pyrrolizine-5-carboxylate (4g)

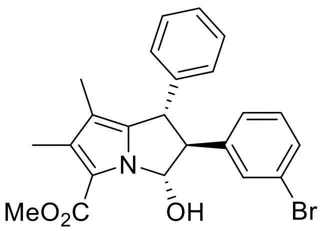

According to general procedure A: 51.8 mg (0.200 mmol) **1a**, 119 mg (0.598 mmol) **2f**, 5 h; Purified by silica-gel flash column chromatography (4 % EtOAc in hexanes);

Yield: 47.3 mg, 0.107 mmol, 54 % as an orange solid, **e.r.:** 94.5 : 5.5, **$[a]^{25}_{D}$:** = +138° (c = 1.00, $CHCl_3$).

R_f = 0.88 (Hex/EE 3:1).

m.p. = 97- 101 °C.

^{1}H-NMR (400 MHz, $CDCl_3$): δ = 7.42 (ddd, *J* = 7.9, 2.0, 1.1 Hz, 1H), 7.37 (t, *J* = 1.8 Hz, 1H), 7.33-7.27 (m, 2H), 7.25-7.21 (m, 1H), 7.20-7.16 (m, 2H), 7.14 (d, *J* = 1.3 Hz, 1H), 7.10 (dt, *J* = 7.8, 1.4 Hz, 1H), 6.00 (d, *J* = 5.0 Hz, 1H), 5.88 (s, 1H), 4.20 (d, *J* = 6.9 Hz, 1H), 3.89 (s, 3H), 3.68 (dd, *J* = 7.0, 4.9 Hz, 1H), 2.27 (s, 3H), 1.60 (s, 3H).

^{13}C-NMR (100 MHz, $CDCl_3$): δ = 164.2, 142.6, 141.0, 140.7, 134.1, 130.8, 130.7, 130.5, 128.9, 127.9, 127.4, 126.7, 123.1, 114.5, 112.4, 88.4, 65.4, 51.6, 51.4, 11.6, 8.6.

FT-IR (thin film): ṽ [cm^{-1}] = 3437, 2951, 2922, 1657, 1475, 1449, 1282, 1141, 757.

UV-Vis (CH_2Cl_2): λ [nm] = 228, 287.

HRMS (ESI-TOF) m/z: $[M - H_2O + H]^+$ Calcd. for $C_{23}H_{21}BrNO_2$ 422.0761; Found 422.0745.

HPLC IE column (90 % hexane, 10 % *iso*-propanol, 1 mL/min, 284 nm) τ_{major} = 8.4 min, τ_{minor} = 9.2 min.

Methyl (1*S*,2*S*,3*S*)-3-hydroxy-2-(3-methoxyphenyl)-6,7-dimethyl-1-phenyl-2,3-dihydro-1*H*-pyrrolizine-5-carboxylate (4h)

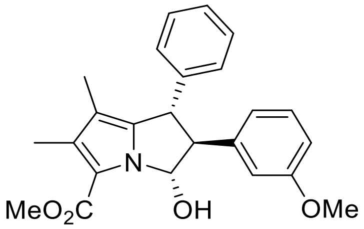

According to general procedure A: 51.9 mg (0.200 mmol) **1a**, 99.0 mg (0.659 mmol) **2h**, 7 h; Purified by silica-gel flash column chromatography (4-5 % EtOAc in hexanes);

Yield: 44.4 mg, 0.113 mmol, 57 % as a colorless oil, **e.r.:** 91 : 9, **$[a]^{25}_{D}$:** = +142° (c = 1.00, $CHCl_3$).

R_f = 0.61 (Hex/EE 3:1).

^{1}H-NMR (400 MHz, $CDCl_3$): δ = 7.32-7.26 (m, 2H), 7.25-7.22 (m, 1H), 7.18-7.15 (m, 2H), 6.82 (dt, *J* = 8.3, 1.6 Hz, 1H), 6.80-6.76 (m, 1H), 6.72 (p, *J* = 1.1 Hz, 1H), 6.03 (dd, *J* = 5.0, 2.0 Hz, 1H), 5.81 (s, 1H), 4.22 (d, *J* = 6.8 Hz, 1H), 3.89 (s, 3H), 3.77 (s, 3H), 3.69 (dd, *J* = 6.7, 5.0 Hz, 1H), 2.27 (s, 3H), 1.61 (s, 3H).

^{13}C-NMR (100 MHz, $CDCl_3$): δ = 164.2, 160.0, 142.0, 141.4, 141.2, 133.9, 130.0, 128.8, 127.9, 127.2, 120.0, 114.4, 113.7, 112.7, 112.3, 88.6, 65.7, 55.4, 51.5, 51.4, 11.6, 8.7.

FT-IR (thin film): ṽ [cm^{-1}] = 3442, 2952, 2921, 1657, 1467, 1450, 1283, 1140, 757.

UV-Vis (CH_2Cl_2): λ [nm] = 229, 284.

HRMS (ESI-TOF) m/z: [M - H_2O + H]$^+$ Calcd. for $C_{24}H_{24}NO_3$ 374.1762; Found 374.1745.

HPLC IA column (98 % hexane, 2 % *iso*-propanol, 0.5 mL/min, 284 nm) τ_{minor} = 28.6 min, τ_{major} = 30.7 min.

Methyl (1*S*,2*S*,3*S*)-3-hydroxy-2-(4-methoxyphenyl)-6,7-dimethyl-1-phenyl-2,3-dihydro-1*H*-pyrrolizine-5-carboxylate (4i)

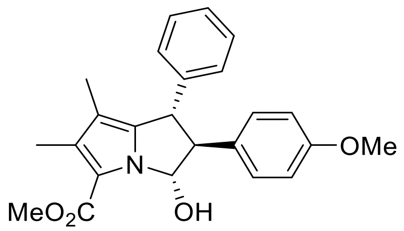

According to general procedure A: 51.7 mg (0.199 mmol) **1a**, 99.0 mg (0.659 mmol) **2i**, 5 h; Purified by silica-gel flash column chromatography (4-5 % EtOAc in hexanes);

Yield: 51.0 mg, 0.130 mmol, 65 % as a colorless oil, **e.r.:** 95 : 5, **[a]$^{24}_{D}$:** = +178° (c = 1.00, $CHCl_3$).

R_f = 0.77 (Hex/EE 3:1).

^{1}H-NMR (400 MHz, $CDCl_3$): δ = 7.30-7.22 (m, 3H), 7.15 (dd, *J* = 6.7, 1.7 Hz, 1H), 7.11 (d, *J* = 8.7 Hz, 1H), 6.88-6.83 (m, 2H), 5.98 (dd, *J* = 5.1, 1.8 Hz, 1H), 5.86 (s, 1H), 4.18 (d, *J* = 7.1 Hz, 1H), 3.89 (s, 3H), 3.80 (s, 3H), 3.65 (dd, *J* = 7.2, 5.2 Hz, 1H), 2.27 (s, 3H), 1.61 (s, 3H).

^{13}C-NMR (100 MHz, $CDCl_3$): δ = 164.2, 159.0, 141.3, 141.1, 133.8, 132.2, 128.9, 128.8, 127.9, 127.1, 114.4, 114.3, 112.3, 88.7, 65.1, 55.4, 51.6, 51.5, 11.6, 8.7.

FT-IR (KBr): ṽ [cm^{-1}] = 3434, 2953, 2916, 1656, 1515, 1451, 1285, 1143.

UV-Vis (CH_2Cl_2): λ [nm] = 230, 286.

HRMS (ESI-TOF) m/z: [M - H_2O + H]$^+$ Calcd. for $C_{24}H_{24}NO_3$ 374.1762; Found 374.1751.

HPLC IA column (90 % hexane, 10 % *iso*-propanol, 0.5 mL/min, 284 nm) τ_{major} = 20.7 min, τ_{minor} = 22.8 min.

Methyl (1*S*,2*S*,3*S*)-3-hydroxy-6,7-dimethyl-1-phenyl-2-(4-(trifluoromethyl)phenyl)-2,3-dihydro-1*H*-pyrrolizine-5-carboxylate (4j)

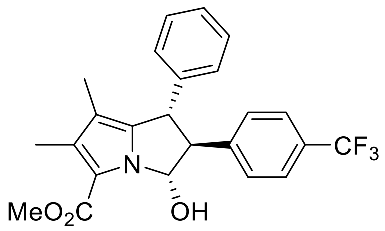

According to general procedure A: 52.0 mg (0.200 mmol) **1a**, 116 mg (0.617 mmol) **2j**, 5 h; Purified by silica-gel flash column chromatography (4 % EtOAc in hexanes);

Yield: 48.5 mg, 0.113 mmol, 56 % as a colorless oil, **e.r.:** 85 : 15, **[a]$^{25}_{D}$:** = +122° (c = 1.00, $CHCl_3$).

R$_f$ = 0.80 (Hex/EE 3:1).

^{1}H-NMR (400 MHz, $CDCl_3$): δ = 7.59 (d, *J* = 8.1 Hz, 2H), 7.34-7.29 (m, 3H), 7.29-7.25 (m, 2H), 7.16 (d, *J* = 1.8 Hz, 1H), 7.14 (d, *J* = 1.4 Hz, 1H), 6.03 (d, *J* = 5.0 Hz, 1H), 5.91 (s, 1H), 4.21 (d, *J* = 7.0 Hz, 1H), 3.90 (s, 3H), 3.79 (dd, *J* = 7.0, 5.0 Hz, 1H), 2.27 (s, 3H), 1.61 (s, 3H).

^{19}F-NMR (376 MHz, $CDCl_3$): δ = 62.6.

^{13}C-NMR (100 MHz, $CDCl_3$): δ = 164.3, 144.2, 140.9, 140.6, 134.1, 129.8 (d, *J* = 32.6 Hz), 129.0, 128.3, 127.9, 127.4, 126.0 (q, J = 3.8 Hz), 124.2 (q, *J* = 272.1 Hz), 114.6, 112.5, 88.3, 65.5, 51.6, 51.4, 11.6, 8.6.

FT-IR (thin film): ṽ [cm^{-1}] = 3435, 2958, 2925, 1656, 1470, 1453, 1328, 1291, 1146.

UV-Vis (CH_2Cl_2): λ [nm] = 229, 287.

HRMS (ESI-TOF) m/z: $[M - H_2O + H]^+$ Calcd. for $C_{24}H_{21}F_3NO_2$ 412.1530; Found 412.1520.

HPLC IE column (90 % hexane, 10 % *iso*-propanol, 1 mL/min, 284 nm) τ_{major} = 6.2 min, τ_{minor} = 8.6 min.

Methyl (1*S*,2*S*,3*S*)-6-ethyl-3-hydroxy-7-methyl-1,2-diphenyl-2,3-dihydro-1*H*-pyrrolizine-5-carboxylate (4k)

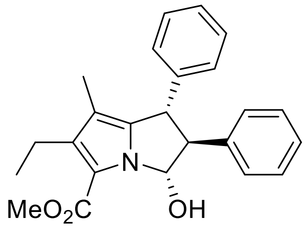

According to general procedure A: 56.4 mg (0.260 mmol) **1k**, 72.1 mg (0.600 mmol) **2a**, 5 h; Purified by silica-gel flash column chromatography (4 % EtOAc in hexanes);

Yield: 44.4 mg, 0.117 mmol, 57 % as a colorless oil, **e.r.:** 91.5 : 8.5, **[a]$^{25}_{D}$:** +168° (c = 1.00, $CHCl_3$).

R$_f$ = 0.77 (Hex/EE 3:1).

^{1}H-NMR (400 MHz, $CDCl_3$): δ = 7.35-7.23 (m, 6H), 7.22-7.18 (m, 2H), 7.16 (dt, *J* = 7.0, 1.5 Hz, 2H), 6.04 (ddd, *J* = 4.9, 2.1, 1.1 Hz, 1H), 5.87 (d, *J* = 2.1 Hz, 1H), 4.23 (d, *J* = 7.0 Hz, 1H), 3.89 (d, *J* = 1.1 Hz, 3H), 3.71 (td, *J* = 6.3, 5.5, 1.7 Hz, 1H), 2.81-2.66 (m, 2H), 1.63 (s, 3H), 1.14 (td, *J* = 7.4, 1.1 Hz, 3H).

^{13}C-NMR (100 MHz, $CDCl_3$): δ = 164.1, 141.4, 141.1, 140.6, 140.2, 129.0, 128.8, 128.0, 127.9, 127.5, 127.2, 113.6, 111.5, 88.7, 65.9, 51.5, 51.5, 19.4, 15.3, 8.5.

FT-IR (KBr): ṽ [cm^{-1}] = 3435, 2954, 2921, 1651, 1471, 1455, 1282, 1138.

UV-Vis (CH_2Cl_2): λ [nm] = 228, 288.

HRMS (ESI-TOF) m/z: [M - H_2O + H]$^+$ Calcd. for $C_{24}H_{24}NO_2$ 358.1813; Found 358.1813.

HPLC IA column (90 % hexane, 10 % *iso*-propanol, 0.5 mL/min, 284 nm) τ_{major} = 12.0 min, τ_{minor} = 12.9 min.

Methyl (1*S*,2*S*,3*S*)-3-hydroxy-1,2-diphenyl-2,3,6,7,8,9-hexahydro-1*H*-pyrrolo[2,1-*a*]isoindole-5-carboxylate (4l)

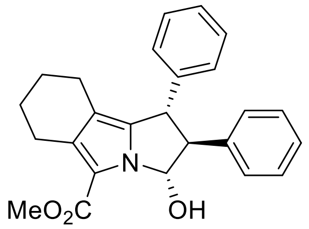

According to general procedure A: 56.9 mg (0.200 mmol) **1l**, 72.1 mg (0.600 mmol) **2a**, 4.5 h; Purified by silica-gel flash column chromatography (4-7 % EtOAc in hexanes);

Yield: 40.5 mg, 0.105 mmol, 52 % as a colorless oil, **e.r.:** 93 : 7, **[a]$^{24}_{D}$:** = +153° (c = 0.68, $CHCl_3$).

R_f = 0.80 (Hex/EE 3:1).

^{1}H-NMR (400 MHz, $CDCl_3$): δ = 7.36-7.27 (m, 5H), 7.26-7.20 (m, 3H), 7.18-7.14 (m, 2H), 6.07 (dd, *J* = 5.3, 1.5 Hz, 1H), 5.94 (s, 1H), 4.24 (d, *J* = 7.4 Hz, 1H), 3.87 (s, 3H), 3.74 (dd, *J* = 7.4, 5.3 Hz, 1H), 2.83 (td, *J* = 6.3, 2.1 Hz, 2H), 2.13 (dt, *J* = 15.7, 5.9 Hz, 1H), 1.95 (dt, *J* = 15.5, 6.4 Hz, 1H), 1.72 (qdt, *J* = 9.7, 6.6, 3.8 Hz, 1H), 1.65-1.57 (m, 2H).

^{13}C-NMR (100 MHz, $CDCl_3$): δ = 164.0, 140.5, 140.0, 139.9, 135.7, 129.0, 128.8, 127.9, 127.9, 127.5, 127.2, 114.1, 113.0, 88.5, 65.8, 51.5, 51.4, 24.4, 23.2, 23.1, 21.1.

FT-IR (KBr): ṽ [cm^{-1}] = 3425, 2926, 2851, 1655, 1469, 1452, 1276, 1128.

UV-Vis (CH_2Cl_2): λ [nm] = 228, 289.

HRMS (ESI-TOF) m/z: [M - H_2O + H]$^+$ Calcd. for $C_{25}H_{24}NO_2$ 370.1813; Found 370.1808.

HPLC IA column (90 % hexane, 10 % *iso*-propanol, 1 mL/min, 284 nm) τ_{major} = 5.9 min, τ_{minor} = 6.8 min.

Methyl (1*S*,2*S*,3*S*)-3-hydroxy-2-(4-methoxyphenyl)-6,7-dimethyl-1-(naphthalen-2-yl)-2,3-dihydro-1*H*-pyrrolizine-5-carboxylate (4m)

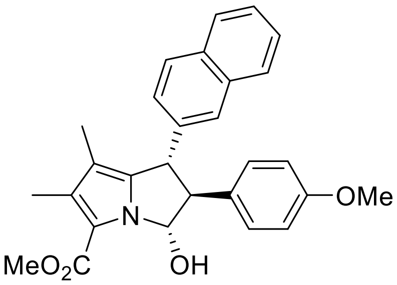

According to general procedure A: 62.4 mg (0.202 mmol) **1m**, 94.0 mg (0.626 mmol) **2i**, 6 h; Purified by silica-gel flash column chromatography (4-5 % EtOAc in hexanes);

Yield: 58.5 mg, 0.132 mmol, 66 % as a white foam, **e.r.:** 98.5 : 1.5, **$[a]^{24}_{D}$:** = +276° (c = 0.50, $CHCl_3$).

R_f = 0.61 (Hex/EE 3:1).

m.p. = 67 – 69 °C.

^{1}H-NMR (400 MHz, $CDCl_3$): δ = 7.83-7.77 (m, 2H), 7.77-7.72 (m, 1H), 7.59 (d, *J* = 1.8 Hz, 1H), 7.45 (dt, *J* = 6.2, 3.4 Hz, 2H), 7.31 (dd, *J* = 8.5, 1.8 Hz, 1H), 7.13 (dt, *J* = 8.7, 1.9 Hz, 2H), 6.86 (dt, *J* = 8.7, 2.0 Hz, 2H), 6.04 (d, *J* = 5.2 Hz, 1H), 5.92 (s, 1H), 4.35 (d, *J* = 7.2 Hz, 1H), 3.91 (s, 3H), 3.80 (s, 3H), 3.75 (dd, *J* = 7.2, 5.1 Hz, 1H), 2.28 (s, 3H), 1.59 (s, 3H).

^{13}C-NMR (100 MHz, $CDCl_3$): δ = 164.3, 159.0, 141.2, 138.5, 133.9, 133.6, 132.8, 132.1, 129.0, 128.8, 127.9, 127.8, 126.6, 126.2, 126.1, 125.8, 114.5, 114.4, 112.5, 88.8, 65.1, 55.4, 51.8, 51.6, 11.7, 8.7.

FT-IR (KBr): ṽ [cm^{-1}] = 3434, 2951, 2922, 1658, 1513, 1449, 1281, 1251, 1139.

UV-Vis (CH_2Cl_2): λ [nm] = 229, 287.

HRMS (ESI-TOF) m/z: [M - H_2O + H]$^+$ Calcd. for $C_{28}H_{26}NO_3$ 424.1918; Found 424.1925.

HPLC IA column (90 % hexane, 10 % *iso*-propanol, 1 mL/min, 284 nm) τ_{minor} = 13.0 min, τ_{major} = 15.2 min.

Methyl (1*S*,2*S*,3*S*)-3-hydroxy-1,2-bis(4-methoxyphenyl)-6,7-dimethyl-2,3-dihydro-1*H*-pyrrolizine-5-carboxylate (4n)

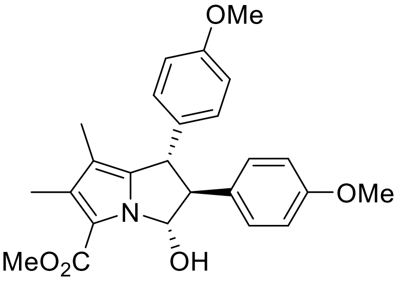

According to general procedure A: 58.0 mg (0.200 mmol) **1n**, 94.0 mg (0.626 mmol) **2i**, 6 h; Purified by silica-gel flash column chromatography (4 % EtOAc in hexanes);

Yield: 51.1 mg, 0.121 mmol, 61 % as a colorless oil, **e.r.:** 96.5 : 3.5, **[a]$^{25}_{D}$:** = +226° (c = 1.00, $CHCl_3$).

R_f = 0.66 (Hex/EE 3:1).

^{1}H-NMR (400 MHz, $CDCl_3$): δ = 7.11 (dt, *J* = 8.6, 1.9 Hz, 2H), 7.07 (dt, *J* = 8.3, 2.0 Hz, 2H), 6.85 (dt, *J* = 8.7, 2.0 Hz, 2H), 6.81 (dt, *J* = 8.7, 2.0 Hz, 2H), 5.97 (dd, *J* = 5.2, 2.0 Hz, 1H), 5.88 (bs, 1H), 4.13 (d, *J* = 7.2 Hz, 1H), 3.88 (s, 3H), 3.79 (s, 3H), 3.78 (s, 3H), 3.61 (dd, *J* = 7.3, 5.2 Hz, 1H), 2.27 (s, 3H), 1.62 (s, 3H).

^{13}C-NMR (100 MHz, $CDCl_3$): δ = 164.2, 159.0, 158.7, 141.6, 133.8, 133.2, 132.2, 128.9, 128.9, 114.3, 114.1, 112.2, 88.6, 65.3, 55.4, 55.3, 51.5, 50.9, 11.6, 8.6.

FT-IR (thin film): ṽ [cm^{-1}] = 3434, 3012, 2953, 1658, 1514, 1448, 1281, 1250, 1140.

UV-Vis (CH_2Cl_2): λ [nm] = 232, 287.

HRMS (ESI-TOF) m/z: [M - H_2O + H]$^+$ Calcd. for $C_{25}H_{26}NO_4$ 404.1867; Found 404.1872.

HPLC IA column (90 % hexane, 10 % *iso*-propanol, 1 mL/min, 284 nm) τ_{minor} = 12.3 min, τ_{major} = 16.6 min.

Methyl (1*S*,2*S*,3*S*)-1-(benzo[*d*][1,3]dioxol-5-yl)-3-hydroxy-2-(4-methoxyphenyl)-6,7-dimethyl-2,3-dihydro-1*H*-pyrrolizine-5-carboxylate (4o)

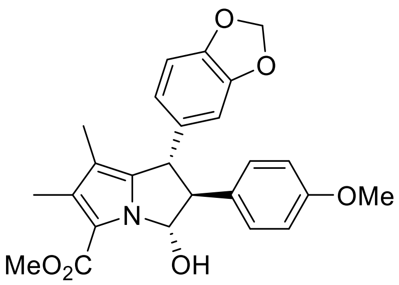

According to general procedure A: 61.1 mg (0.202 mmol) **1o**, 91.0 mg (0.606 mmol) **2i**, 3.5 h; Purified by silica-gel flash column chromatography (4-5 % EtOAc in hexanes);

Yield: 61.9 mg, 0.142 mmol, 70 % as a colorless oil, **e.r.:** 96.5 : 3.5, **[a]$^{24}_{D}$:** = +246° (c = 1.00, $CHCl_3$).

R_f = 0.67 (Hex/EE 3:1).

^{1}H-NMR (400 MHz, $CDCl_3$): δ = 7.11 (dt, *J* = 8.7, 2.0 Hz, 2H), 6.86 (dt, *J* = 8.7, 2.0 Hz, 2H), 6.70 (d, *J* = 8.0 Hz, 1H), 6.67 (d, *J* = 1.8 Hz, 1H), 6.59 (dd, *J* = 8.0, 1.8 Hz, 1H), 5.95 (dd, *J* = 5.2, 1.4 Hz, 1H), 5.93 (s, 1H), 5.83 (s, 1H), 4.10 (d, *J* = 7.0 Hz, 1H), 3.88 (s, 3H), 3.80 (s, 3H), 3.60 (dd, *J* = 7.1, 5.1 Hz, 1H), 2.27 (s, 3H), 1.65 (s, 3H).

^{13}C-NMR (100 MHz, $CDCl_3$): δ = 164.2, 159.0, 148.1, 146.7, 141.3, 135.1, 133.8, 132.2, 128.8, 121.1, 114.3, 112.2, 108.3, 108.2, 101.1, 88.6, 65.2, 55.4, 51.5, 51.3, 11.6, 9.3.

FT-IR (KBr): ṽ [cm^{-1}] = 3417, 2952, 2917, 1659, 1488, 1444, 1282, 1249, 1142.

UV-Vis (CH_2Cl_2): λ [nm] = 231, 287.

HRMS (ESI-TOF) m/z: [M - H_2O + H]$^+$ Calcd. for $C_{25}H_{24}NO_5$ 418.1660; Found 418.1653.

HPLC IA column (90 % hexane, 10 % *iso*-propanol, 1 mL/min, 284 nm) τ_{minor} = 14.3 min, τ_{major} = 20.0 min.

Methyl (1*S*,2*S*,3*S*)-1-(3,5-dimethylphenyl)-3-hydroxy-2-(4-methoxyphenyl)-6,7-dimethyl-2,3-dihydro-1*H*-pyrrolizine-5-carboxylate (4p)

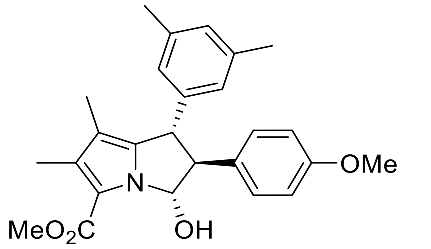

According to general procedure A: 58.1 mg (0.202 mmol) **1p**, 91.0 mg (0.606 mmol) **2i**, 5 h; Purified by silica-gel flash column chromatography (4 % EtOAc in hexanes);

Yield: 58.7 mg, 0.140 mmol, 69 % as a colorless oil, **e.r.:** 98 : 2, **$[a]^{24}_{D}$:** = +232° (c = 0.50, $CHCl_3$).

R_f = 0.86 (Hex/EE 3:1).

^{1}H-NMR (400 MHz, $CDCl_3$): δ = 7.13 (dt, *J* = 8.7, 3.0 Hz, 2H), 6.88 (d, *J* = 2.3 Hz, 2H), 6.86 (s, 1H), 6.77 (s, 2H), 5.97 (dd, *J* = 5.2, 1.8 Hz, 1H), 5.89 (s, 1H), 4.12 (d, *J* = 7.1 Hz, 1H), 3.89 (s, 3H), 3.80 (s, 3H), 3.68 (dd, *J* = 7.2, 5.1 Hz, 1H), 2.29 (s, 3H), 2.26 (s, 6H), 1.63 (s, 3H).

^{13}C-NMR (100 MHz, $CDCl_3$): δ = 164.2, 158.9, 141.7, 140.9, 138.2, 138.2, 133.8, 132.4, 128.9, 128.8, 125.7, 114.3, 114.2, 112.2, 88.8, 65.0, 55.4, 51.5, 51.4, 21.4, 21.4, 11.6, 8.7.

FT-IR (KBr): ṽ [cm^{-1}] = 3427, 2951, 2919, 1655, 1611, 1515, 1447, 1278, 1139.

UV-Vis (CH_2Cl_2): λ [nm] = 231, 286.

HRMS (ESI-TOF) m/z: $[M - H_2O + H]^+$ Calcd. for $C_{26}H_{28}NO_3$ 402.2075; Found 402.2070.

HPLC IE column (90 % hexane, 10 % *iso*-propanol, 1 mL/min, 284 nm) τ_{major} = 13.0 min, τ_{minor} = 24.3 min.

Methyl (1*S*,2*S*,3*S*)-1-(4-(*tert*-butyl)phenyl)-3-hydroxy-2-(4-methoxyphenyl)-6,7-dimethyl-2,3-dihydro-1*H*-pyrrolizine-5-carboxylate (4q)

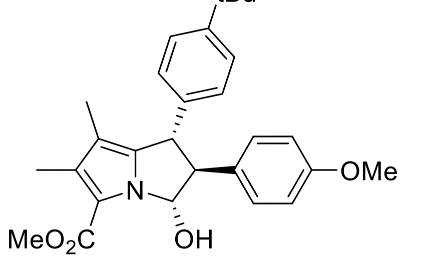

According to general procedure A: 62.4 mg (0.198 mmol) **1q**, 91.0 mg (0.606 mmol) **2i**, 3.5 h; Purified by silica-gel flash column chromatography (4 % EtOAc in hexanes);

Yield: 56.8 mg, 0.127 mmol, 64 % as a colorless oil, **e.r.:** 98.5 : 1.5, **$[a]^{24}_{D}$:** = +234° (c = 1.00, $CHCl_3$).

R_f = 0.79 (Hex/EE 3:1).

^{1}H-NMR (400 MHz, $CDCl_3$): δ = 7.28 (dt, *J* = 8.4, 1.8 Hz, 2H), 7.13 (dt, *J* = 8.7, 2.0 Hz, 2H), 7.08 (dt, *J* = 8.3, 1.8 Hz, 2H), 6.86 (dt, *J* = 8.7, 2.0 Hz, 2H), 5.96 (d, *J* = 4.8 Hz, 1H), 5.78 (s, 1H), 4.18 (d, *J* = 6.7 Hz, 1H), 3.88 (s, 3H), 3.80 (s, 3H), 3.67 (dd, *J* = 6.7, 4.8 Hz, 1H), 2.27 (s, 3H), 1.64 (s, 3H), 1.30 (s, 9H).

^{13}C-NMR (100 MHz, $CDCl_3$): δ = 164.2, 158.9, 149.8, 141.7, 138.0, 133.8, 132.7, 128.8, 127.4, 125.6, 114.3, 114.2, 112.3, 88.8, 64.8, 55.4, 51.5, 51.0, 34.6, 31.5, 11.6, 8.7.

FT-IR (KBr): ṽ [cm^{-1}] = 3420, 2956, 1656, 1515, 1449, 1283, 1252, 1141.

UV-Vis (CH_2Cl_2): λ [nm] = 231, 286.

HRMS (ESI-TOF) m/z: [M - H_2O + H]$^+$ Calcd. for $C_{28}H_{32}NO_3$ 430.2388; Found 430.2386.

HPLC IE column (90 % hexane, 10 % *iso*-propanol, 1 mL/min, 284 nm) τ_{major} = 11.7 min, τ_{minor} = 20.5 min.

Methyl (1*S*,2*S*,3*S*)-3-hydroxy-2-(4-methoxyphenyl)-6,7-dimethyl-1-(*p*-tolyl)-2,3-dihydro-1*H*-pyrrolizine-5-carboxylate (4r)

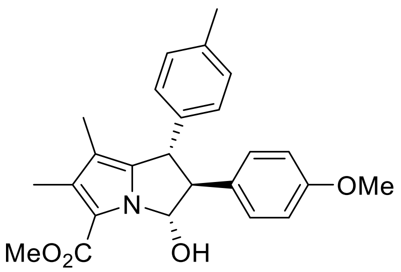

According to general procedure A: 54.8 mg (0.200 mmol) **1r**, 91.0 mg (0.606 mmol) **2i**, 3.5 h; Purified by silica-gel flash column chromatography (4-5 % EtOAc in hexanes);

Yield: 48.7 mg, 0.120 mmol, 60 % as a colorless oil, **e.r.:** 96.5 : 3.5, **[a]$^{25}_{D}$:** = +194° (c = 1.00, $CHCl_3$).

R_f = 0.65 (Hex/EE 3:1).

^{1}H-NMR (400 MHz, $CDCl_3$): δ = 7.12 (dt, *J* = 8.7, 2.0 Hz, 2H), 7.09 (dt, *J* = 8.1, 2.1 Hz, 2H), 7.04 (dt, *J* = 8.2, 1.9 Hz, 2H), 6.86 (dt, *J* = 8.7, 2.1 Hz, 2H), 5.97 (dd, *J* = 5.2, 1.6 Hz, 1H), 5.87 (s, 1H), 4.15 (d, *J* = 7.2 Hz, 1H), 3.89 (s, 3H), 3.80 (s, 3H), 3.63 (dd, *J* = 7.1, 5.2 Hz, 1H), 2.32 (s, 3H), 2.27 (s, 3H), 1.62 (s, 3H).

^{13}C-NMR (100 MHz, $CDCl_3$): δ = 164.2, 158.9, 141.5, 138.0, 136.6, 133.8, 132.2, 129.5, 128.9, 127.8, 114.3, 114.3, 112.2, 88.7, 65.2, 55.4, 51.5, 51.2, 21.2, 11.6, 8.7.

FT-IR (thin film): ṽ [cm^{-1}] = 3418, 2952, 2922, 1657, 1514, 1448, 1281, 1251, 1140.

UV-Vis (CH_2Cl_2): λ [nm] = 230, 286.

HRMS (ESI-TOF) m/z: [M - H_2O + H]$^+$ Calcd. for $C_{25}H_{26}NO_3$ 388.1918; Found 388.1927.

HPLC IA column (90 % hexane, 10 % *iso*-propanol, 1 mL/min, 284 nm) τ_{minor} = 9.4 min, τ_{major} = 11.3 min.

Methyl (1*S*,2*S*,3*S*)-3-hydroxy-1-(3-methoxyphenyl)-2-(4-methoxyphenyl)-6,7-dimethyl-2,3-dihydro-1*H*-pyrrolizine-5-carboxylate (4s)

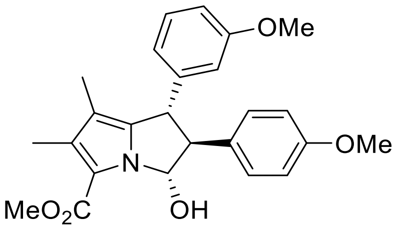

According to general procedure A: 58.0 mg (0.200 mmol) **1s**, 91.0 mg (0.606 mmol) **2i**, 9 h; Purified by silica-gel flash column chromatography (4-5 % EtOAc in hexanes);

Yield: 44.0 mg, 0.104 mmol, 52 % as a colorless oil, **e.r.:** 97.5 : 2.5, **[a]$^{24}_{D}$:** = +179° (c = 0.52, $CHCl_3$).

R$_f$ = 0.78 (Hex/EE 3:1).

^{1}H-NMR (400 MHz, $CDCl_3$): δ = 7.19 (t, *J* = 7.9 Hz, 1H), 7.12 (dt, *J* = 8.7, 1.8 Hz, 2H), 6.86 (dt, *J* = 8.7, 1.9 Hz, 2H), 6.78 (ddd, *J* = 8.3, 2.6, 1.0 Hz, 1H), 6.74 (dt, *J* = 7.7, 1.3 Hz, 1H), 6.72-6.71 (m, 1H), 5.98 (dd, *J* = 5.1, 1.9 Hz, 1H), 5.84 (s, 1H), 4.16 (d, *J* = 7.0 Hz, 1H), 3.89 (s, 3H), 3.80 (s, 3H), 3.74 (s, 3H), 3.66 (dd, *J* = 7.1, 5.1 Hz, 1H), 2.27 (s, 3H), 1.65 (s, 3H).

^{13}C-NMR (100 MHz, $CDCl_3$): δ = 164.2, 160.0, 159.0, 142.7, 141.2, 133.8, 132.3, 129.7, 128.9, 120.3, 114.4, 114.3, 113.5, 112.5, 112.3, 88.7, 64.9, 55.4, 55.3, 51.5, 51.5, 11.6, 8.7.

FT-IR (KBr): ṽ [cm^{-1}] = 3434, 2954, 2918, 1657, 1515, 1449, 1283, 1250, 1138.

UV-Vis (CH_2Cl_2): λ [nm] = 229, 285.

HRMS (ESI-TOF) m/z: [M - H_2O + H]$^+$ Calcd. for $C_{25}H_{26}NO_4$ 404.1867; Found 404.1862.

HPLC IE column (90 % hexane, 10 % *iso*-propanol, 1 mL/min, 284 nm) τ_{major} = 22.3 min, τ_{minor} = 33.1 min.

Methyl (1*S*,2*S*,3*S*)-1-(4-bromophenyl)-3-hydroxy-2-(4-methoxyphenyl)-6,7-dimethyl-2,3-dihydro-1*H*-pyrrolizine-5-carboxylate (4t)

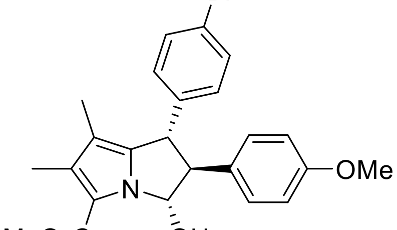

According to general procedure A: 68.2 mg (0.202 mmol) **1t**, 94.0 mg (0.626 mmol) **2i**, 6 h; Purified by silica-gel flash column chromatography (4 % EtOAc in hexanes);

Yield: 48.3 mg, 0.103 mmol, 51 % as a white foam, **e.r.:** 96.5 : 3.5, **[a]$^{24}_{D}$:** = +265° (c = 0.51, $CHCl_3$).

R$_f$ = 0.82 (Hex/EE 3:1).

m.p. = 144 – 146 °C.

^{1}H-NMR (400 MHz, $CDCl_3$): δ = 7.40 (dt, *J* = 8.4, 1.8 Hz, 2H), 7.09 (dt, *J* = 8.7, 1.9 Hz, 2H), 7.02 (dt, *J* = 8.4, 2.3 Hz, 2H), 6.86 (dt, *J* = 8.6, 3.0 Hz, 2H), 5.97 (d, *J* = 5.1 Hz, 1H), 5.84 (s, 1H), 4.13 (d, *J* = 7.1 Hz, 1H), 3.89 (s, 3H), 3.80 (s, 3H), 3.58 (dd, *J* = 7.1, 5.1 Hz, 1H), 2.27 (s, 3H), 1.62 (s, 3H).

^{13}C-NMR (100 MHz, $CDCl_3$): δ = 164.2, 159.1, 140.5, 140.2, 133.8, 131.9, 131.8, 129.6, 128.9, 121.0, 114.6, 114.4, 112.3, 88.6, 65.2, 55.4, 51.6, 51.0, 11.6, 8.7.

FT-IR (KBr): ṽ [cm^{-1}] = 3418, 2950, 2919, 1656, 1515, 1447, 1277, 1252, 1141.

UV-Vis (CH_2Cl_2): λ [nm] = 231, 286.

HRMS (ESI-TOF) m/z: $[M - H_2O + H]^+$ Calcd. for $C_{24}H_{23}BrNO_3$ 452.0867; Found 452.0853.

HPLC IE column (90 % hexane, 10 % *iso*-propanol, 1 mL/min, 284 nm) τ_{major} = 14.8 min, τ_{minor} = 20.6 min.

Methyl (1*S*,2*S*,3*S*)-1-(2,4-dimethoxyphenyl)-3-hydroxy-2-(4-methoxyphenyl)-6,7-dimethyl-2,3-dihydro-1*H*-pyrrolizine-5-carboxylate (4u)

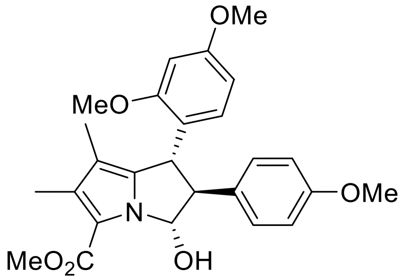

According to general procedure A: 63.8 mg (0.200 mmol) **1u**, 91.0 mg (0.600 mmol) **2i**, 5 h; Purified by silica-gel flash column chromatography (5-7 % EtOAc in hexanes);

Yield: 28.6 mg, 0.063 mmol, 32 % as a colorless oil, **e.r.:** 86 : 14, **$[a]^{24}_{D}$:** = +170° (c = 0.16, $CHCl_3$).

R_f = 0.65 (Hex/EE 3:1).

^{1}H-NMR (400 MHz, $CDCl_3$): δ = 7.10 (dt, *J* = 8.7, 3.0 Hz, 2H), 7.00 (dd, *J* = 9.1, 2.2 Hz, 1H), 6.83 (dt, *J* = 8.7, 1.8 Hz, 2H), 6.44-6.40 (m, 2H), 5.93 (d, *J* = 4.1 Hz, 1H), 5.47 (s, 1H), 4.57 (d, *J* = 5.8 Hz, 1H), 3.87 (s, 3H), 3.79 (s, 3H), 3.78 (s, 3H), 3.63 (dd, *J* = 5.8, 4.0 Hz, 1H), 3.60 (s, 3H), 2.28 (s, 3H), 1.66 (s, 3H).

^{13}C-NMR (100 MHz, $CDCl_3$): δ = 164.0, 159.8, 158.6, 158.0, 142.1, 133.9, 133.7, 129.0, 128.5, 122.3, 114.2, 114.0, 112.1, 104.7, 98.7, 88.8, 63.7, 55.4, 55.4, 55.4, 51.4, 43.8, 11.6, 8.6.

FT-IR (thin film): ṽ [cm^{-1}] = 3439, 2953, 2932, 1659, 1513, 1450, 1283, 1251, 1139.

UV-Vis (CH_2Cl_2): λ [nm] = 232, 286.

HRMS (ESI-TOF) m/z: $[M - H_2O + H]^+$ Calcd. for $C_{26}H_{28}NO_5$ 434.1973; Found 434.1962.

HPLC IA column (90 % hexane, 10 % *iso*-propanol, 1 mL/min, 284 nm) τ_{minor} = 14.4 min, τ_{major} = 19.2 min.

Methyl (1*S*,2*S*,3*S*)-3-hydroxy-2-(4-methoxyphenyl)-1-phenyl-2,3,6,7,8,9-hexahydro-1*H*-pyrrolo[2,1-*a*]isoindole-5-carboxylate (4v)

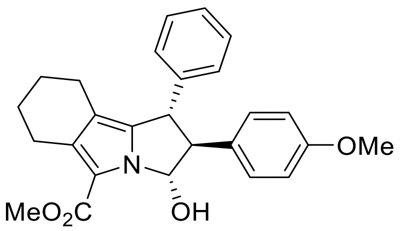

According to general procedure A: 58.0 mg (0.203 mmol) **1l**, 94.0 mg (0.626 mmol) **2i**, 6 h; Purified by silica-gel flash column chromatography (4 % EtOAc in hexanes);

Yield: 35.4 mg, 0.085 mmol, 42 % as a colorless oil, **e.r.:** 97 : 3, **$[a]^{25}_{D}$:** = +160° (c = 1.00, $CHCl_3$).

R_f = 0.66 (Hex/EE 3:1).

^{1}H-NMR (400 MHz, $CDCl_3$): δ = 7.31 – 7.27 (m, 2H), 7.26 – 7.21 (m, 1H), 7.18 – 7.09 (m, 4H), 6.86 (dt, *J* = 8.7, 2.0 Hz, 2H), 6.01 (dd, *J* = 5.5, 1.8 Hz, 1H), 5.98 (s, 1H), 4.19 (d, *J* = 7.7 Hz, 1H), 3.87 (s, 3H), 3.80 (s, 3H), 3.67 (dd, *J* = 7.7, 5.5 Hz, 1H), 2.83 (dt, *J* = 6.4, 3.2 Hz, 2H), 2.12 (dt, *J* = 15.8, 6.0 Hz, 1H), 2.00 – 1.89 (m, 1H), 1.71 (dtd, *J* = 10.5, 6.5, 3.1 Hz, 2H), 1.65 – 1.54 (m, 2H).

^{13}C-NMR (100 MHz, $CDCl_3$): δ = 163.9, 159.0, 140.5, 139.9, 135.6, 131.7, 129.0, 128.8, 127.9, 127.1, 114.3, 114.1, 113.1, 88.6, 65.2, 55.4, 51.5, 51.5, 24.4, 23.2, 23.1, 21.1.

FT-IR (KBr): ṽ [cm^{-1}] = 3434, 2934, 2855, 1648, 1467, 1453, 1278, 1254, 1134.

UV-Vis (CH_2Cl_2): λ [nm] = 230, 286.

HRMS (ESI-TOF) m/z: [M - H_2O + H]$^+$ Calcd. for $C_{26}H_{26}NO_3$ 400.1918; Found 400.1914.

HPLC IA column (90 % hexane, 10 % *iso*-propanol, 1 mL/min, 284 nm) τ_{major} = 8.2 min, τ_{minor} = 9.2 min.

Methyl (1*S*,2*S*,3*S*)-6-ethyl-3-hydroxy-2-(4-methoxyphenyl)-7-methyl-1-phenyl-2,3-dihydro-1*H*-pyrrolizine-5-carboxylate (4w)

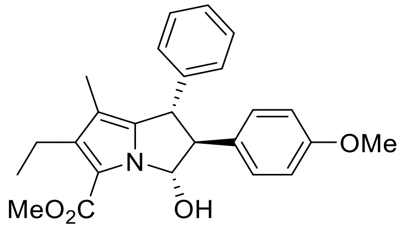

According to general procedure A: 55.7 mg (0.204 mmol) **1k**, 94.0 mg (0.626 mmol) **2i**, 6 h; Purified by silica-gel flash column chromatography (4 % EtOAc in hexanes);

Yield: 47.9 mg, 0.118 mmol, 58 % as a colorless oil, **e.r.:** 95 : 5, **$[a]^{25}_{D}$:** = +180° (c = 1.00, $CHCl_3$).

R_f = 0.71 (Hex/EE 3:1).

^{1}H-NMR (400 MHz, $CDCl_3$): δ = 7.28 (ddt, *J* = 8.0, 6.2, 1.3 Hz, 2H), 7.26-7.20 (m, 1H), 7.16 (d, *J* = 1.7 Hz, 1H), 7.14 (d, *J* = 1.7 Hz, 2H), 7.12 (d, *J* = 2.1 Hz, 1H), 6.86 (dt, *J* = 8.7, 2.0 Hz, 2H),

5.99 (d, *J* = 5.3 Hz, 1H), 5.92 (s, 1H), 4.19 (d, *J* = 7.4 Hz, 1H), 3.89 (s, 3H), 3.80 (s, 3H), 3.65 (dd, *J* = 7.4, 5.3 Hz, 1H), 2.73 (qd, *J* = 7.5, 5.3 Hz, 2H), 1.62 (s, 3H), 1.14 (t, *J* = 7.5 Hz, 3H).

^{13}C-NMR (100 MHz, $CDCl_3$): δ = 164.1, 159.0, 141.4, 141.0, 140.5, 132.0, 129.0, 128.8, 127.9, 127.1, 114.3, 113.6, 111.5, 88.7, 65.2, 55.4, 51.5, 19.3, 15.3, 8.5.

FT-IR (KBr): ṽ [cm^{-1}] = 3419, 2971, 2927, 1651, 1517, 1459, 1456, 1292, 1250, 1145.

UV-Vis (CH_2Cl_2): λ [nm] = 230, 286.

HRMS (ESI-TOF) m/z: [M - H_2O + H]$^+$ Calcd. for $C_{25}H_{26}NO_3$ 388.1918; Found 388.1915.

HPLC IE column (90 % hexane, 10 % *iso*-propanol, 1 mL/min, 284 nm) τ_{major} = 11.7 min, τ_{minor} = 20.2 min.

Methyl (1*S*,2*S*,3*S*)-3-hydroxy-2-(4-methoxyphenyl)-7-methyl-1,6-diphenyl-2,3-dihydro-1*H*-pyrrolizine-5-carboxylate (4x)

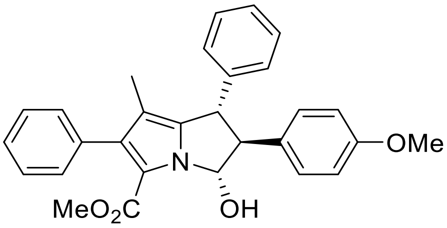

According to general procedure A: 64.9 mg (0.202 mmol) **1x**, 91.0 mg (0.606 mmol) **2i**, 9 h; Purified by silica-gel flash column chromatography (4-5 % EtOAc in hexanes);

Yield: 24.1 mg, 0.053 mmol, 26 % as a colorless oil, **e.r.:** 88 : 12, **$[a]^{24}_{D}$:** = +303° (c = 0.16, $CHCl_3$).

R_f = 0.77 (Hex/EE 3:1).

^{1}H-NMR (400 MHz, $CDCl_3$): δ = 7.38 (ddd, *J* = 7.7, 6.6, 1.1 Hz, 2H), 7.31 (tdd, *J* = 5.7, 4.3, 2.1 Hz, 5H), 7.27-7.24 (m, 1H), 7.20 (dt, *J* = 6.8, 1.5 Hz, 2H), 7.17 (dt, *J* = 8.7, 2.1 Hz, 2H), 6.89 (dt, *J* = 8.7, 3.0 Hz, 2H), 6.09 (dd, *J* = 5.3, 2.1 Hz, 1H), 5.97 (d, *J* = 2.1 Hz, 1H), 4.27 (d, *J* = 7.4 Hz, 1H), 3.81 (s, 3H), 3.73 (dd, *J* = 7.4, 5.3 Hz, 1H), 3.61 (s, 3H), 1.54 (s, 3H).

^{13}C-NMR (100 MHz, $CDCl_3$): δ = 163.8, 159.1, 141.2, 140.7, 138.0, 135.5, 131.9, 130.3, 129.0, 128.8, 128.0, 127.6, 127.2, 126.9, 114.4, 113.7, 112.4, 89.1, 65.2, 55.4, 51.6, 51.4, 9.3.

FT-IR (thin film): ṽ [cm^{-1}] = 3440, 3026, 2952, 2923, 1662, 1515, 1463, 1251, 1179.

UV-Vis (CH_2Cl_2): λ [nm] = 230, 286.

HRMS (ESI-TOF) m/z: [M - H_2O + H]$^+$ Calcd. for $C_{29}H_{26}NO_3$ 436.1918; Found 436.1915.

HPLC IA column (90 % hexane, 10 % *iso*-propanol, 1 mL/min, 284 nm) τ_{minor} = 7.5 min, τ_{major} = 10.5 min.

Methyl (1*S*,2*S*,3*S*)-1,2-bis(4-bromophenyl)-3-hydroxy-6,7-dimethyl-2,3-dihydro-1*H*-pyrrolizine-5-carboxylate (4y)

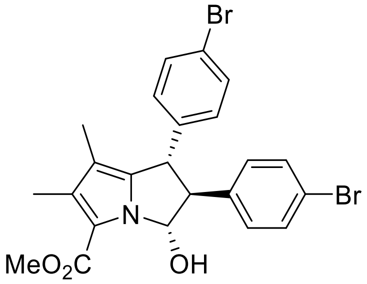

According to general procedure A: 67.2 mg (0.199 mmol) **1t**, 120.1 mg (0.603 mmol) **2f**, 7 h; Purified by silica-gel flash column chromatography (4 % EtOAc in hexanes);

Yield: 67.3 mg, 0.130 mmol, 65 % as an off-white solid, **e.r.:** 90.5 : 9.5, **$[a]^{25}_{D}$:** = +158° (c = 0.76, $CHCl_3$).

R_f = 0.81 (Hex/EE 3:1).

m.p. = 126 – 128 °C.

^{1}H-NMR (400 MHz, $CDCl_3$): δ = 7.45 (dd, *J* = 8.4, 1.9 Hz, 2H), 7.41 (dd, *J* = 8.4, 1.8 Hz, 2H), 7.05 (dt, *J* = 8.4, 2.4 Hz, 2H), 7.02 (dt, *J* = 8.5, 1.8 Hz, 2H), 5.98 (dd, *J* = 5.0, 1.7 Hz, 1H), 5.86 (d, *J* = 2.1 Hz, 1H), 4.12 (d, *J* = 7.0 Hz, 1H), 3.89 (s, 3H), 3.60 (dd, *J* = 7.0, 5.0 Hz, 1H), 2.27 (s, 3H), 1.61 (s, 3H).

^{13}C-NMR (100 MHz, $CDCl_3$): δ = 164.2, 140.2, 139.8, 138.8, 134.0, 132.2, 132.0, 129.6, 129.5, 121.6, 121.3, 114.7, 112.4, 88.3, 65.3, 51.6, 50.9, 11.6, 8.7.

FT-IR (thin film): ṽ [cm^{-1}] = 3429, 2952, 2924, 1660, 1489, 1448, 1281, 1141.

UV-Vis (CH_2Cl_2): λ [nm] = 232, 286.

HRMS (ESI-TOF) m/z: [M - H_2O + H]$^+$ Calcd. for $C_{23}H_{20}Br_2NO_2$ 499.9866; Found 499.9817.

HPLC IE column (90 % hexane, 10 % *iso*-propanol, 1 mL/min, 284 nm) τ_{major} = 8.8 min, τ_{minor} = 12.4 min.

Methyl (1*S*,2*S*,3*S*)-3-hydroxy-6,7-dimethyl-1-(naphthalen-2-yl)-2-phenyl-2,3-dihydro-1*H*-pyrrolizine-5-carboxylate (4z)

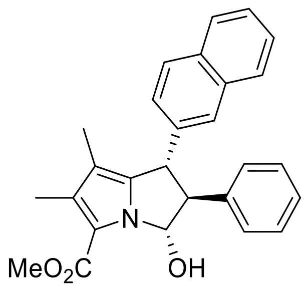

According to general procedure A: 61.9 mg (0.200 mmol) **1m**, 72.1 mg (0.600 mmol) **2a**, 4.5 h; Purified by silica-gel flash column chromatography (5 % EtOAc in hexanes);

Yield: 52.4 mg, 0.127 mmol, 64 % as a colorless oil, **e.r.:** 93 : 7, **$[a]^{25}_{D}$:** = +268° (c = 1.00, $CHCl_3$).

R_f = 0.73 (Hex/EE 3:1).

^{1}H-NMR (400 MHz, $CDCl_3$): δ = 7.85-7.78 (m, 2H), 7.75 (dd, *J* = 6.1, 3.4 Hz, 1H), 7.61 (d, *J* = 1.8 Hz, 1H), 7.46 (dt, *J* = 6.2, 3.4 Hz, 2H), 7.33 (dtt, *J* = 8.6, 6.9, 3.6 Hz, 4H), 7.23-7.20 (m, 2H), 6.11 (dd, *J* = 5.0, 2.0 Hz, 1H), 5.89 (s, 1H), 4.41 (d, *J* = 6.9 Hz, 1H), 3.92 (s, 3H), 3.83 (dd, *J* = 6.9, 4.9 Hz, 1H), 2.30 (s, 3H), 1.61 (s, 3H).

^{13}C-NMR (100 MHz, $CDCl_3$): δ = 164.2, 141.3, 140.3, 138.5, 134.0, 133.6, 132.8, 129.0, 128.8, 127.9, 127.9, 127.8, 127.5, 126.6, 126.2, 126.0, 125.9, 114.5, 112.4, 88.7, 65.7, 51.8, 51.5, 11.7, 8.7.

FT-IR (KBr): ṽ [cm^{-1}] = 3442, 2952, 2919, 1654, 1471, 1448, 1281, 1138.

UV-Vis (CH_2Cl_2): λ [nm] = 231, 288.

HRMS (ESI-TOF) m/z: [M - H_2O + H]$^+$ Calcd. for $C_{27}H_{24}NO_2$ 394.1813; Found 394.1805.

HPLC IA column (98 % hexane, 2 % *iso*-propanol, 1 mL/min, 284 nm) τ_{minor} = 16.1 min, τ_{minor} = 18.5 min.

Methyl (1*S*,2*S*,3*S*)-3-hydroxy-1-(4-methoxyphenyl)-6,7-dimethyl-2-phenyl-2,3-dihydro-1*H*-pyrrolizine-5-carboxylate (4aa)

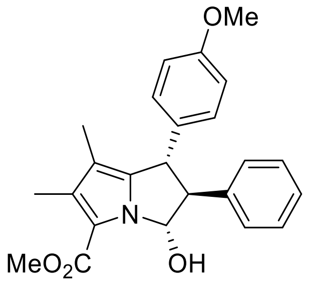

According to general procedure A: 58.0 mg (0.200 mmol) **1n**, 72.1 mg (0.600 mmol) **2a**, 1.5 h; Purified by silica-gel flash column chromatography (6-8 % EtOAc in hexanes);

Yield: 51.5 mg, 0.132 mmol, 66 % as a colorless oil, **e.r.:** 85 : 15, **$[a]^{25}_{D}$:** = +202° (c = 1.00, $CHCl_3$).

R_f = 0.71 (Hex/EE 3:1).

^{1}H-NMR (400 MHz, $CDCl_3$): δ = 7.35-7.27 (m, 3H), 7.20-7.16 (m, 2H), 7.10-7.05 (m, 2H), 6.84-6.79 (m, 2H), 6.02 (d, *J* = 5.0 Hz, 1H), 5.83 (s, 1H), 4.17 (d, *J* = 6.9 Hz, 1H), 3.88 (s, 3H), 3.78 (s, 3H), 3.67 (dd, *J* = 7.0, 5.0 Hz, 1H), 2.27 (s, 3H), 1.62 (s, 3H).

^{13}C-NMR (100 MHz, $CDCl_3$): δ = 164.2, 158.8, 141.7, 140.4, 133.9, 133.2, 129.0, 127.8, 127.4, 114.3, 114.2, 112.2, 88.6, 65.9, 55.4, 51.5, 50.9, 11.6, 8.6

FT-IR (KBr): ṽ [cm^{-1}] = 3444, 2952, 2921, 1661, 1511, 1448, 1278, 1142.

UV-Vis (CH_2Cl_2): λ [nm] = 230, 288.

HRMS (ESI-TOF) m/z: [M - H_2O + H]$^+$ Calcd. for $C_{24}H_{24}NO_3$ 374.1762; Found 374.1763.

HPLC IA column (90 % hexane, 10 % *iso*-propanol, 1 mL/min, 286 nm) τ_{minor} = 9.3 min, τ_{major} = 10.2 min.

1.5 Large scale synthesis of **4m**

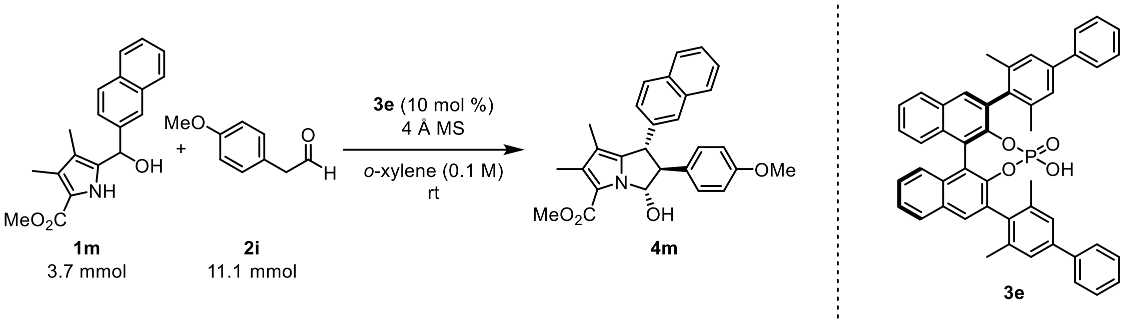

In a round-bottom flask, 1.14 g (1*H*-pyrrol-2-yl)(aryl)methanol **1m** (3.70 mmol, 1.0 equiv) and 1.11 g pulverized 4 Å MS were suspended in 37 mL *o*-xylol. 1.60 ml freshly distilled aryl acetaldehyde **2i** (1.67 g, 11.10 mmol, 3.0 equiv) was added, followed by catalyst **3e** (0.37 mmol, 10 mol%) and stirred for 6.5 h at rt. Reaction mixture was filtered over cellite and solvents were removed under reduced pressure to obtain a yellowish-brown oil. This residue was purified by silica-gel flash column chromatography (3-5 % EtOAc in hexanes) to obtain **4m** as white foam (1.07 g, 2.43 mmol, 66 %) with 98 : 2 *e.r.*.

1.6 Procedure for functionalization of hemiacetal **4a**

Methyl (1*S*,2*S*,3*R*)-6,7-dimethyl-1,2-diphenyl-3-(1H-pyrrol-2-yl)-2,3-dihydro-1*H*-pyrrolizine-5-carboxylate (5)

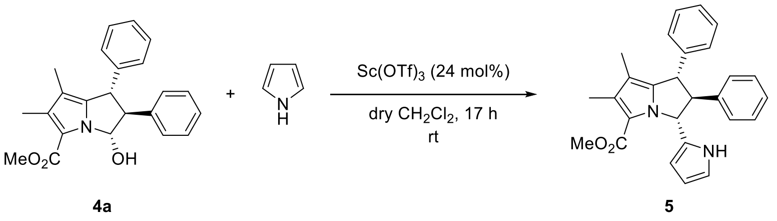

Flame dried flask was charged with 24.1 mg pyrrolizine **4a** (67 µmol, 1.0 equiv) in 2 mL dry CH_2Cl_2. 8.0 mg $Sc(OTf)_3$ (16 µmol, 24 mol%) was added, followed by 13.8 µl freshly distilled pyrrole (13.4 mg, 200 µmol, 3.0 equiv). Reaction mixture was stirred for 17 h at rt and filtered through a cotton plug. Solvents were removed under reduced pressure and the residue was purified by silica-gel flash column chromatography (7 % EtOAc in hexanes) to obtain **5**.

Yield: 23.9 mg, 58 µmol, 87 % as a purple solid, **e.r.:** 95 : 5, **[a]$^{25}_{D}$:** = +22° (c = 1.00, $CHCl_3$).

R$_f$ = 0.31 (Hex/EE 9:1).

m.p. = 164 – 166 °C.

^{1}H-NMR (300 MHz, CDCl$_3$): δ = 9.38 (s, 1H), 7.37 – 7.24 (m, 6H), 7.18 – 7.11 (m, 4H), 6.60 (dt, *J* = 4.0, 1.9 Hz, 1H), 5.91 (q, *J* = 2.9 Hz, 1H), 5.86 (d, *J* = 2.3 Hz, 1H), 5.41 (p, *J* = 1.7 Hz, 1H), 4.46 (d, *J* = 2.6 Hz, 1H), 4.12 (t, *J* = 2.5 Hz, 1H), 3.82 (s, 3H), 2.32 (s, 3H), 1.80 (s, 3H).

^{13}C-NMR (75 MHz, CDCl$_3$): δ = 163.5, 145.4, 141.8, 140.8, 133.5, 132.0, 129.2, 128.8, 127.9, 127.3, 127.1, 126.5, 117.6, 114.9, 113.0, 107.7, 106.8, 64.5, 64.0, 51.7, 51.0, 12.1, 9.3.

FT-IR (KBr): ṽ [cm^{-1}] = 3399, 2949, 2921, 1675, 1493, 1442, 1279, 1125.

UV-Vis (CH$_2$Cl$_2$): λ [nm] = 228, 285.

HRMS (ESI-TOF) m/z: [M + H]$^+$ Calcd. for $C_{27}H_{27}N_2O_2$ 411.2073; Found 411.2075.

HPLC IA column (95 % hexane, 5 % *iso*-propanol, 1 mL/min, 284 nm) τ_{major} = 5.1 min, τ_{minor} = 12.9 min.

Methyl (1*S*,2*S*)-6,7-dimethyl-3-oxo-1,2-diphenyl-2,3-dihydro-1H-pyrrolizine-5-carboxylate (6)

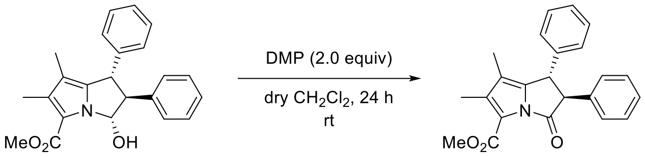

4a **6**

Flame-dried flask was charged with 36.7 mg pyrrolizine **4a** (102 µmol, 1.0 equiv) in 4 mL dry CH_2Cl_2. 85 mg DMP (200 µmol, 2.0 equiv) was added and stirred for 24 h at rt. Reaction was terminated with 6 mL water and extracted with 3x 15 mL CH_2Cl_2. Combined organic extracts were dried over Na_2SO_4, filtered and solvent was removed under reduced pressure. Residue was purified by silica-gel flash column chromatography (13 % Et_2O in hexanes) to obtain **6**.

Yield: 27.5 mg, 77 µmol, 73 % as an orange solid, **e.r.:** 95 : 5, **[a]$^{25}_{D}$:** = +4° (c = 1.00, $CHCl_3$).

R_f = 0.63 (Hex/Et_2O 1:1).

m.p. = 117 – 119 °C.

^{1}H-NMR (400 MHz, $CDCl_3$): δ = 7.38 – 7.27 (m, 6H), 7.16 (dt, *J* = 6.4, 1.7 Hz, 2H), 7.11 (dt, *J* = 6.4, 1.7 Hz, 2H), 4.37 (dd, *J* = 5.0, 1.3 Hz, 1H), 4.11 (d, *J* = 5.0 Hz, 1H), 3.89 (s, 3H), 2.32 (s, 3H), 1.65 (s, 3H).

^{13}C-NMR (75 MHz, $CDCl_3$): δ = 169.4, 161.2, 141.4, 141.2, 140.0, 137.3, 129.2, 129.2, 128.2, 128.0, 127.7, 127.4, 116.6, 116.5, 63.5, 51.6, 48.0, 11.4, 8.7.

FT-IR (thin film): ṽ [cm^{-1}] = 3443, 3028, 2921, 1767, 1714, 1612, 1439, 1282, 1143.

UV-Vis (CH_2Cl_2): λ [nm] = 228, 252, 291.

HRMS (ESI-TOF) m/z: [M + H]$^+$ Calcd. for $C_{23}H_{22}NO_3$ 360.1600; Found 360.1601.

HPLC IB column (95 % hexane, 5 % *iso*-propanol, 1 mL/min, 288 nm) τ_{minor} = 14.4 min, τ_{major} = 17.1 min.

1.7 Isolation of Dimer

Dimethyl 5,5'-(oxybis(phenylmethylene))bis(3,4-dimethyl-1H-pyrrole-2-carboxylate) 7

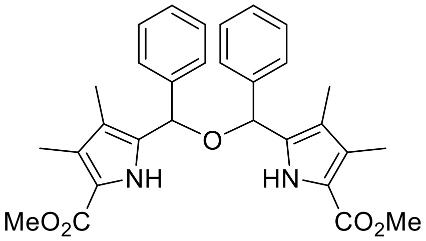

Compound **7** was found in all reactions during the optimization for **4a**. Similar compounds were found in the crude ^{1}H-NMR of all cycloaddition products **4**.

R_f = 0.12 (Hex/Et_2O 7:3).

^{1}H-NMR (400 MHz, $CDCl_3$): δ = 8.73 (s, 2H), 7.47 – 7.29 (m, 10H), 5.47 (s, 2H), 3.80 (s, 6H), 2.25 (s, 6H), 1.78 (s, 6H).

^{13}C-NMR (75 MHz, $CDCl_3$): δ = 162.1, 139.6, 131.8, 128.9, 128.3, 127.5, 127.1, 118.8, 118.4, 73.1, 51.1, 10.5, 8.8.

FT-IR (thin film): ṽ [cm^{-1}] = 3303, 2924, 1670, 1450, 1258, 1108.

UV-Vis (CH_2Cl_2): λ [nm] = 229, 281.

HRMS (ESI-TOF) m/z: $[M + Na]^+$ Calcd. for $C_{30}H_{33}N_2O_5Na$ 523.2209; Found 523.2188.

2. NMR Spectra of Products

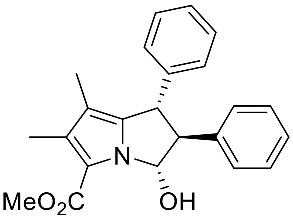

4a

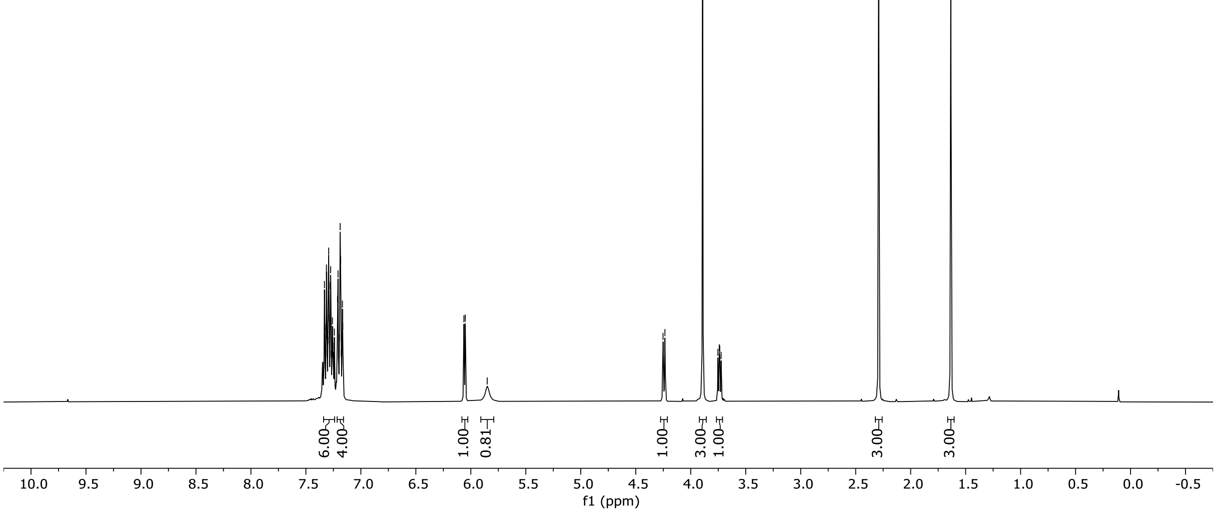

^{1}H-NMR (400 MHz, $CDCl_3$)

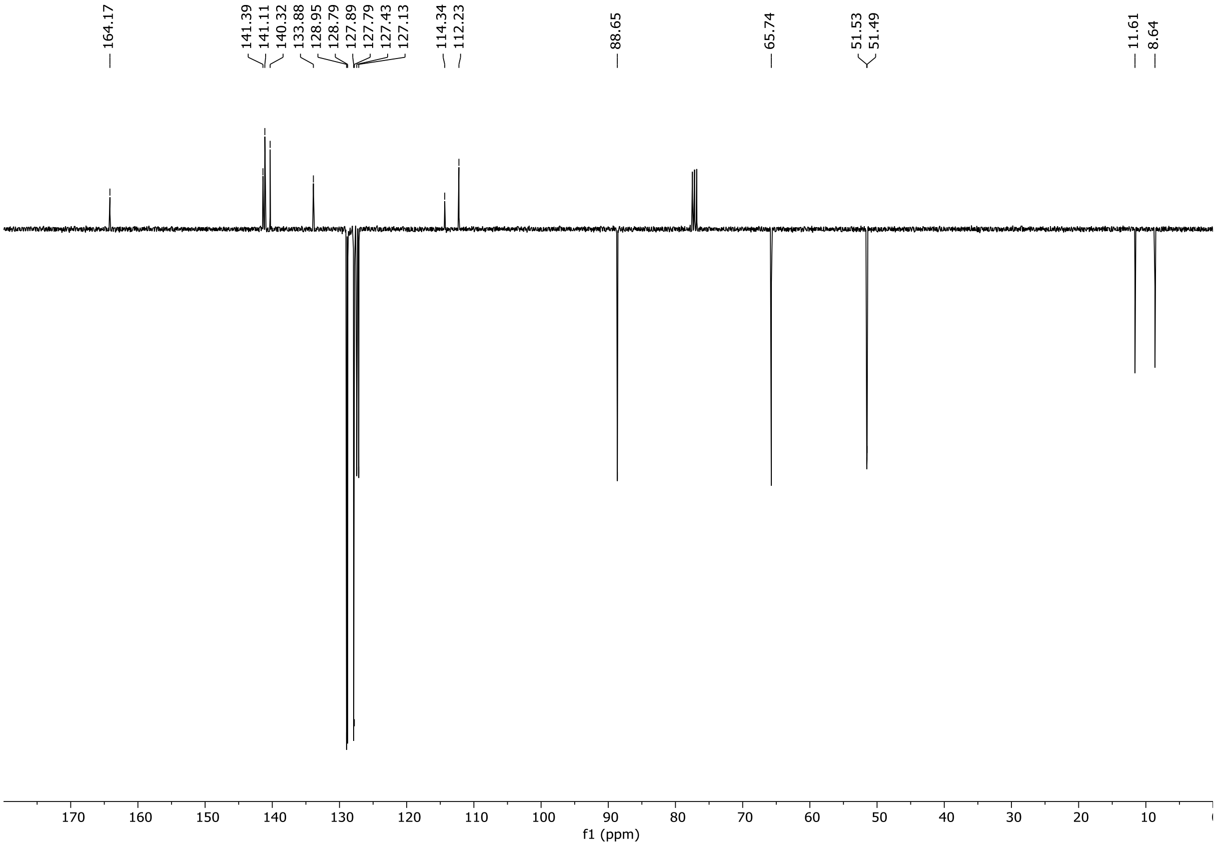

^{13}C-NMR (100 MHz, $CDCl_3$)

4b

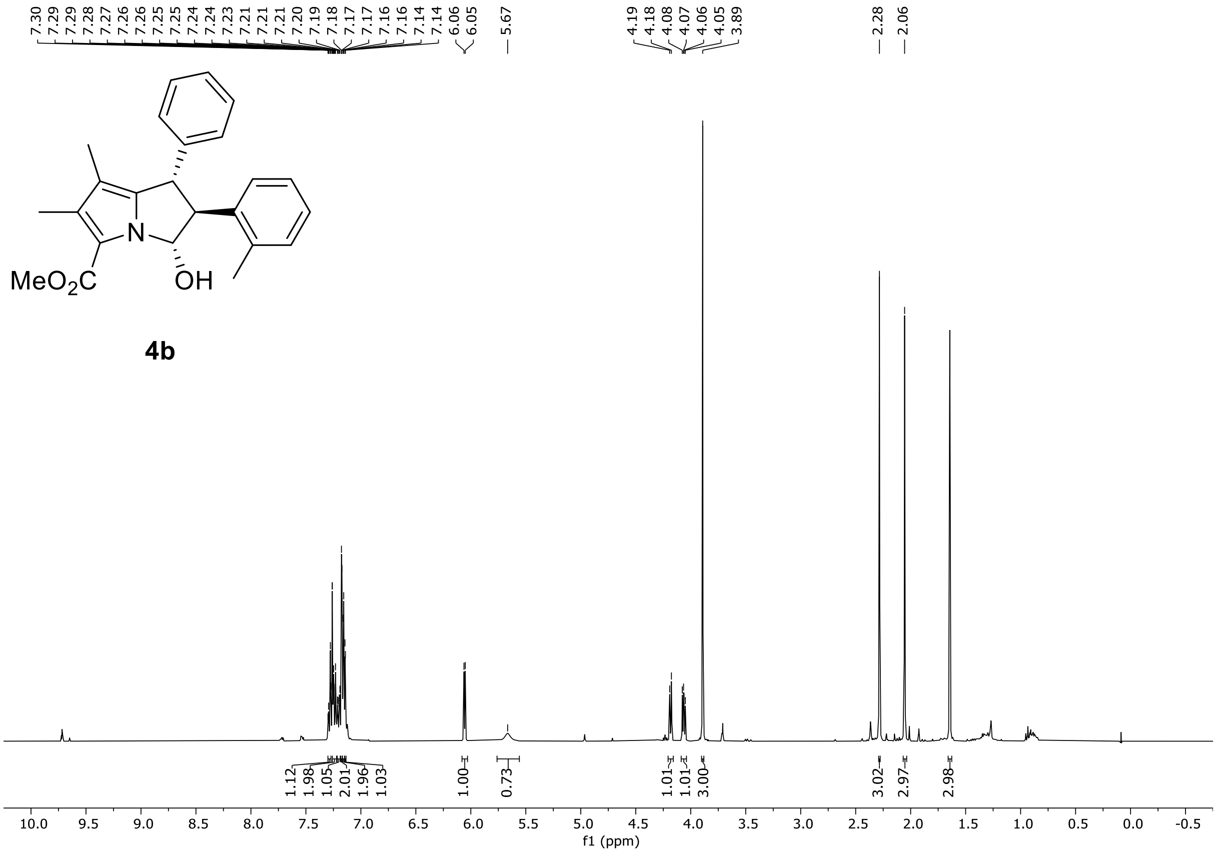

^{1}H-NMR (400 MHz, $CDCl_3$)

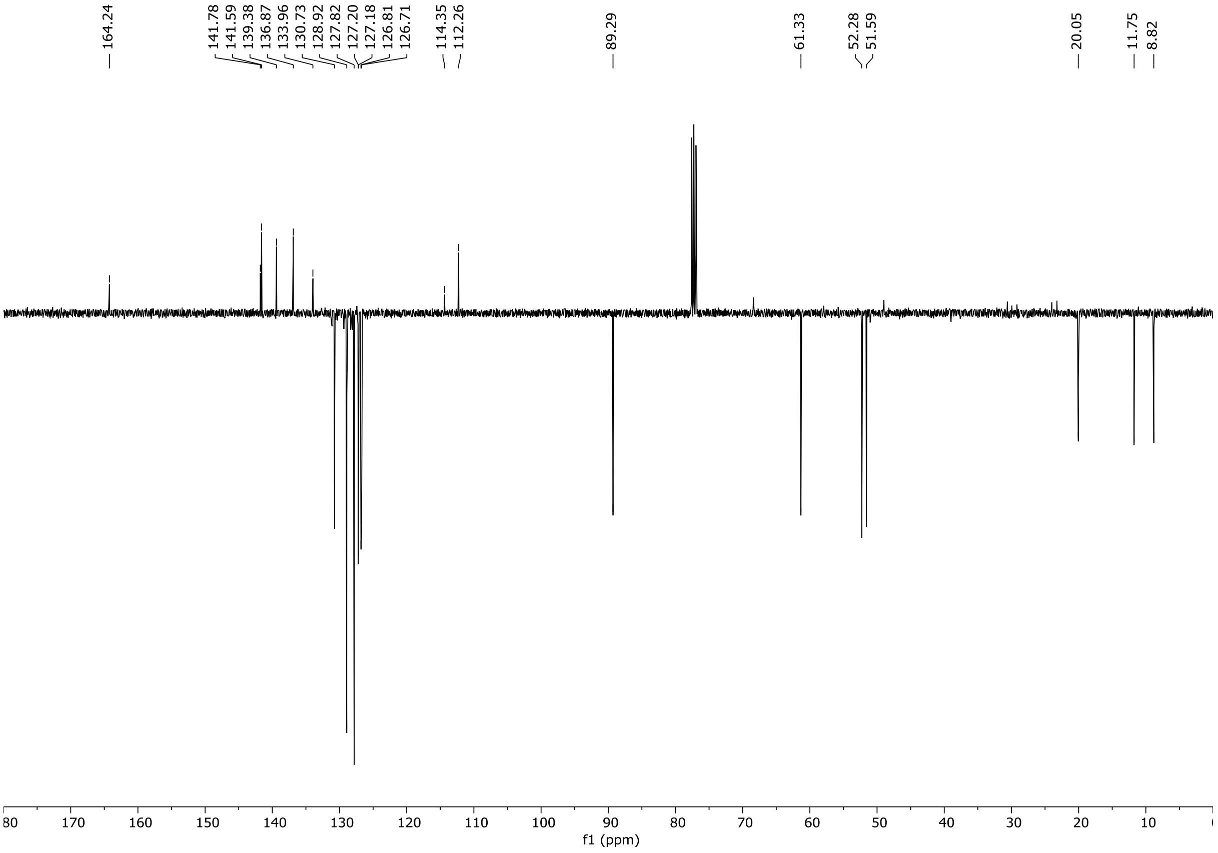

^{13}C-NMR (100 MHz, $CDCl_3$)

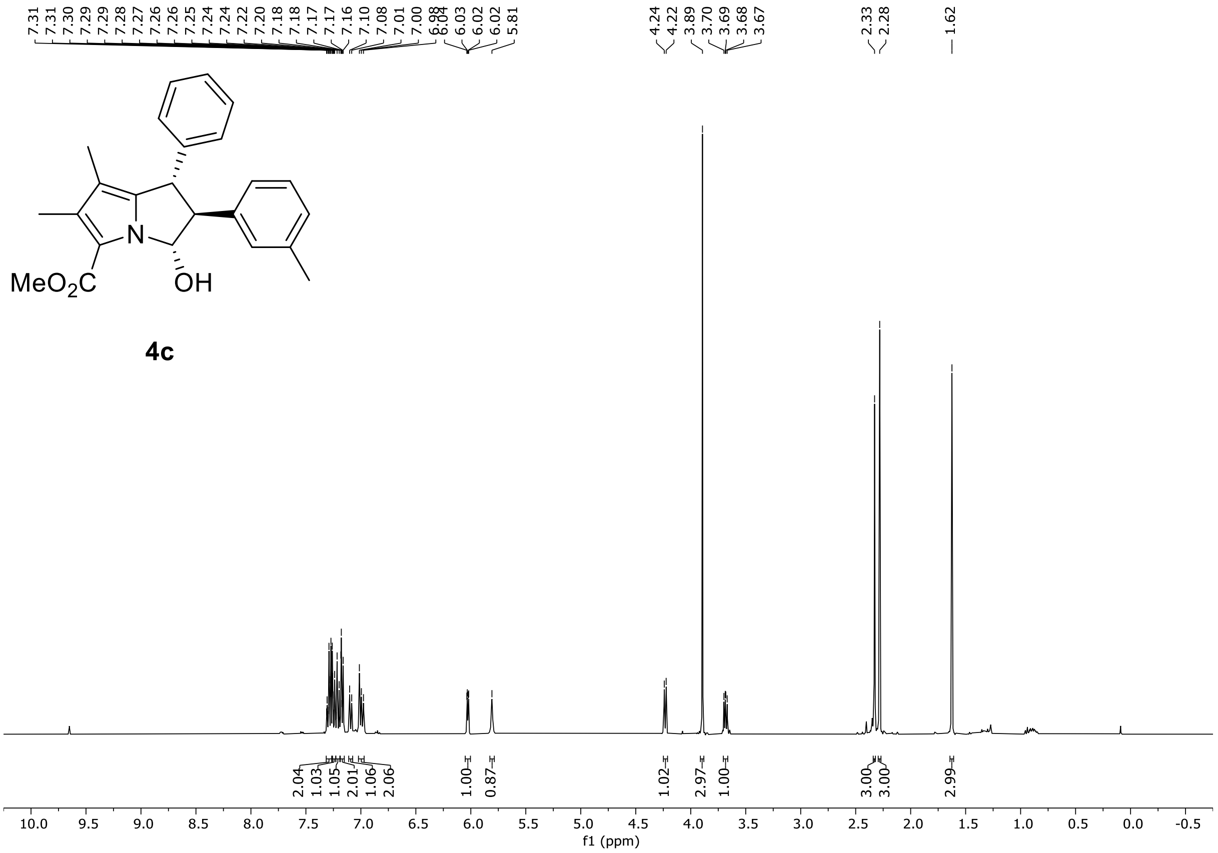

^{1}H-NMR (400 MHz, $CDCl_3$)

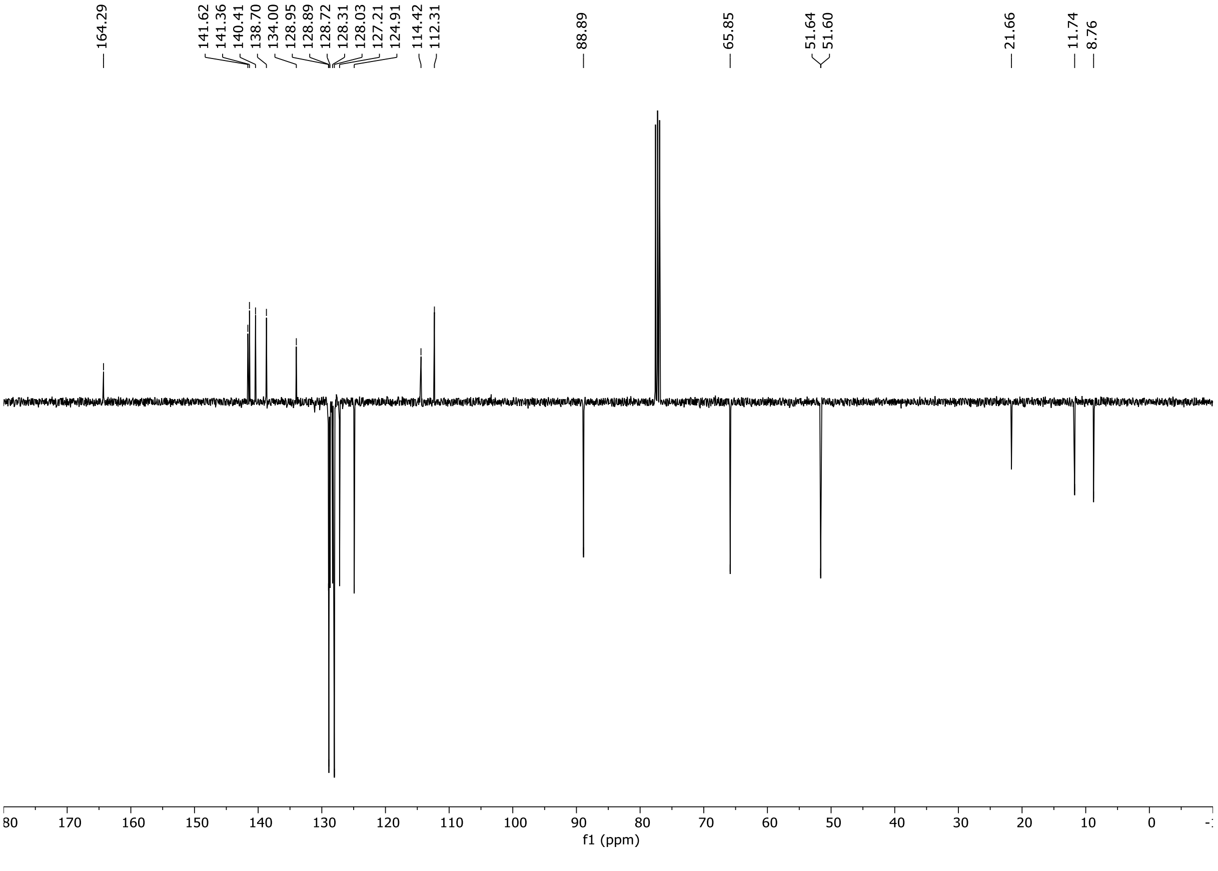

^{13}C-NMR (100 MHz, $CDCl_3$)

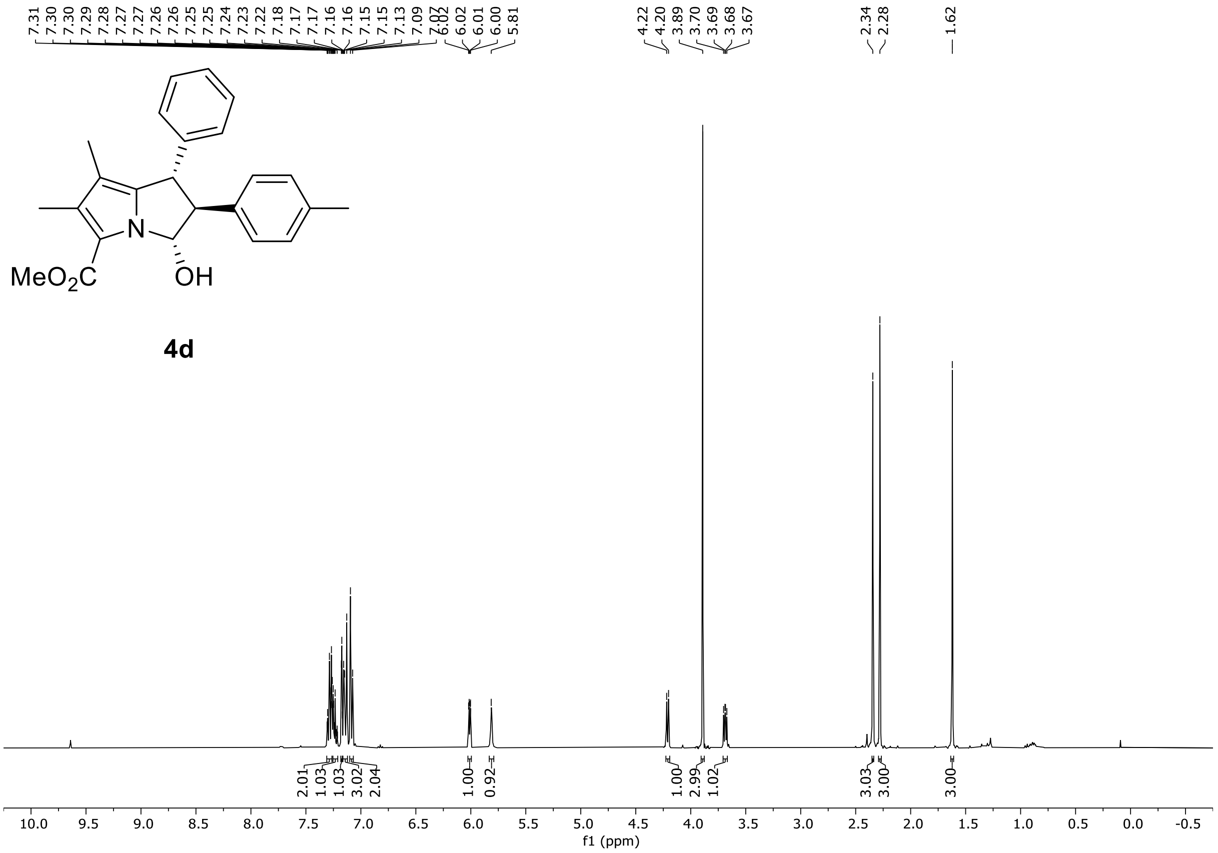

^{1}H-NMR (400 MHz, $CDCl_3$)

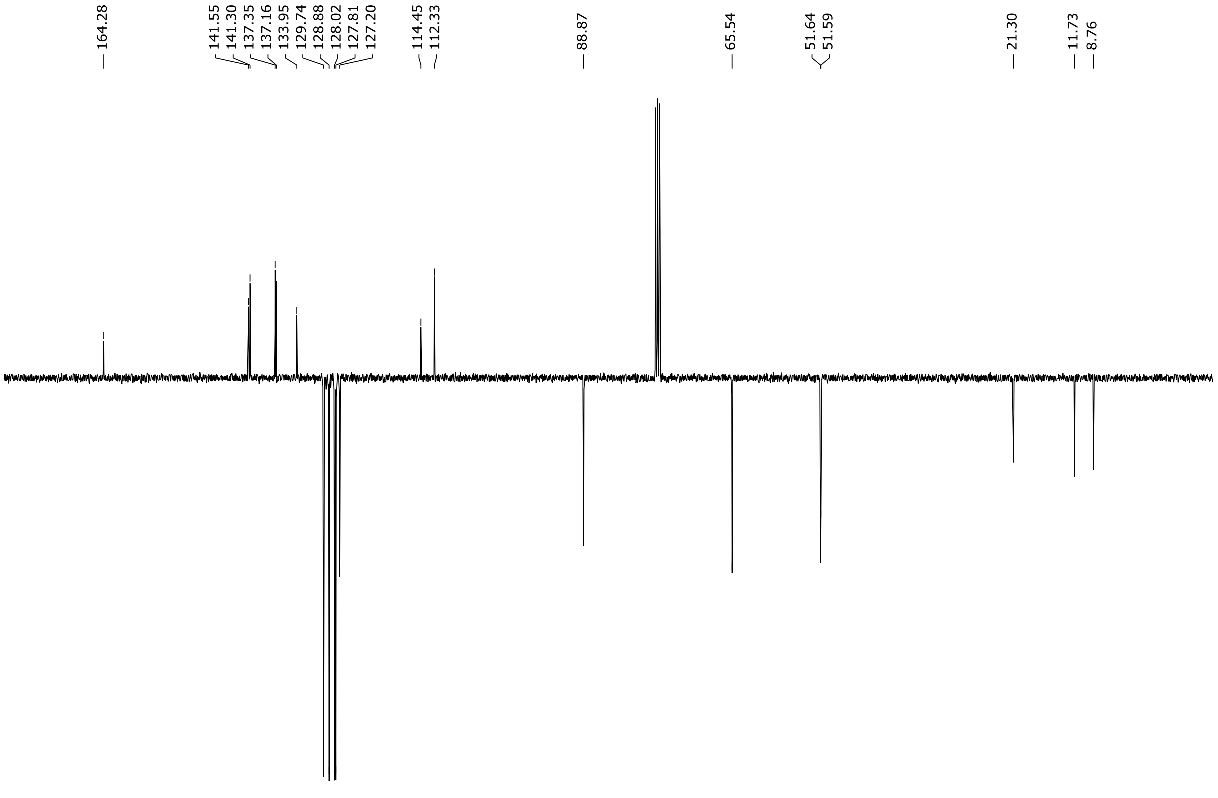

^{13}C-NMR (100 MHz, $CDCl_3$)

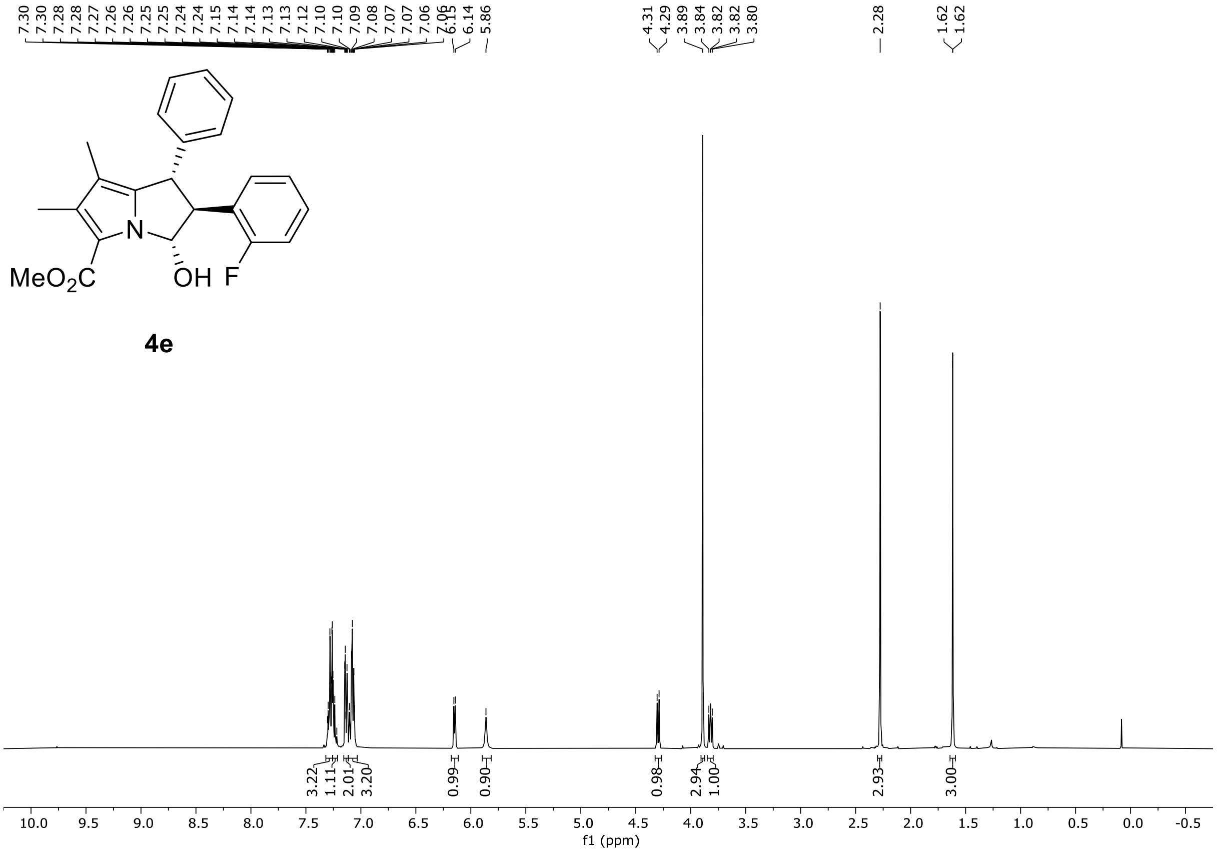

^{1}H-NMR (400 MHz, $CDCl_3$)

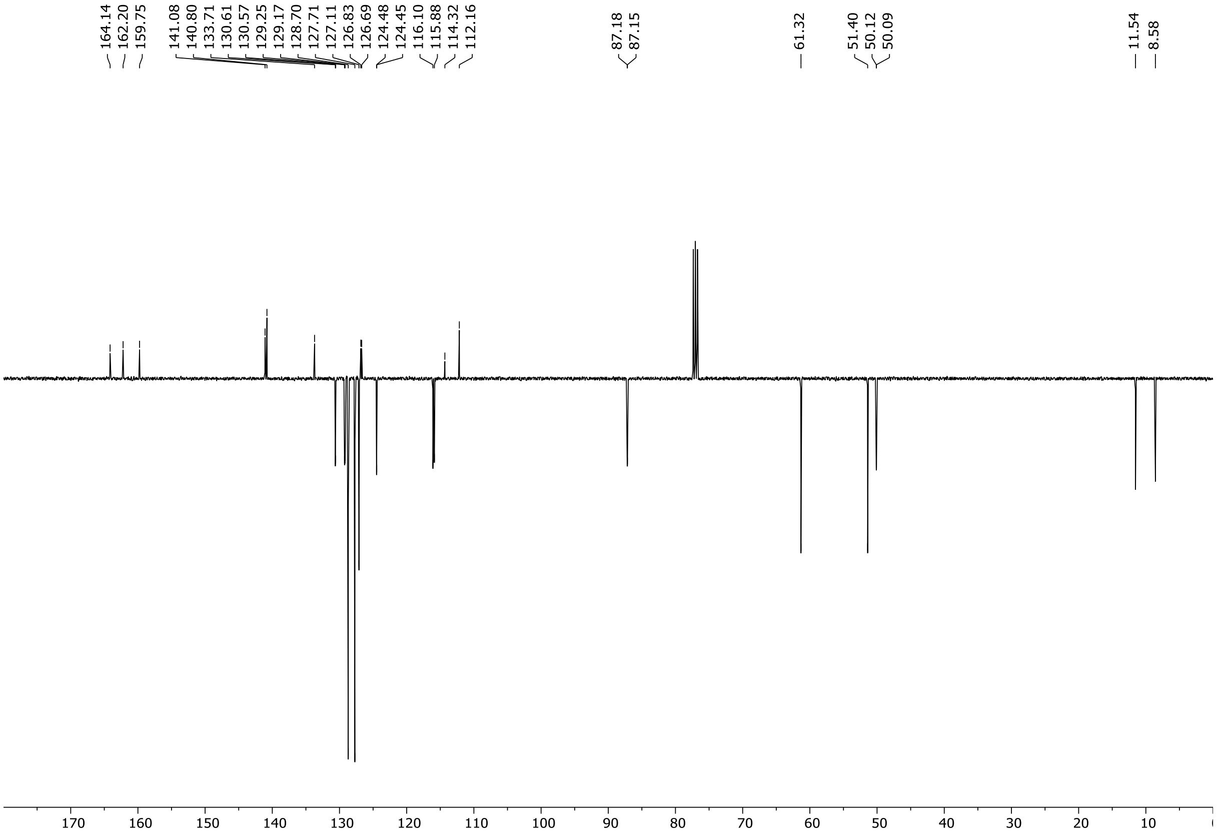

^{13}C-NMR (100 MHz, $CDCl_3$)

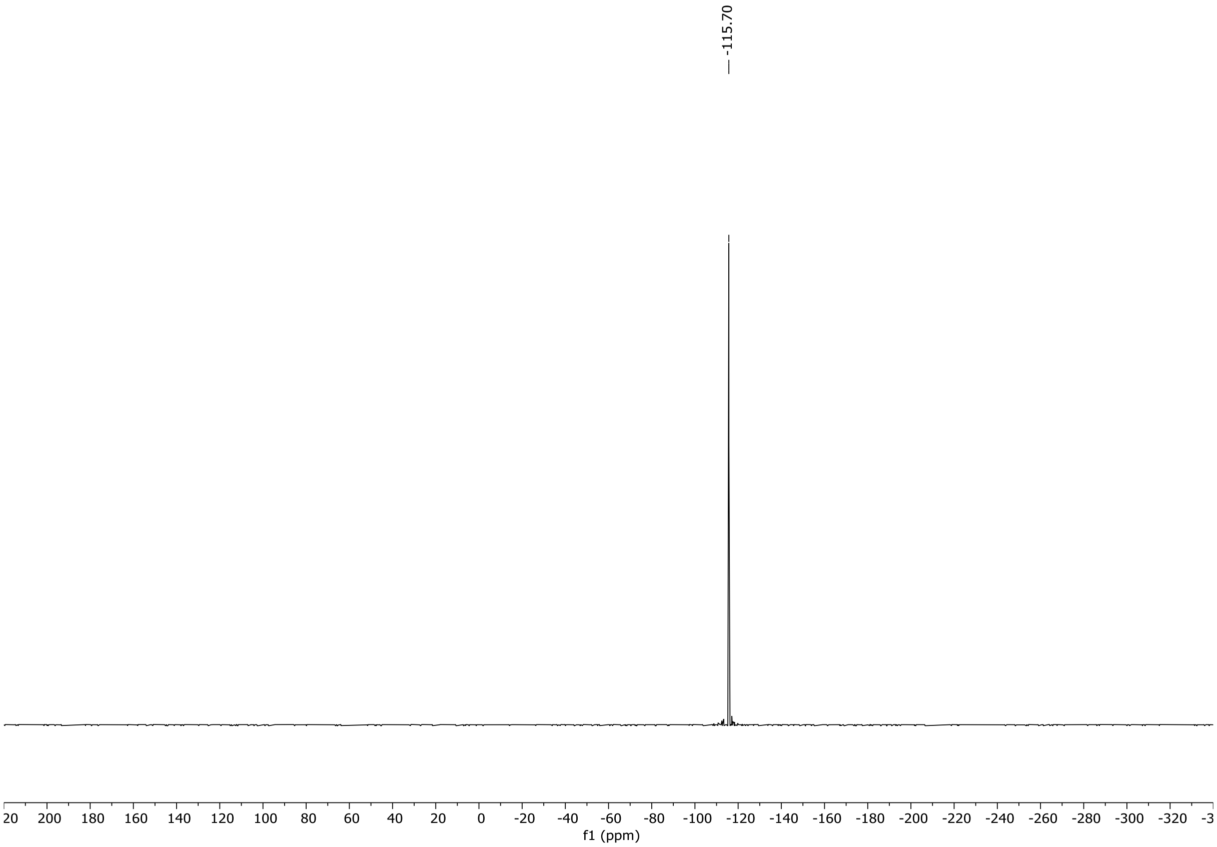

^{19}F-NMR (376 MHz, $CDCl_3$)

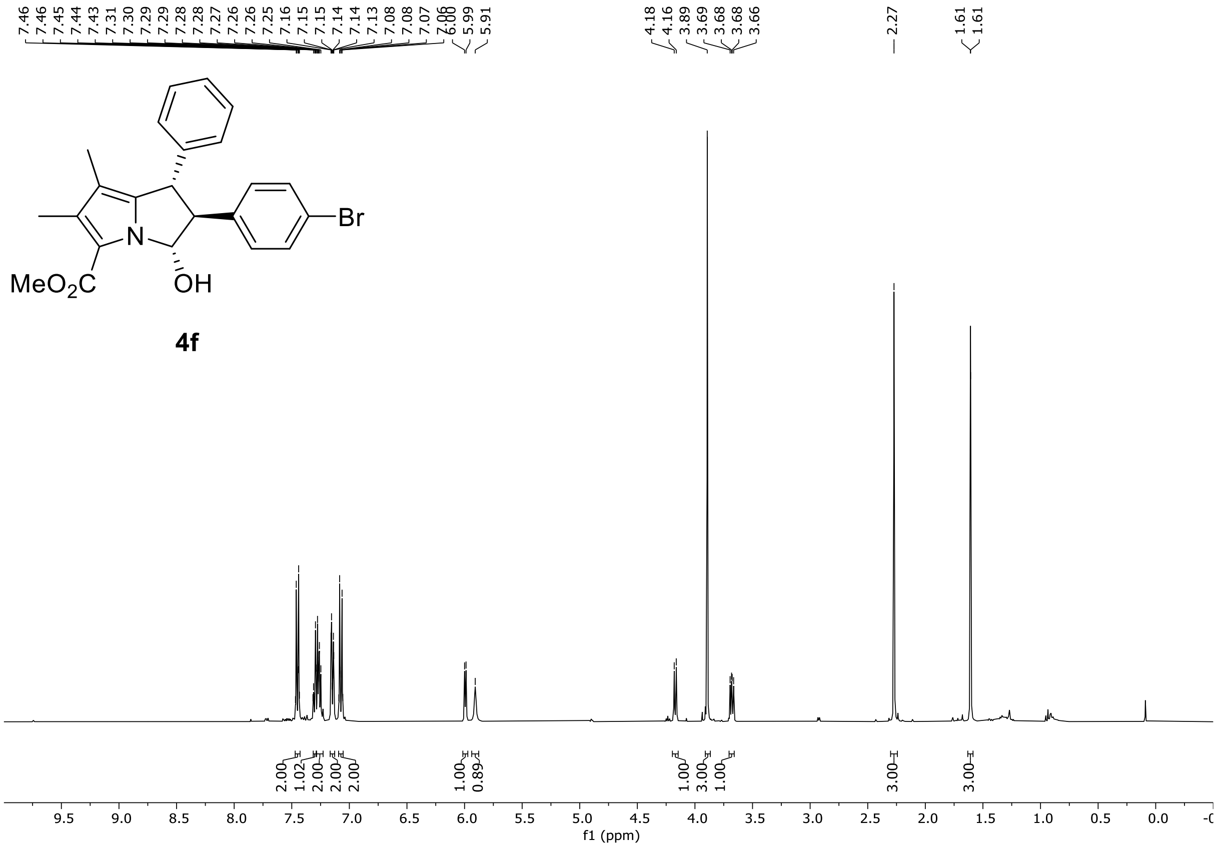

^{1}H-NMR (400 MHz, $CDCl_3$)

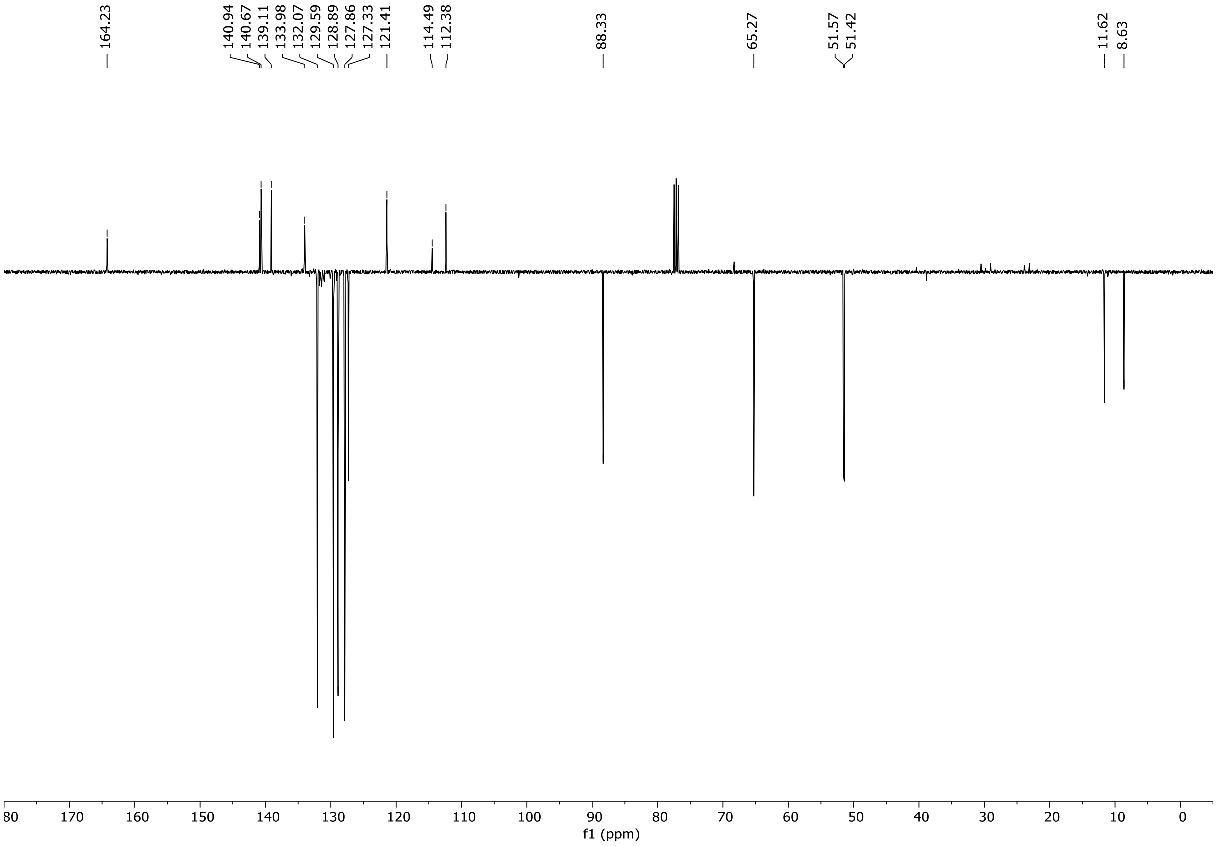

^{13}C-NMR (100 MHz, $CDCl_3$)

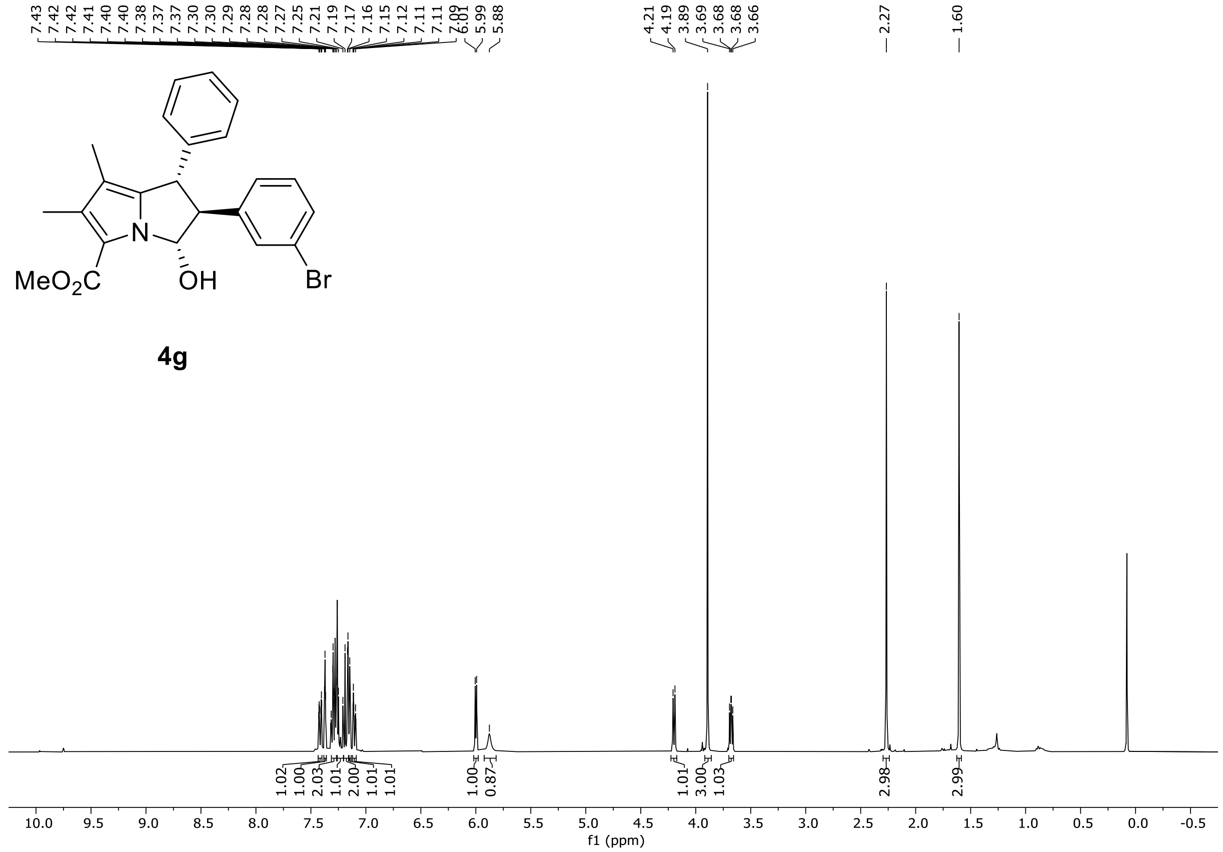

4g

^{1}H-NMR (400 MHz, $CDCl_3$)

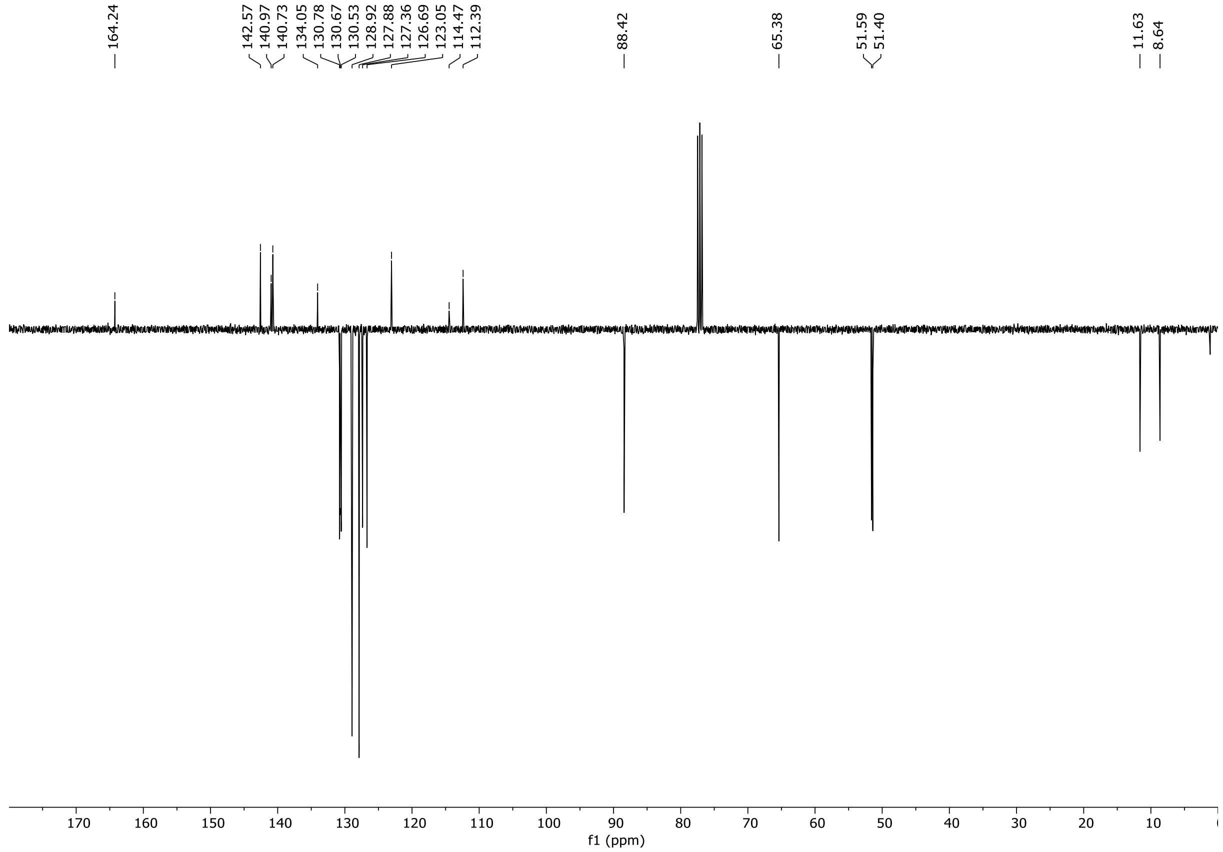

^{13}C-NMR (100 MHz, $CDCl_3$)

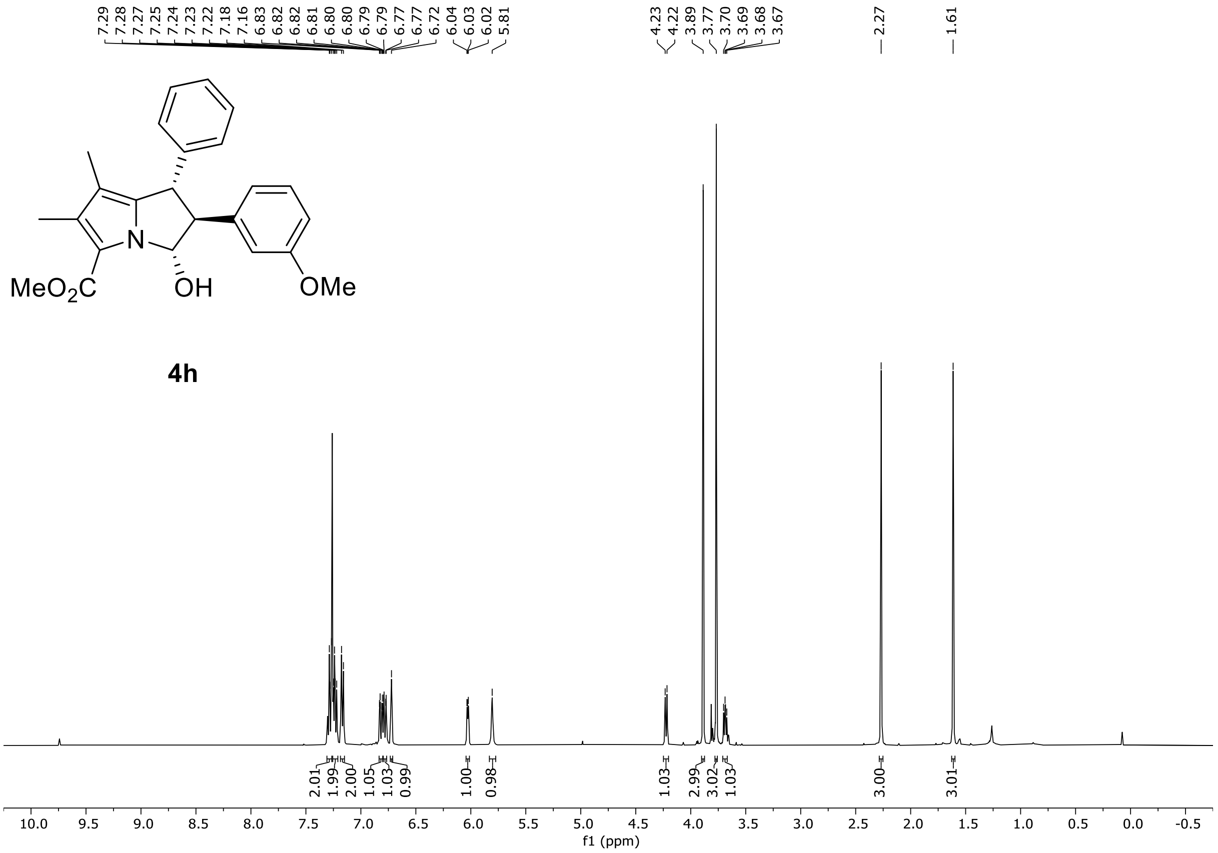

^{1}H-NMR (400 MHz, $CDCl_3$)

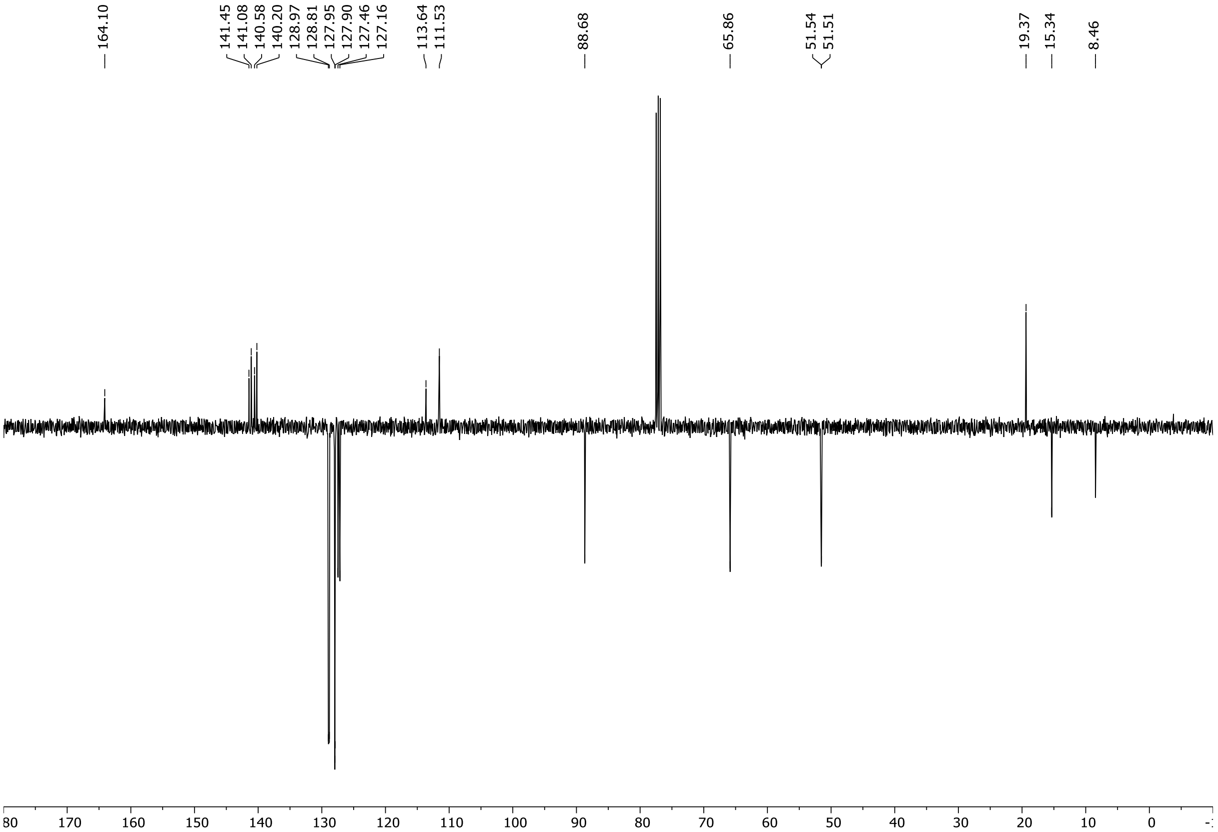

^{13}C-NMR (100 MHz, $CDCl_3$)

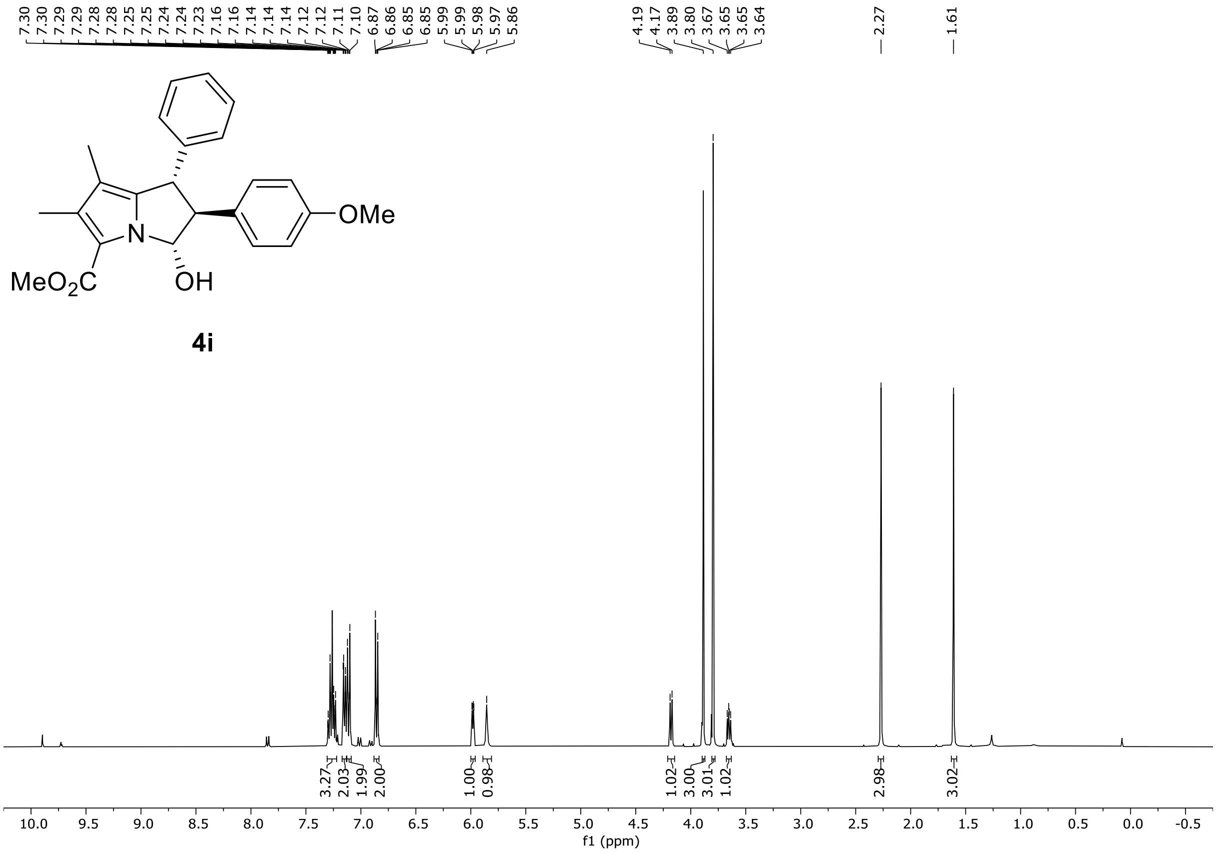

4i

^{1}H-NMR (400 MHz, $CDCl_3$)

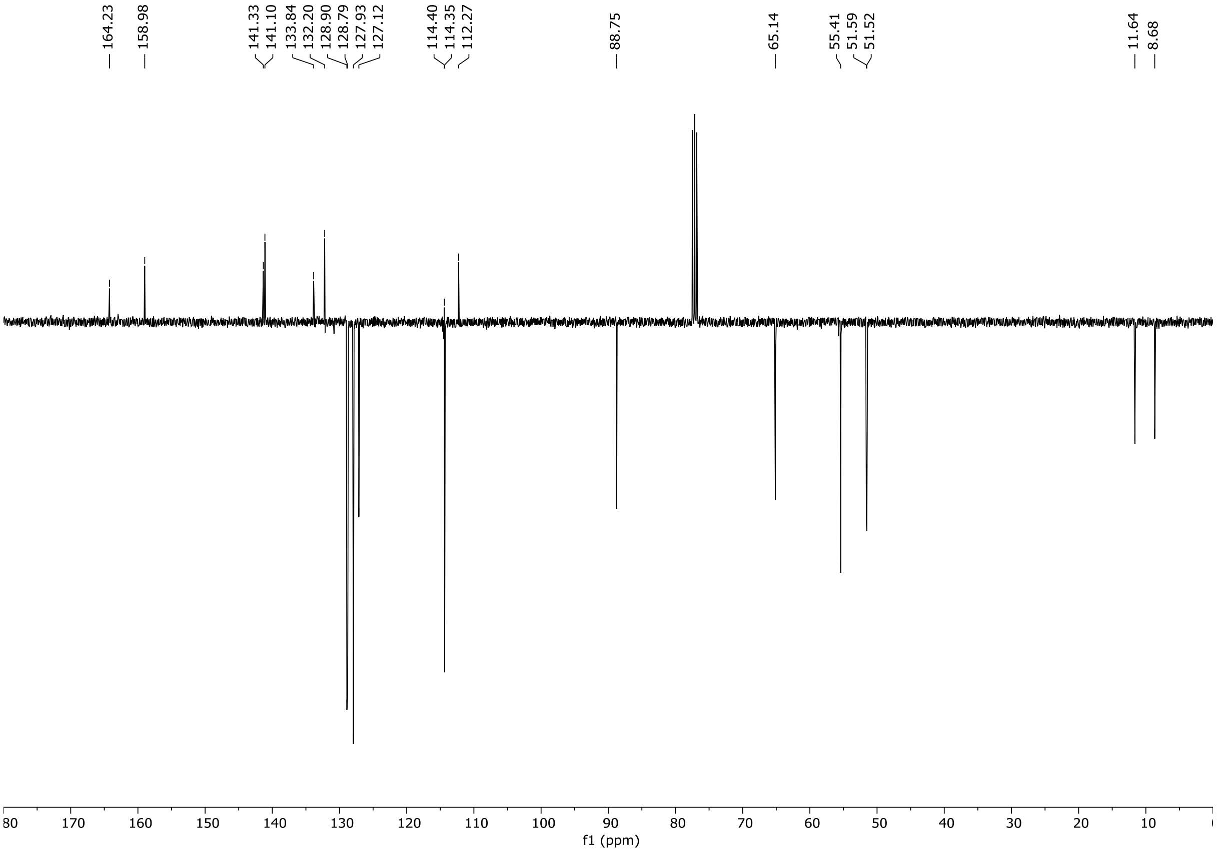

^{13}C-NMR (100 MHz, $CDCl_3$)

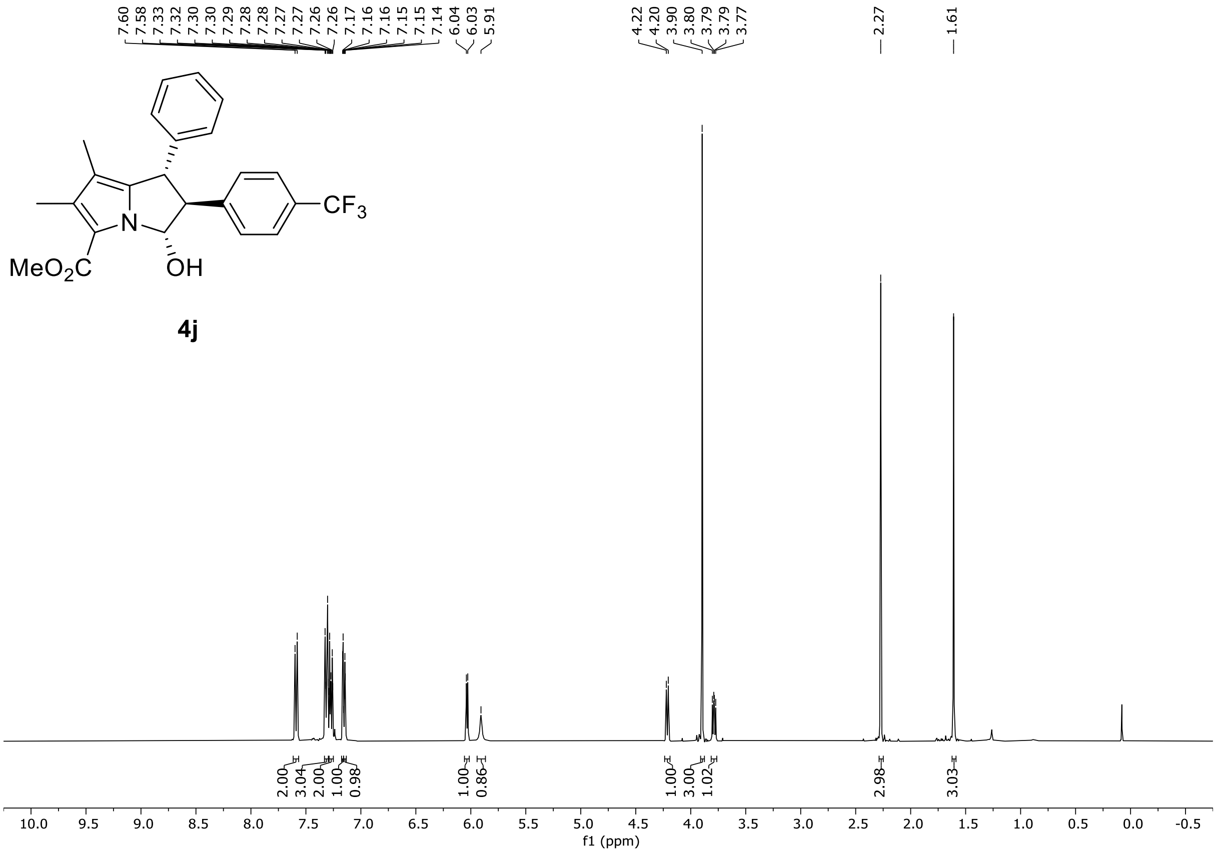

^{1}H-NMR (400 MHz, $CDCl_3$)

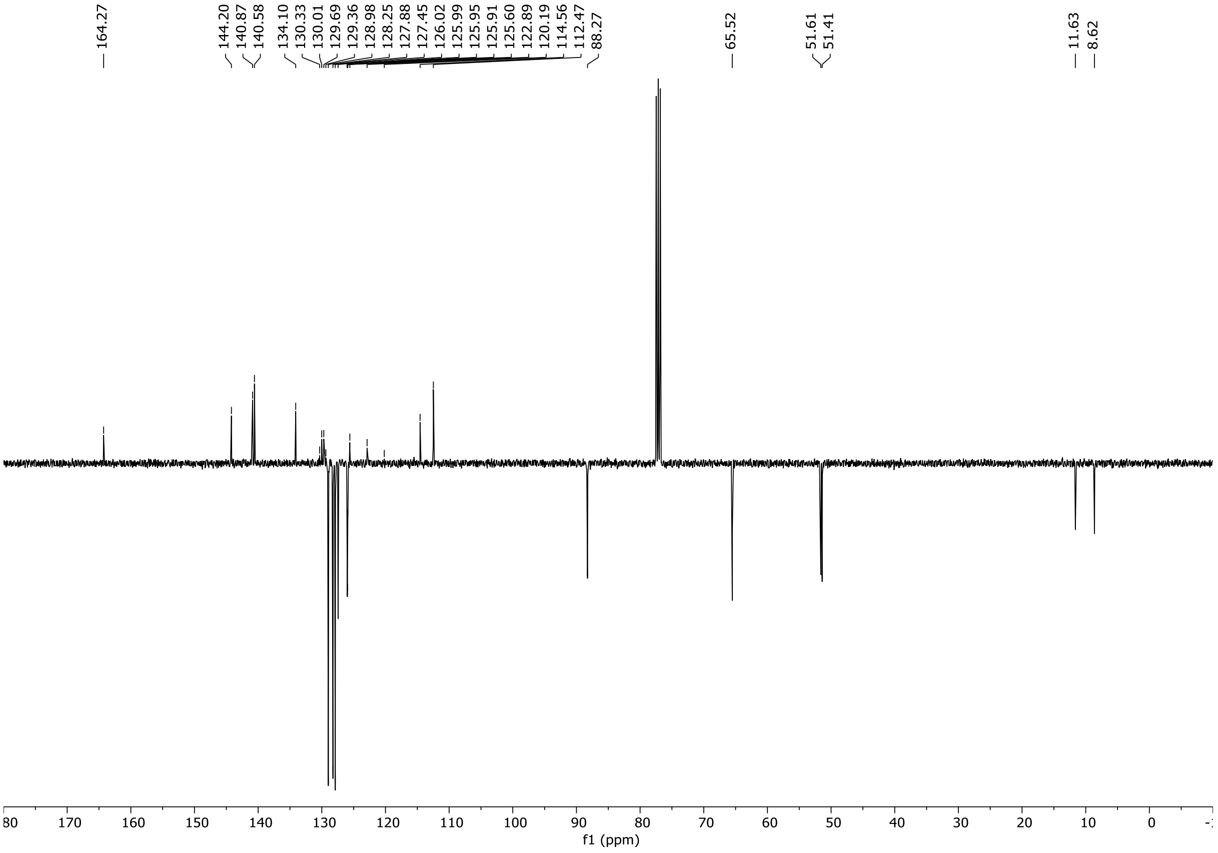

^{13}C-NMR (100 MHz, $CDCl_3$)

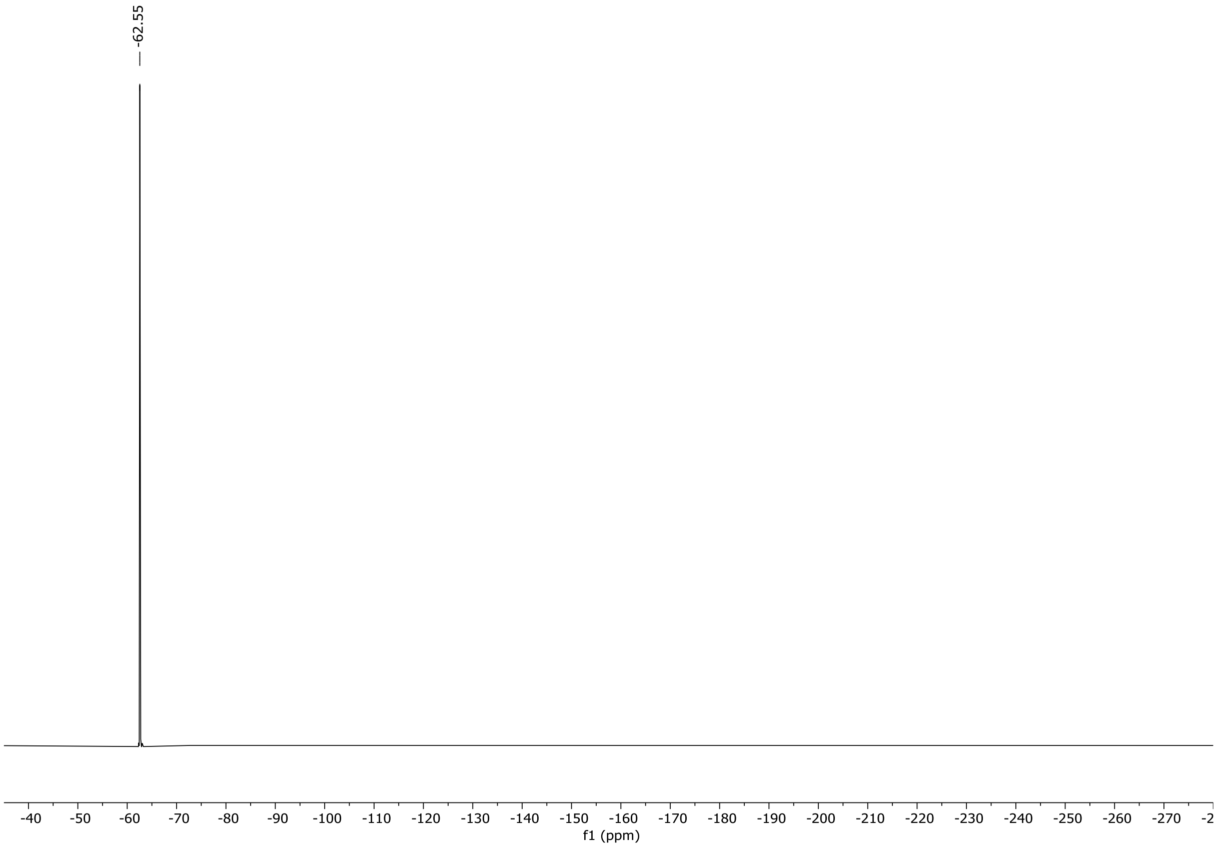

^{19}F-NMR (376 MHz, $CDCl_3$)

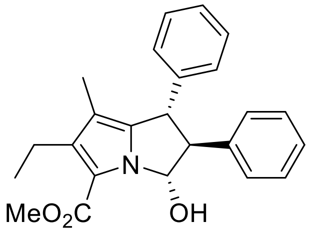

4k

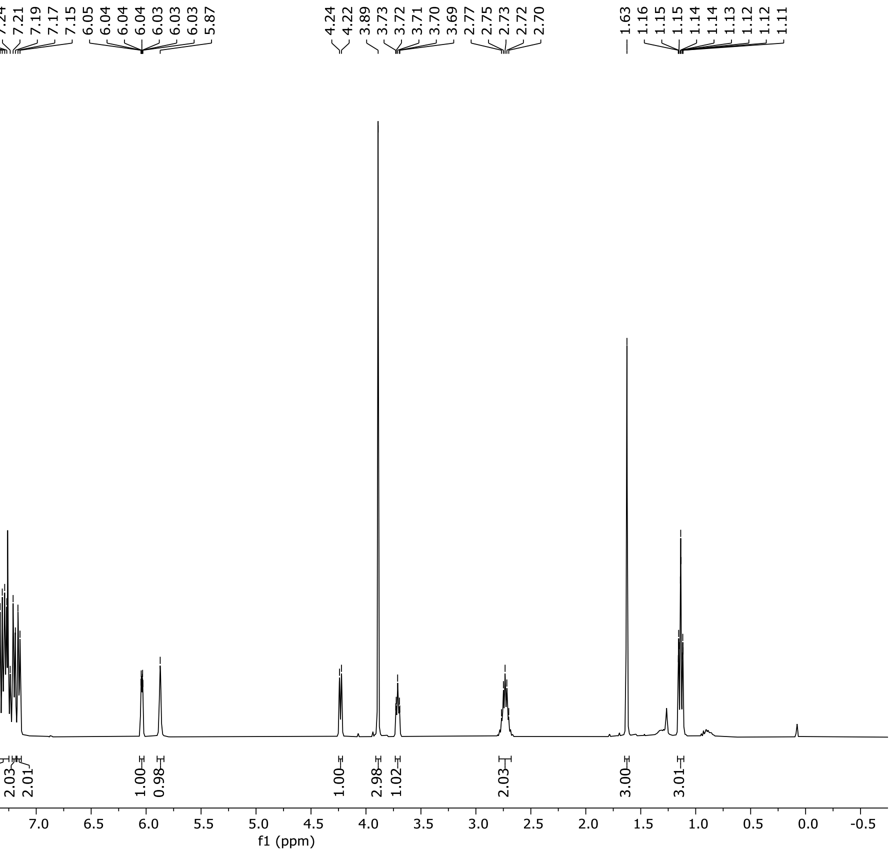

^{1}H-NMR (400 MHz, $CDCl_3$)

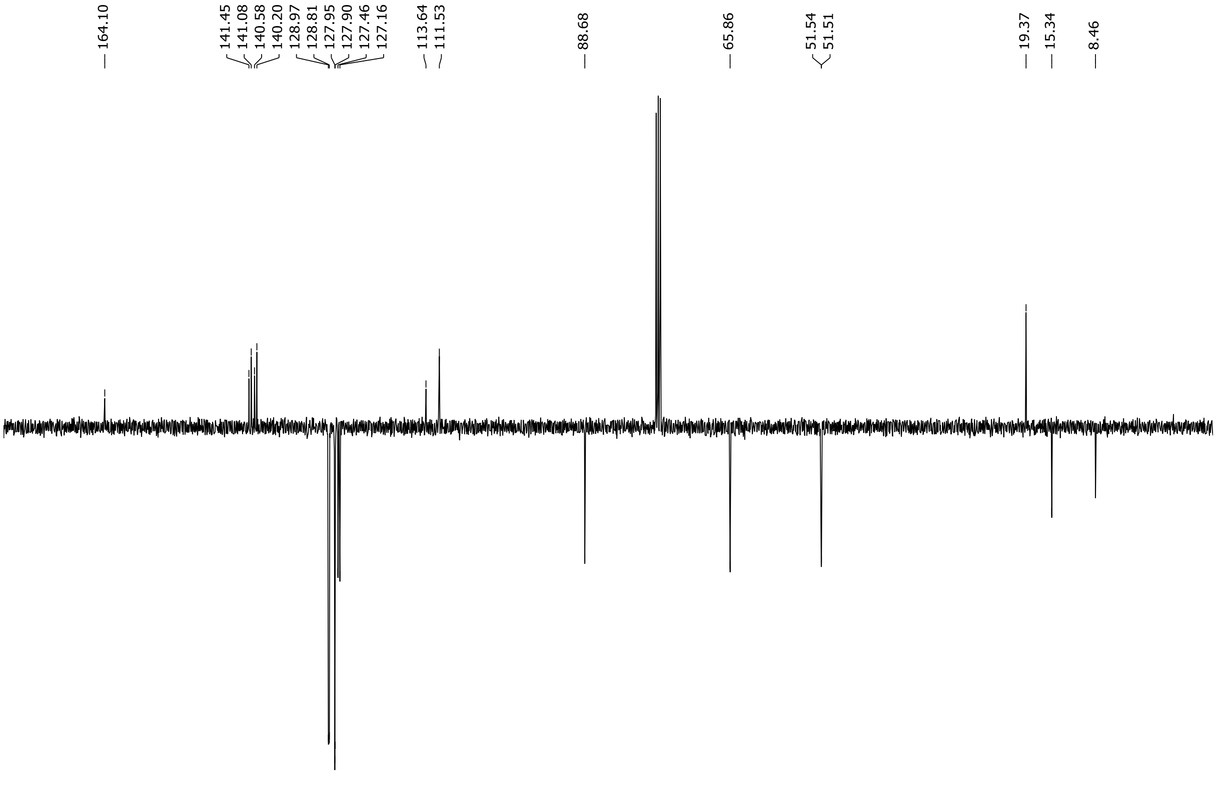

^{13}C-NMR (100 MHz, $CDCl_3$)

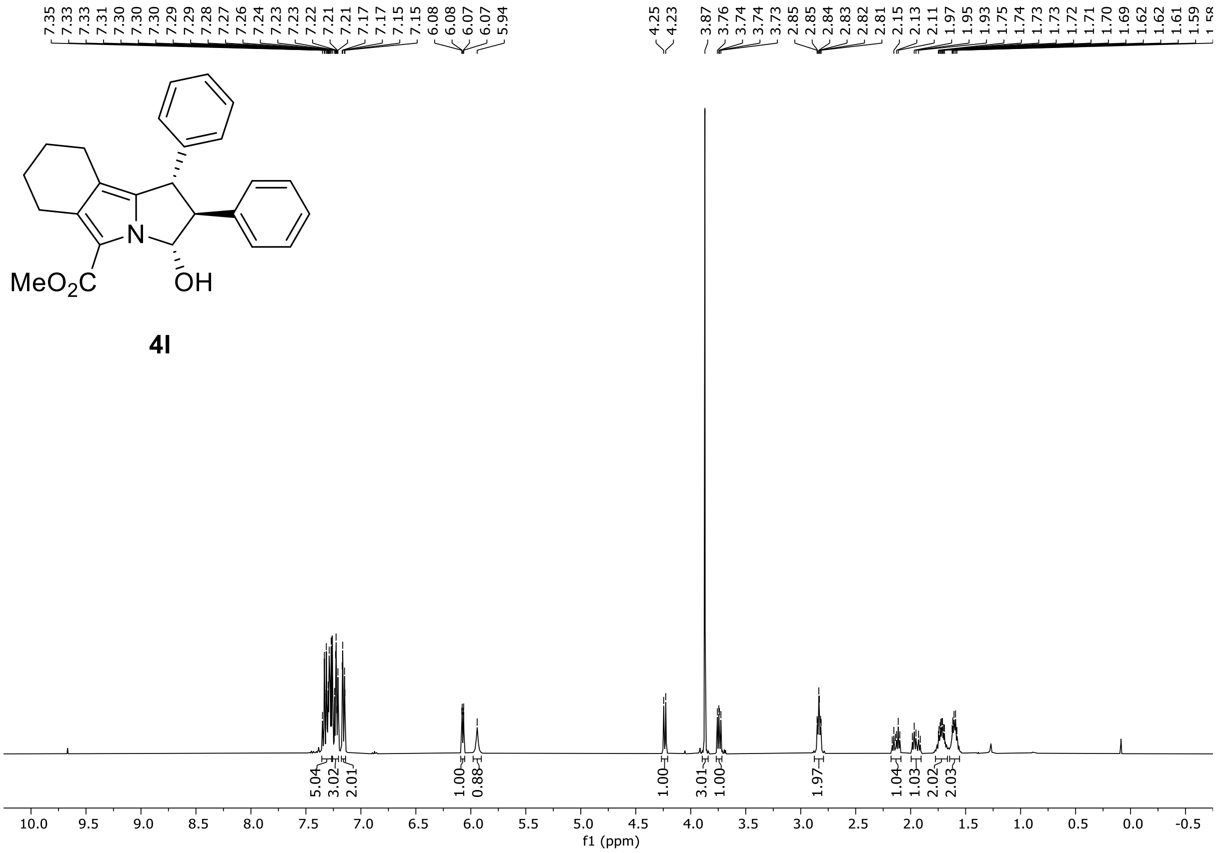

^{1}H-NMR (400 MHz, $CDCl_3$)

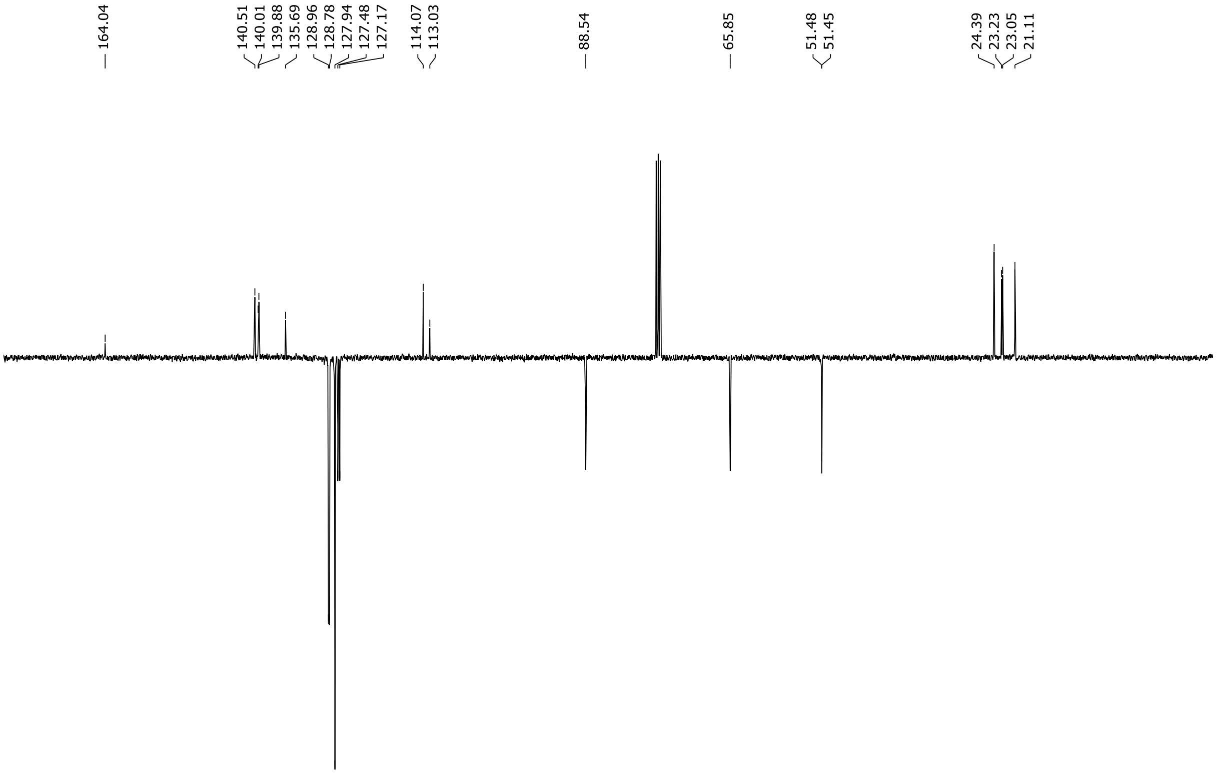

^{13}C-NMR (100 MHz, $CDCl_3$)

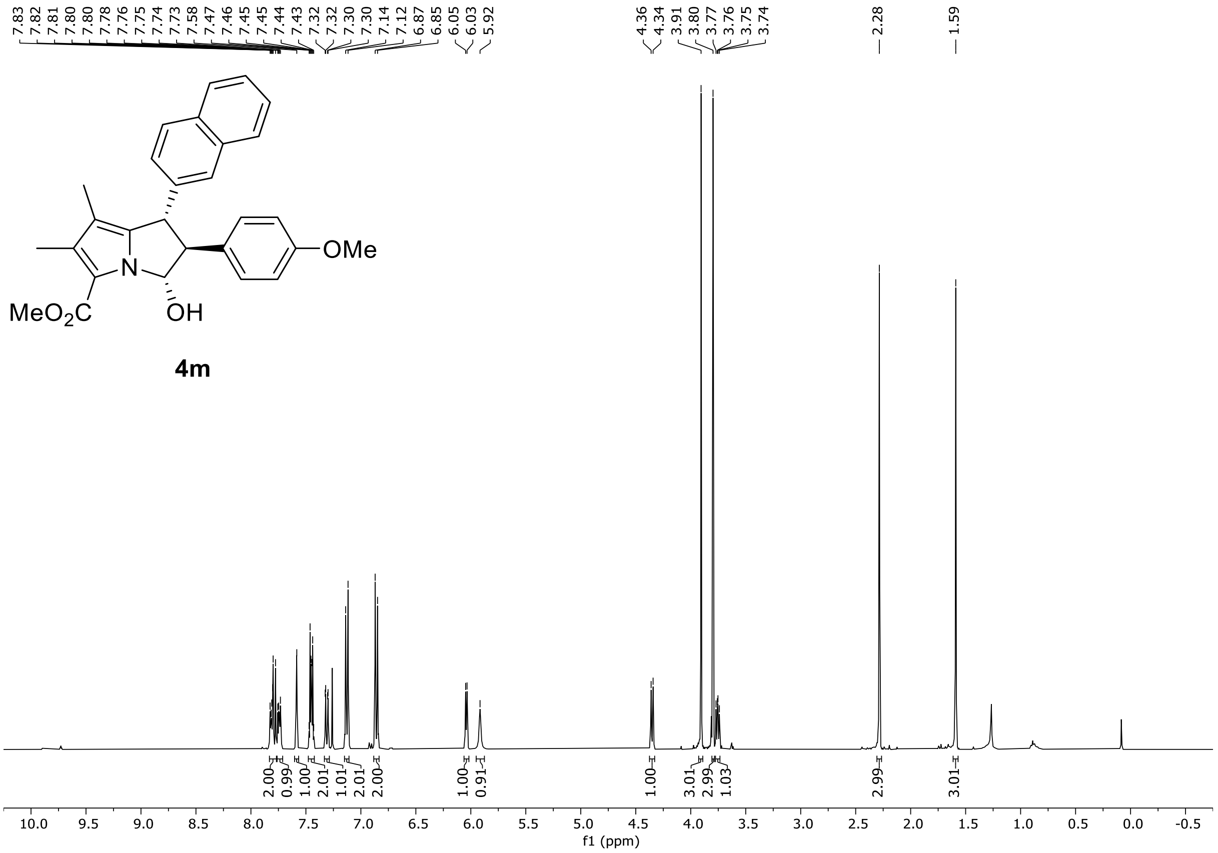

^{1}H-NMR (400 MHz, $CDCl_3$)

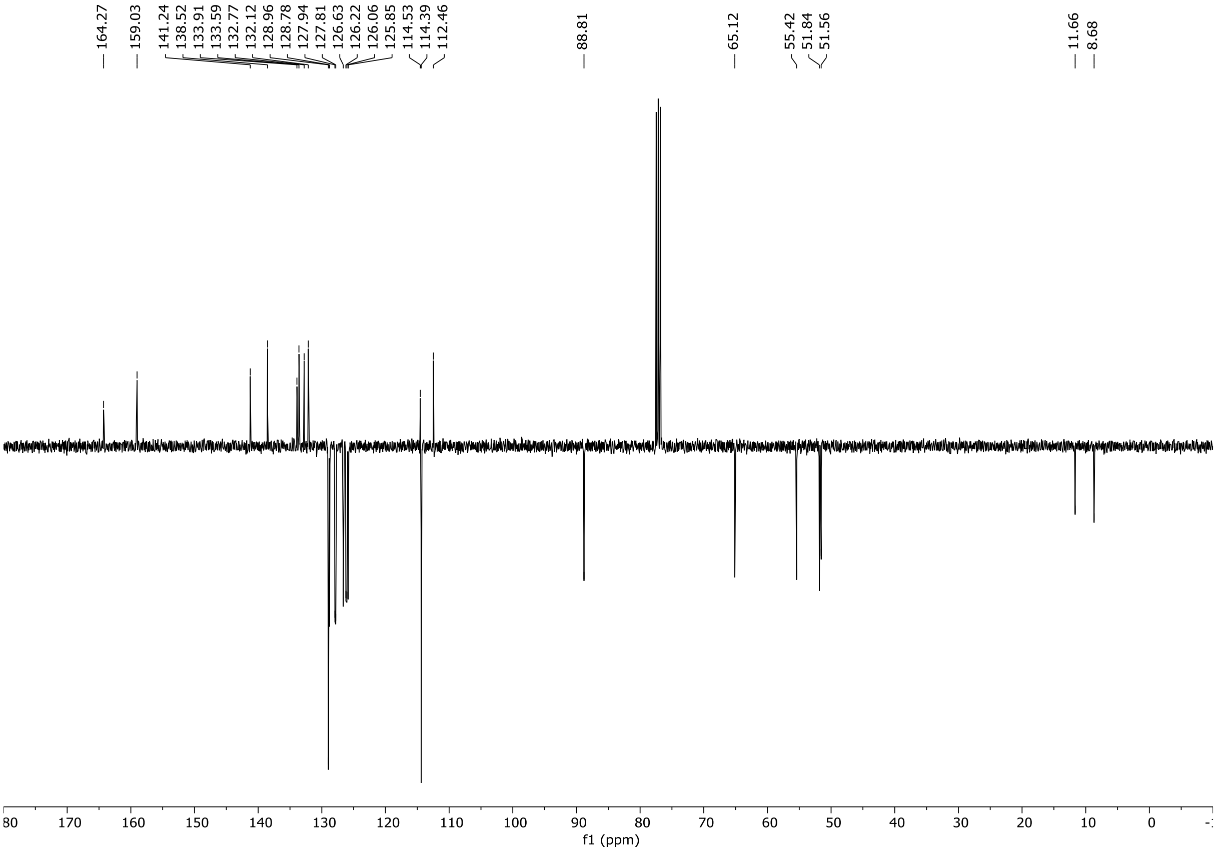

^{13}C-NMR (100 MHz, $CDCl_3$)

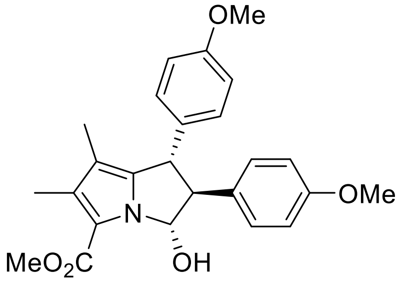

4n

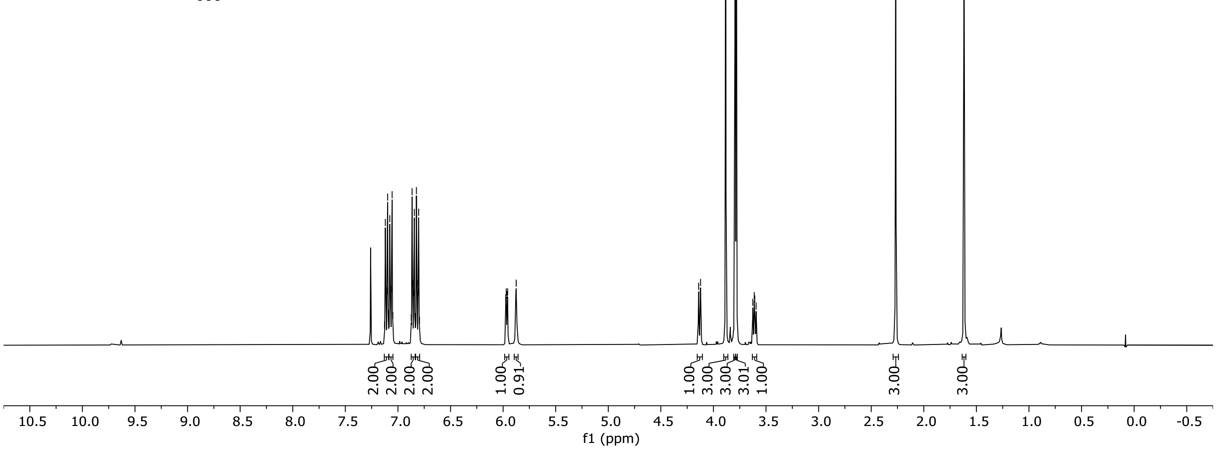

^{1}H-NMR (400 MHz, $CDCl_3$)

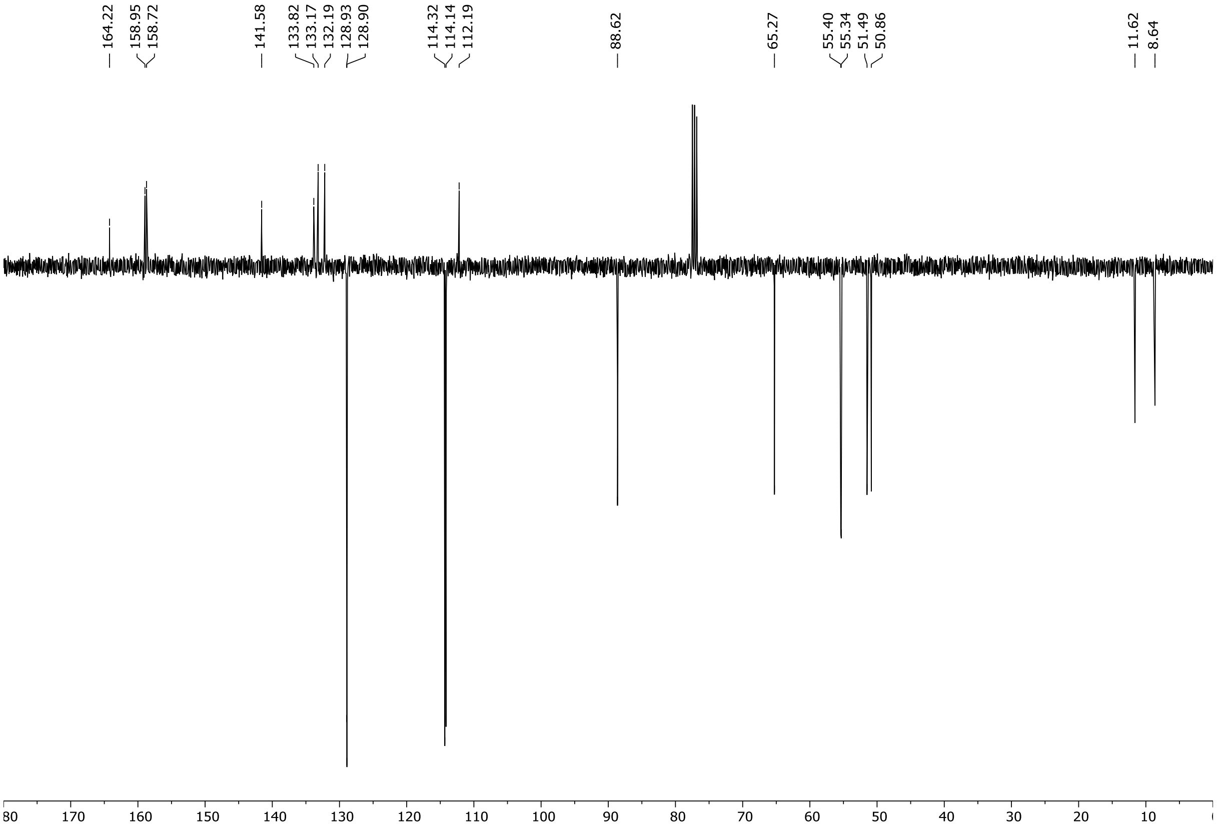

^{13}C-NMR (100 MHz, $CDCl_3$)

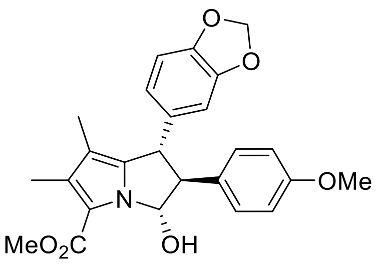

4o

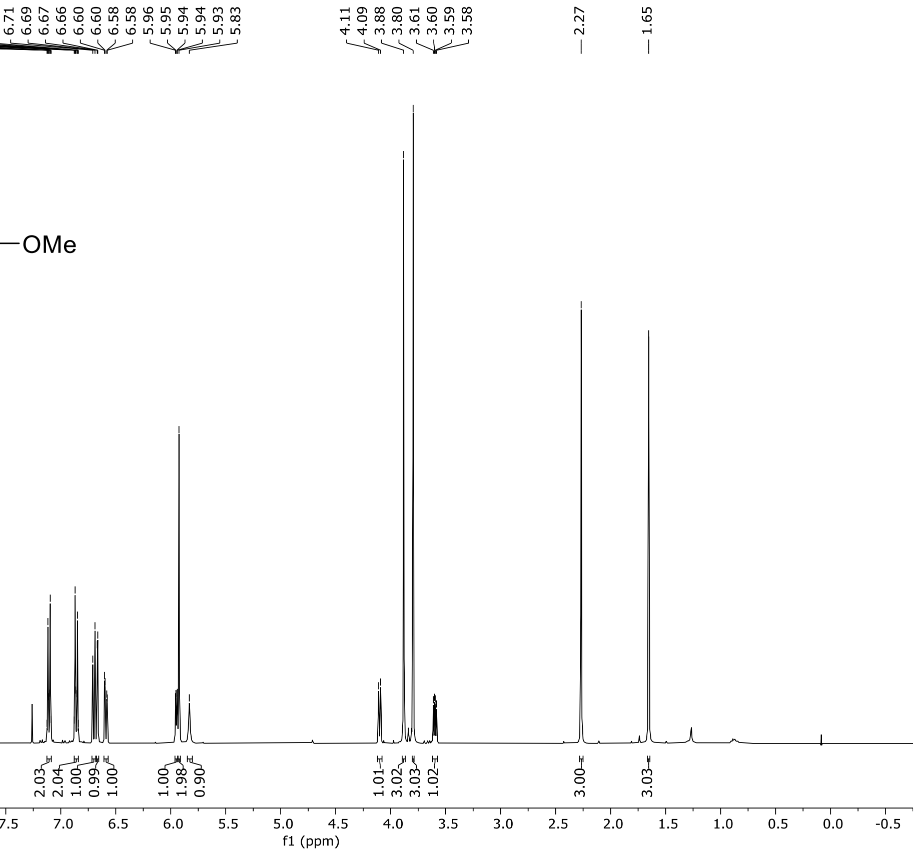

^{1}H-NMR (400 MHz, $CDCl_3$)

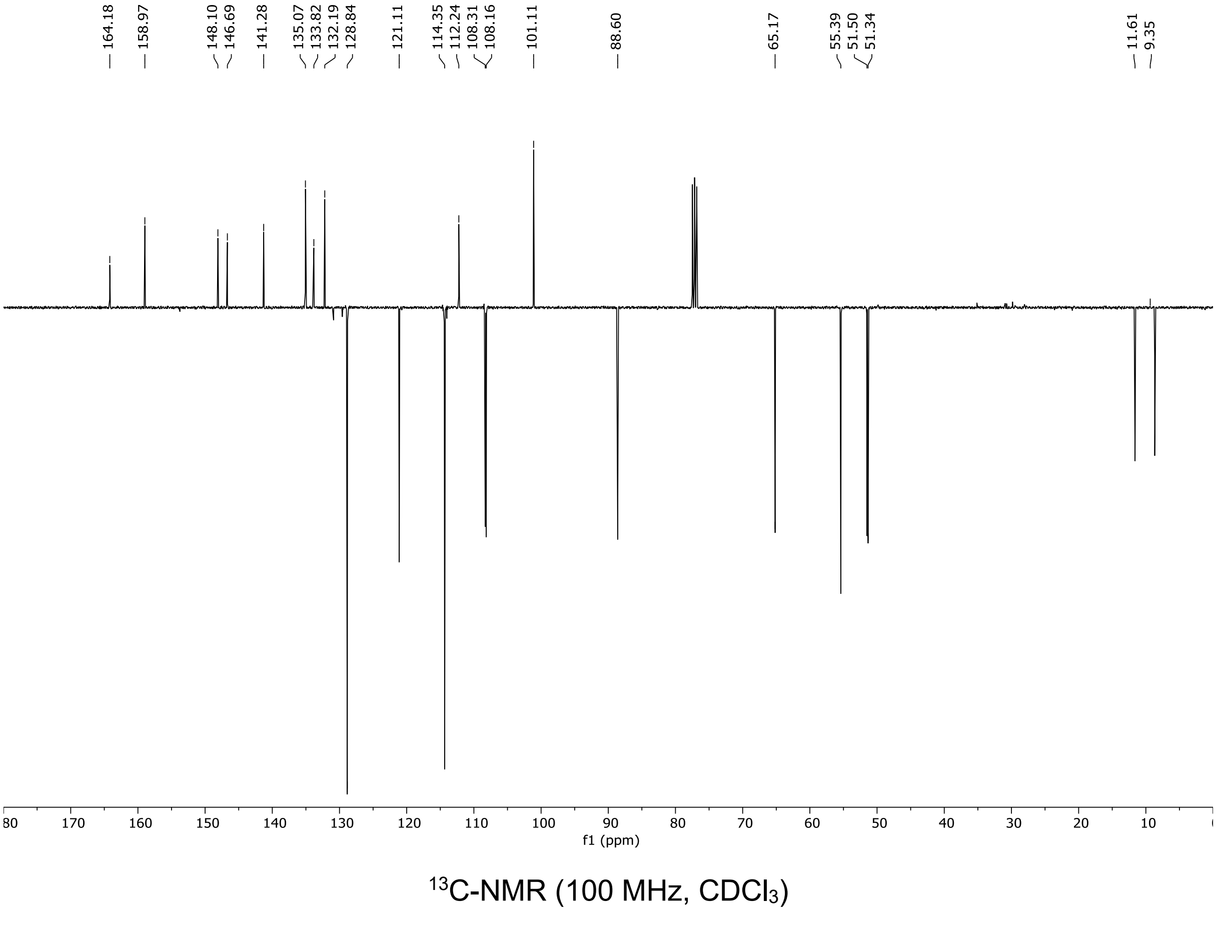

^{13}C-NMR (100 MHz, $CDCl_3$)

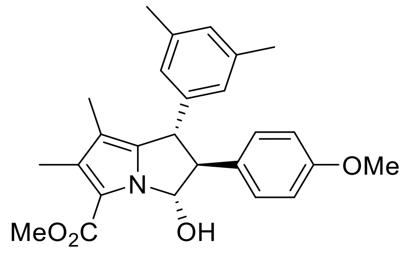

4p

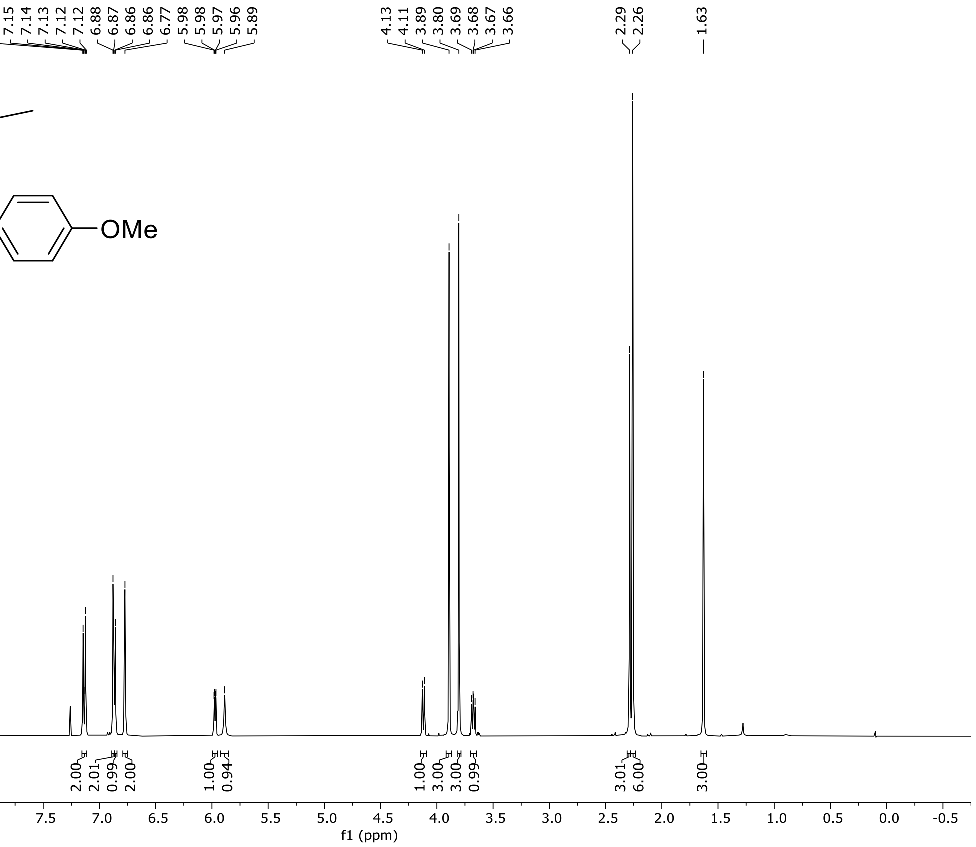

^{1}H-NMR (400 MHz, $CDCl_3$)

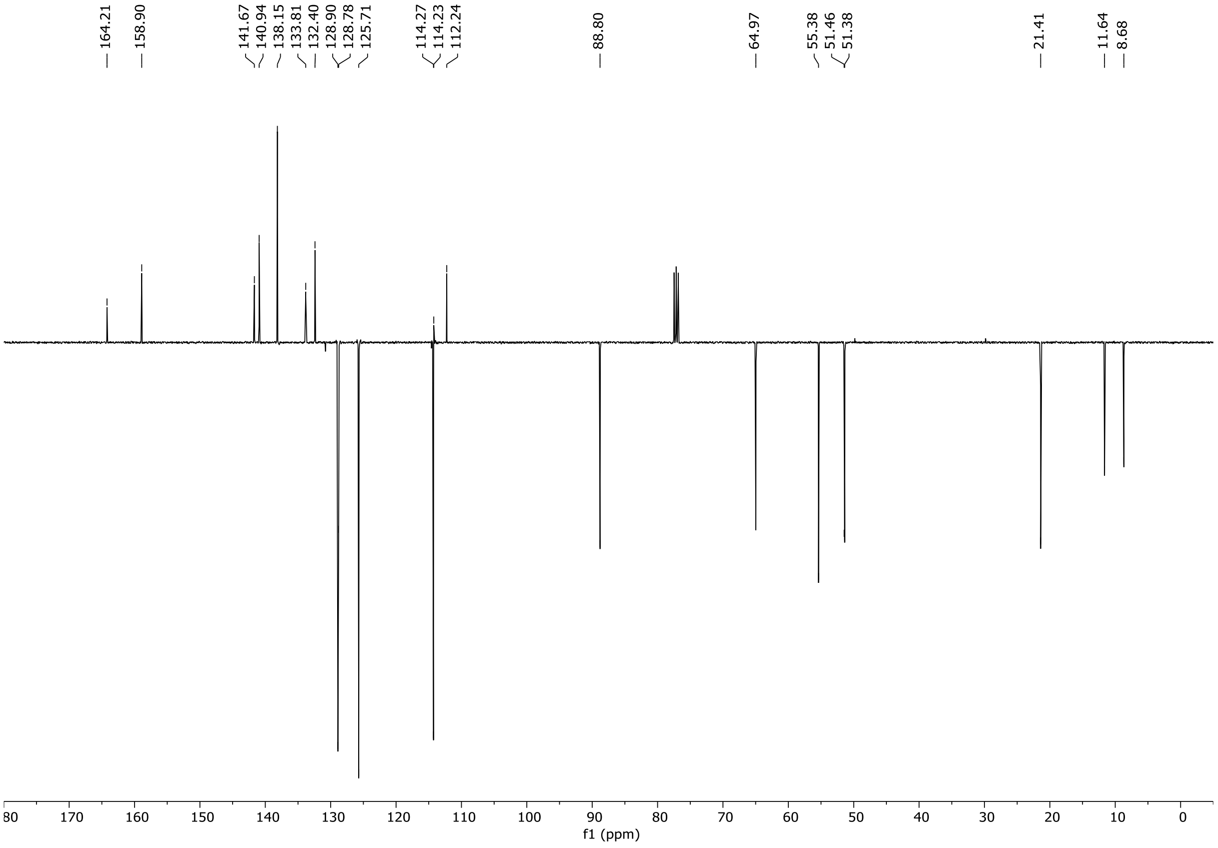

^{13}C-NMR (100 MHz, $CDCl_3$)

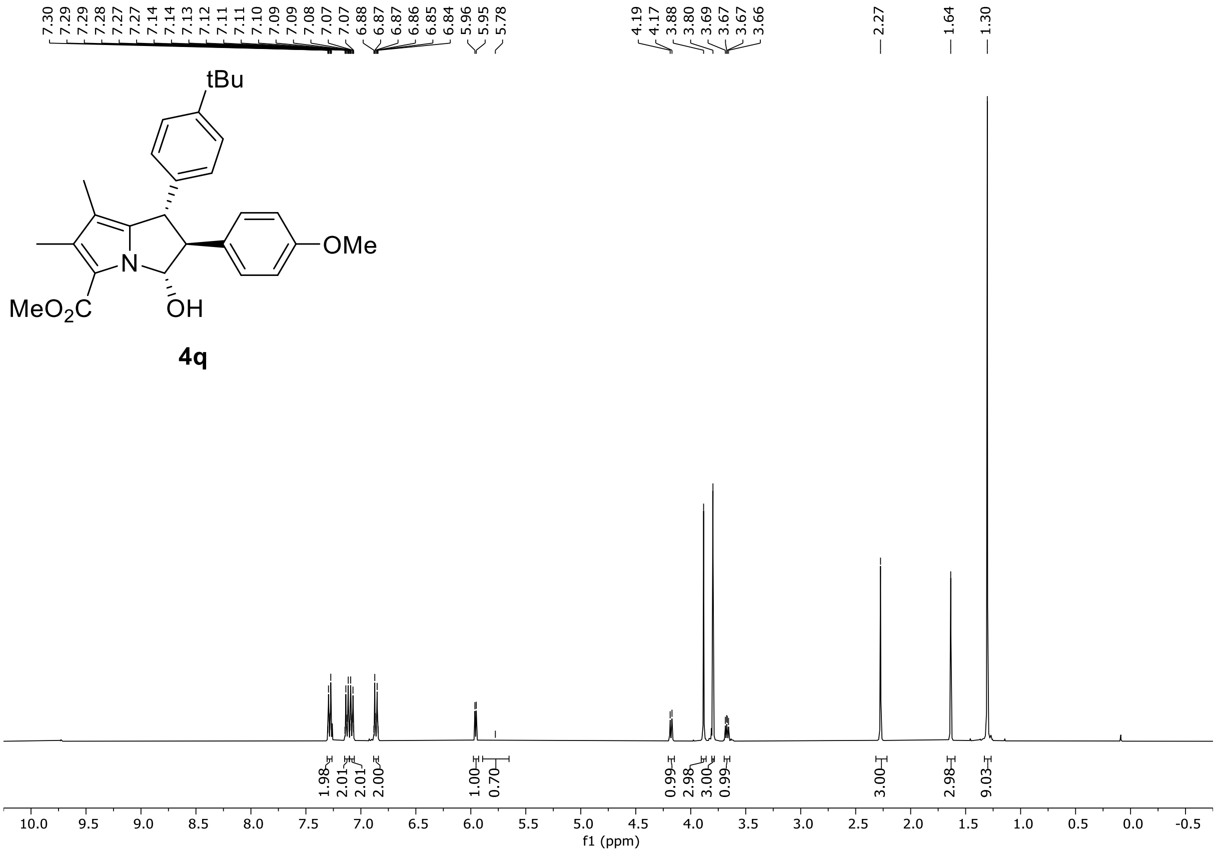

^{1}H-NMR (400 MHz, $CDCl_3$)

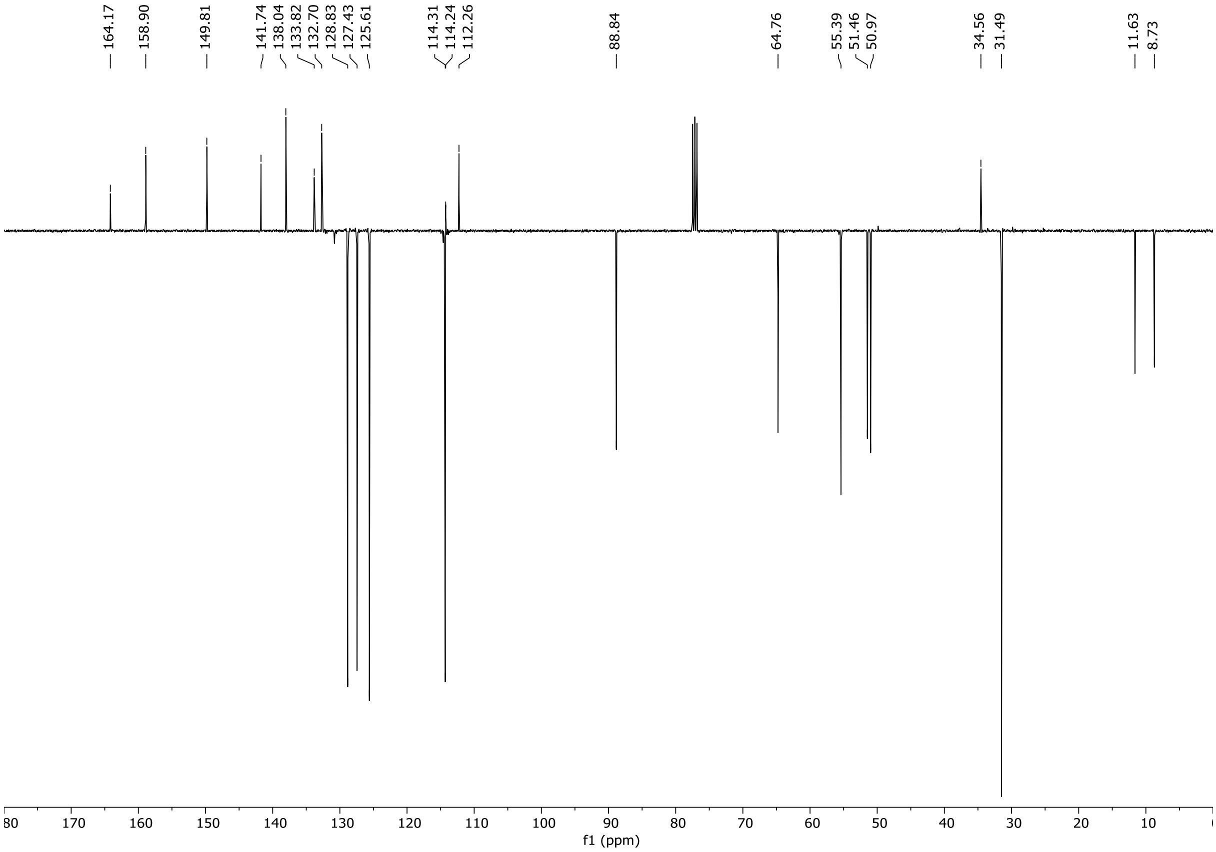

^{13}C-NMR (100 MHz, $CDCl_3$)

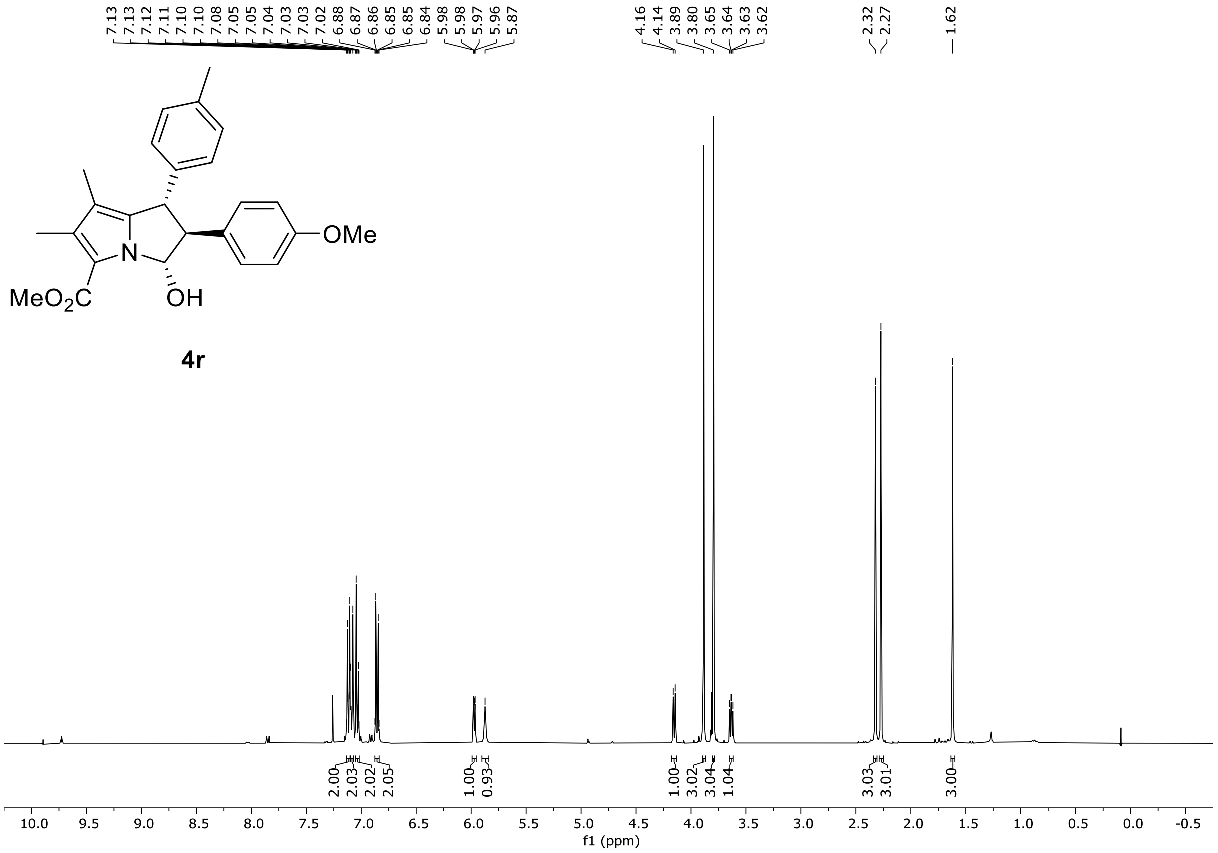

^{1}H-NMR (400 MHz, $CDCl_3$)

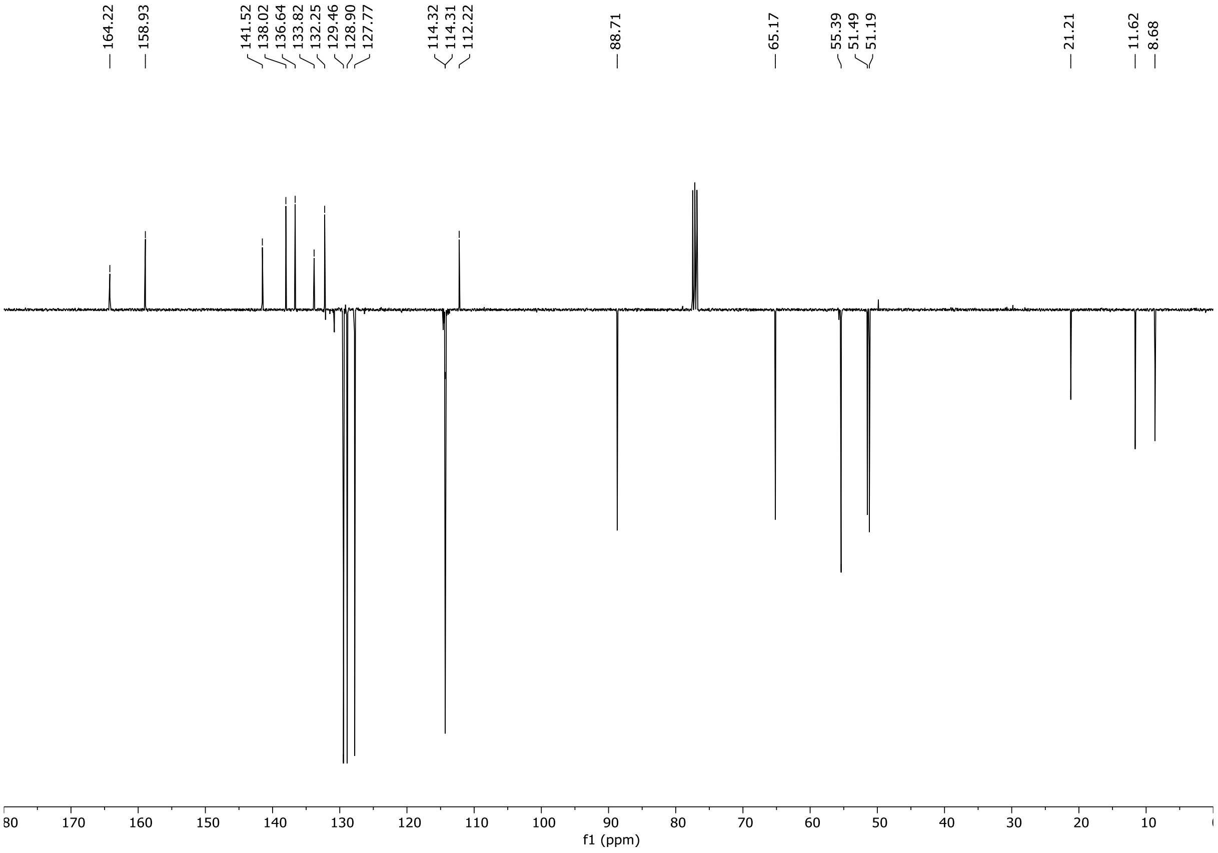

^{13}C-NMR (100 MHz, $CDCl_3$)

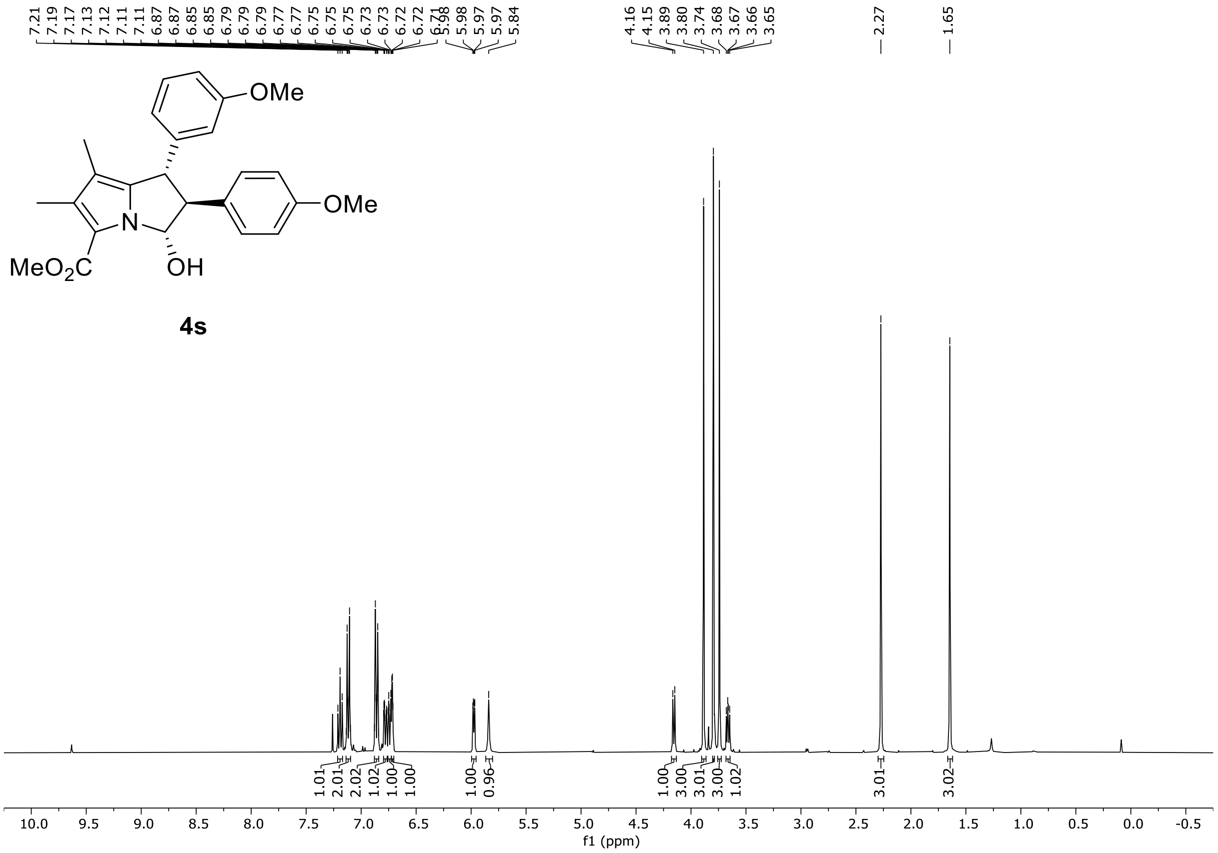

^{1}H-NMR (400 MHz, $CDCl_3$)

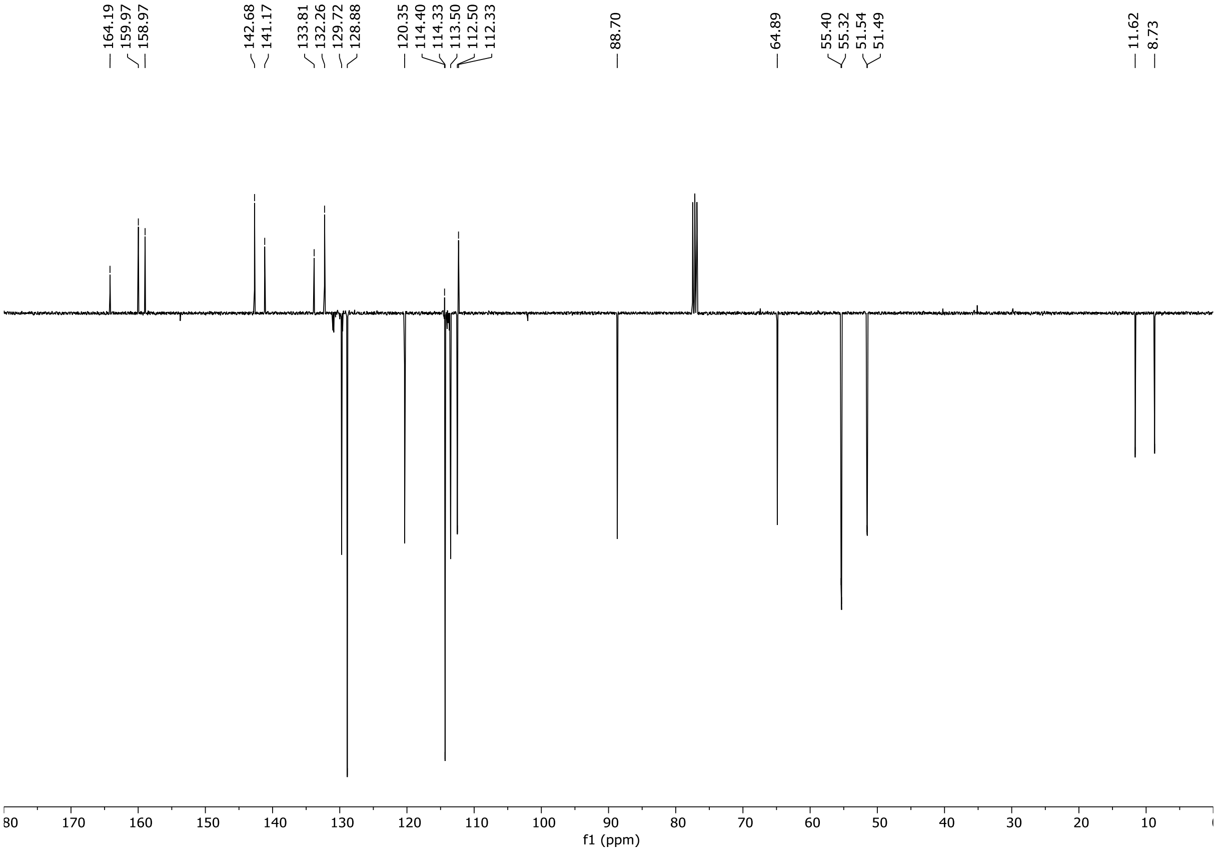

^{13}C-NMR (100 MHz, $CDCl_3$)

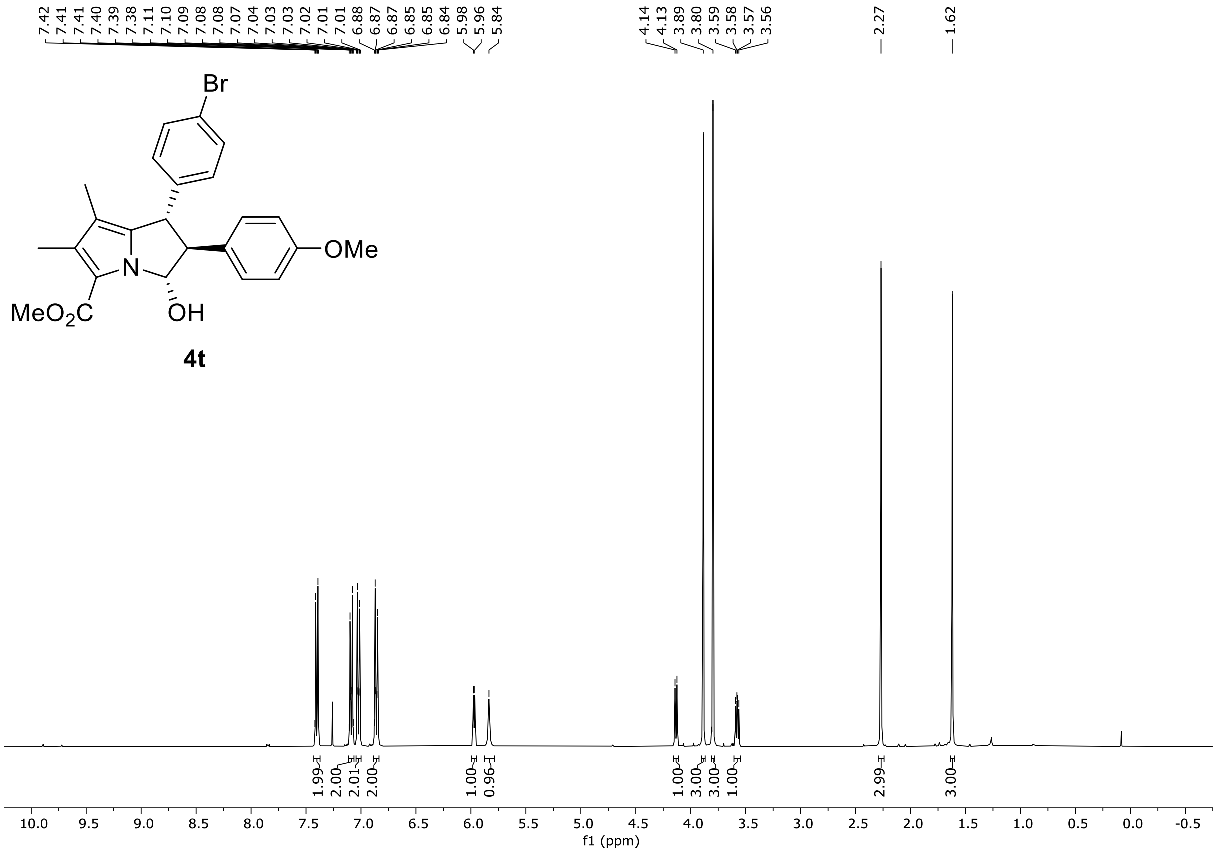

^{1}H-NMR (400 MHz, $CDCl_3$)

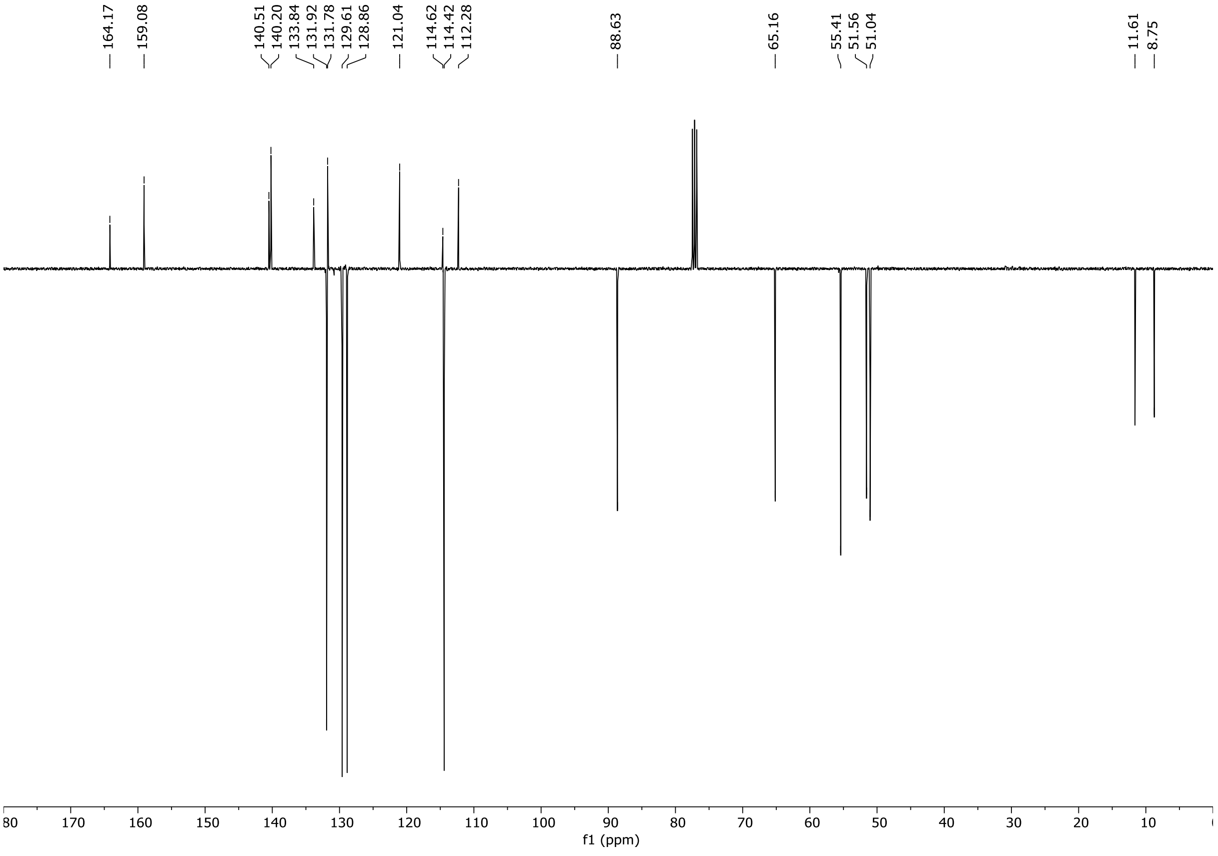

^{13}C-NMR (100 MHz, $CDCl_3$)

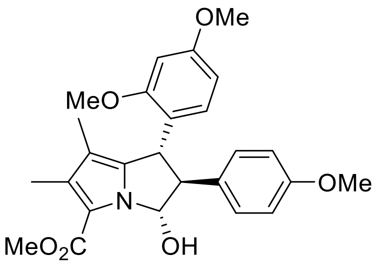

4u

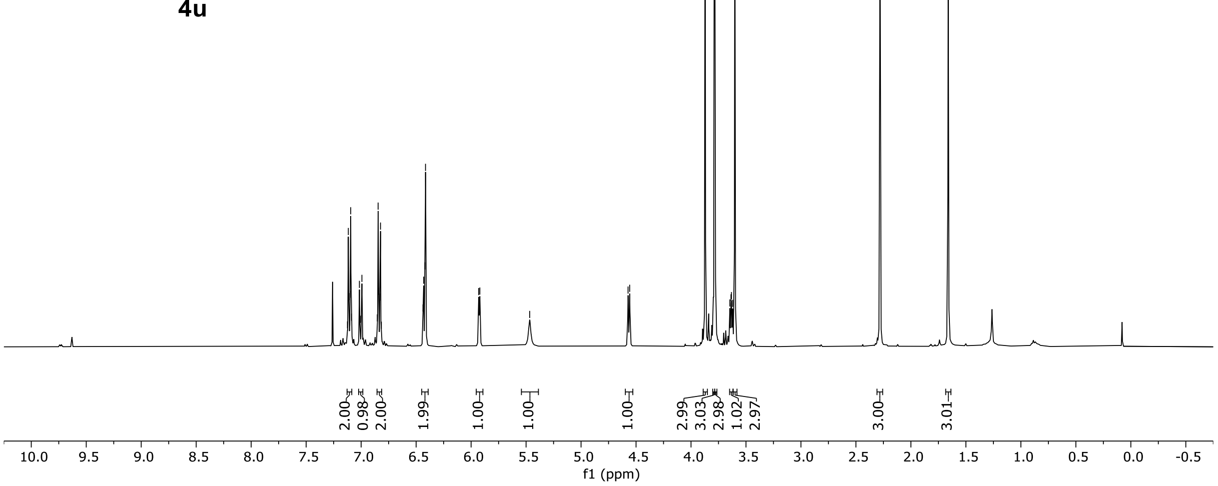

^{1}H-NMR (400 MHz, $CDCl_3$)

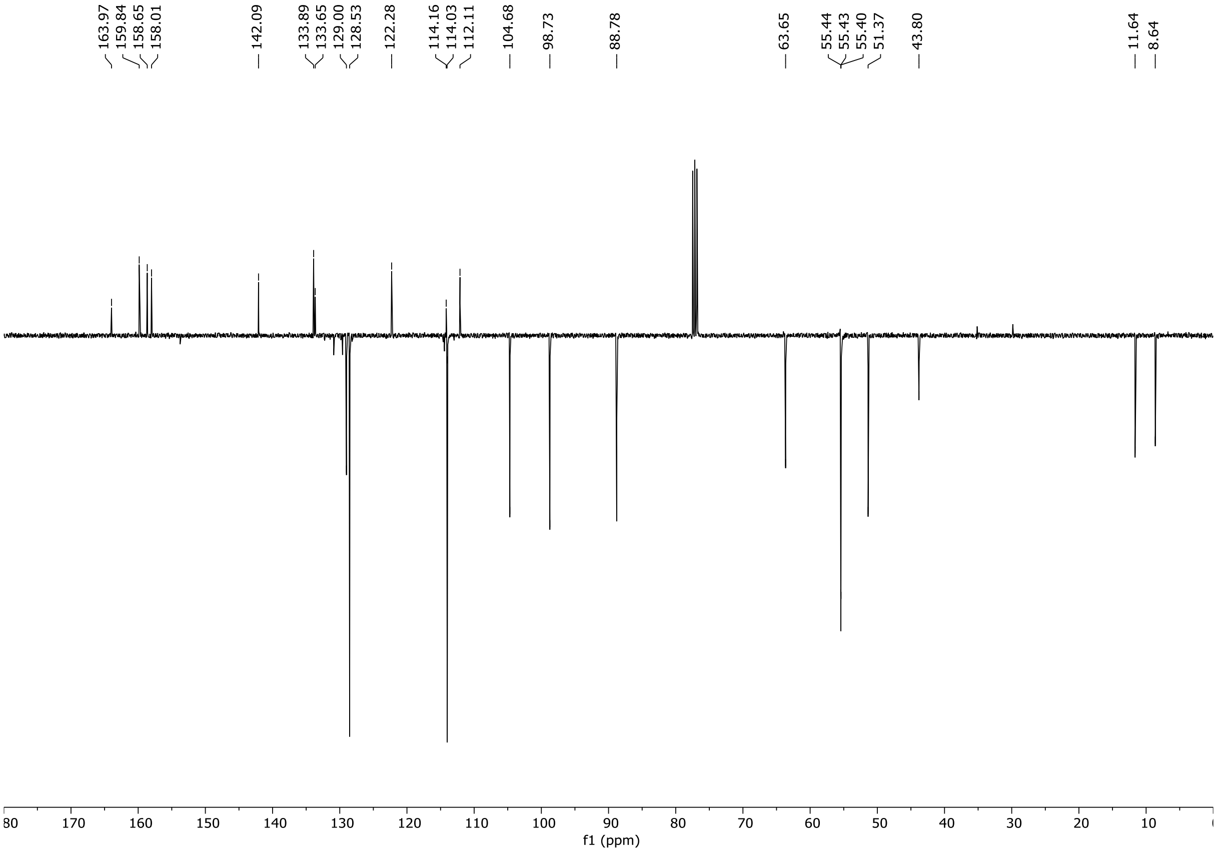

^{13}C-NMR (100 MHz, $CDCl_3$)

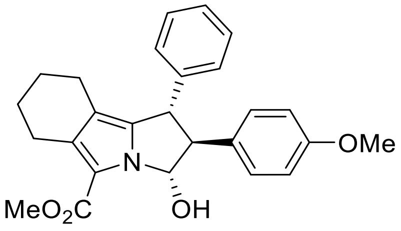

4v

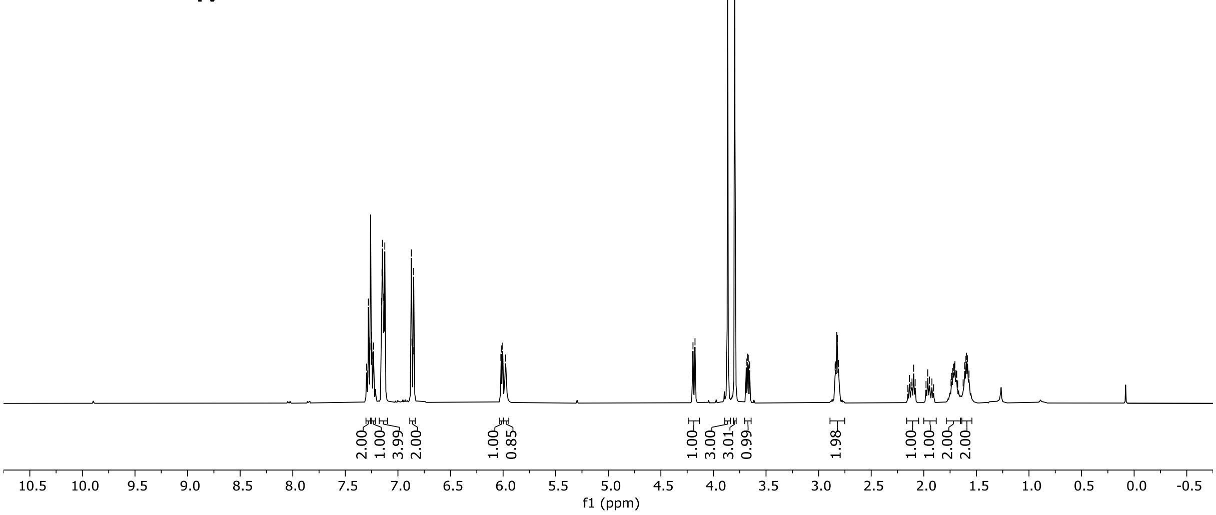

^{1}H-NMR (400 MHz, $CDCl_3$)

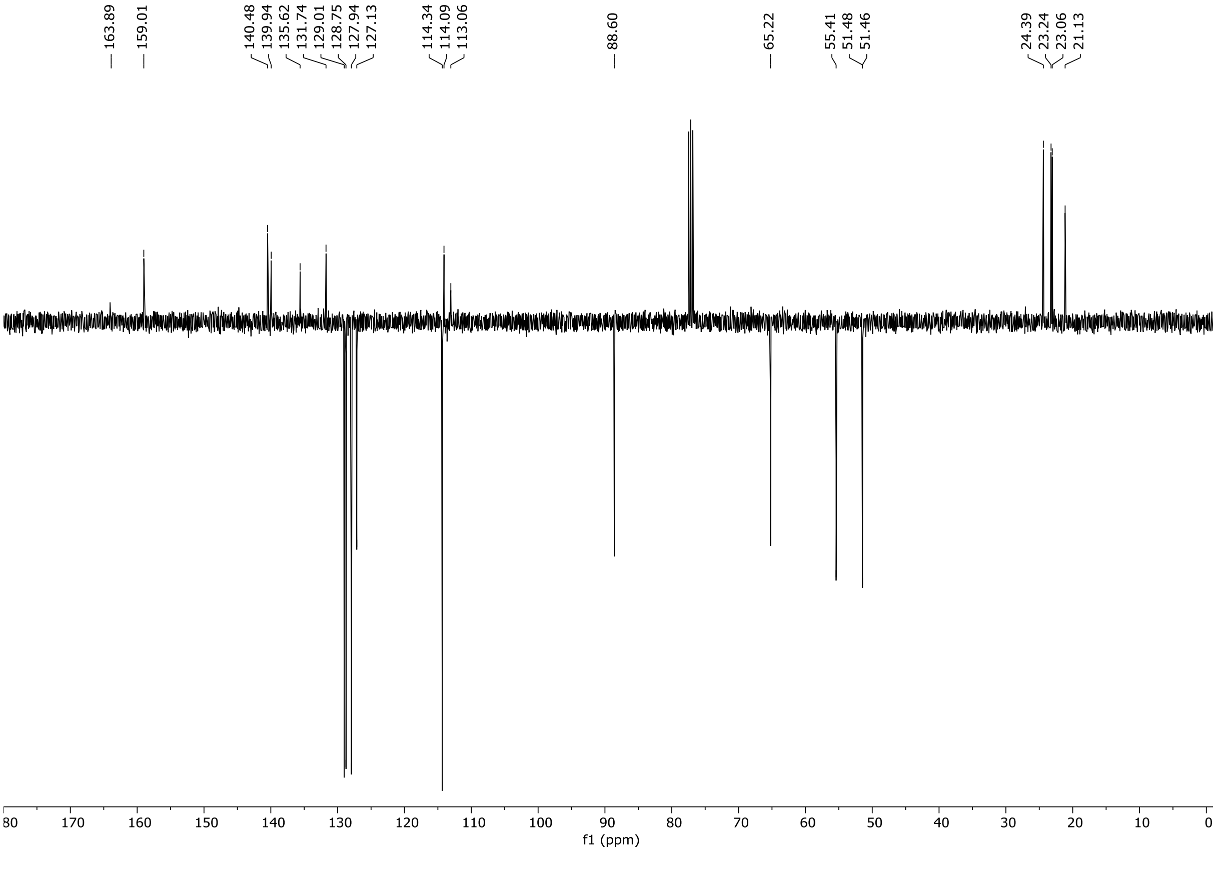

^{13}C-NMR (100 MHz, $CDCl_3$)

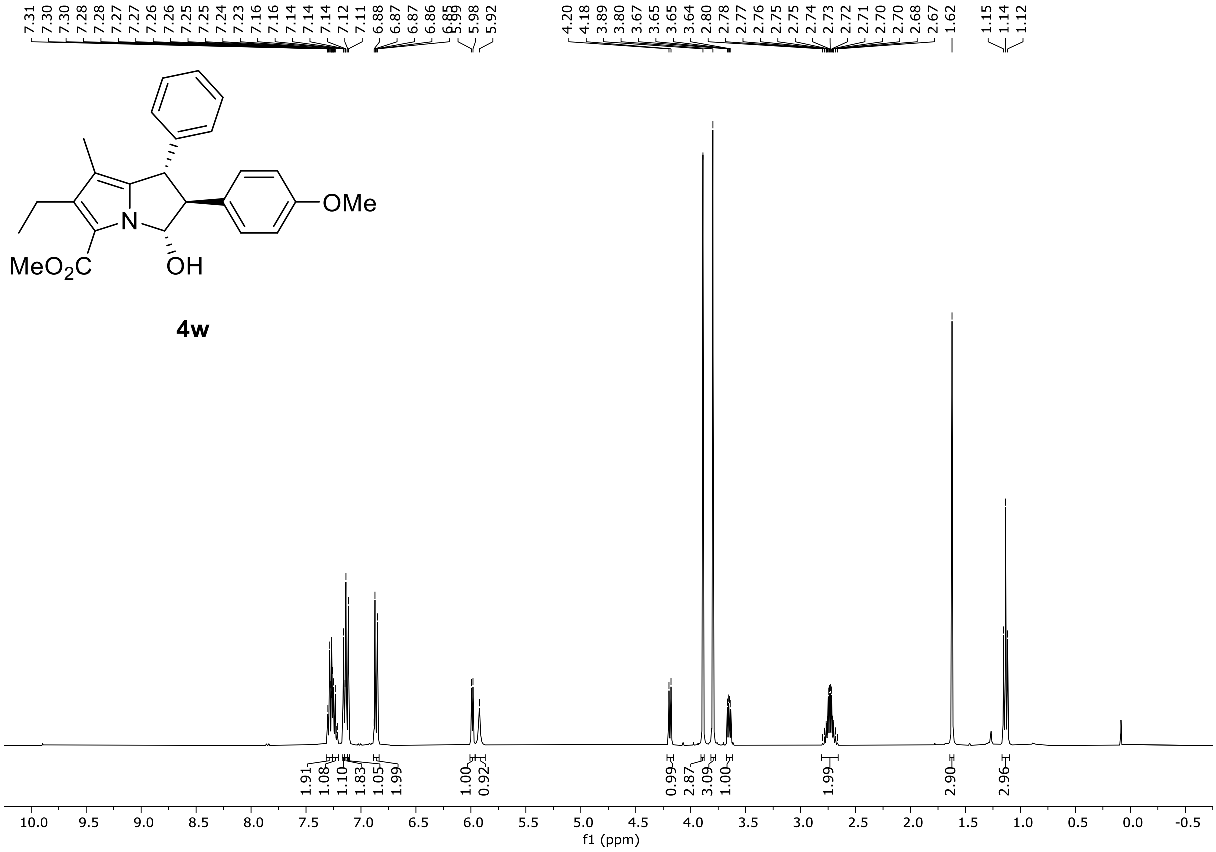

4w

^{1}H-NMR (400 MHz, $CDCl_3$)

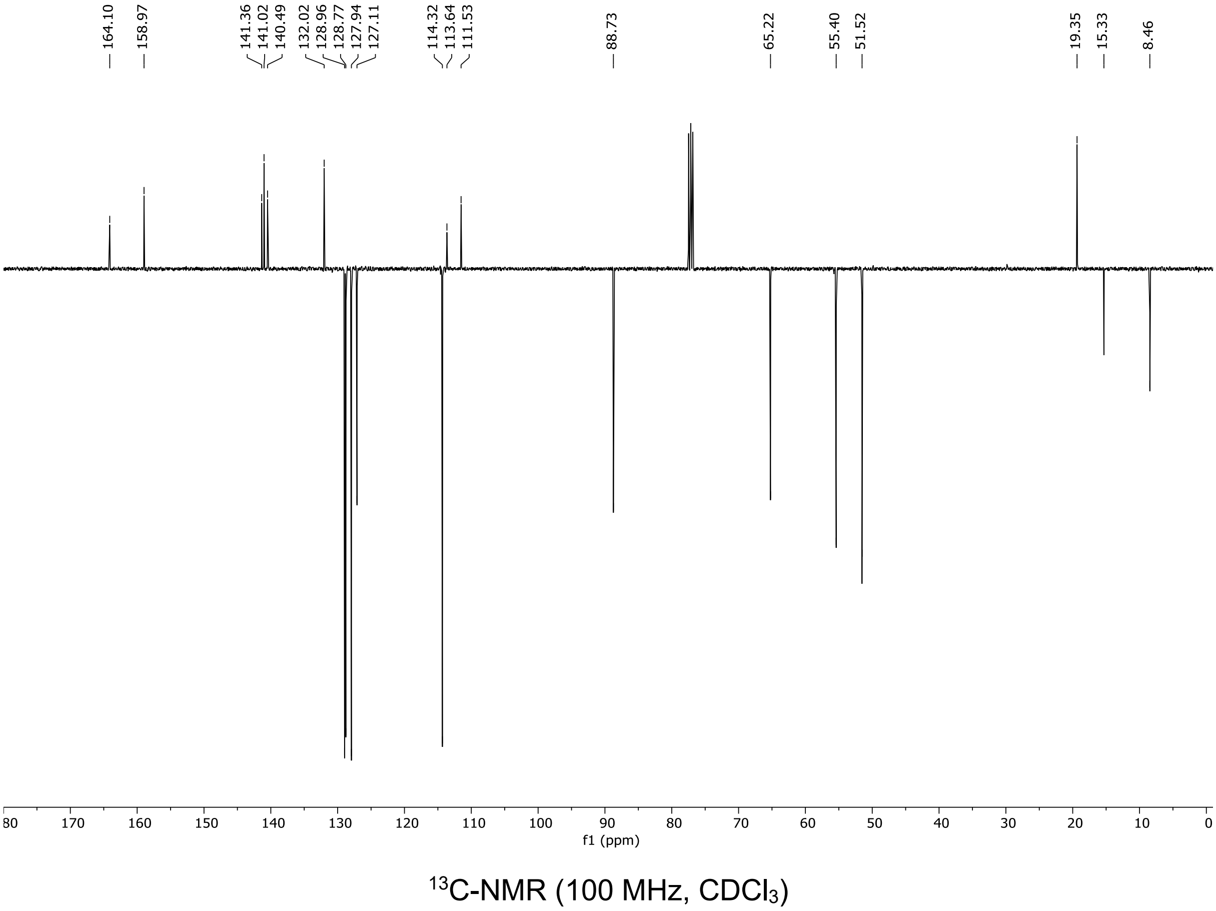

^{13}C-NMR (100 MHz, $CDCl_3$)

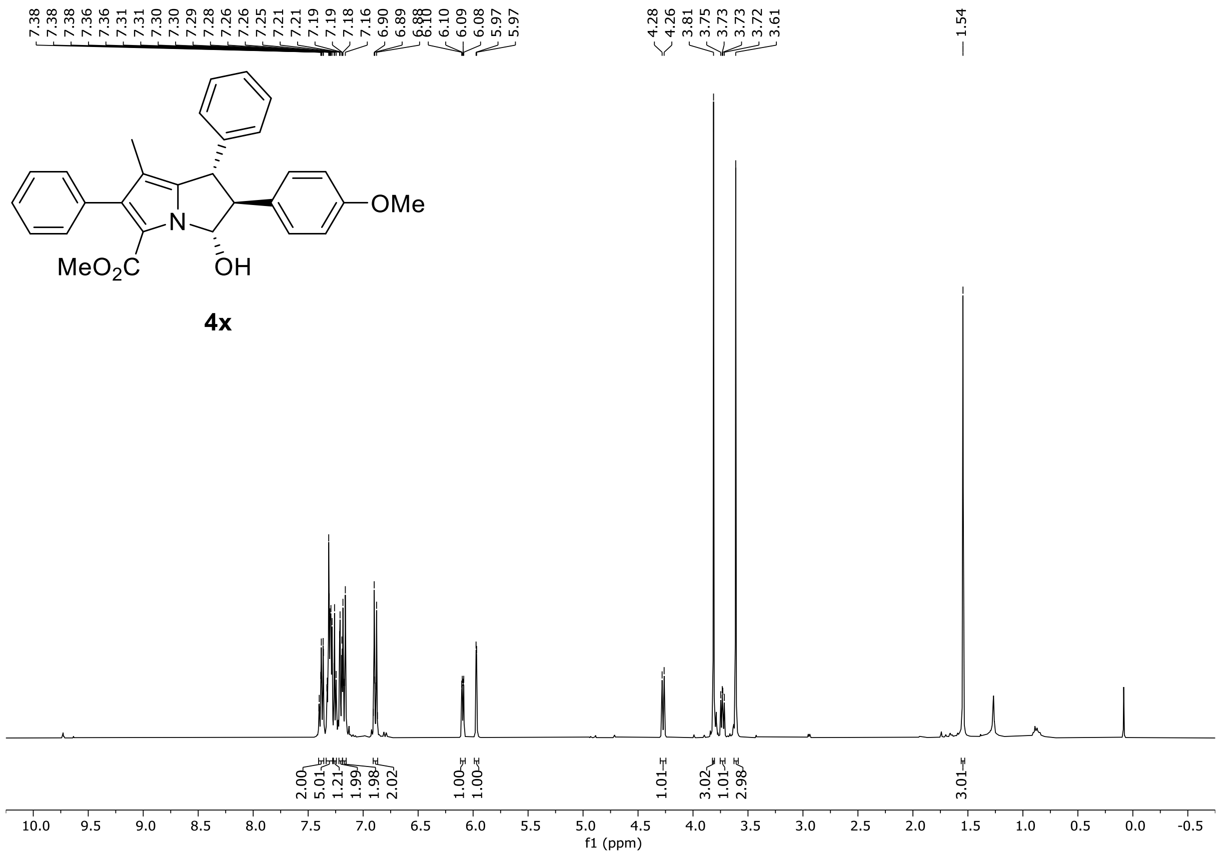

^{1}H-NMR (400 MHz, $CDCl_3$)

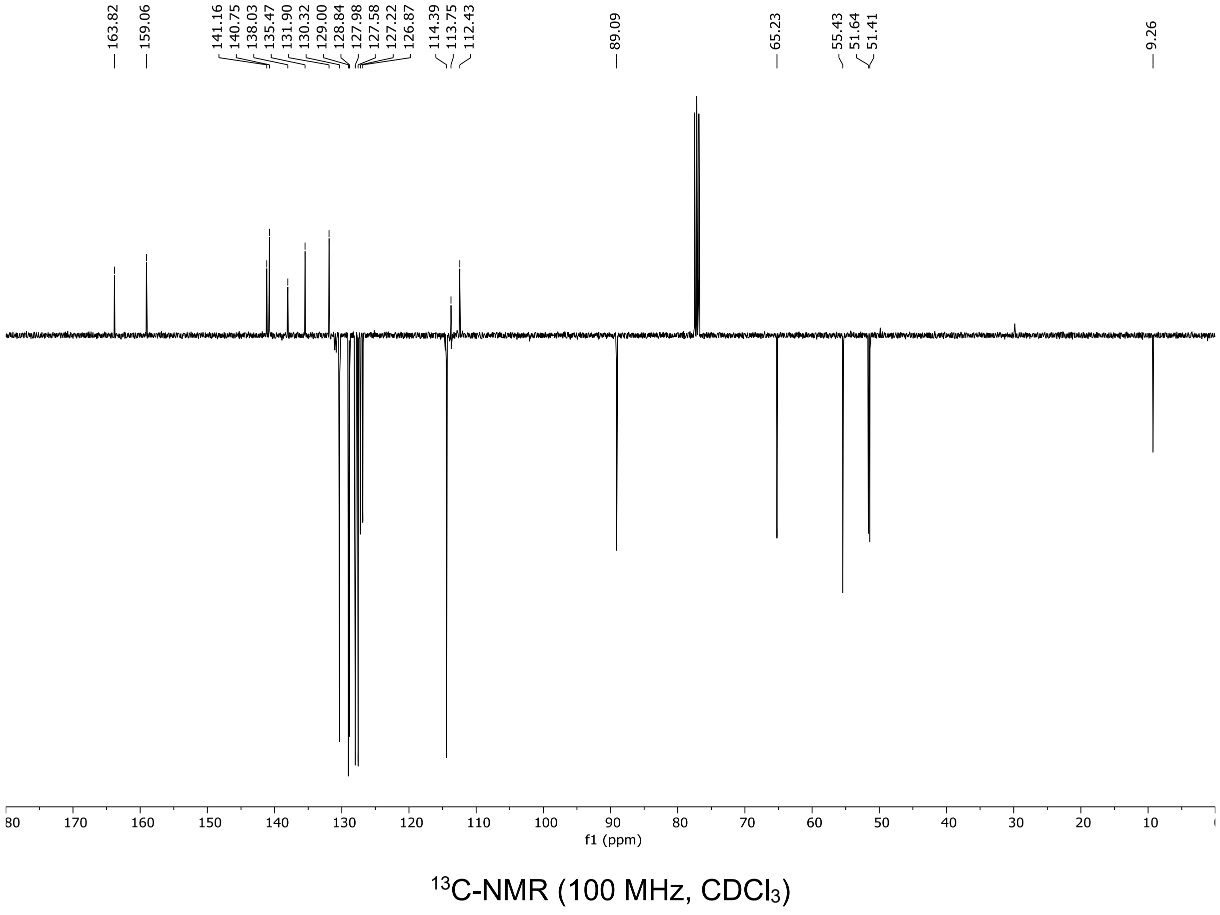

^{13}C-NMR (100 MHz, $CDCl_3$)

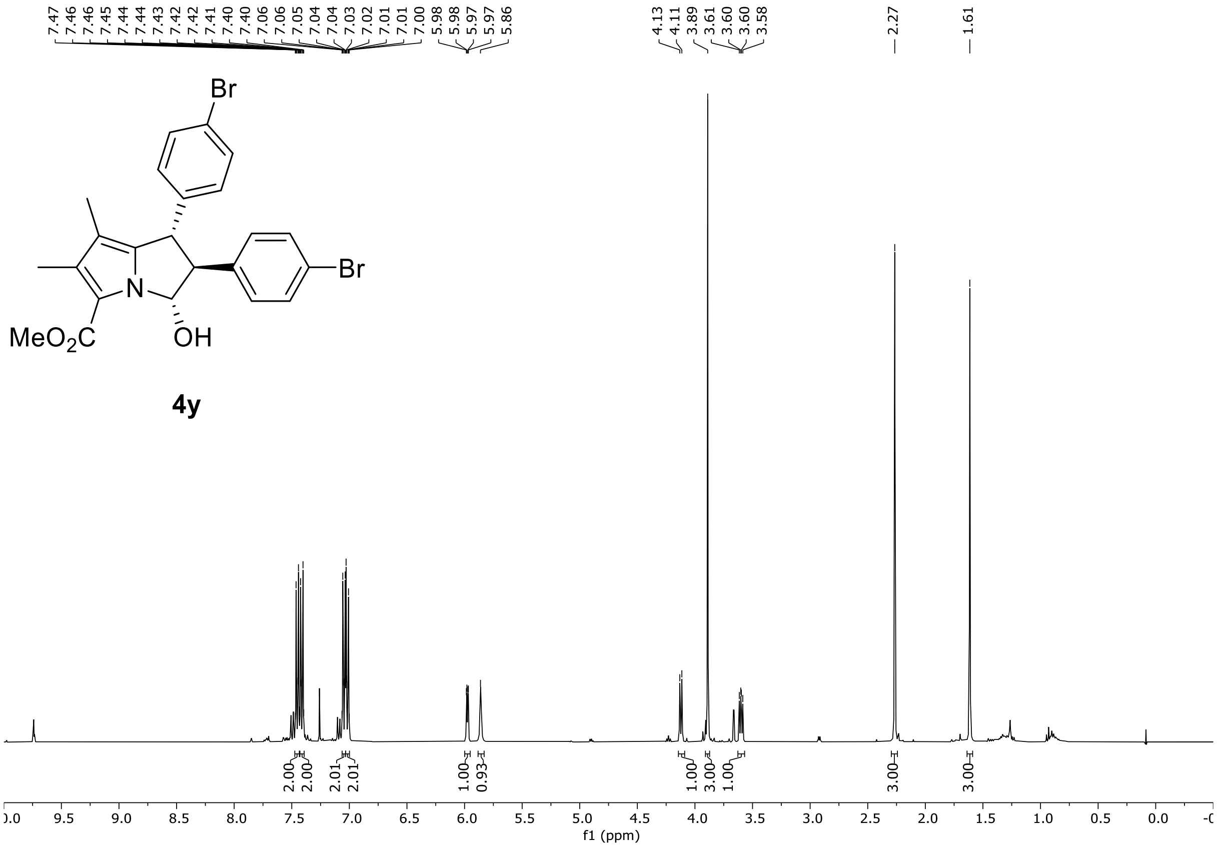

^{1}H-NMR (400 MHz, CDCl$_3$)

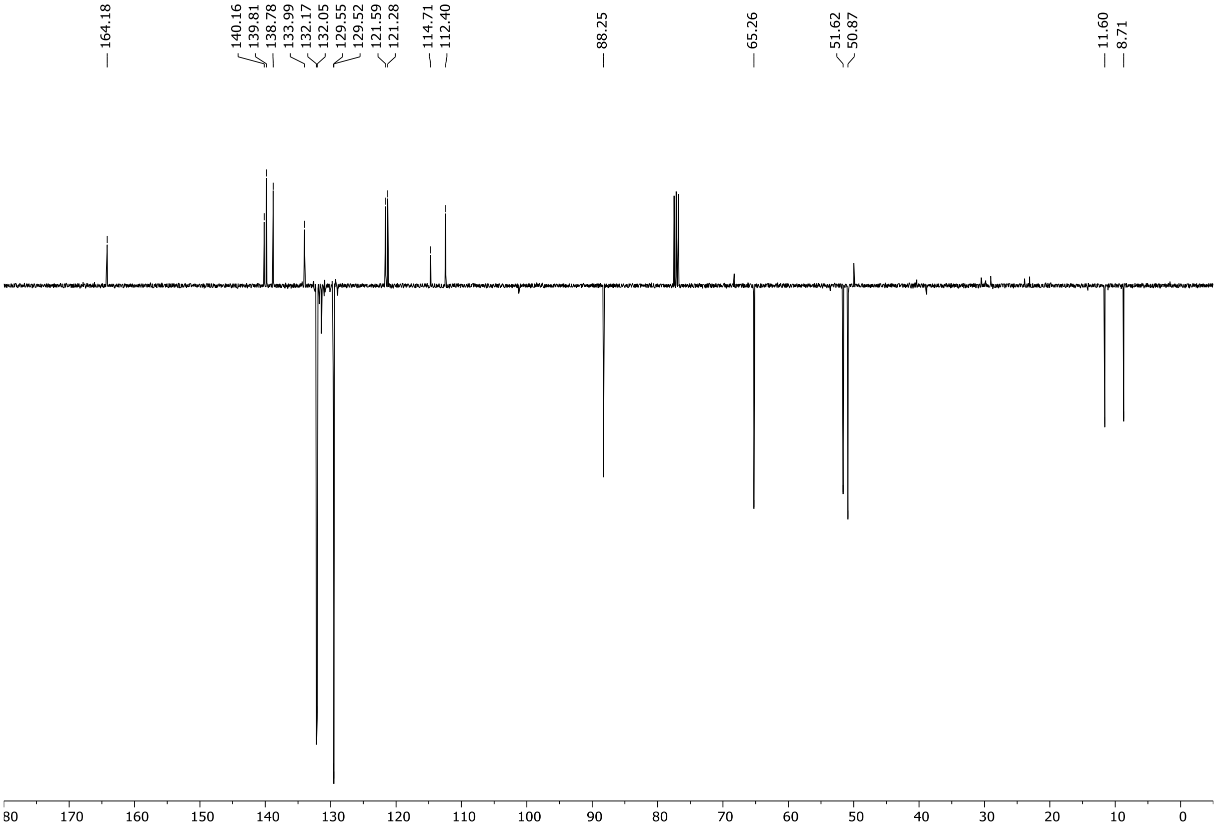

^{13}C-NMR (100 MHz, CDCl$_3$)

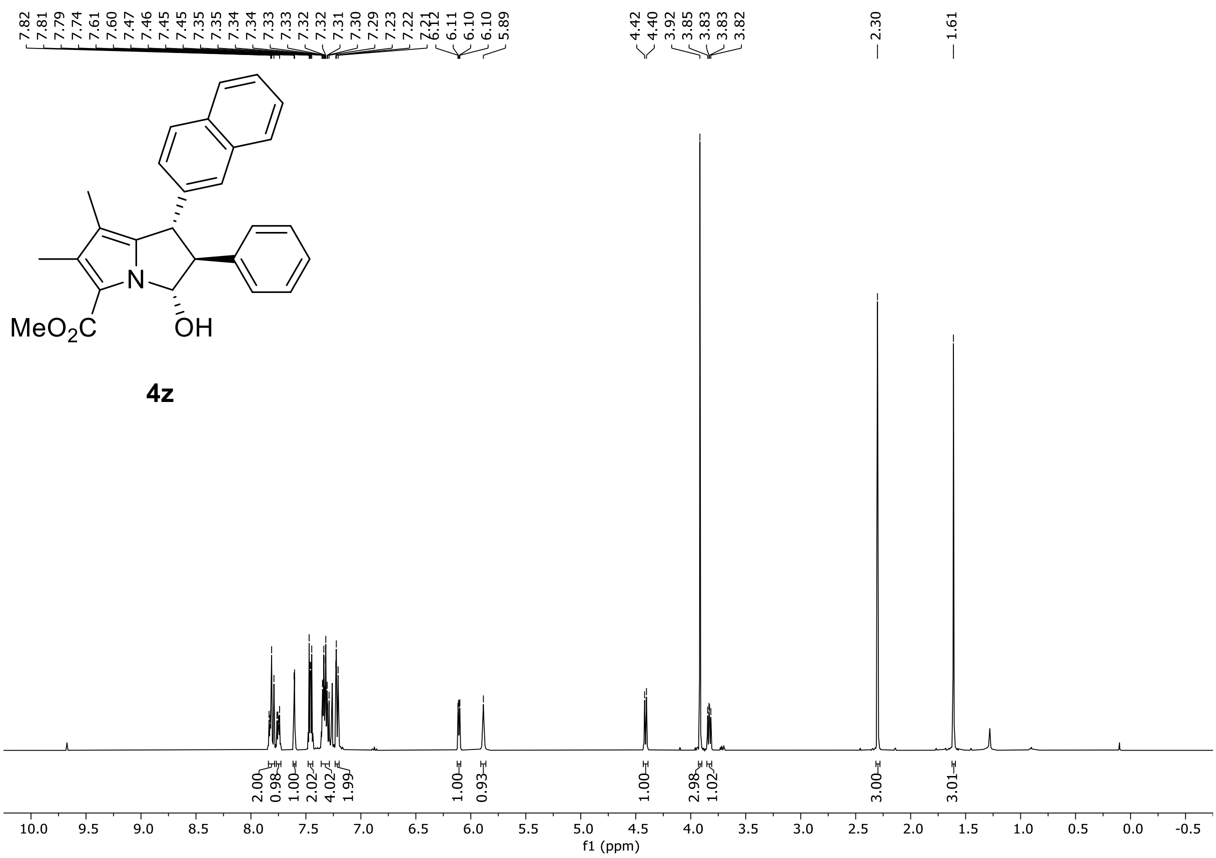

^{1}H-NMR (400 MHz, $CDCl_3$)

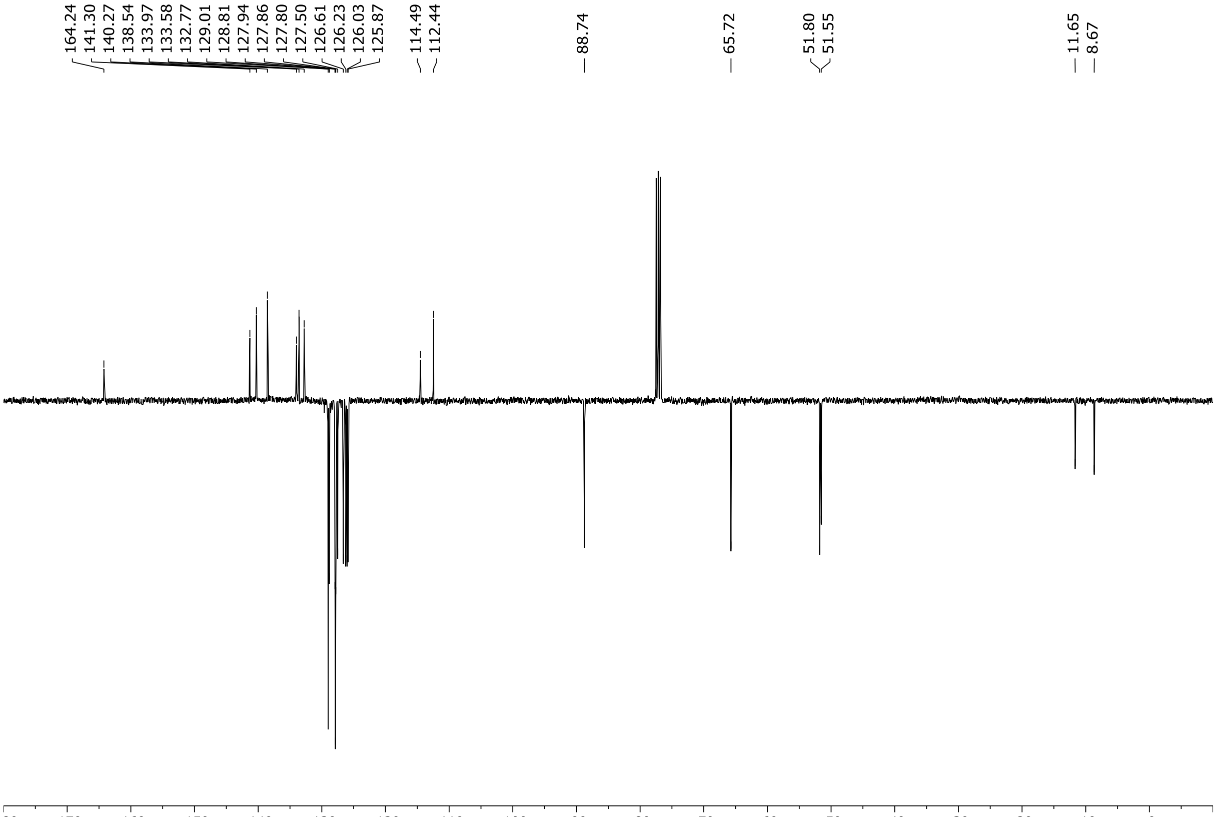

^{13}C-NMR (100 MHz, $CDCl_3$)

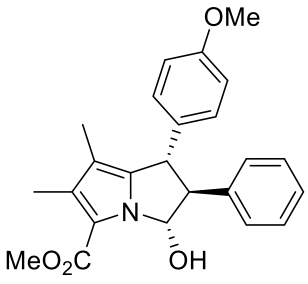

4aa

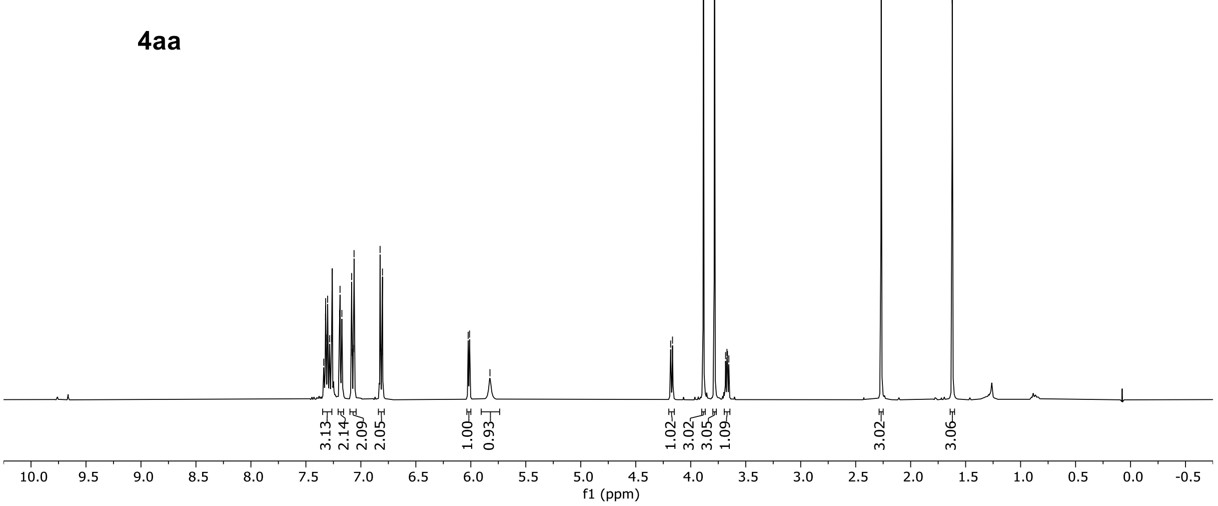

^{1}H-NMR (400 MHz, CDCl$_3$)

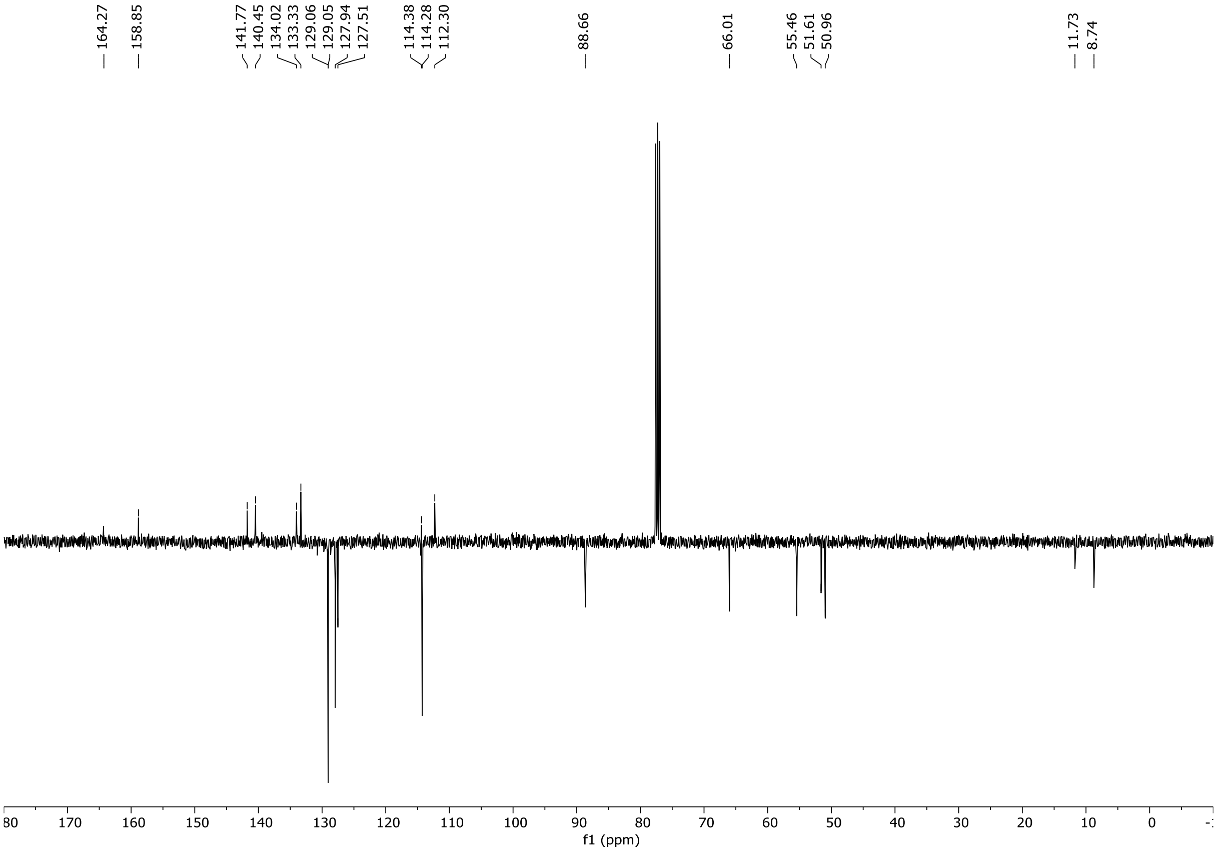

^{13}C-NMR (100 MHz, CDCl$_3$)

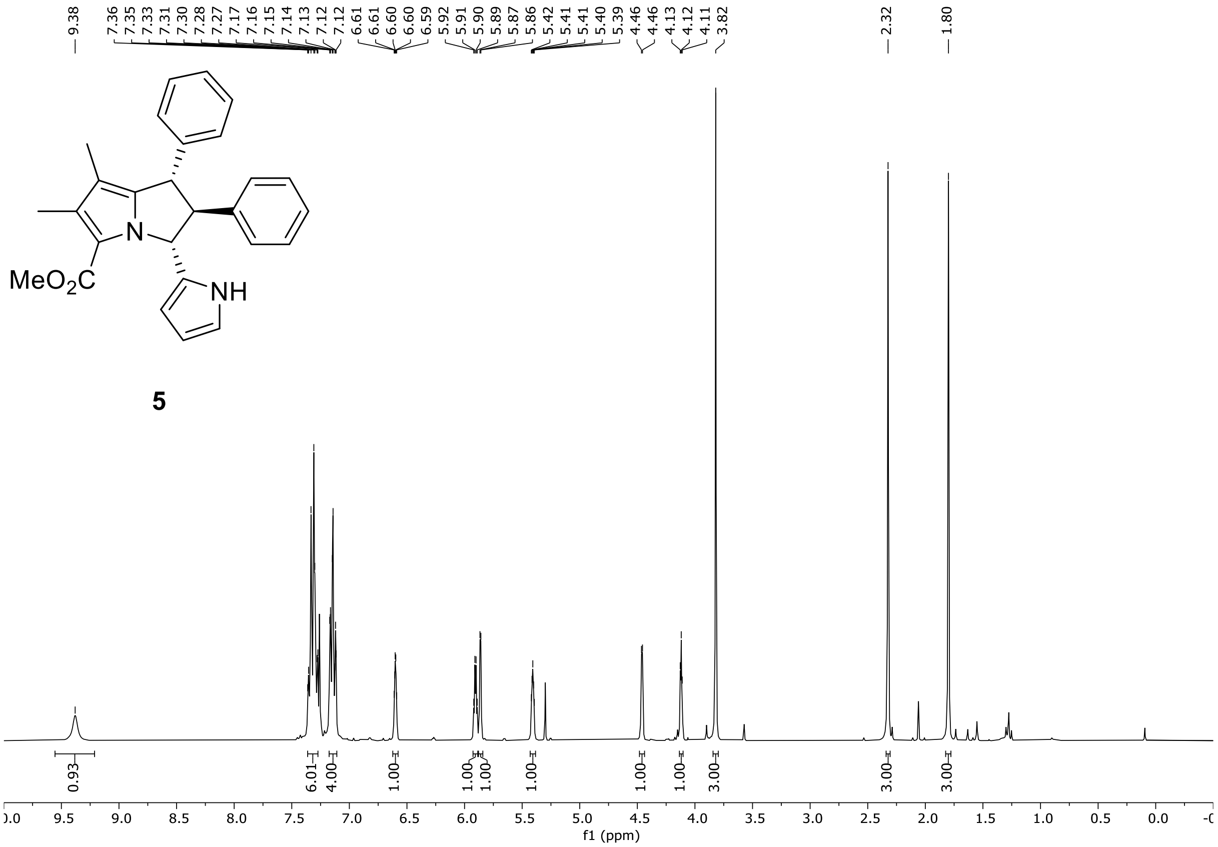

^{1}H-NMR (300 MHz, $CDCl_3$)

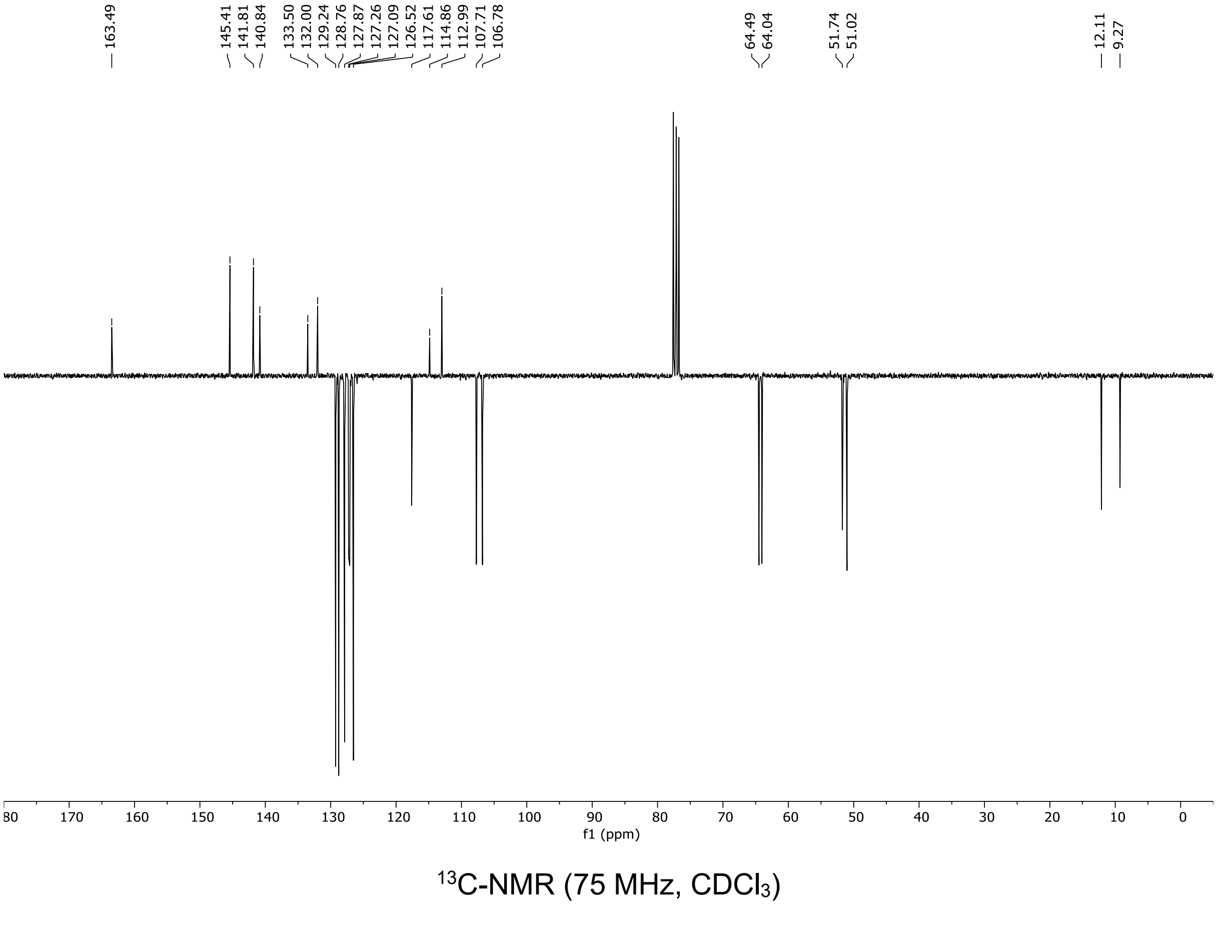

^{13}C-NMR (75 MHz, $CDCl_3$)

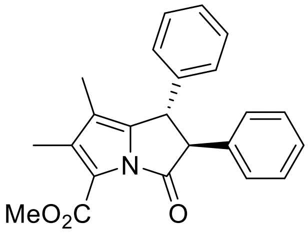

6

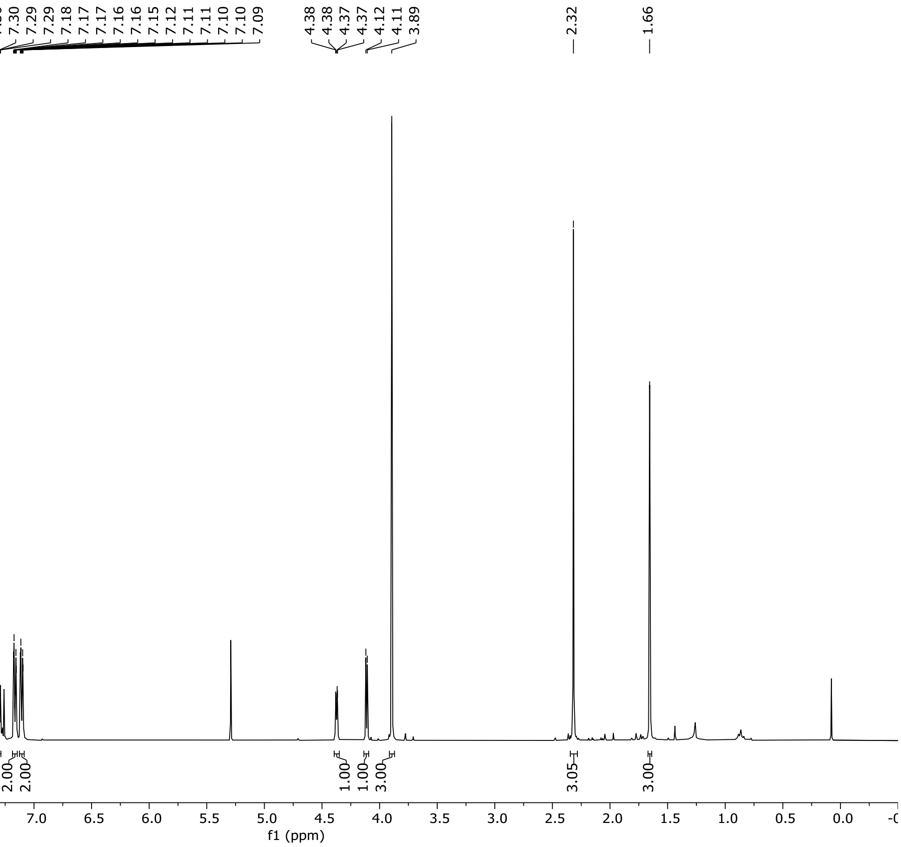

^{1}H-NMR (400 MHz, $CDCl_3$)

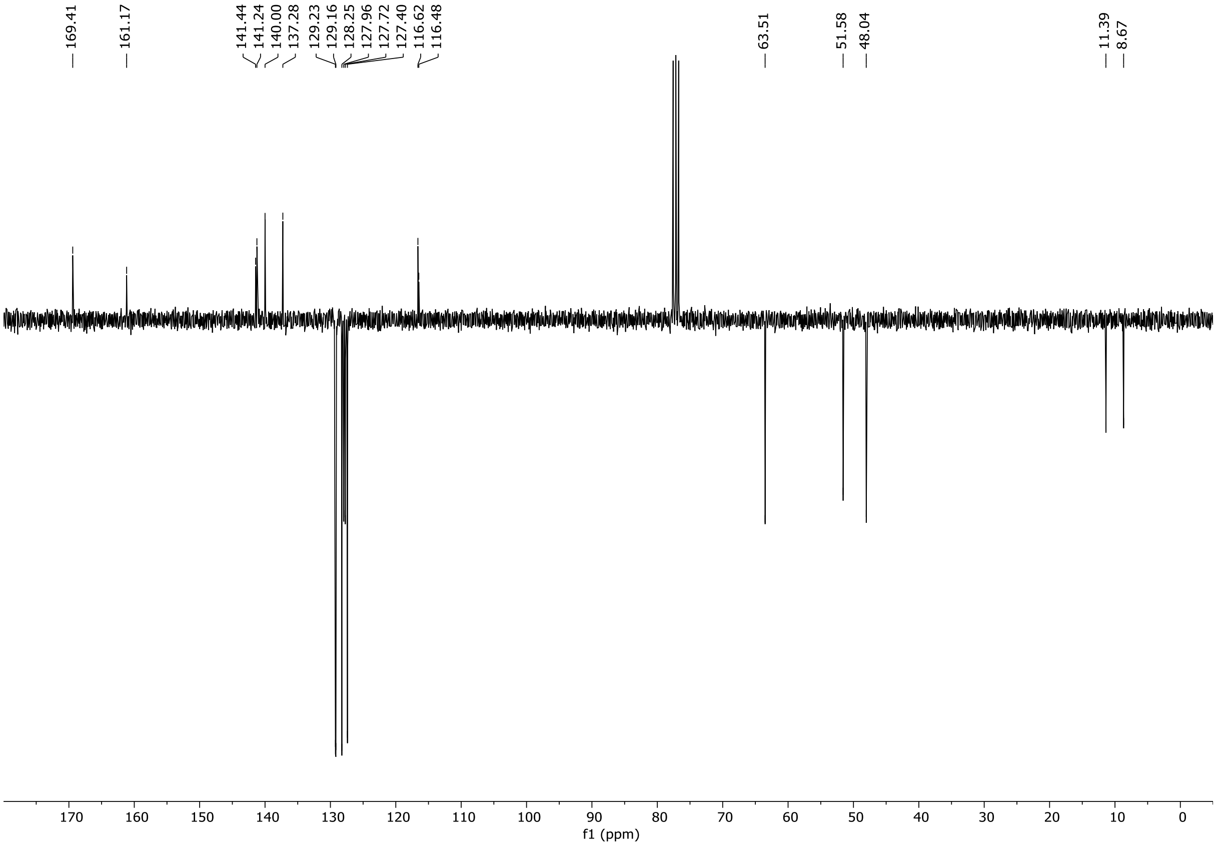

^{13}C-NMR (75 MHz, $CDCl_3$)

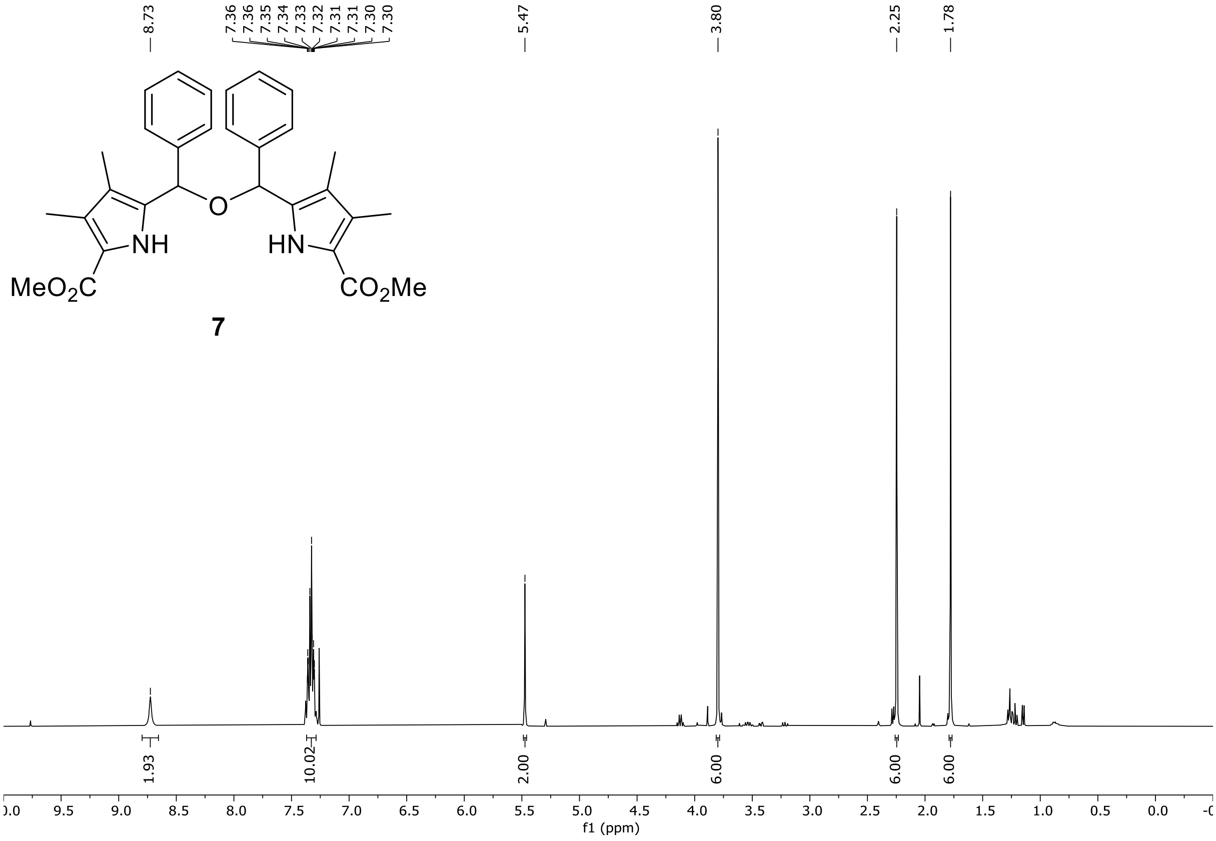

^{1}H-NMR (400 MHz, $CDCl_3$)

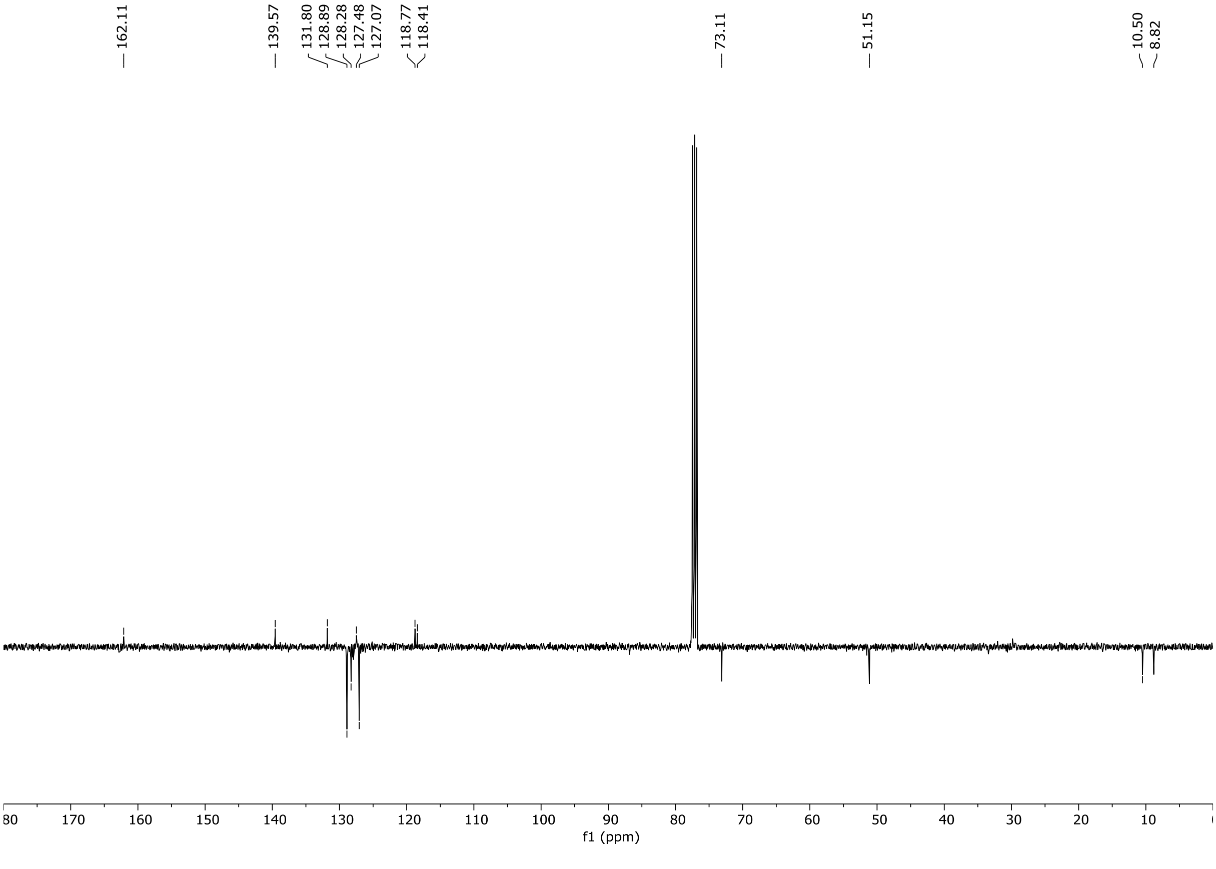

^{13}C-NMR (100 MHz, $CDCl_3$)

3. HPLC Chromatograms

Methyl (1*S*,2*S*,3*S*)-3-hydroxy-6,7-dimethyl-1,2-diphenyl-2,3-dihydro-1*H*-pyrrolizine-5-carboxylate (4a)

IA-column: Hex/ *i*PrOH 90:10 $^{v}/_{v}$, 1.0 ml/ min, λ = 284 nm

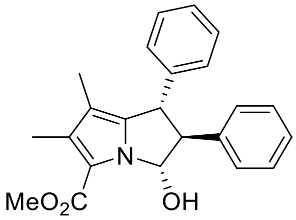

4a

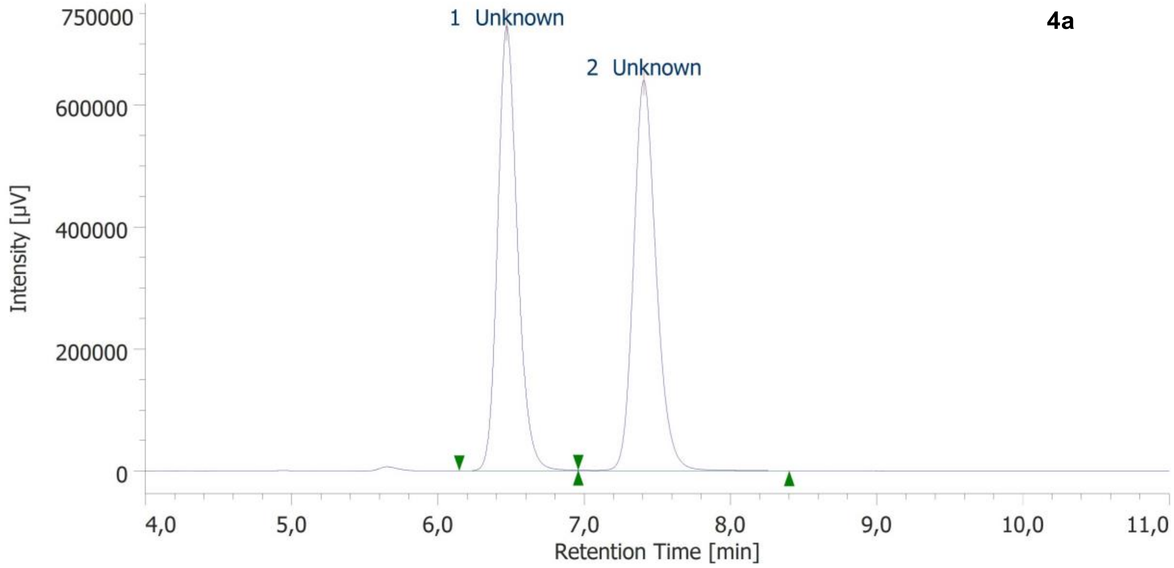

#	Peak Name	CH	tR [min]	Area [µV·sec]	Height [µV]	Area%	Height%	Quantity	NTP	Resolution	Symmetry Factor	Warning
1	Unknown	11	6,468	6861651	729096	49,869	53,249	N/A	11564	3,649	1,199	
2	Unknown	11	7,407	6897786	640129	50,131	46,751	N/A	11604	N/A	1,200	

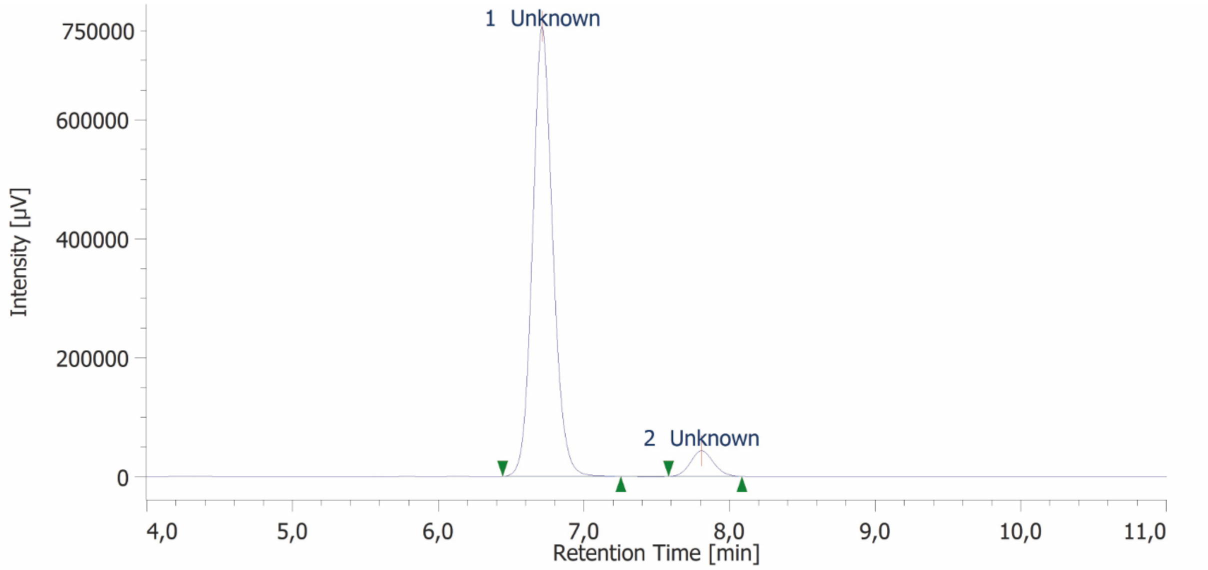

#	Peak Name	CH	tR [min]	Area [µV·sec]	Height [µV]	Area%	Height%	Quantity	NTP	Resolution	Symmetry Factor	Warning
1	Unknown	11	6,713	7285286	755356	93,841	94,606	N/A	11867	4,074	1,103	
2	Unknown	11	7,807	478159	43064	6,159	5,394	N/A	11463	N/A	1,086	

Methyl (1*S*,2*S*,3*S*)-3-hydroxy-6,7-dimethyl-1-phenyl-2-(*o*-tolyl)-2,3-dihydro-1*H*-pyrrolizine-5-carboxylate (4b)

IA-column: Hex/ *i*PrOH 90:10 $^{v}/_{v}$, 1.0 ml/ min, λ = 284 nm

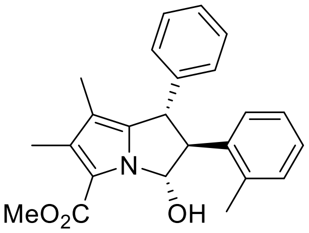

4b

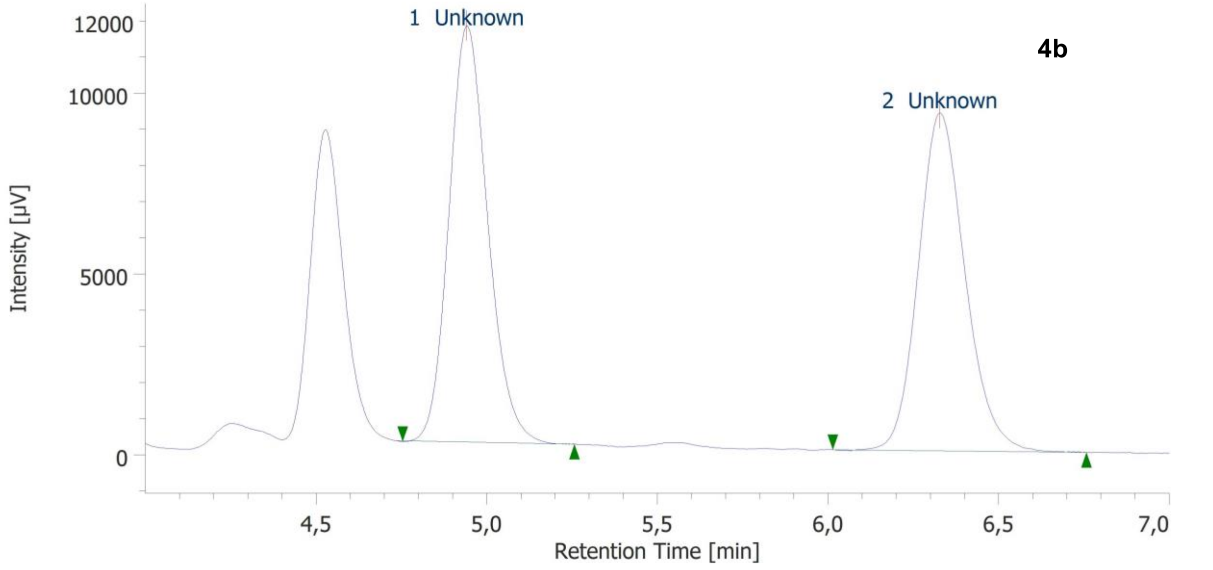

#	Peak Name	CH	tR [min]	Area [µV·sec]	Height [µV]	Area%	Height%	Quantity	NTP	Resolution	Symmetry Factor	Warning
1	Unknown	11	4,940	90949	11504	50,586	55,206	N/A	9209	6,157	1,179	
2	Unknown	11	6,327	88842	9335	49,414	44,794	N/A	10608	N/A	1,173	

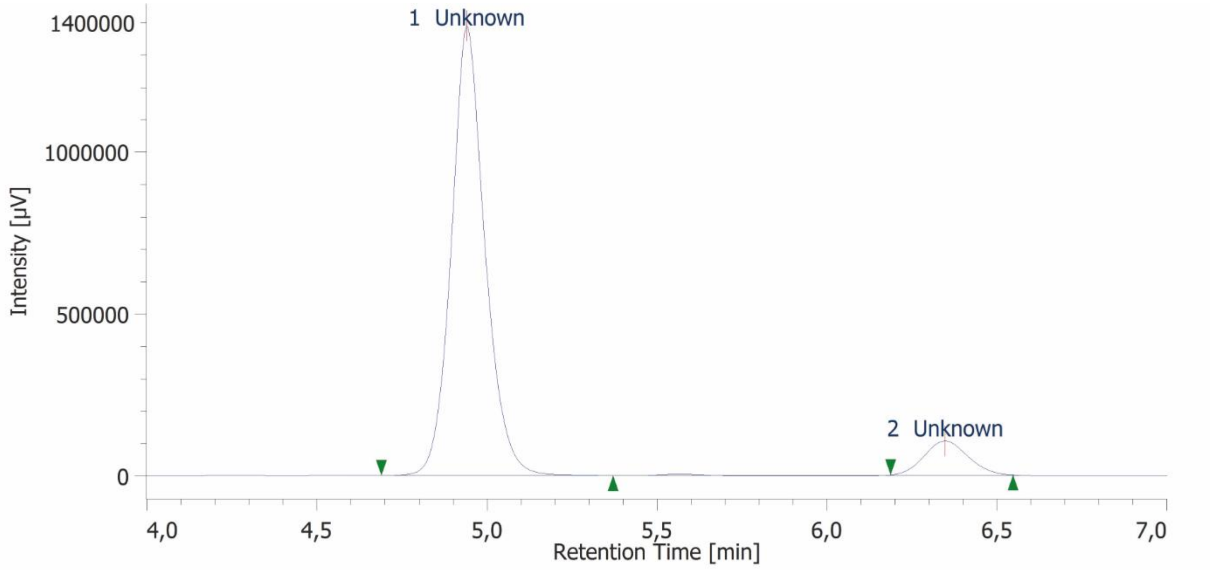

#	Peak Name	CH	tR [min]	Area [µV·sec]	Height [µV]	Area%	Height%	Quantity	NTP	Resolution	Symmetry Factor	Warning
1	Unknown	11	4,940	9536977	1387111	90,725	92,858	N/A	13173	6,837	1,139	
2	Unknown	11	6,347	975039	106691	9,275	7,142	N/A	11148	N/A	1,105	

Methyl (1*S*,2*S*,3*S*)-3-hydroxy-6,7-dimethyl-1-phenyl-2-(*m*-tolyl)-2,3-dihydro-1*H*-pyrrolizine-5-carboxylate (4c)

IA-column: Hex/ *i*PrOH 98:2 $^v/_v$, 1.0 ml/ min, λ = 284 nm

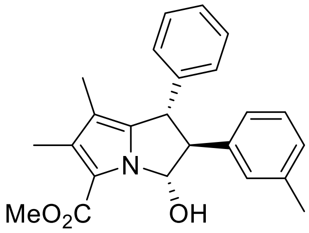

4c

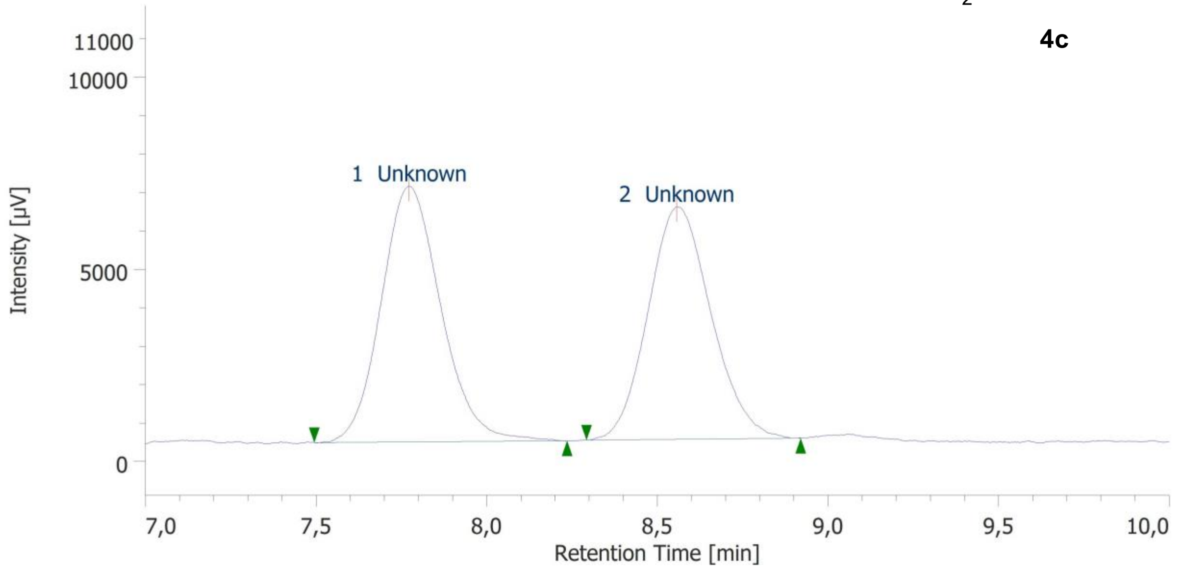

#	Peak Name	CH	tR [min]	Area [µV·sec]	Height [µV]	Area%	Height%	Quantity	NTP	Resolution	Symmetry Factor	Warning
1	Unknown	11	7,772	80803	6651	51,041	52,364	N/A	9739	2,417	1,141	
2	Unknown	11	8,557	77506	6050	48,959	47,636	N/A	10352	N/A	1,145	

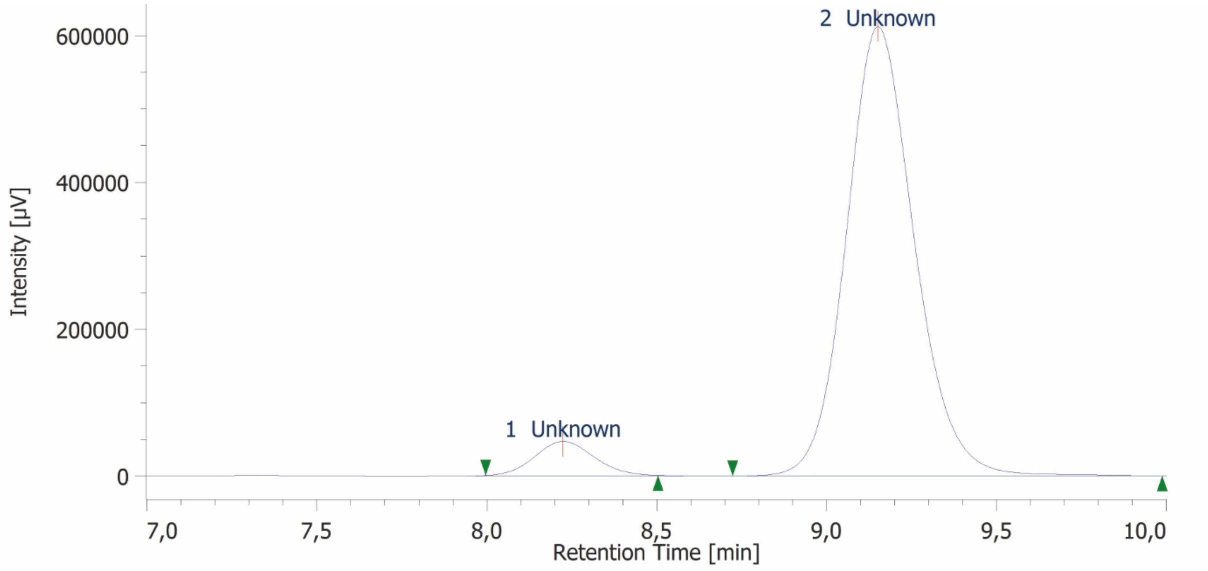

#	Peak Name	CH	tR [min]	Area [µV·sec]	Height [µV]	Area%	Height%	Quantity	NTP	Resolution	Symmetry Factor	Warning
1	Unknown	11	8,223	578489	47022	6,361	7,138	N/A	10437	2,736	1,100	
2	Unknown	11	9,150	8515348	611691	93,639	92,862	N/A	10491	N/A	1,136	

Methyl (1*S*,2*S*,3*S*)-3-hydroxy-6,7-dimethyl-1-phenyl-2-(*p*-tolyl)-2,3-dihydro-1*H*-pyrrolizine-5-carboxylate (4d)

IA-column: Hex/ *i*PrOH 90:10 v/v, 1.0 ml/ min, λ = 284 nm

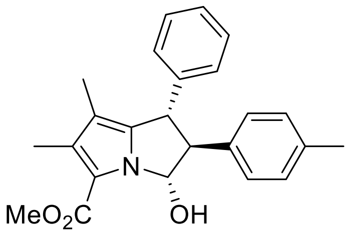

4d

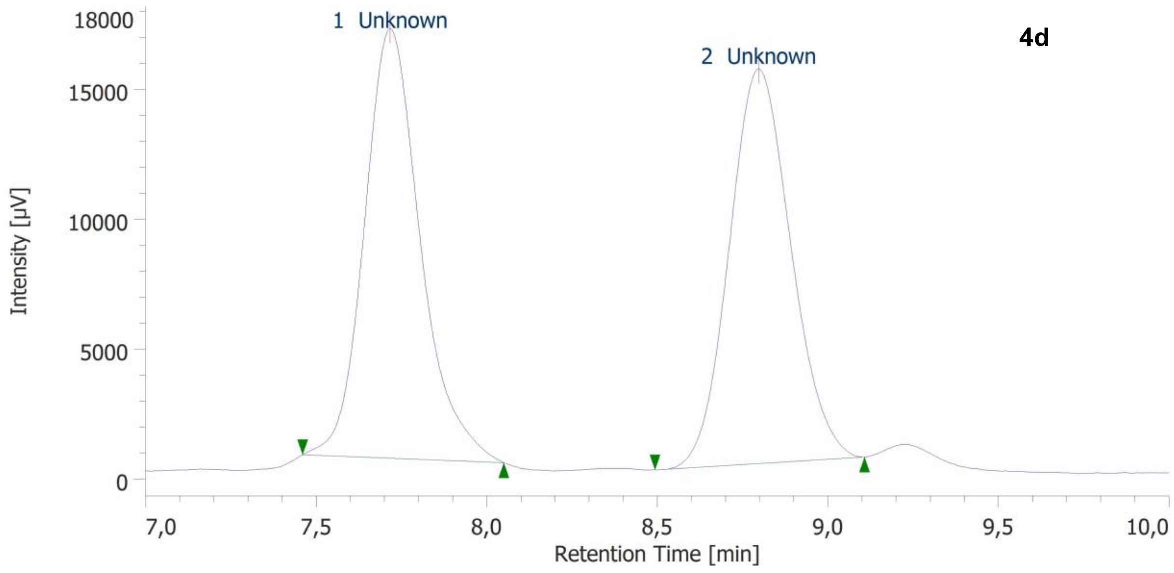

#	Peak Name	CH	tR [min]	Area [μV·sec]	Height [μV]	Area%	Height%	Quantity	NTP	Resolution	Symmetry Factor	Warning
1	Unknown	11	7,717	188403	16529	49,725	52,127	N/A	11452	3,491	1,219	
2	Unknown	11	8,797	190489	15180	50,275	47,873	N/A	11235	N/A	1,099	

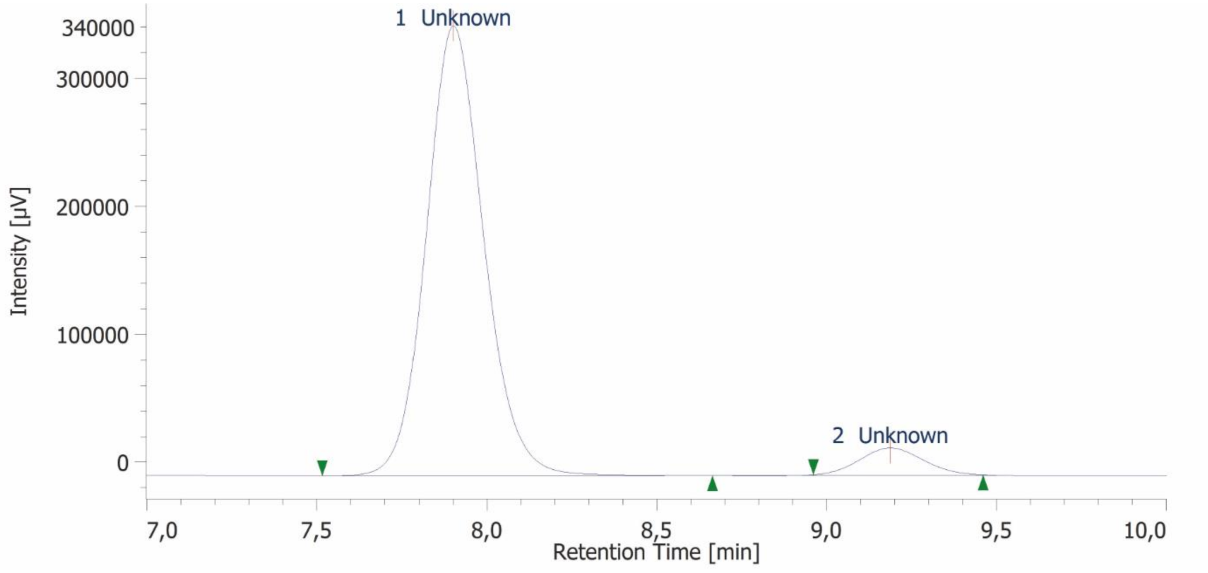

#	Peak Name	CH	tR [min]	Area [μV·sec]	Height [μV]	Area%	Height%	Quantity	NTP	Resolution	Symmetry Factor	Warning
1	Unknown	11	7,900	4144435	351402	93,490	94,221	N/A	10886	3,935	1,127	
2	Unknown	11	9,187	288582	21551	6,510	5,779	N/A	10846	N/A	1,078	

Methyl (1*S*,2*S*,3*S*)-2-(2-fluorophenyl)-3-hydroxy-6,7-dimethyl-1-phenyl-2,3-dihydro-1*H*-pyrrolizine-5-carboxylate (4e)

IA-column: Hex/ *i*PrOH 90:10 $^{v}/_{v}$, 1.0 ml/ min, λ = 284 nm

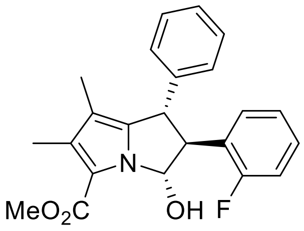

4e

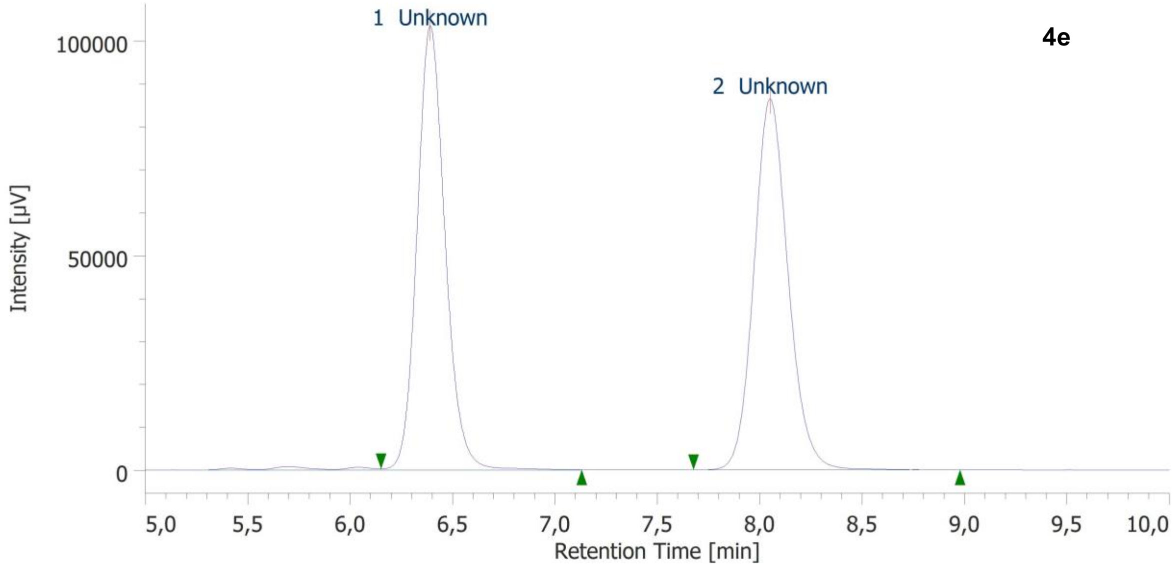

#	Peak Name	CH	tR [min]	Area [µV·sec]	Height [µV]	Area%	Height%	Quantity	NTP	Resolution	Symmetry Factor	Warning
1	Unknown	11	6,390	985439	103428	49,556	54,475	N/A	10967	6,131	1,144	
2	Unknown	11	8,050	1003111	86435	50,444	45,525	N/A	11603	N/A	1,124	

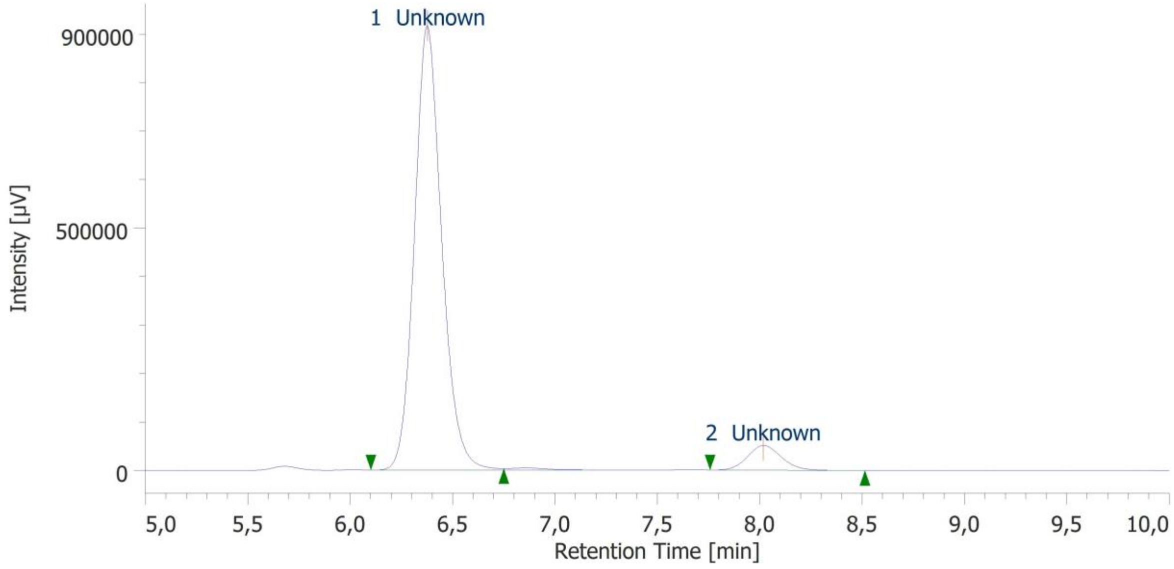

#	Peak Name	CH	tR [min]	Area [µV·sec]	Height [µV]	Area%	Height%	Quantity	NTP	Resolution	Symmetry Factor	Warning
1	Unknown	11	6,377	8466540	915696	93,624	94,745	N/A	11703	6,170	1,139	
2	Unknown	11	8,017	576585	50791	6,376	5,255	N/A	11636	N/A	1,124	

Methyl (1*S*,2*S*,3*S*)-2-(4-bromophenyl)-3-hydroxy-6,7-dimethyl-1-phenyl-2,3-dihydro-1*H*-pyrrolizine-5-carboxylate (4f)

IE-column: Hex/ *i*PrOH 90:10 v/v, 1.0 ml/ min, λ = 284 nm

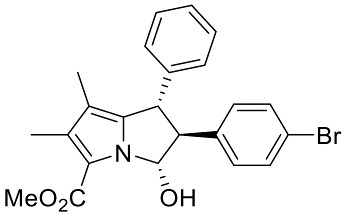

4f

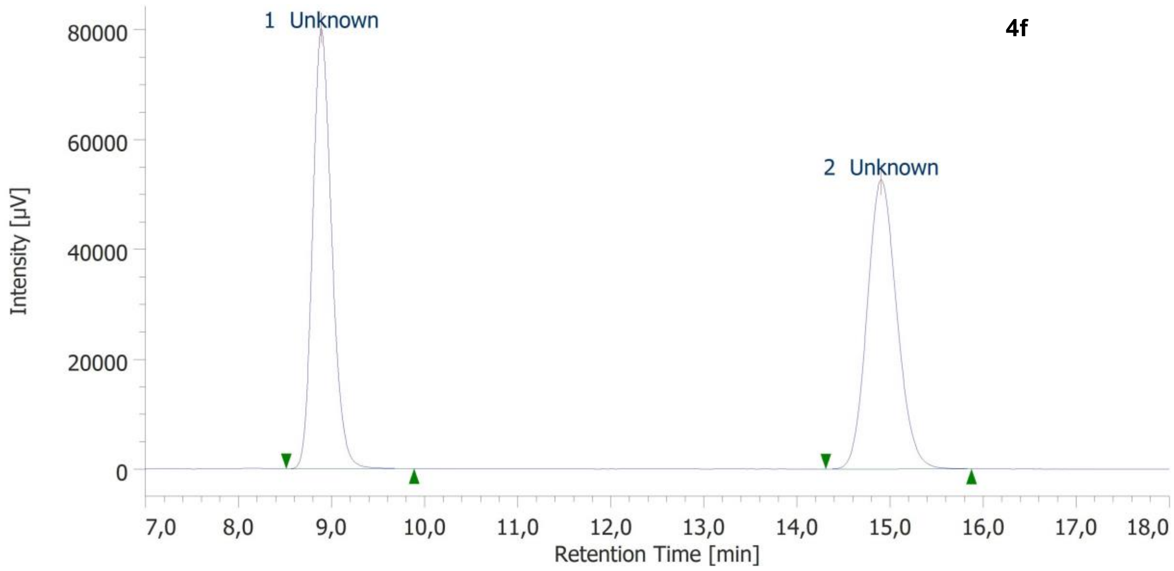

#	Peak Name	CH	tR [min]	Area [µV·sec]	Height [µV]	Area%	Height%	Quantity	NTP	Resolution	Symmetry Factor	Warning
1	Unknown	11	8,890	1138588	80131	49,369	60,375	N/A	9331	12,683	1,190	
2	Unknown	11	14,903	1167675	52590	50,631	39,625	N/A	10470	N/A	1,120	

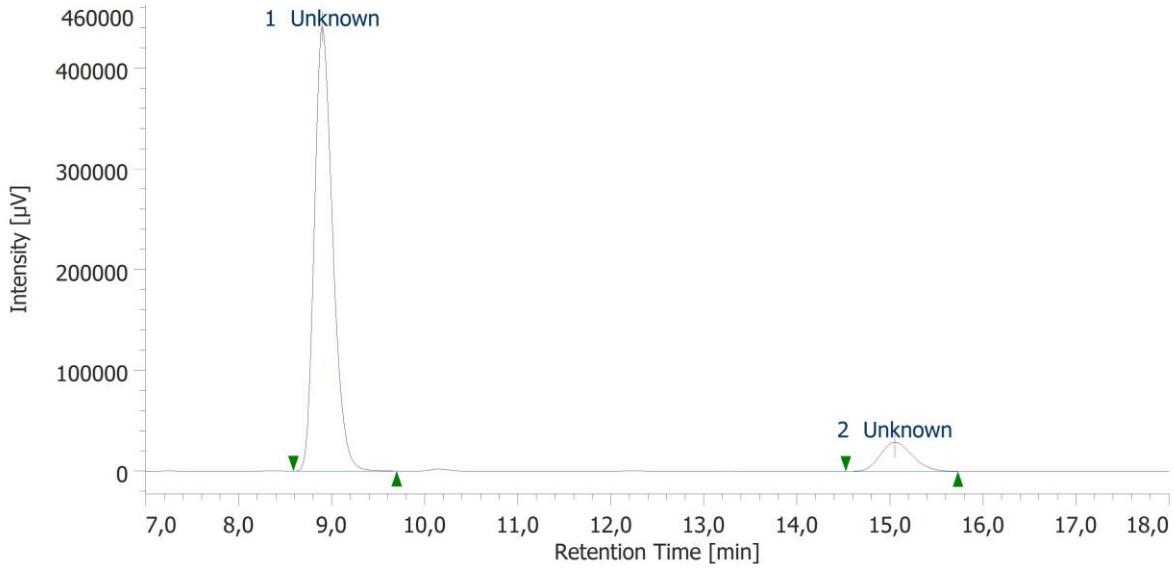

#	Peak Name	CH	tR [min]	Area [µV·sec]	Height [µV]	Area%	Height%	Quantity	NTP	Resolution	Symmetry Factor	Warning
1	Unknown	11	8,897	6042062	441193	89,732	93,850	N/A	9992	12,514	1,285	
2	Unknown	11	15,050	691404	28909	10,268	6,150	N/A	9130	N/A	1,170	

Methyl (1*S*,2*S*,3*S*)-2-(3-bromophenyl)-3-hydroxy-6,7-dimethyl-1-phenyl-2,3-dihydro-1*H*-pyrrolizine-5-carboxylate (4g)

IE-column: Hex/ *i*PrOH 90:10 v/v, 1.0 ml/ min, λ = 284 nm

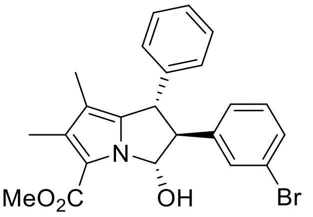

4g

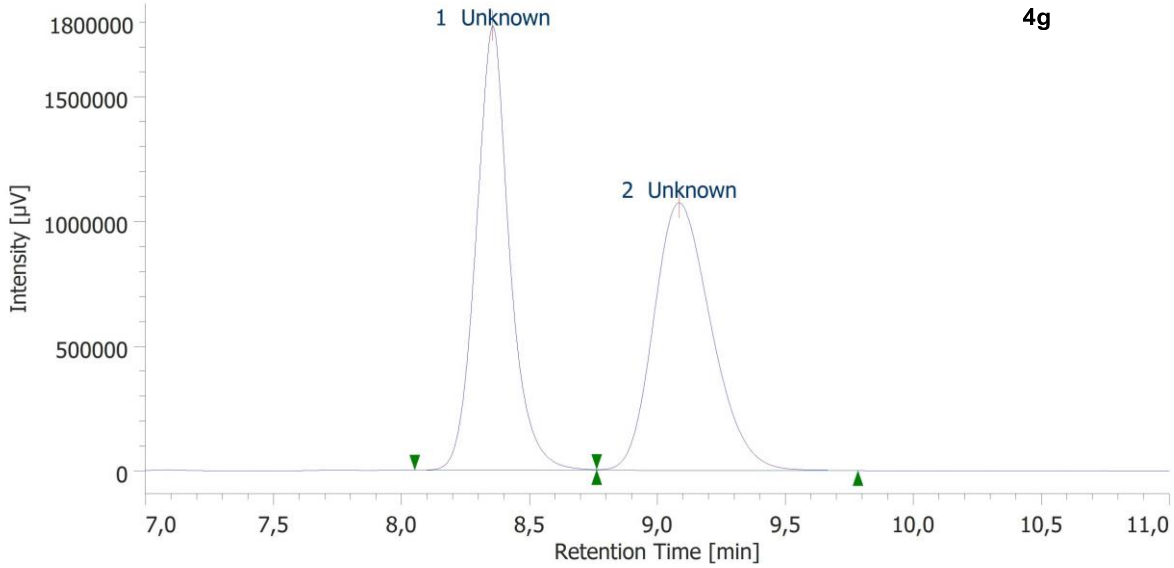

#	Peak Name	CH	tR [min]	Area [µV·sec]	Height [µV]	Area%	Height%	Quantity	NTP	Resolution	Symmetry Factor	Warning
1	Unknown	11	8,357	16232101	1780251	49,191	62,405	N/A	21889	2,291	1,115	
2	Unknown	11	9,085	16766207	1072479	50,809	37,595	N/A	7791	N/A	1,220	

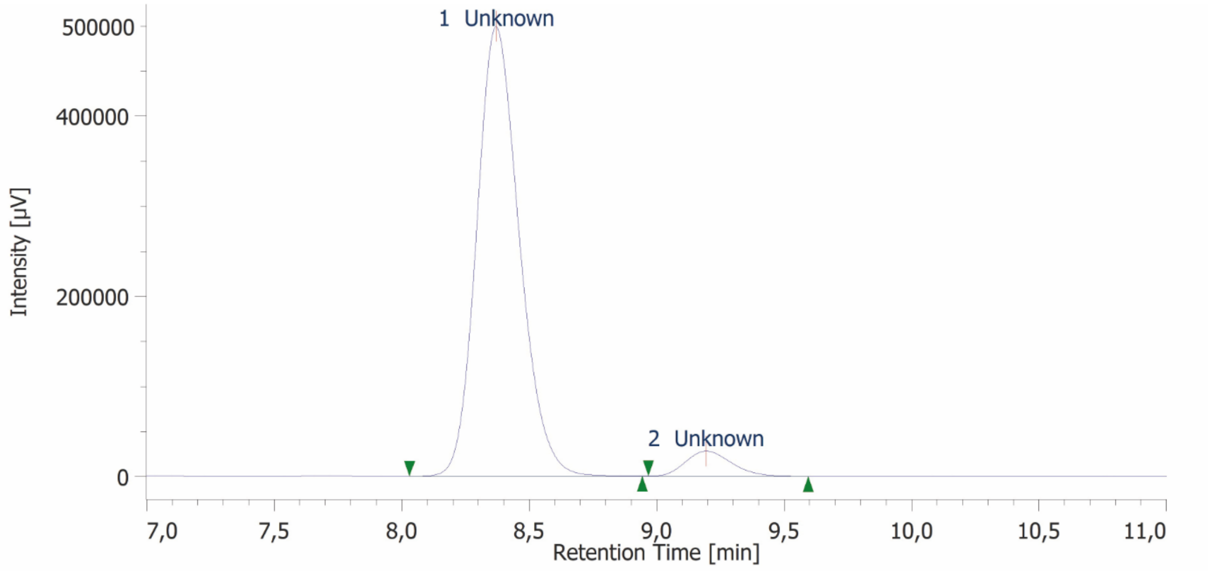

#	Peak Name	CH	tR [min]	Area [µV·sec]	Height [µV]	Area%	Height%	Quantity	NTP	Resolution	Symmetry Factor	Warning
1	Unknown	11	8,370	5814903	499075	94,279	94,688	N/A	12205	2,600	1,176	
2	Unknown	11	9,193	352852	28000	5,721	5,312	N/A	12267	N/A	1,204	

Methyl (1*S*,2*S*,3*S*)-3-hydroxy-2-(3-methoxyphenyl)-6,7-dimethyl-1-phenyl-2,3-dihydro-1*H*-pyrrolizine-5-carboxylate (4h)

IA-column: Hex/ *i*PrOH 98:2 $^{v}/_{v}$, 0.5 ml/ min, λ = 284 nm

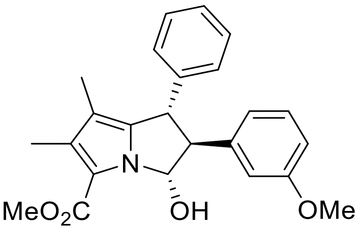

4h

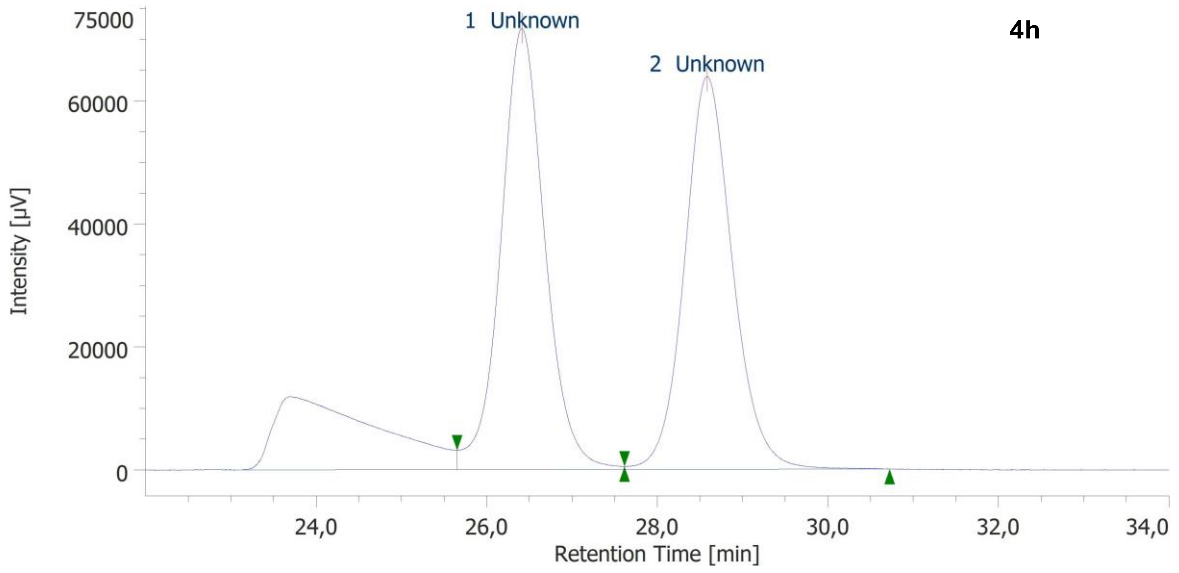

#	Peak Name	CH	tR [min]	Area [µV·sec]	Height [µV]	Area%	Height%	Quantity	NTP	Resolution	Symmetry Factor	Warning
1	Unknown	11	26,412	2570613	71643	50,737	52,894	N/A	13662	2,292	1,022	
2	Unknown	11	28,583	2495901	63804	49,263	47,106	N/A	13174	N/A	1,106	

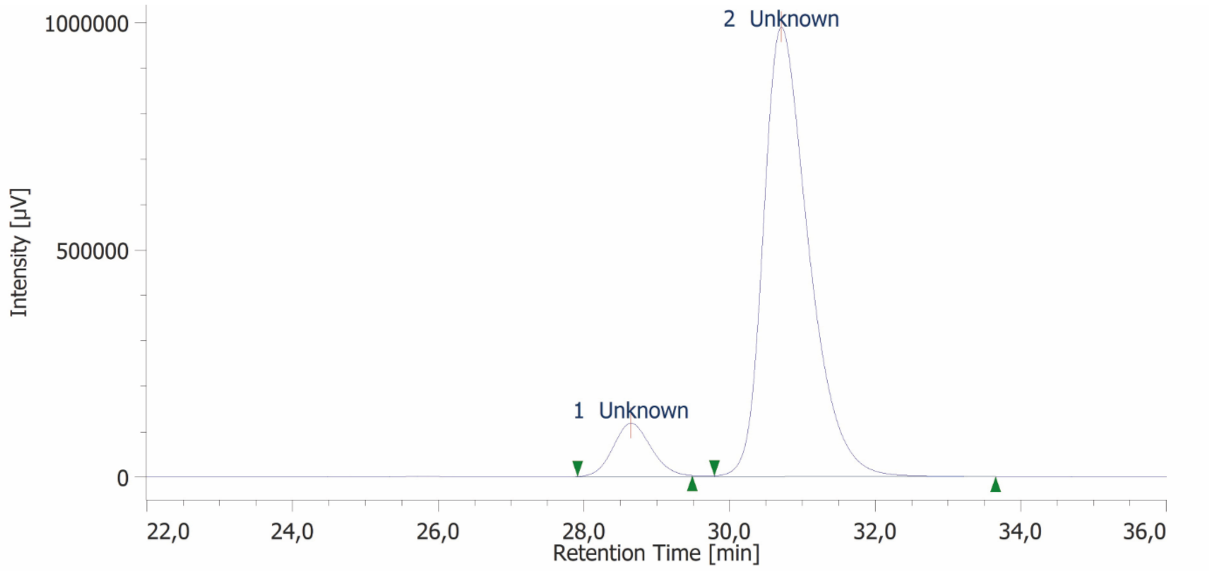

#	Peak Name	CH	tR [min]	Area [µV·sec]	Height [µV]	Area%	Height%	Quantity	NTP	Resolution	Symmetry Factor	Warning
1	Unknown	11	28,643	4326960	118457	9,183	10,693	N/A	14515	2,018	1,133	
2	Unknown	11	30,710	42793938	989379	90,817	89,307	N/A	12401	N/A	1,413	

Methyl (1*S*,2*S*,3*S*)-3-hydroxy-2-(4-methoxyphenyl)-6,7-dimethyl-1-phenyl-2,3-dihydro-1*H*-pyrrolizine-5-carboxylate (4i)

IA-column: Hex/ *i*PrOH 90:10 $^v/_v$, 0.5 ml/ min, λ = 284 nm

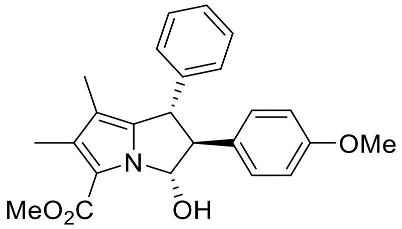

4i

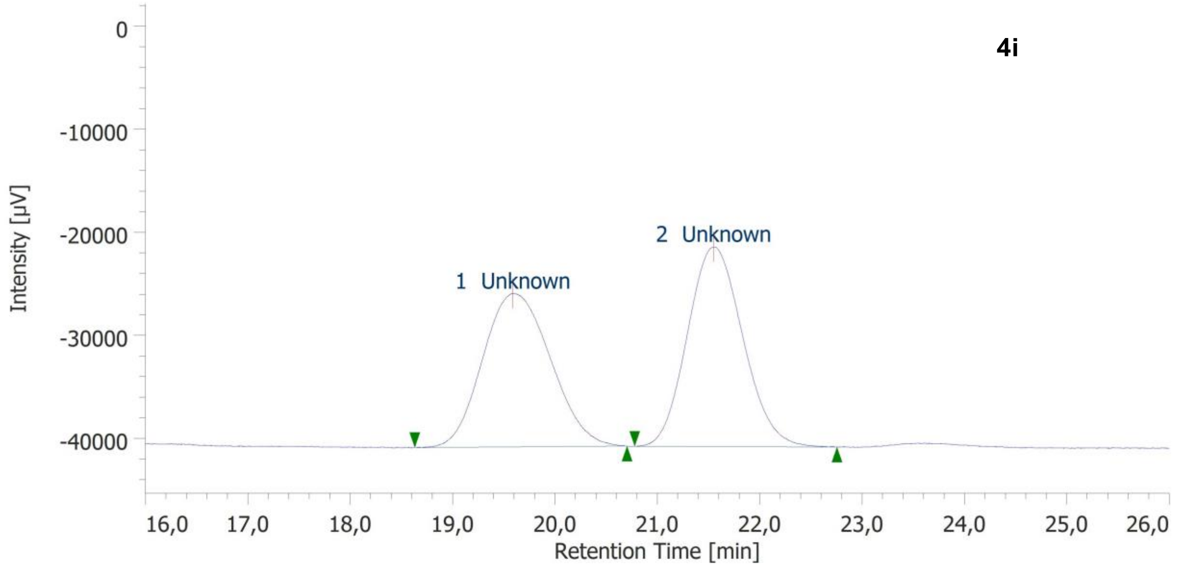

#	Peak Name	CH	tR [min]	Area [µV·sec]	Height [µV]	Area%	Height%	Quantity	NTP	Resolution	Symmetry Factor	Warning
1	Unknown	11	19,587	685193	14875	48,512	43,449	N/A	3923	1,748	1,148	
2	Unknown	11	21,548	727223	19360	51,488	56,551	N/A	7431	N/A	1,121	

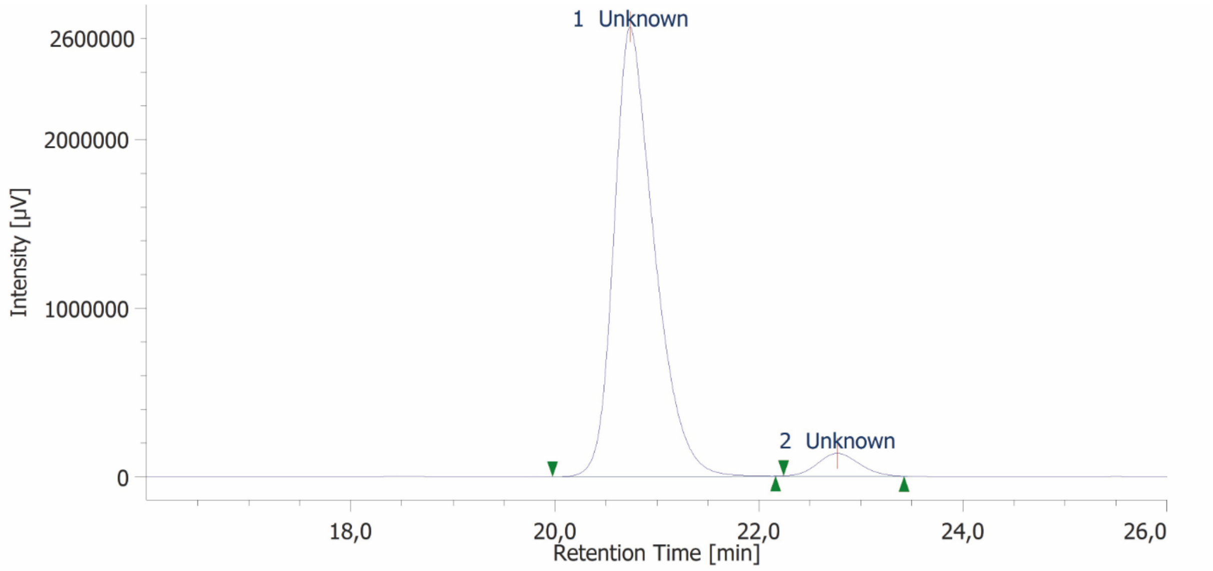

#	Peak Name	CH	tR [min]	Area [µV·sec]	Height [µV]	Area%	Height%	Quantity	NTP	Resolution	Symmetry Factor	Warning
1	Unknown	11	20,737	73539808	2662631	94,848	95,105	N/A	13890	2,780	1,360	
2	Unknown	11	22,767	3994290	137054	5,152	4,895	N/A	14336	N/A	1,061	

Methyl (1*S*,2*S*,3*S*)-3-hydroxy-6,7-dimethyl-1-phenyl-2-(4-(trifluoromethyl)phenyl)-2,3-dihydro-1*H*-pyrrolizine-5-carboxylate (4j)

IA-column: Hex/ *i*PrOH 90:10 $^{v}/_{v}$, 1.0 ml/ min, λ = 284 nm

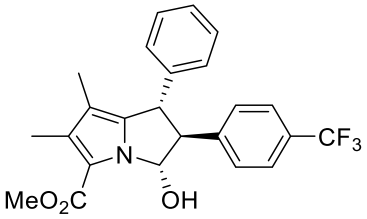

4j

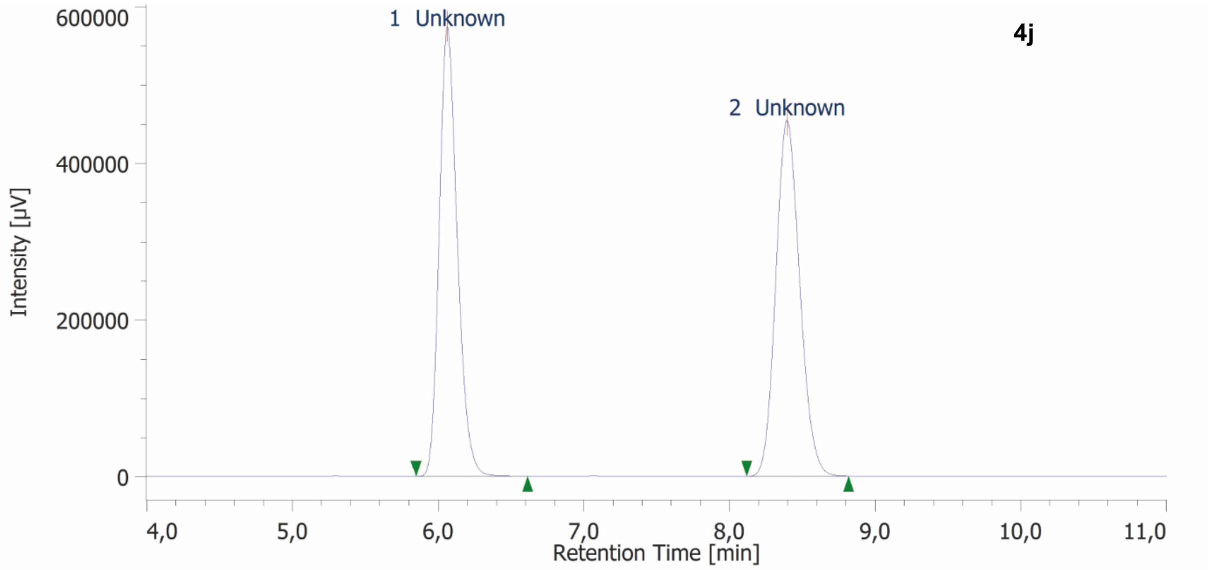

#	Peak Name	CH	tR [min]	Area [µV·sec]	Height [µV]	Area%	Height%	Quantity	NTP	Resolution	Symmetry Factor	Warning
1	Unknown	11	6,060	4950175	574355	48,797	55,819	N/A	11847	8,946	1,237	
2	Unknown	11	8,393	5194301	454600	51,203	44,181	N/A	12494	N/A	1,137	

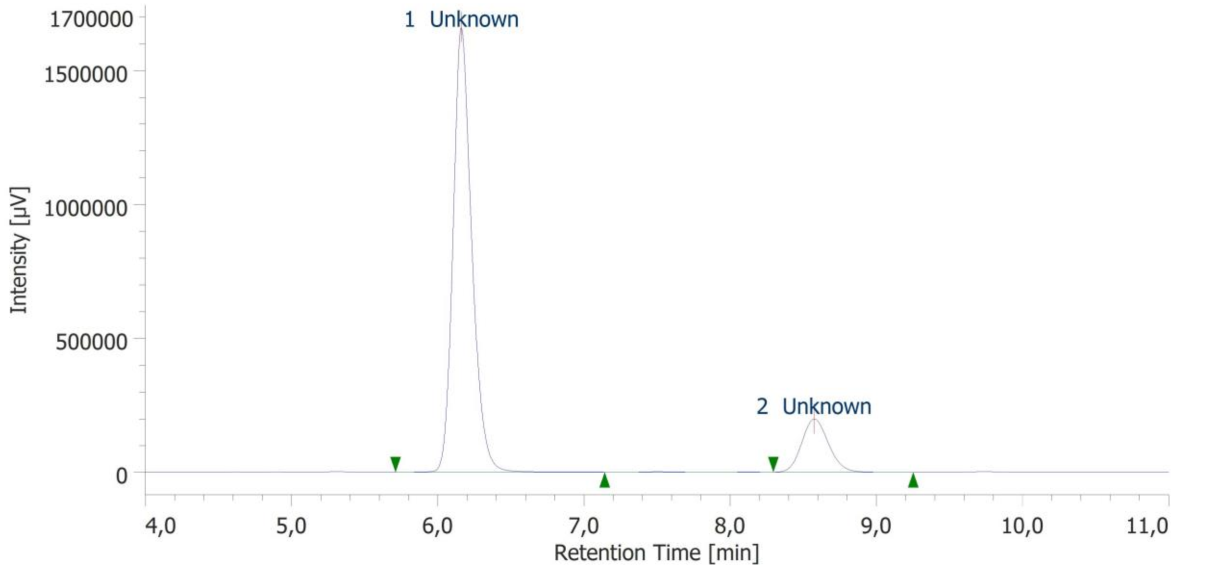

#	Peak Name	CH	tR [min]	Area [µV·sec]	Height [µV]	Area%	Height%	Quantity	NTP	Resolution	Symmetry Factor	Warning
1	Unknown	11	6,160	14794186	1660650	85,443	89,297	N/A	11886	8,673	1,233	
2	Unknown	11	8,573	2520559	199040	14,557	10,703	N/A	10671	N/A	1,137	

Methyl (1*S*,2*S*,3*S*)-6-ethyl-3-hydroxy-7-methyl-1,2-diphenyl-2,3-dihydro-1*H*-pyrrolizine-5-carboxylate (4k)

IA-column: Hex/ *i*PrOH 90:10 $^{v}/_{v}$, 0.5 ml/ min, λ = 284 nm

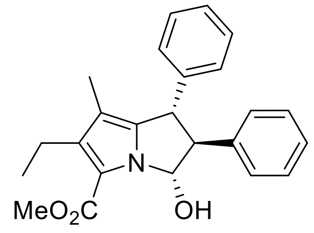

4k

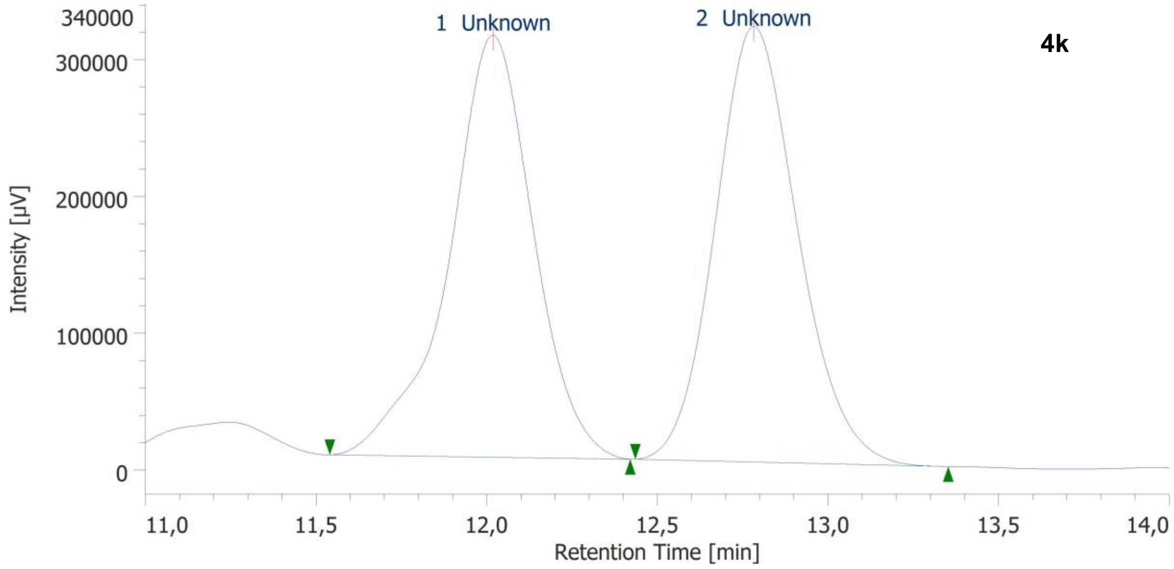

#	Peak Name	CH	tR [min]	Area [µV·sec]	Height [µV]	Area%	Height%	Quantity	NTP	Resolution	Symmetry Factor	Warning
1	Unknown	11	12,018	5283336	308215	49,748	49,201	N/A	13058	1,783	0,894	
2	Unknown	11	12,782	5336906	318225	50,252	50,799	N/A	13635	N/A	1,139	

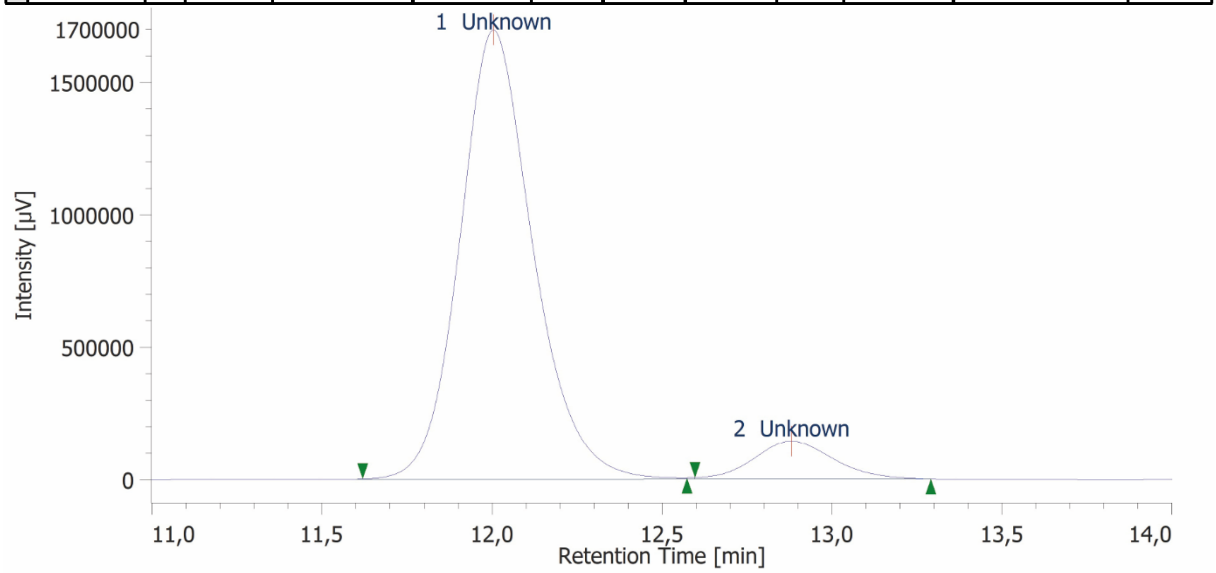

#	Peak Name	CH	tR [min]	Area [µV·sec]	Height [µV]	Area%	Height%	Quantity	NTP	Resolution	Symmetry Factor	Warning
1	Unknown	11	12,003	25328237	1692461	91,660	92,220	N/A	15926	2,200	1,170	
2	Unknown	11	12,880	2304492	142786	8,340	7,780	N/A	15153	N/A	1,084	

Methyl (1*S*,2*S*,3*S*)-3-hydroxy-1,2-diphenyl-2,3,6,7,8,9-hexahydro-1*H*-pyrrolo[2,1-*a*]isoindole-5-carboxylate (4l)

IA-column: Hex/ *i*PrOH 90:10 $^{v}/_{v}$, 1.0 ml/ min, λ = 284 nm

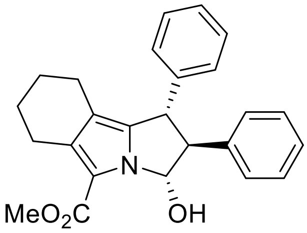

4l

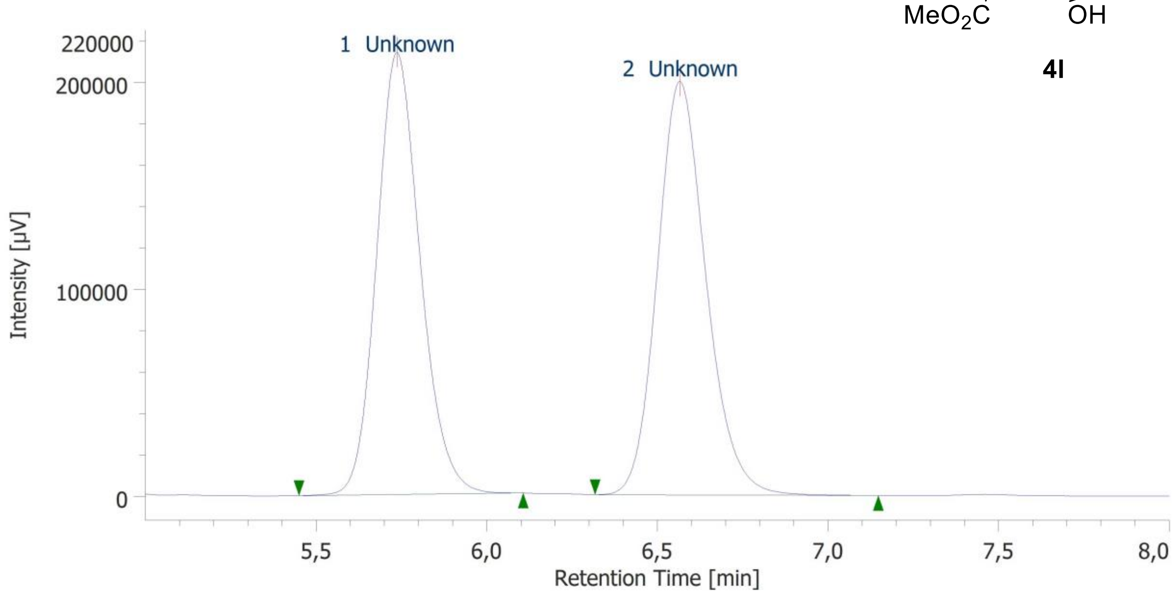

#	Peak Name	CH	tR [min]	Area [µV·sec]	Height [µV]	Area%	Height%	Quantity	NTP	Resolution	Symmetry Factor	Warning
1	Unknown	11	5,737	1893220	213529	48,858	51,676	N/A	10016	3,431	1,156	
2	Unknown	11	6,567	1981759	199682	51,142	48,324	N/A	10536	N/A	1,161	

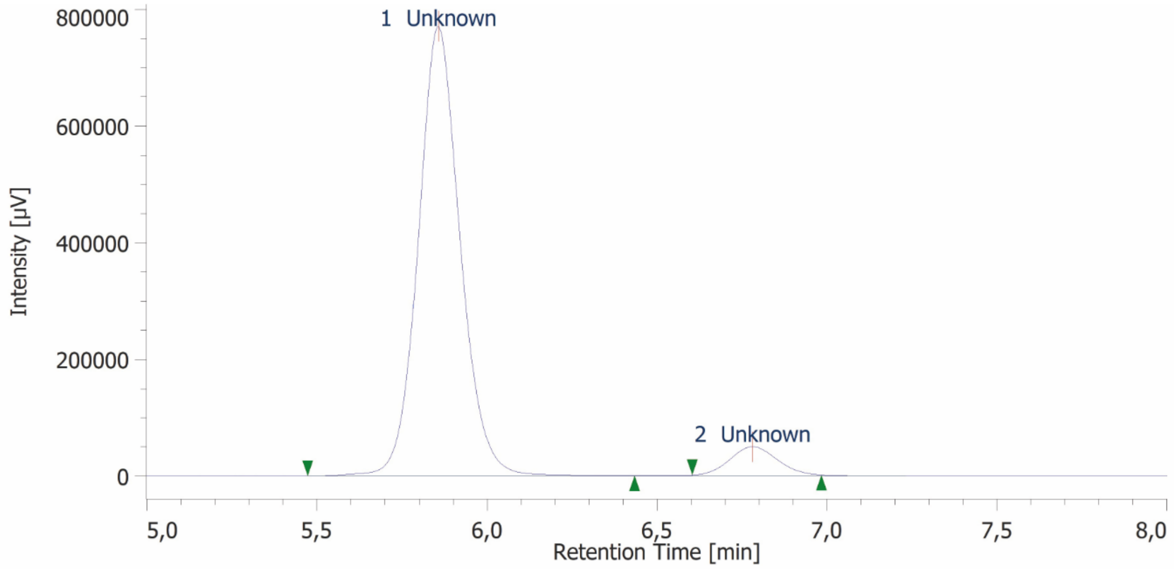

#	Peak Name	CH	tR [min]	Area [µV·sec]	Height [µV]	Area%	Height%	Quantity	NTP	Resolution	Symmetry Factor	Warning
1	Unknown	11	5,857	6605796	770114	92,980	93,885	N/A	11597	3,878	1,097	
2	Unknown	11	6,780	498717	50157	7,020	6,115	N/A	10882	N/A	1,090	

Methyl (1*S*,2*S*,3*S*)-3-hydroxy-2-(4-methoxyphenyl)-6,7-dimethyl-1-(naphthalen-2-yl)-2,3-dihydro-1*H*-pyrrolizine-5-carboxylate (4m)

IA-column: Hex/ *i*PrOH 90:10 $^{v}/_{v}$, 1.0 ml/ min, λ = 284 nm

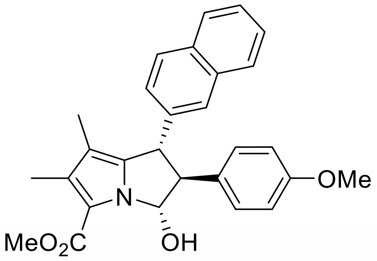

4m

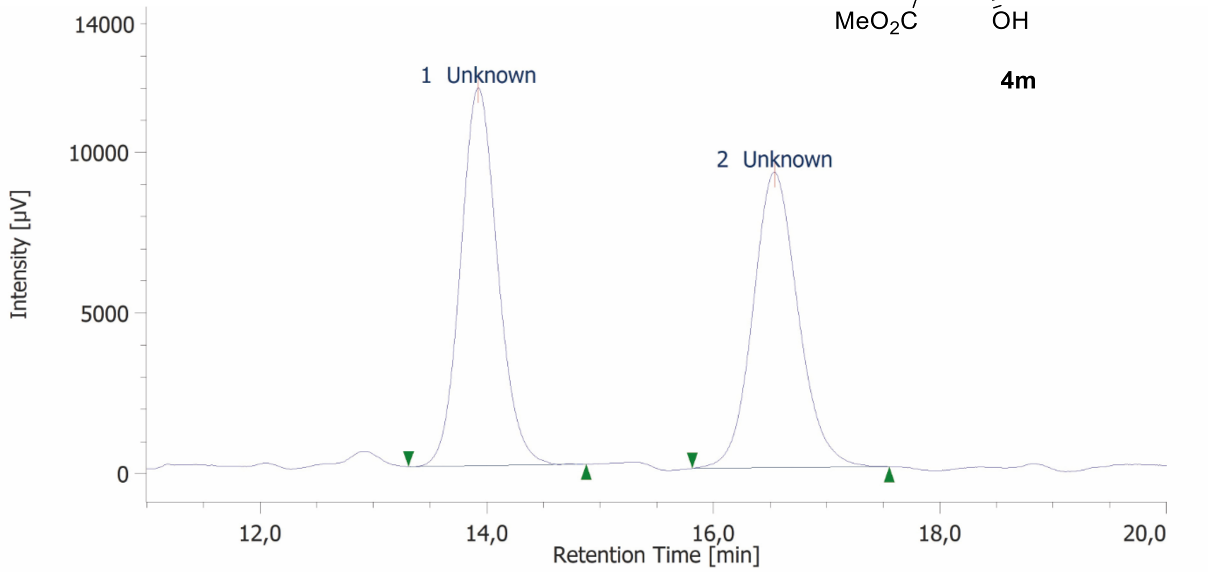

#	Peak Name	CH	tR [min]	Area [µV·sec]	Height [µV]	Area%	Height%	Quantity	NTP	Resolution	Symmetry Factor	Warning
1	Unknown	11	13,923	267942	11763	51,160	56,104	N/A	8869	4,018	1,110	
2	Unknown	11	16,540	255796	9203	48,840	43,896	N/A	8575	N/A	1,110	

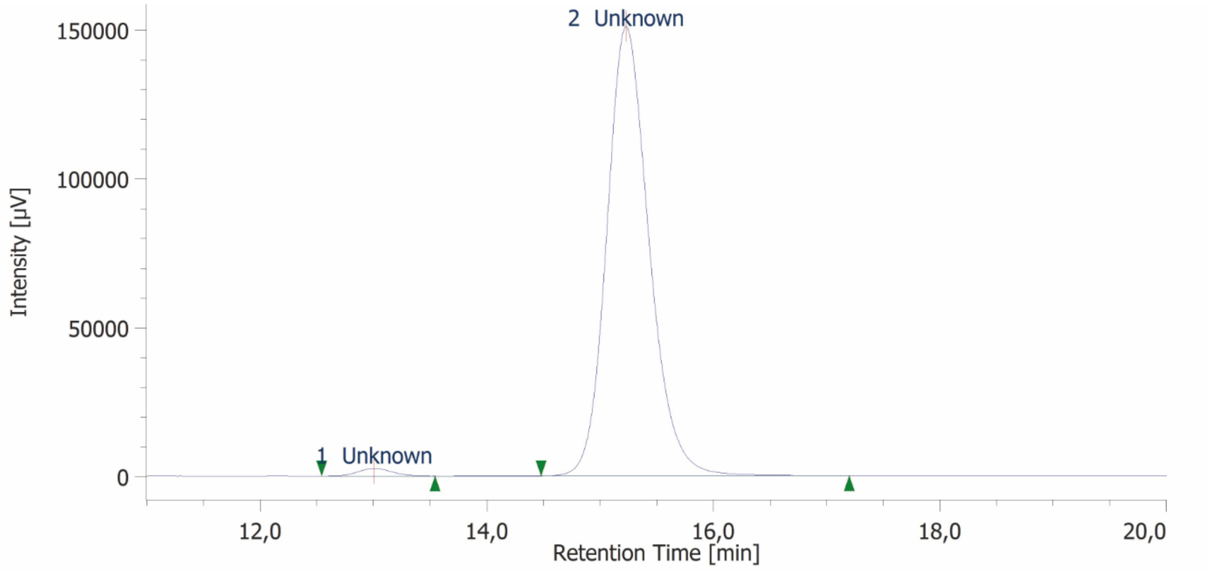

#	Peak Name	CH	tR [min]	Area [µV·sec]	Height [µV]	Area%	Height%	Quantity	NTP	Resolution	Symmetry Factor	Warning
1	Unknown	11	13,003	52733	2570	1,327	1,676	N/A	9220	3,691	1,104	
2	Unknown	11	15,227	3922095	150795	98,673	98,324	N/A	8353	N/A	1,185	

Methyl (1*S*,2*S*,3*S*)-3-hydroxy-1,2-bis(4-methoxyphenyl)-6,7-dimethyl-2,3-dihydro-1*H*-pyrrolizine-5-carboxylate (4n)

IA-column: Hex/ *i*PrOH 90:10 $^v/_v$, 1.0 ml/ min, λ = 284 nm

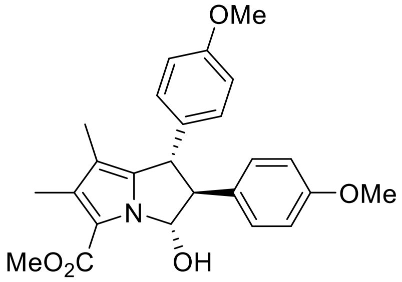

4n

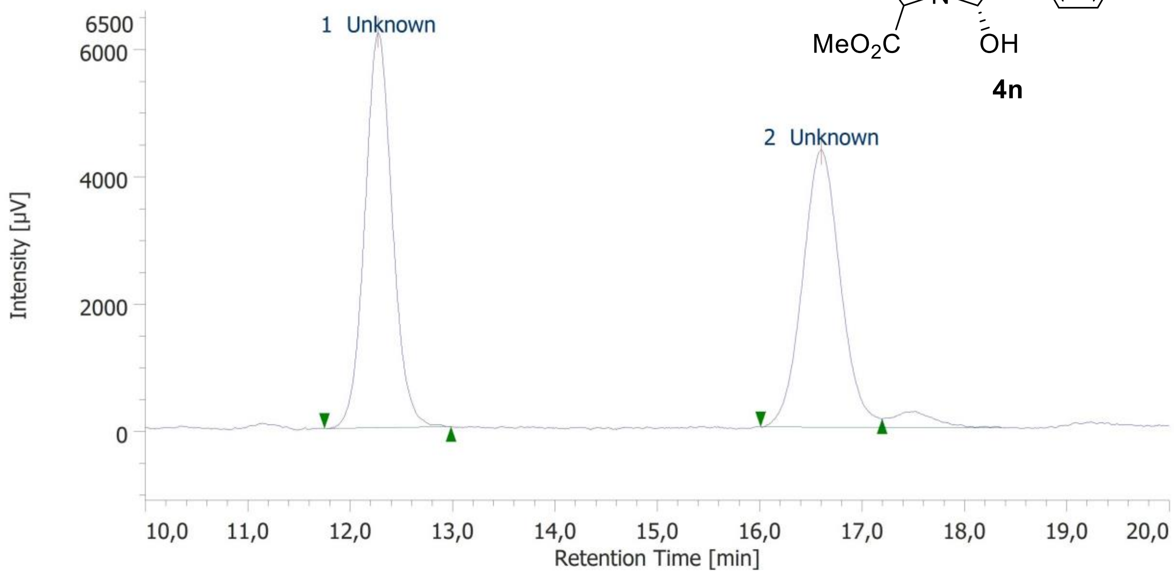

#	Peak Name	CH	tR [min]	Area [µV·sec]	Height [µV]	Area%	Height%	Quantity	NTP	Resolution	Symmetry Factor	Warning
1	Unknown	11	12,273	115924	6201	50,963	58,739	N/A	10401	7,588	1,073	
2	Unknown	11	16,600	111542	4356	49,037	41,261	N/A	10058	N/A	1,075	

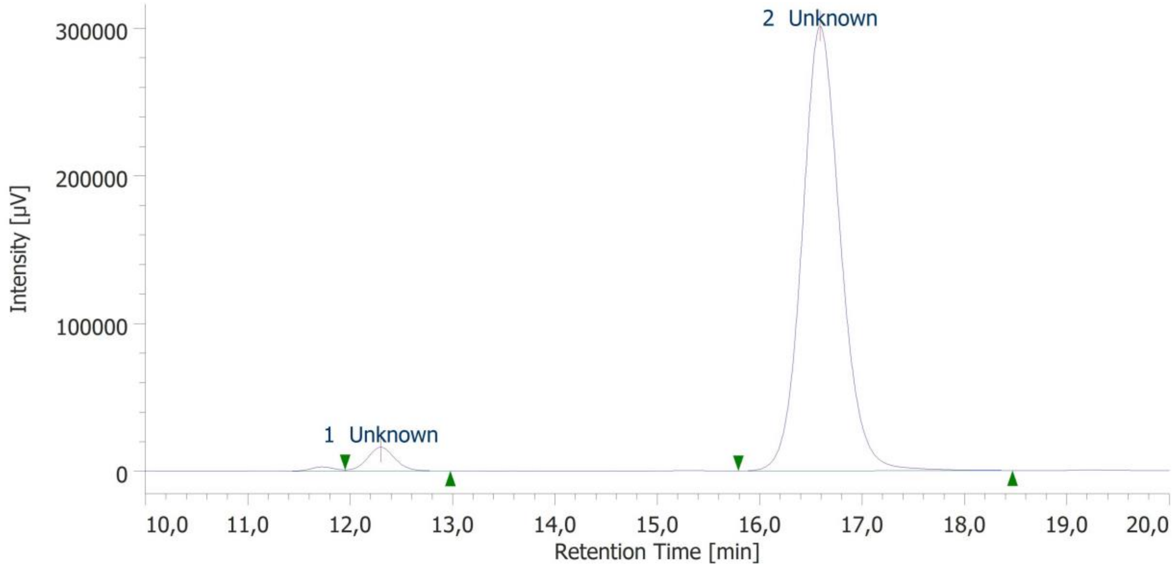

#	Peak Name	CH	tR [min]	Area [µV·sec]	Height [µV]	Area%	Height%	Quantity	NTP	Resolution	Symmetry Factor	Warning
1	Unknown	11	12,297	299994	16080	3,691	5,073	N/A	10500	7,519	1,012	
2	Unknown	11	16,587	7827930	300875	96,309	94,927	N/A	9982	N/A	1,115	

Methyl (1*S*,2*S*,3*S*)-1-(benzo[*d*][1,3]dioxol-5-yl)-3-hydroxy-2-(4-methoxyphenyl)-6,7-dimethyl-2,3-dihydro-1*H*-pyrrolizine-5-carboxylate (4o)

IA-column: Hex/ *i*PrOH 90:10 $^{v}/_{v}$, 1.0 ml/ min, λ = 284 nm

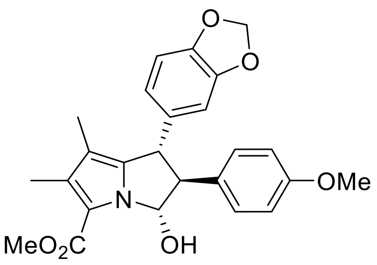

4o

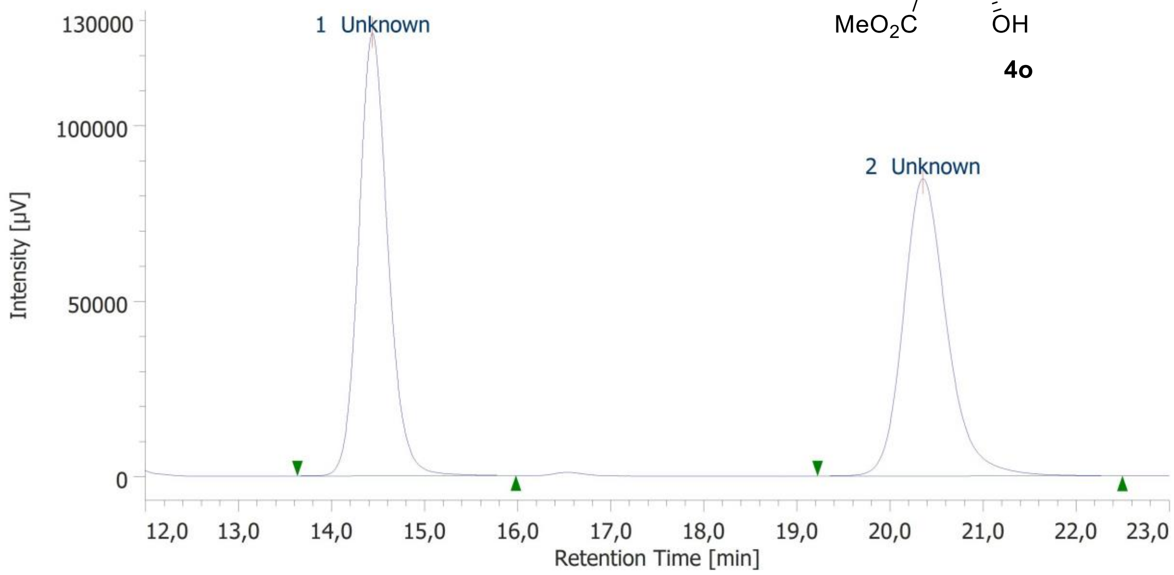

#	Peak Name	CH	tR [min]	Area [µV·sec]	Height [µV]	Area%	Height%	Quantity	NTP	Resolution	Symmetry Factor	Warning
1	Unknown	11	14,437	2762376	126233	49,981	59,858	N/A	10636	8,582	1,141	
2	Unknown	11	20,350	2764507	84654	50,019	40,142	N/A	9810	N/A	1,226	

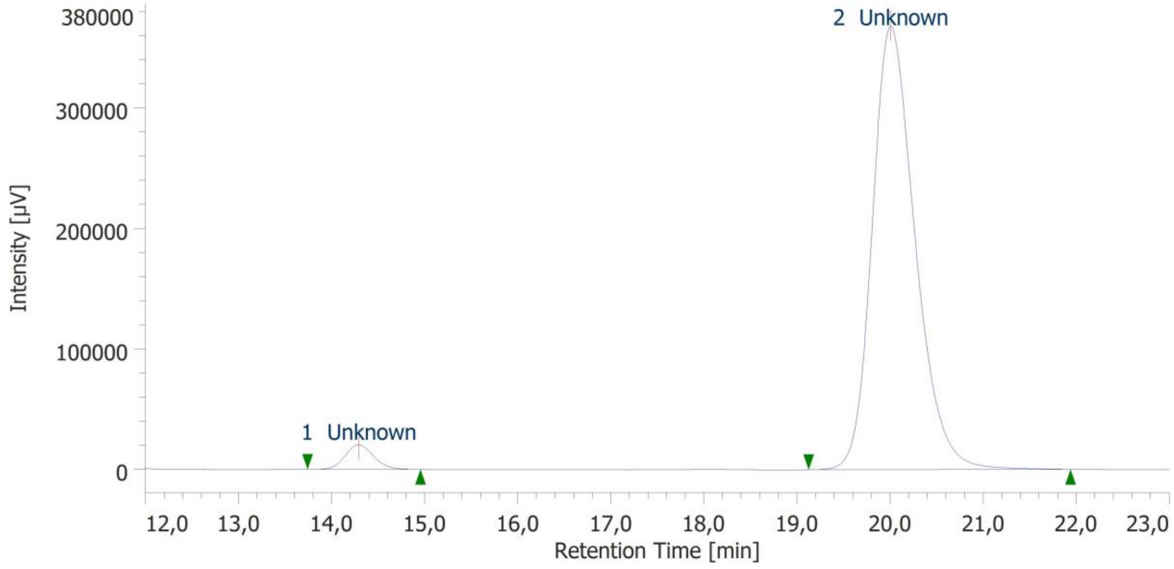

#	Peak Name	CH	tR [min]	Area [µV·sec]	Height [µV]	Area%	Height%	Quantity	NTP	Resolution	Symmetry Factor	Warning
1	Unknown	11	14,290	435833	20419	3,575	5,257	N/A	10587	8,317	1,112	
2	Unknown	11	20,003	11756723	367975	96,425	94,743	N/A	9476	N/A	1,264	

Methyl (1*S*,2*S*,3*S*)-1-(3,5-dimethylphenyl)-3-hydroxy-2-(4-methoxyphenyl)-6,7-dimethyl-2,3-dihydro-1*H*-pyrrolizine-5-carboxylate (4p)

IE-column: Hex/ *i*PrOH 90:10 $^v/_v$, 1.0 ml/ min, λ = 284 nm

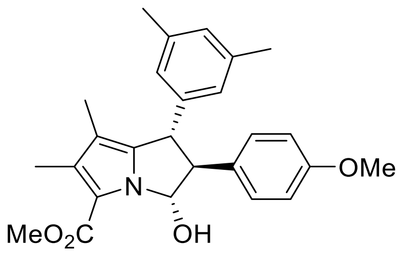

4p

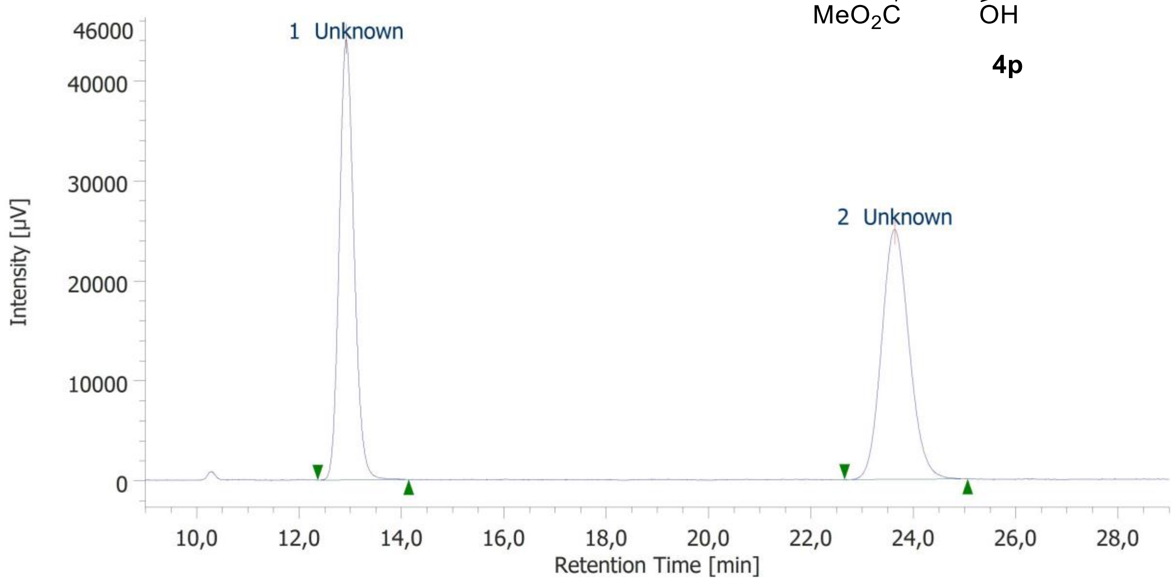

#	Peak Name	CH	tR [min]	Area [µV·sec]	Height [µV]	Area%	Height%	Quantity	NTP	Resolution	Symmetry Factor	Warning
1	Unknown	11	12,920	882393	44021	48,628	63,810	N/A	9818	14,332	1,150	
2	Unknown	11	23,633	932182	24967	51,372	36,190	N/A	9353	N/A	1,119	

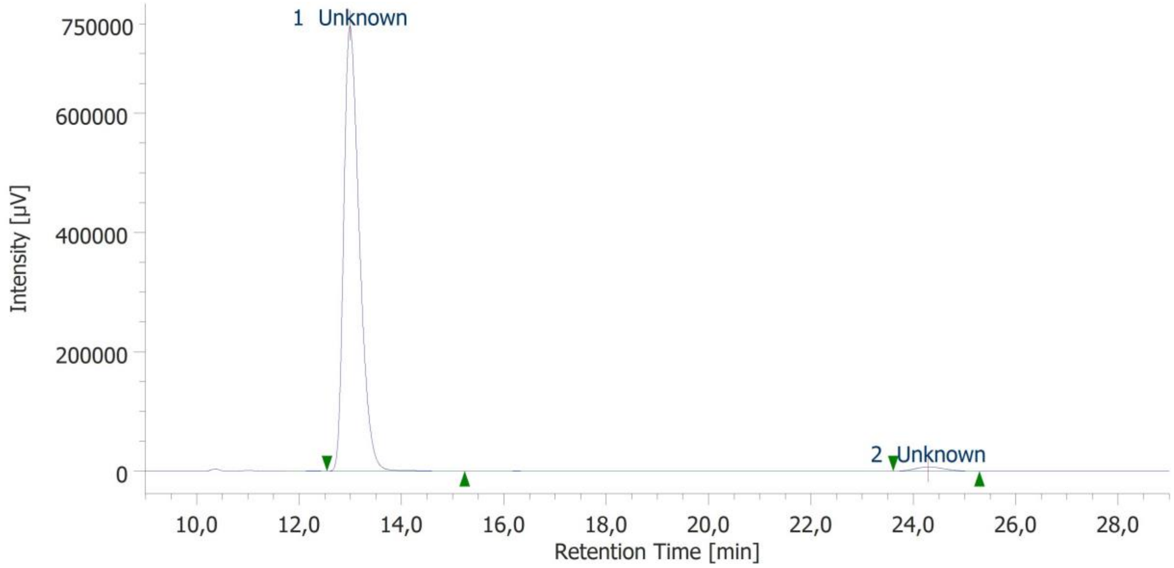

#	Peak Name	CH	tR [min]	Area [µV·sec]	Height [µV]	Area%	Height%	Quantity	NTP	Resolution	Symmetry Factor	Warning
1	Unknown	11	12,993	15847979	746185	98,241	99,050	N/A	8826	14,083	1,434	
2	Unknown	11	24,290	283737	7159	1,759	0,950	N/A	8475	N/A	1,141	

Methyl (1*S*,2*S*,3*S*)-1-(4-(*tert*-butyl)phenyl)-3-hydroxy-2-(4-methoxyphenyl)-6,7-dimethyl-2,3-dihydro-1*H*-pyrrolizine-5-carboxylate (4q)

IE-column: Hex/ *i*PrOH 90:10 $^{v}/_{v}$, 1.0 ml/ min, λ = 284 nm

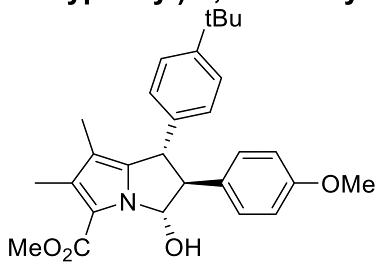

4q

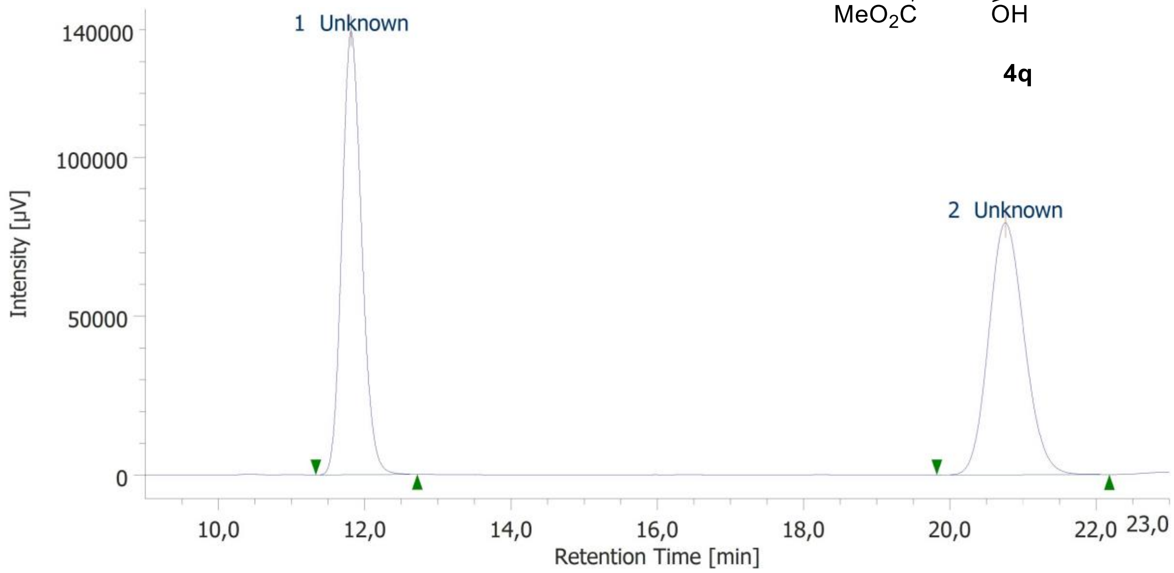

#	Peak Name	CH	tR [min]	Area [µV·sec]	Height [µV]	Area%	Height%	Quantity	NTP	Resolution	Symmetry Factor	Warning
1	Unknown	11	11,810	2613499	139402	49,246	63,735	N/A	9251	12,945	1,159	
2	Unknown	11	20,757	2693559	79318	50,754	36,265	N/A	8611	N/A	1,128	

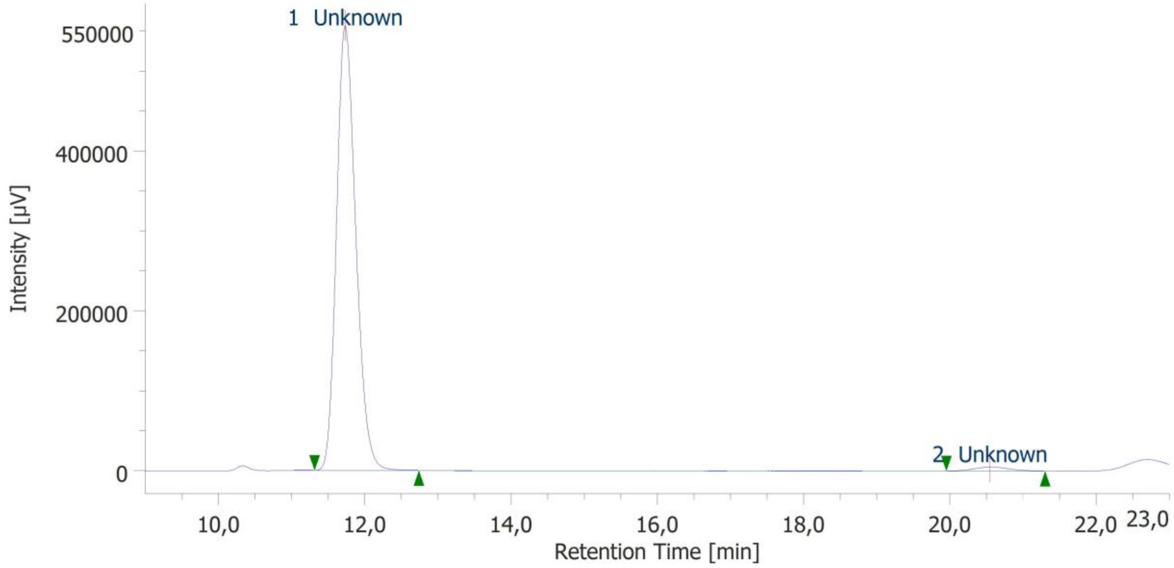

#	Peak Name	CH	tR [min]	Area [µV·sec]	Height [µV]	Area%	Height%	Quantity	NTP	Resolution	Symmetry Factor	Warning
1	Unknown	11	11,730	10207869	556295	98,414	99,111	N/A	9570	13,094	1,211	
2	Unknown	11	20,543	164453	4992	1,586	0,889	N/A	8919	N/A	1,176	

Methyl (1*S*,2*S*,3*S*)-3-hydroxy-2-(4-methoxyphenyl)-6,7-dimethyl-1-(*p*-tolyl)-2,3-dihydro-1*H*-pyrrolizine-5-carboxylate (4r)

IA-column: Hex/ *i*PrOH 90:10 $^{v}/_{v}$, 1.0 ml/ min, λ = 284 nm

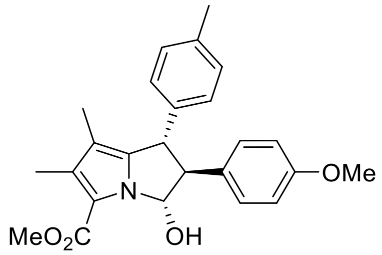

4r

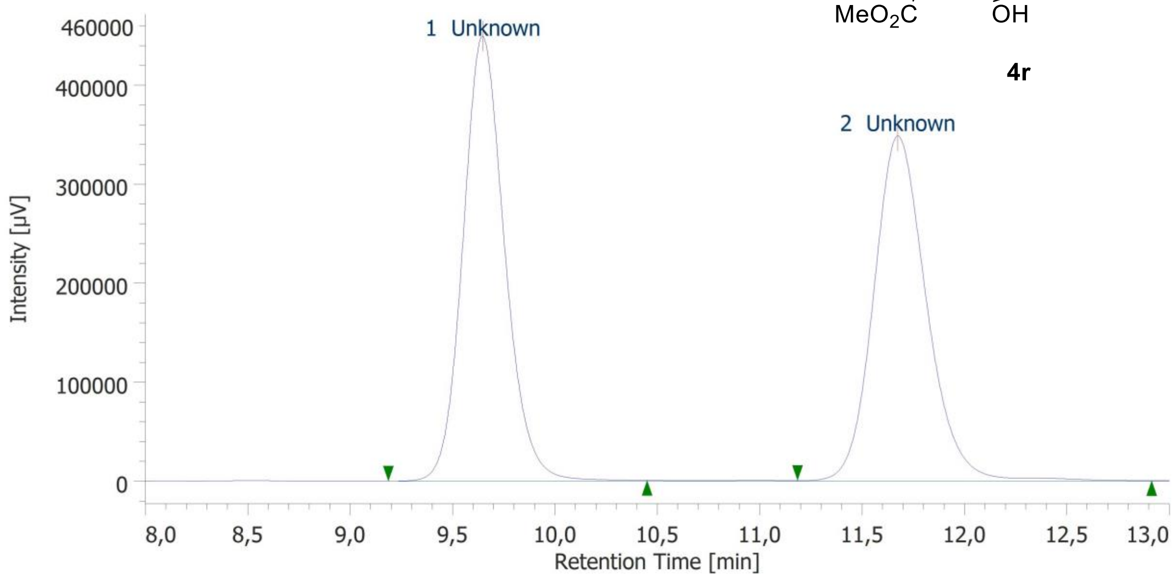

#	Peak Name	CH	tR [min]	Area [µV·sec]	Height [µV]	Area%	Height%	Quantity	NTP	Resolution	Symmetry Factor	Warning
1	Unknown	11	9,647	6380828	448943	50,400	56,303	N/A	11230	4,936	1,130	
2	Unknown	11	11,673	6279482	348424	49,600	43,697	N/A	10335	N/A	1,158	

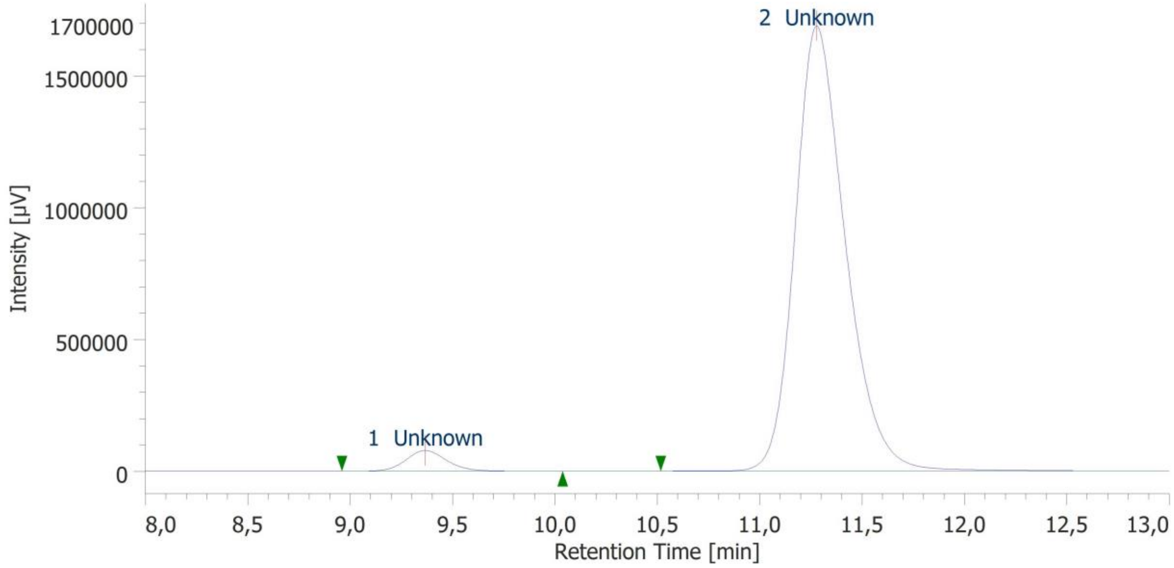

#	Peak Name	CH	tR [min]	Area [µV·sec]	Height [µV]	Area%	Height%	Quantity	NTP	Resolution	Symmetry Factor	Warning
1	Unknown	11	9,367	1067169	78151	3,568	4,424	N/A	11241	4,857	1,110	
2	Unknown	11	11,277	28842416	1688315	96,432	95,576	N/A	10739	N/A	1,264	

Methyl (1*S*,2*S*,3*S*)-3-hydroxy-1-(3-methoxyphenyl)-2-(4-methoxyphenyl)-6,7-dimethyl-2,3-dihydro-1*H*-pyrrolizine-5-carboxylate (4s)

IE-column: Hex/ *i*PrOH 90:10 v/v, 1.0 ml/ min, λ = 284 nm

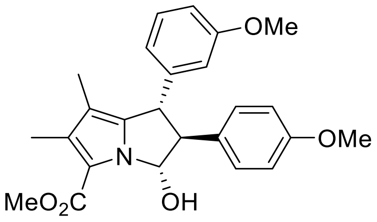

4s

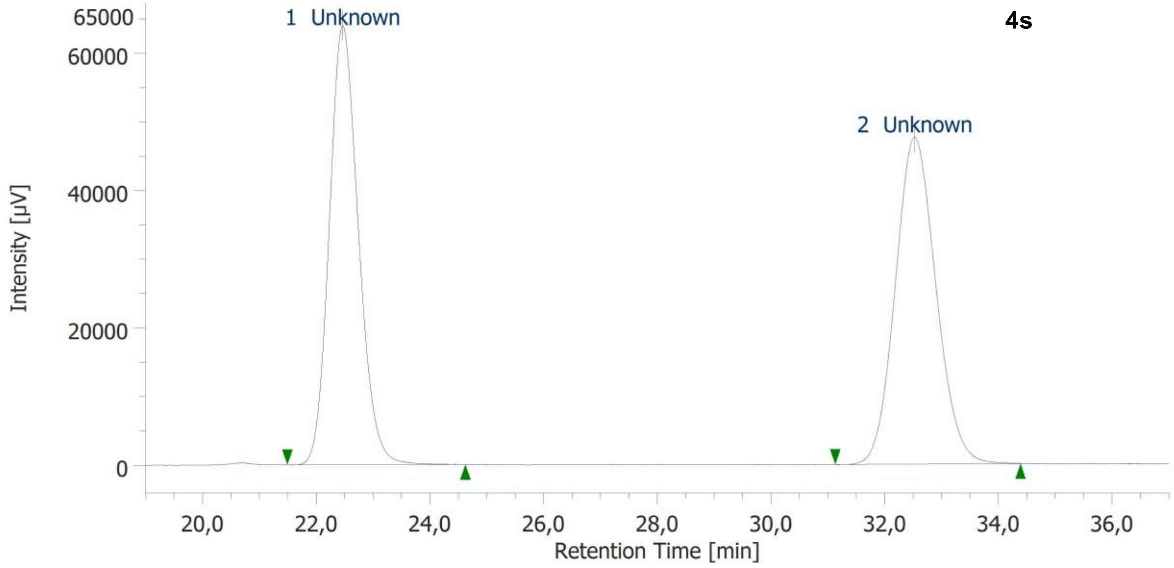

#	Peak Name	CH	tR [min]	Area [µV·sec]	Height [µV]	Area%	Height%	Quantity	NTP	Resolution	Symmetry Factor	Warning
1	Unknown	11	22,467	2275016	63897	49,079	57,324	N/A	9377	9,032	1,155	
2	Unknown	11	32,523	2360364	47569	50,921	42,676	N/A	9940	N/A	1,102	

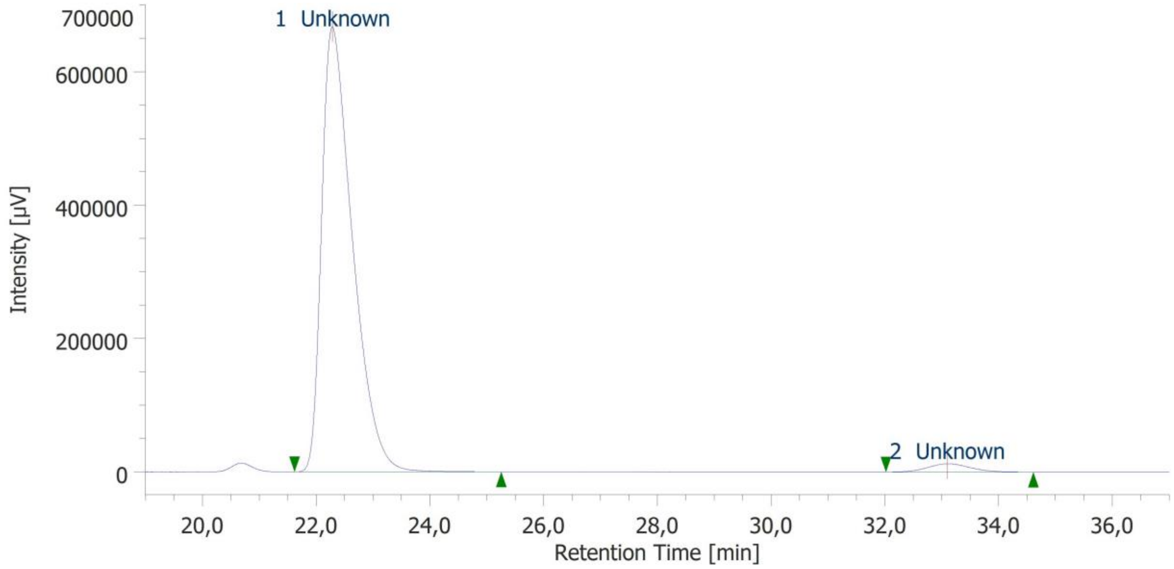

#	Peak Name	CH	tR [min]	Area [µV·sec]	Height [µV]	Area%	Height%	Quantity	NTP	Resolution	Symmetry Factor	Warning
1	Unknown	11	22,283	25096291	667089	97,534	98,184	N/A	8345	9,301	1,686	
2	Unknown	11	33,093	634487	12336	2,466	1,816	N/A	9543	N/A	1,112	

Methyl (1*S*,2*S*,3*S*)-1-(4-bromophenyl)-3-hydroxy-2-(4-methoxyphenyl)-6,7-dimethyl-2,3-dihydro-1*H*-pyrrolizine-5-carboxylate (4t)

IE-column: Hex/ *i*PrOH 90:10 $^{v}/_{v}$, 1.0 ml/ min, λ = 284 nm

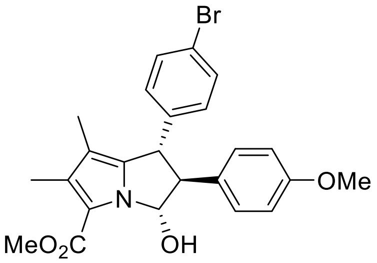

4t

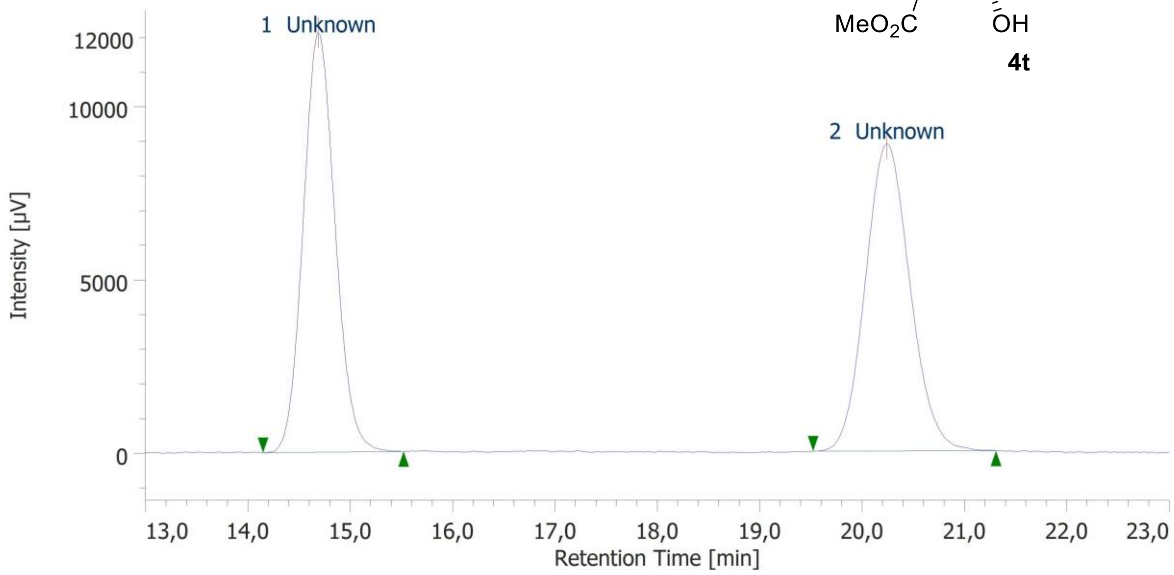

#	Peak Name	CH	tR [min]	Area [µV·sec]	Height [µV]	Area%	Height%	Quantity	NTP	Resolution	Symmetry Factor	Warning
1	Unknown	11	14,687	262252	12099	49,411	57,741	N/A	10681	8,157	1,115	
2	Unknown	11	20,240	268509	8855	50,589	42,259	N/A	10322	N/A	1,099	

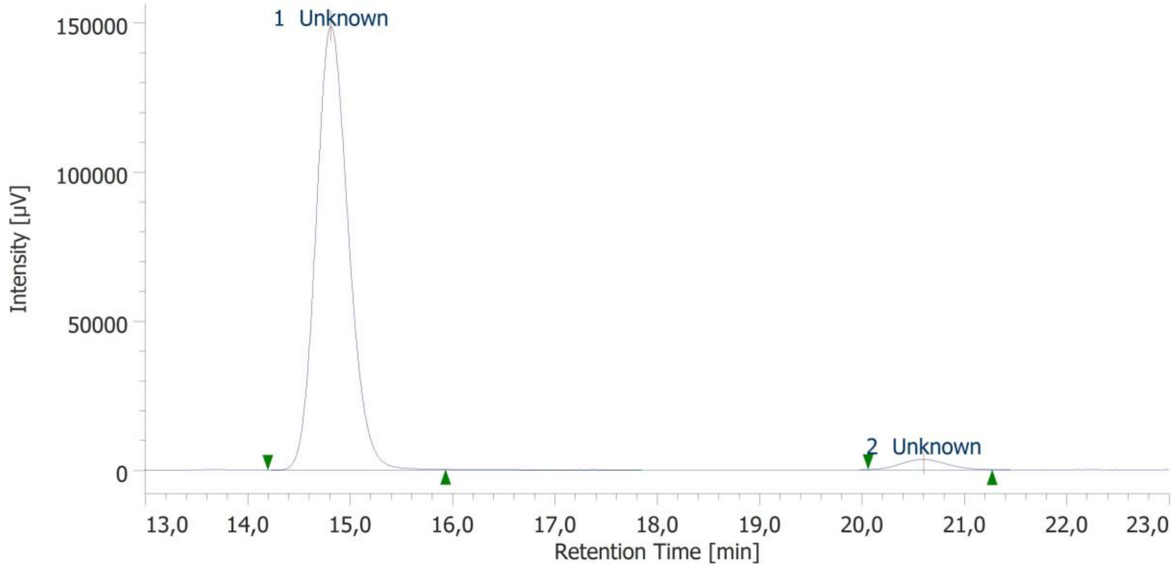

#	Peak Name	CH	tR [min]	Area [µV·sec]	Height [µV]	Area%	Height%	Quantity	NTP	Resolution	Symmetry Factor	Warning
1	Unknown	11	14,810	3282334	148706	96,731	97,684	N/A	10555	8,245	1,162	
2	Unknown	11	20,600	110931	3526	3,269	2,316	N/A	9817	N/A	1,082	

Methyl (1*S*,2*S*,3*S*)-1-(2,4-dimethoxyphenyl)-3-hydroxy-2-(4-methoxyphenyl)-6,7-dimethyl-2,3-dihydro-1*H*-pyrrolizine-5-carboxylate (4u)

IA-column: Hex/ *i*PrOH 90:10 v/v, 1.0 ml/ min, λ = 284 nm

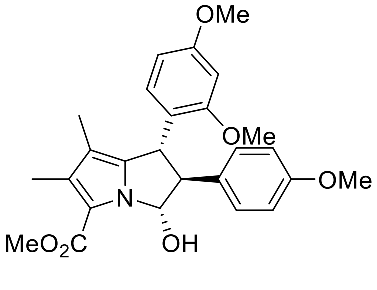

4u

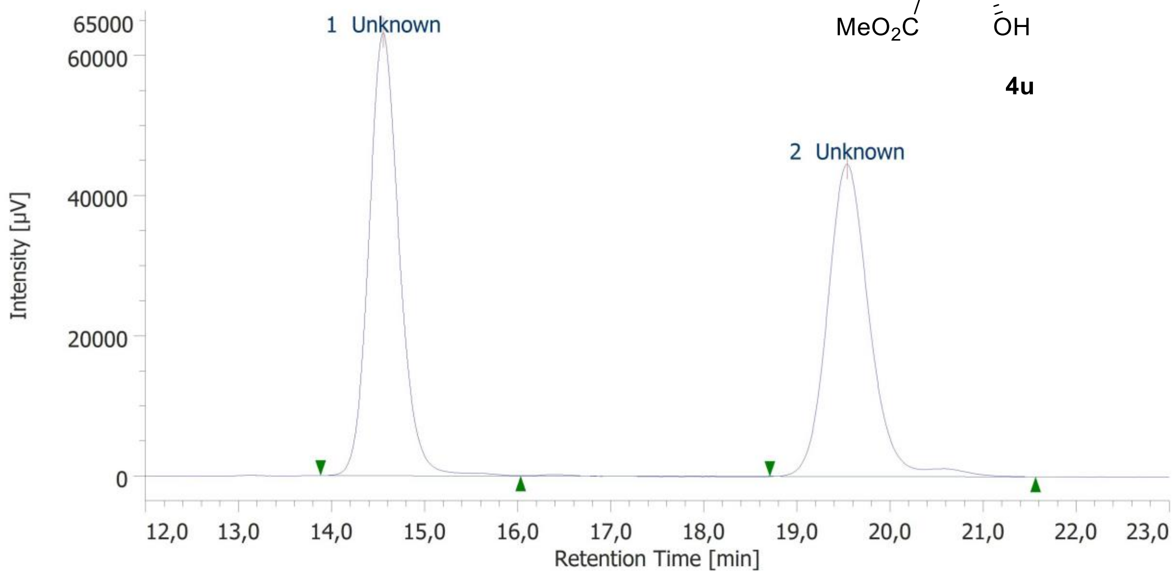

#	Peak Name	CH	tR [min]	Area [µV·sec]	Height [µV]	Area%	Height%	Quantity	NTP	Resolution	Symmetry Factor	Warning
1	Unknown	11	14,553	1432726	63134	50,557	58,641	N/A	10112	7,303	1,138	
2	Unknown	11	19,537	1401175	44527	49,443	41,359	N/A	9798	N/A	1,129	

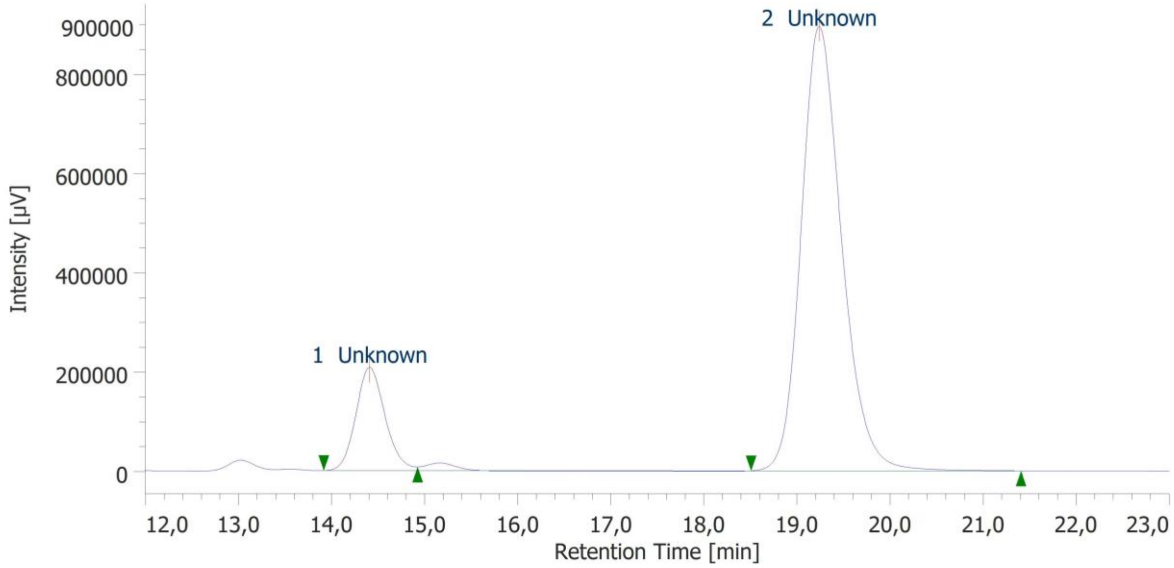

#	Peak Name	CH	tR [min]	Area [µV·sec]	Height [µV]	Area%	Height%	Quantity	NTP	Resolution	Symmetry Factor	Warning
1	Unknown	11	14,407	4534688	207826	14,376	18,840	N/A	10384	7,231	1,158	
2	Unknown	11	19,237	27008270	895303	85,624	81,160	N/A	9883	N/A	1,212	

Methyl (1*S*,2*S*,3*S*)-3-hydroxy-2-(4-methoxyphenyl)-1-phenyl-2,3,6,7,8,9-hexahydro-1*H*-pyrrolo[2,1-*a*]isoindole-5-carboxylate (4v)

IA-column: Hex/ *i*PrOH 90:10 $^{v}/_{v}$, 1.0 ml/ min, λ = 284 nm

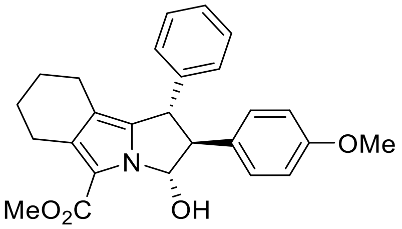

4v

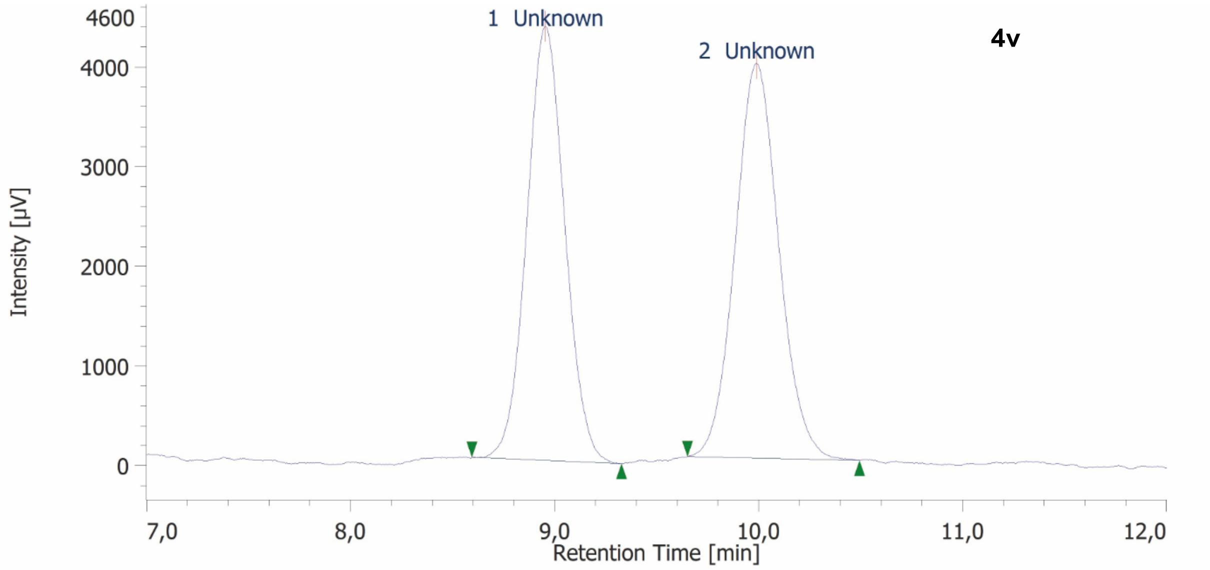

#	Peak Name	CH	tR [min]	Area [µV·sec]	Height [µV]	Area%	Height%	Quantity	NTP	Resolution	Symmetry Factor	Warning
1	Unknown	11	8,953	56448	4349	48,848	52,374	N/A	11091	2,853	1,045	
2	Unknown	11	9,990	59111	3955	51,152	47,626	N/A	10578	N/A	1,067	

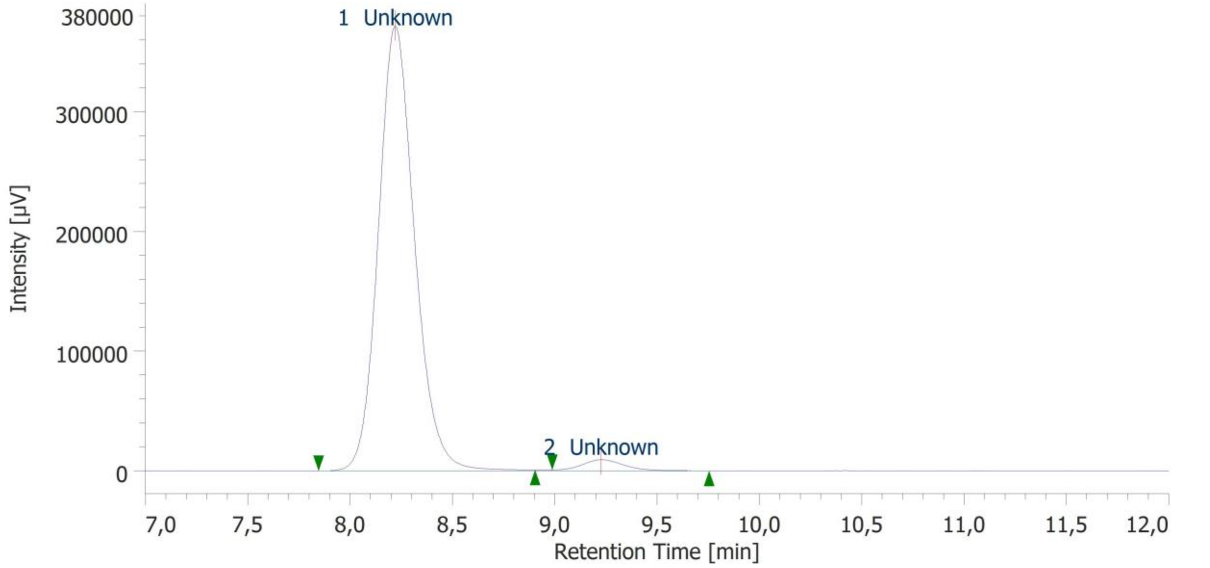

#	Peak Name	CH	tR [min]	Area [µV·sec]	Height [µV]	Area%	Height%	Quantity	NTP	Resolution	Symmetry Factor	Warning
1	Unknown	11	8,220	4571304	371587	97,202	97,588	N/A	10902	2,973	1,152	
2	Unknown	11	9,227	131587	9183	2,798	2,412	N/A	10273	N/A	N/A	

Methyl (1*S*,2*S*,3*S*)-6-ethyl-3-hydroxy-2-(4-methoxyphenyl)-7-methyl-1-phenyl-2,3-dihydro-1*H*-pyrrolizine-5-carboxylate (4w)

IE-column: Hex/ *i*PrOH 90:10 $^{v}/_{v}$, 1.0 ml/ min, λ = 284 nm

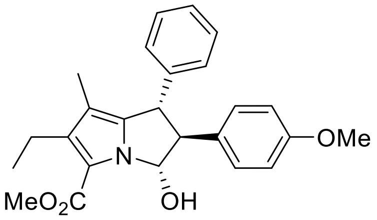

4w

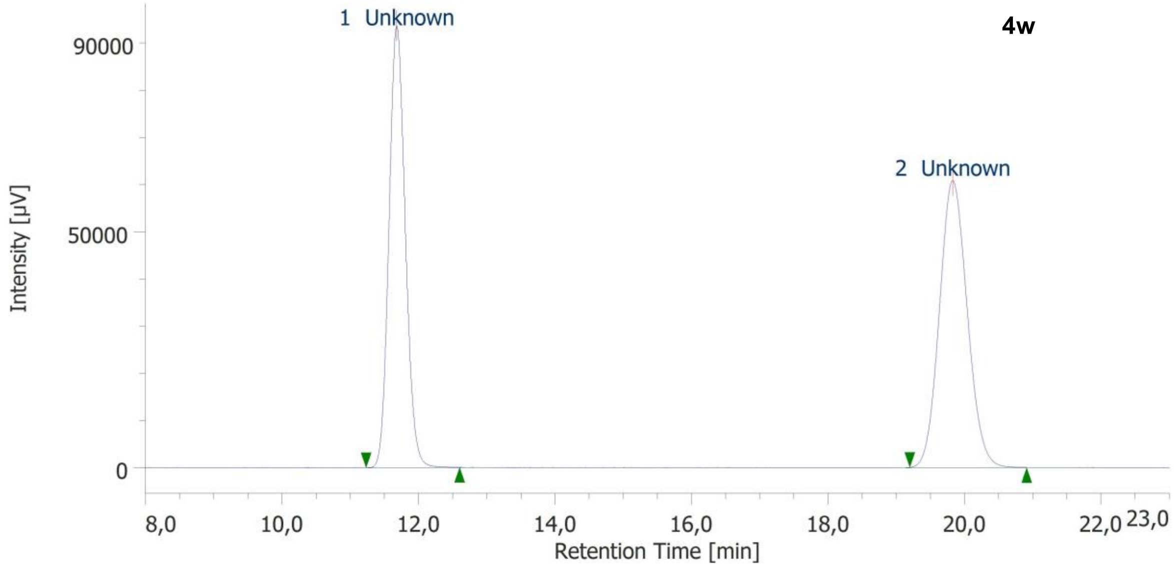

#	Peak Name	CH	tR [min]	Area [µV·sec]	Height [µV]	Area%	Height%	Quantity	NTP	Resolution	Symmetry Factor	Warning
1	Unknown	11	11,680	1557970	93620	47,469	60,611	N/A	11506	13,821	1,119	
2	Unknown	11	19,823	1724096	60841	52,531	39,389	N/A	11299	N/A	1,098	

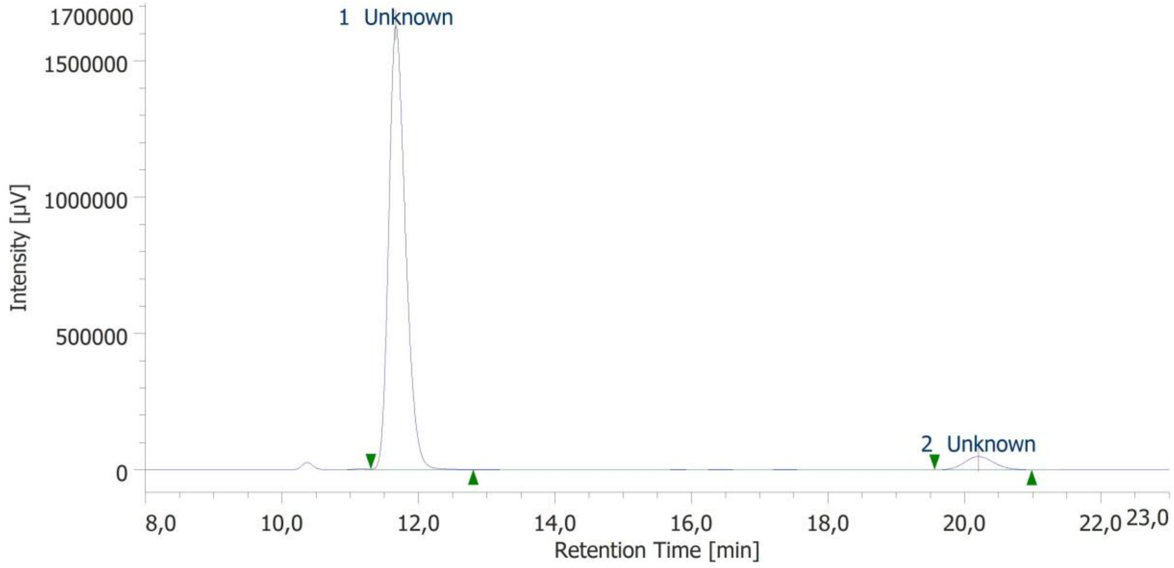

#	Peak Name	CH	tR [min]	Area [µV·sec]	Height [µV]	Area%	Height%	Quantity	NTP	Resolution	Symmetry Factor	Warning
1	Unknown	11	11,667	27712378	1628812	95,033	97,118	N/A	11084	13,834	1,285	
2	Unknown	11	20,200	1448560	48343	4,967	2,882	N/A	10363	N/A	1,105	

Methyl (1*S*,2*S*,3*S*)-3-hydroxy-2-(4-methoxyphenyl)-7-methyl-1,6-diphenyl-2,3-dihydro-1*H*-pyrrolizine-5-carboxylate (4x)

IA-column: Hex/ *i*PrOH 90:10 $^{v}/_{v}$, 1.0 ml/ min, λ = 284 nm

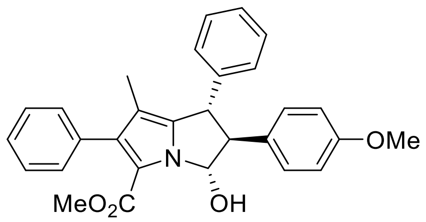

4x

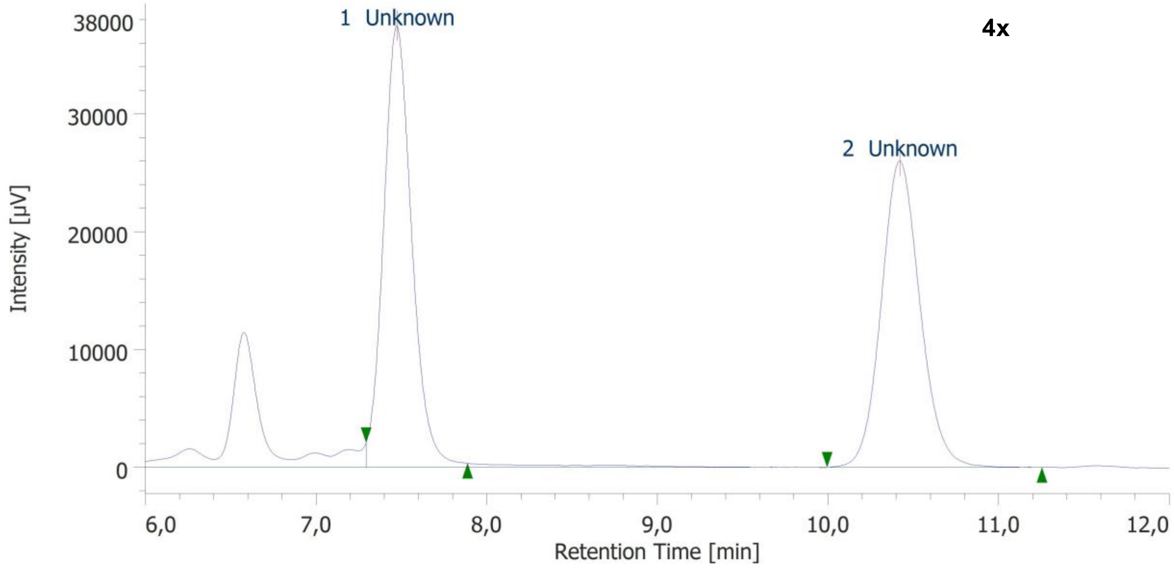

#	Peak Name	CH	tR [min]	Area [µV·sec]	Height [µV]	Area%	Height%	Quantity	NTP	Resolution	Symmetry Factor	Warning
1	Unknown	11	7,473	431144	37453	51,173	59,018	N/A	10223	8,396	N/A	
2	Unknown	11	10,420	411384	26007	48,827	40,982	N/A	10429	N/A	1,102	

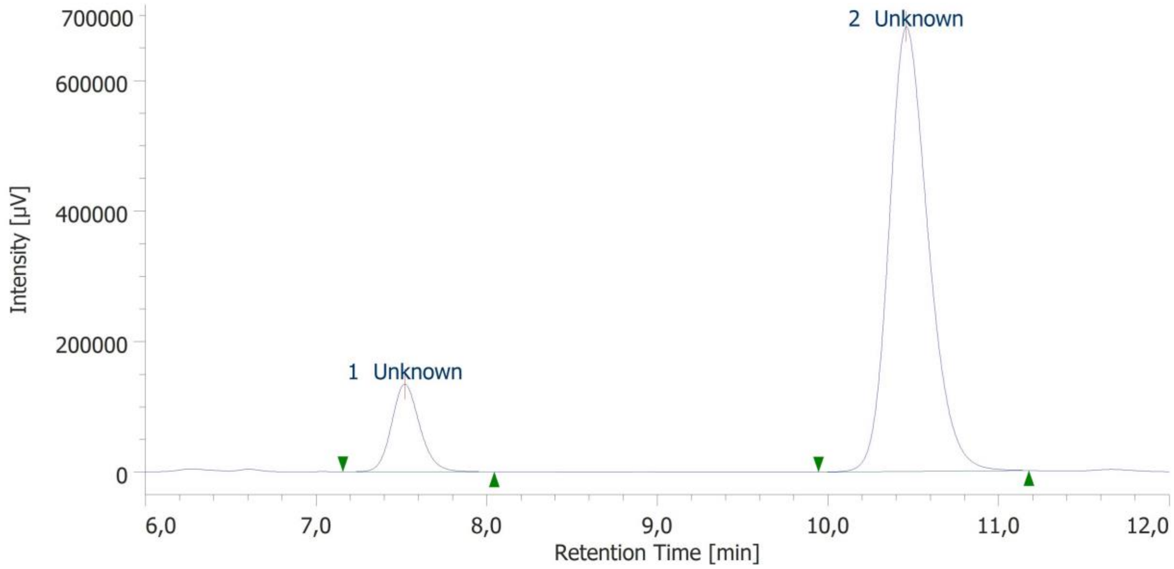

#	Peak Name	CH	tR [min]	Area [µV·sec]	Height [µV]	Area%	Height%	Quantity	NTP	Resolution	Symmetry Factor	Warning
1	Unknown	11	7,520	1540565	134165	12,406	16,465	N/A	10354	8,312	1,133	
2	Unknown	11	10,457	10877540	680700	87,594	83,535	N/A	10262	N/A	1,227	

Methyl (1*S*,2*S*,3*S*)-1,2-bis(4-bromophenyl)-3-hydroxy-6,7-dimethyl-2,3-dihydro-1*H*-pyrrolizine-5-carboxylate (4y)

IE-column: Hex/ *i*PrOH 90:10 $^{v}/_{v}$, 1.0 ml/ min, λ = 284 nm

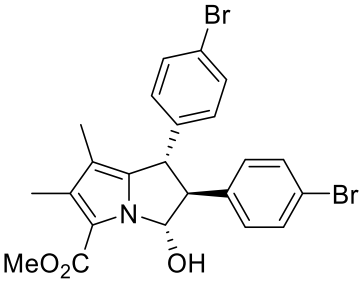

4y

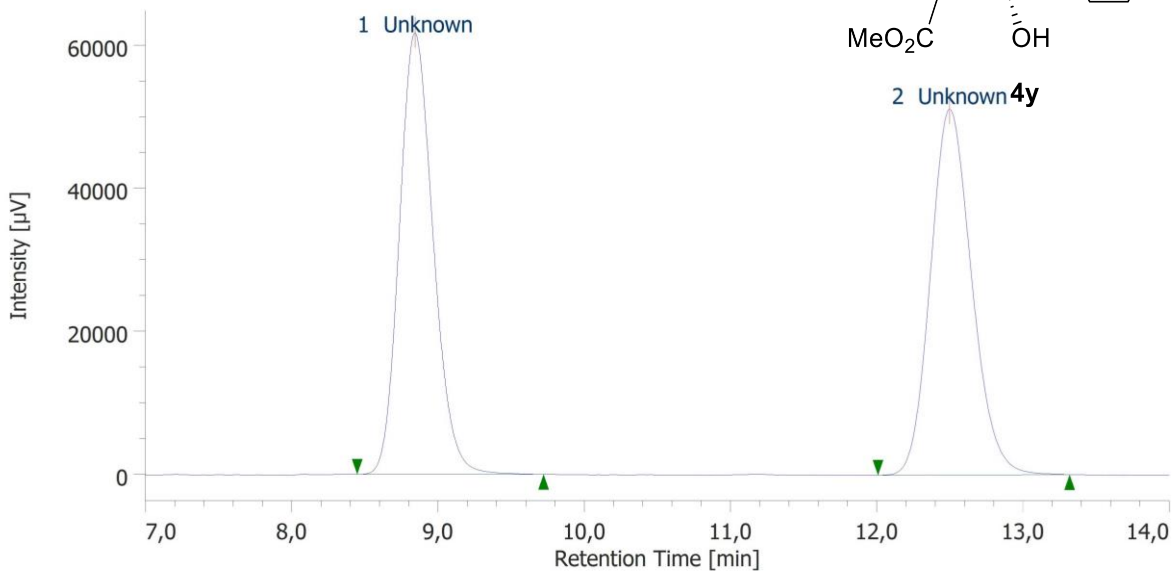

#	Peak Name	CH	tR [min]	Area [µV·sec]	Height [µV]	Area%	Height%	Quantity	NTP	Resolution	Symmetry Factor	Warning
1	Unknown	11	8,843	992385	61729	49,767	54,677	N/A	7080	7,857	1,160	
2	Unknown	11	12,497	1001690	51168	50,233	45,323	N/A	9532	N/A	1,151	

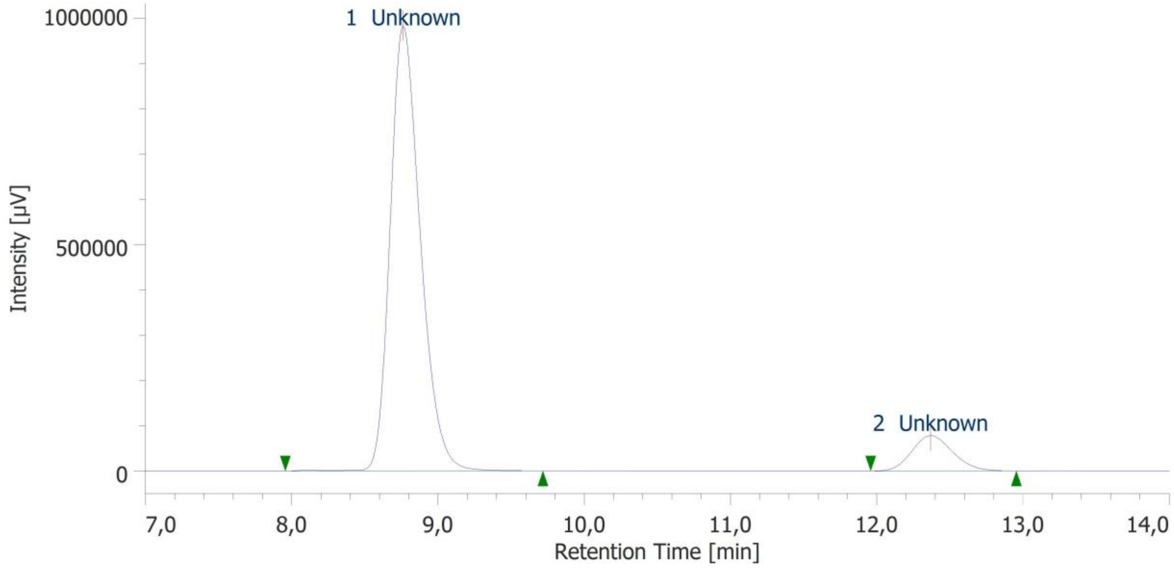

#	Peak Name	CH	tR [min]	Area [µV·sec]	Height [µV]	Area%	Height%	Quantity	NTP	Resolution	Symmetry Factor	Warning
1	Unknown	11	8,760	14109084	982555	90,360	92,661	N/A	8895	8,214	1,283	
2	Unknown	11	12,367	1505261	77819	9,640	7,339	N/A	9444	N/A	1,158	

Methyl (1*S*,2*S*,3*S*)-3-hydroxy-6,7-dimethyl-1-(naphthalen-2-yl)-2-phenyl-2,3-dihydro-1*H*-pyrrolizine-5-carboxylate (4z)

IA-column: Hex/ *i*PrOH 98:2 $^{v}/_{v}$, 1.0 ml/ min, λ = 284 nm

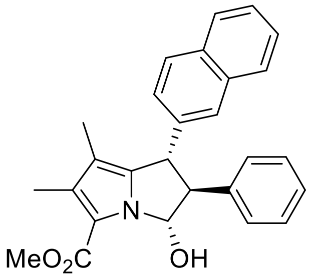

4z

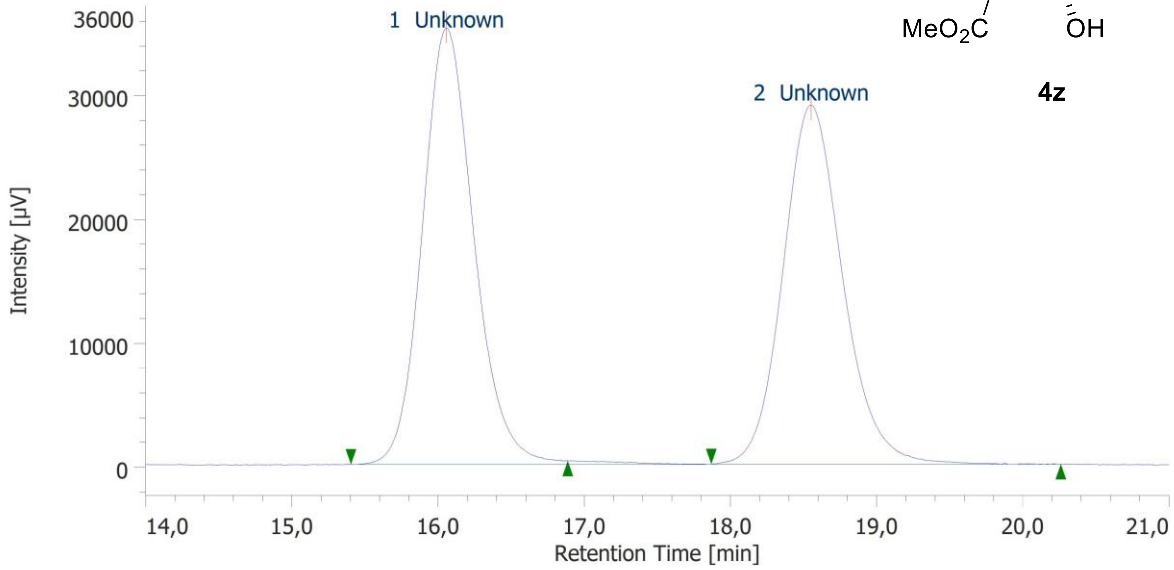

#	Peak Name	CH	tR [min]	Area [µV·sec]	Height [µV]	Area%	Height%	Quantity	NTP	Resolution	Symmetry Factor	Warning
1	Unknown	11	16,055	861409	35204	51,162	54,852	N/A	10432	3,691	1,158	
2	Unknown	11	18,550	822275	28976	48,838	45,148	N/A	10420	N/A	1,141	

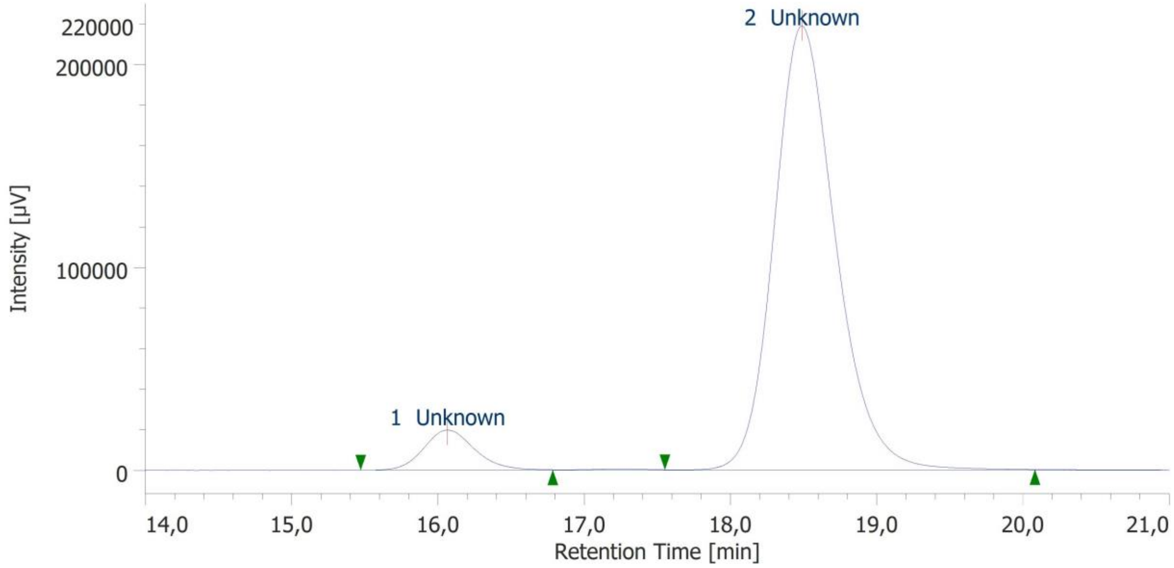

#	Peak Name	CH	tR [min]	Area [µV·sec]	Height [µV]	Area%	Height%	Quantity	NTP	Resolution	Symmetry Factor	Warning
1	Unknown	11	16,065	481430	19877	6,988	8,327	N/A	10694	3,544	1,159	
2	Unknown	11	18,487	6408228	218834	93,012	91,673	N/A	9753	N/A	1,198	

Methyl (1*S*,2*S*,3*S*)-3-hydroxy-1-(4-methoxyphenyl)-6,7-dimethyl-2-phenyl-2,3-dihydro-1*H*-pyrrolizine-5-carboxylate (4aa)

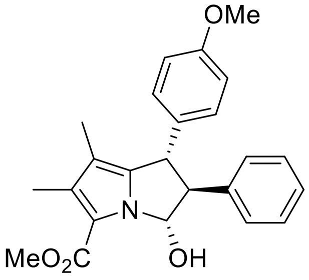

4aa

IA-column: Hex/ *i*PrOH 90:10 $^{v}/_{v}$, 1.0 ml/ min, λ = 284 nm

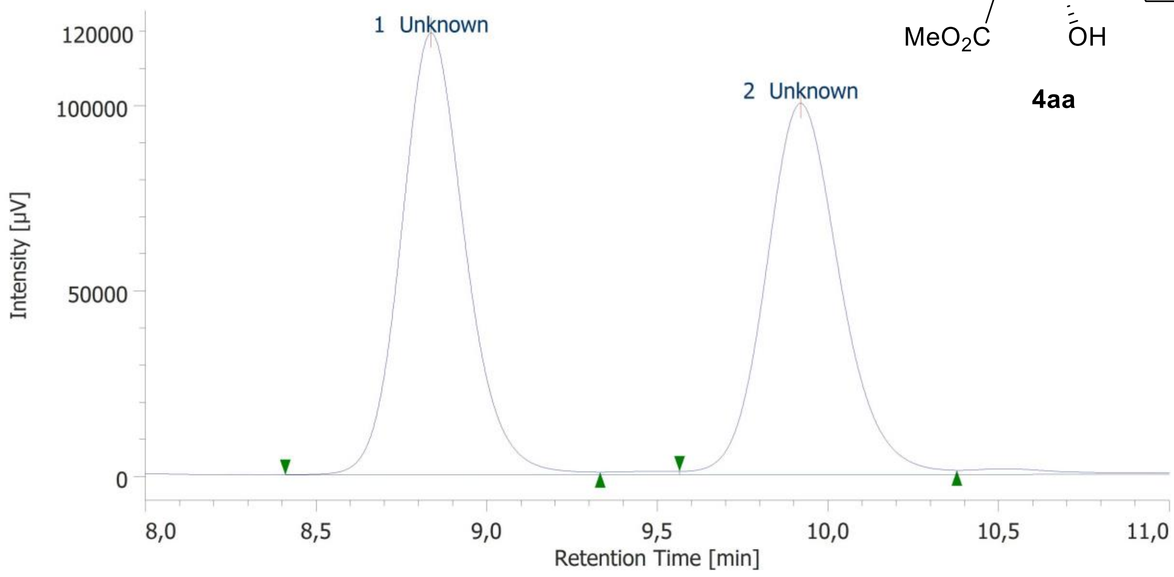

#	Peak Name	CH	tR [min]	Area [μV·sec]	Height [μV]	Area%	Height%	Quantity	NTP	Resolution	Symmetry Factor	Warning
1	Unknown	11	8,837	1556925	119105	50,669	54,354	N/A	11127	2,997	1,141	
2	Unknown	11	9,920	1515814	100024	49,331	45,646	N/A	10366	N/A	1,135	

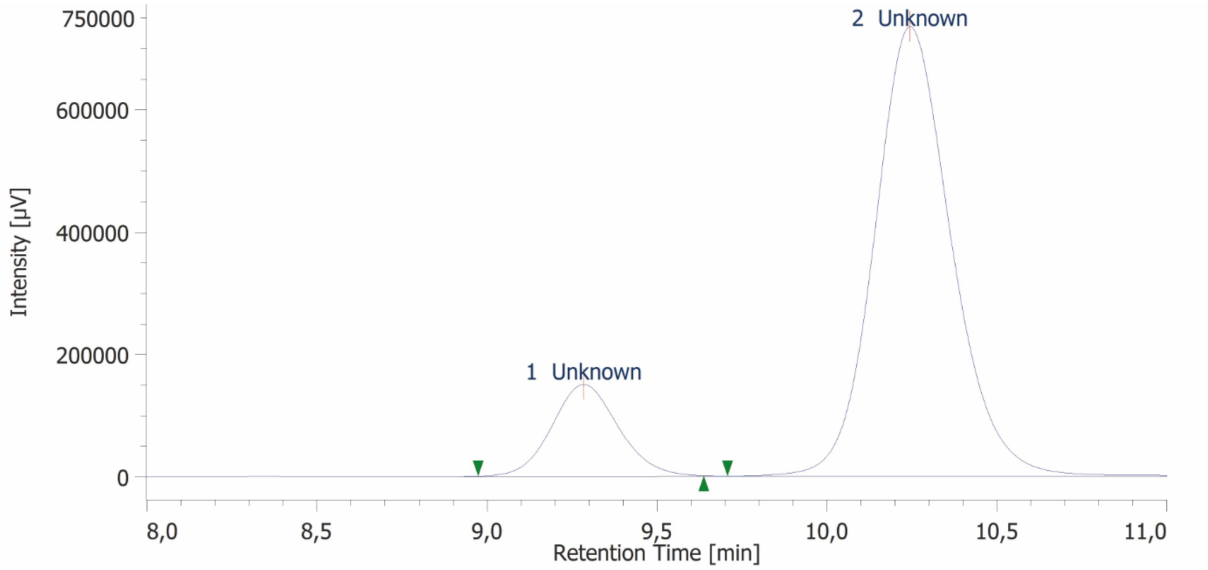

#	Peak Name	CH	tR [min]	Area [μV·sec]	Height [μV]	Area%	Height%	Quantity	NTP	Resolution	Symmetry Factor	Warning
1	Unknown	11	9,283	2068358	150670	15,156	17,019	N/A	10900	2,540	1,097	
2	Unknown	11	10,243	11579081	734616	84,844	82,981	N/A	10369	N/A	1,137	

Methyl (1*S*,2*S*,3*R*)-6,7-dimethyl-1,2-diphenyl-3-(1H-pyrrol-2-yl)-2,3-dihydro-1*H*-pyrrolizine-5-carboxylate (5)

IA-column: Hex/ *i*PrOH 95:5 $^v/_v$, 1.0 ml/ min, λ = 284 nm

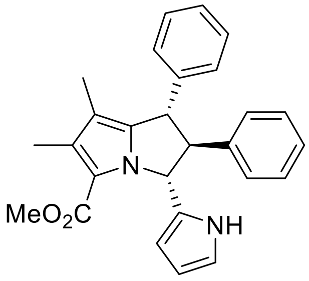

5

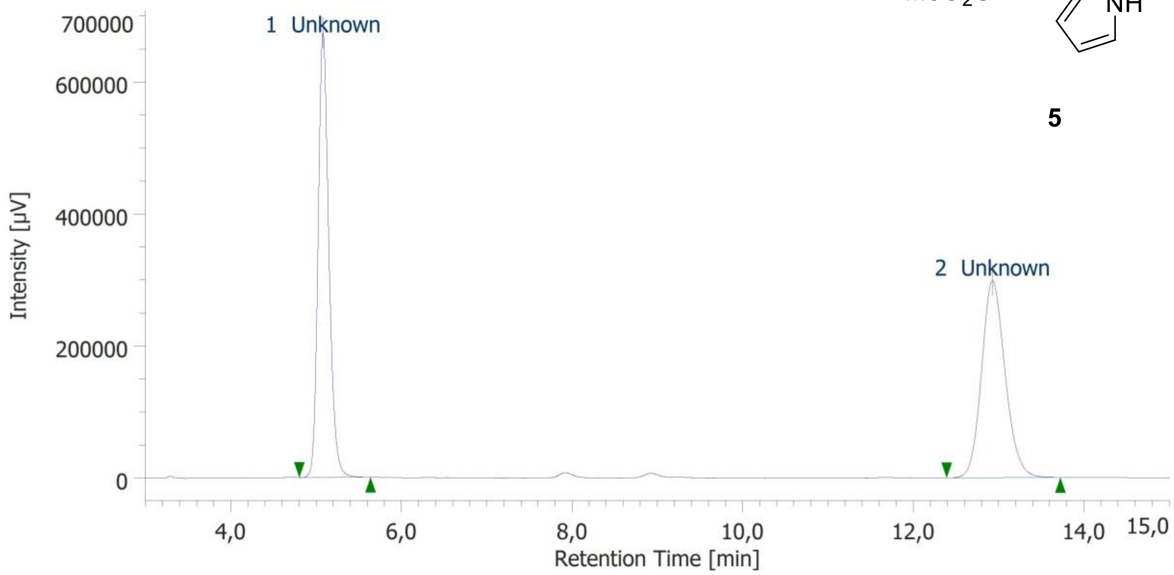

#	Peak Name	CH	tR [min]	Area [µV·sec]	Height [µV]	Area%	Height%	Quantity	NTP	Resolution	Symmetry Factor	Warning
1	Unknown	11	5,080	5888968	673140	50,687	69,268	N/A	8220	21,830	1,213	
2	Unknown	11	12,925	5729324	298648	49,313	30,732	N/A	10841	N/A	1,151	

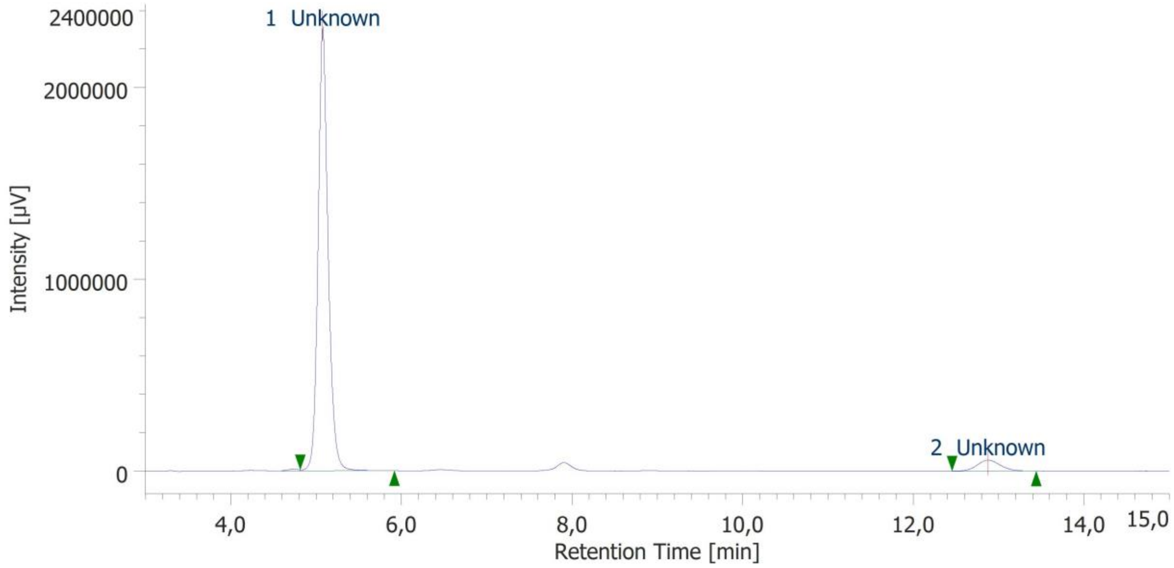

#	Peak Name	CH	tR [min]	Area [µV·sec]	Height [µV]	Area%	Height%	Quantity	NTP	Resolution	Symmetry Factor	Warning
1	Unknown	11	5,077	18851545	2315079	94,633	97,608	N/A	9937	22,502	1,170	
2	Unknown	11	12,873	1069196	56735	5,367	2,392	N/A	10994	N/A	1,124	

Methyl (1*S*,2*S*)-6,7-dimethyl-3-oxo-1,2-diphenyl-2,3-dihydro-1H-pyrrolizine-5-carboxylate (6)

IB-column: Hex/ *i*PrOH 95:5 $^{v}/_{v}$, 1.0 ml/ min, λ = 288 nm

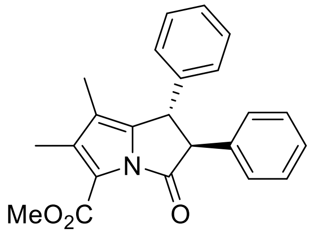

6

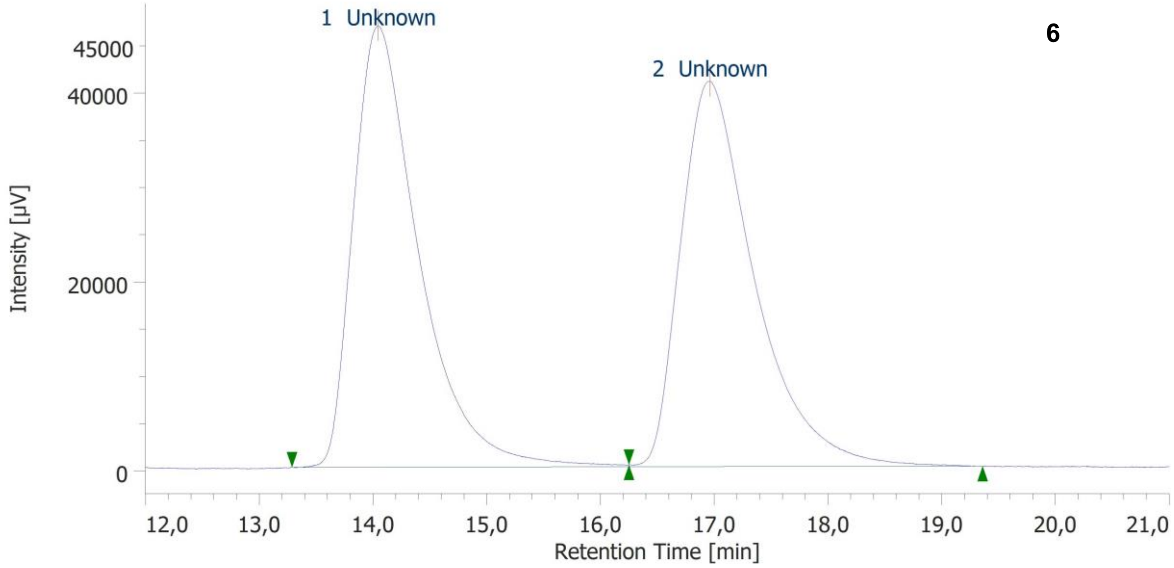

#	Peak Name	CH	tR [min]	Area [µV·sec]	Height [µV]	Area%	Height%	Quantity	NTP	Resolution	Symmetry Factor	Warning
1	Unknown	11	14,045	1863158	46698	50,399	53,393	N/A	3156	2,736	1,676	
2	Unknown	11	16,962	1833646	40763	49,601	46,607	N/A	3557	N/A	1,613	

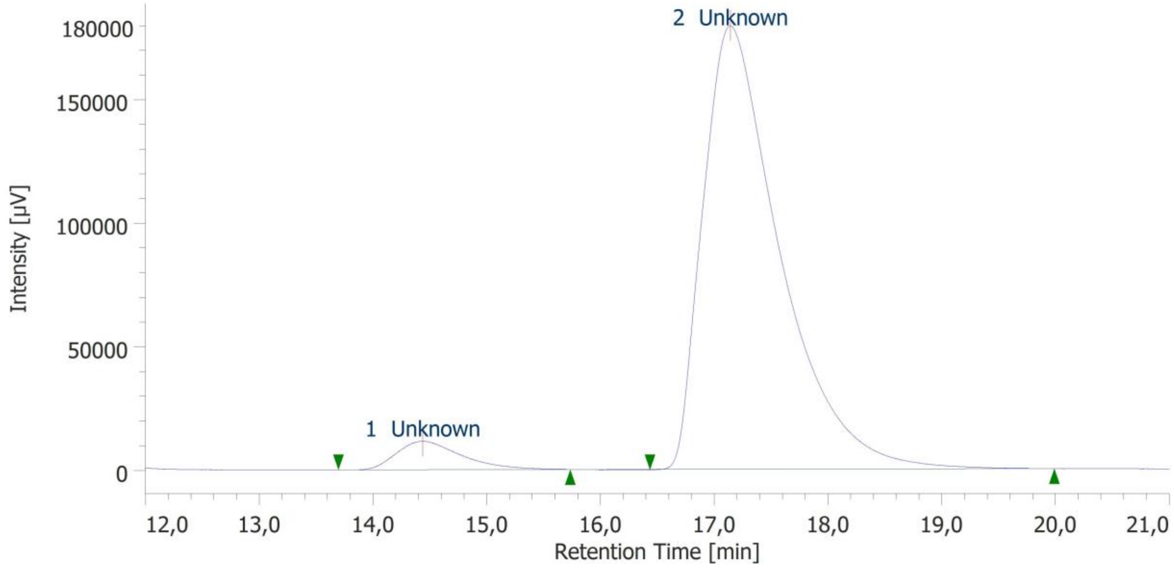

#	Peak Name	CH	tR [min]	Area [µV·sec]	Height [µV]	Area%	Height%	Quantity	NTP	Resolution	Symmetry Factor	Warning
1	Unknown	11	14,437	455321	11498	5,130	6,026	N/A	3185	2,447	1,484	
2	Unknown	11	17,140	8419602	179321	94,870	93,974	N/A	3305	N/A	1,821	

4. Crystallographic Data

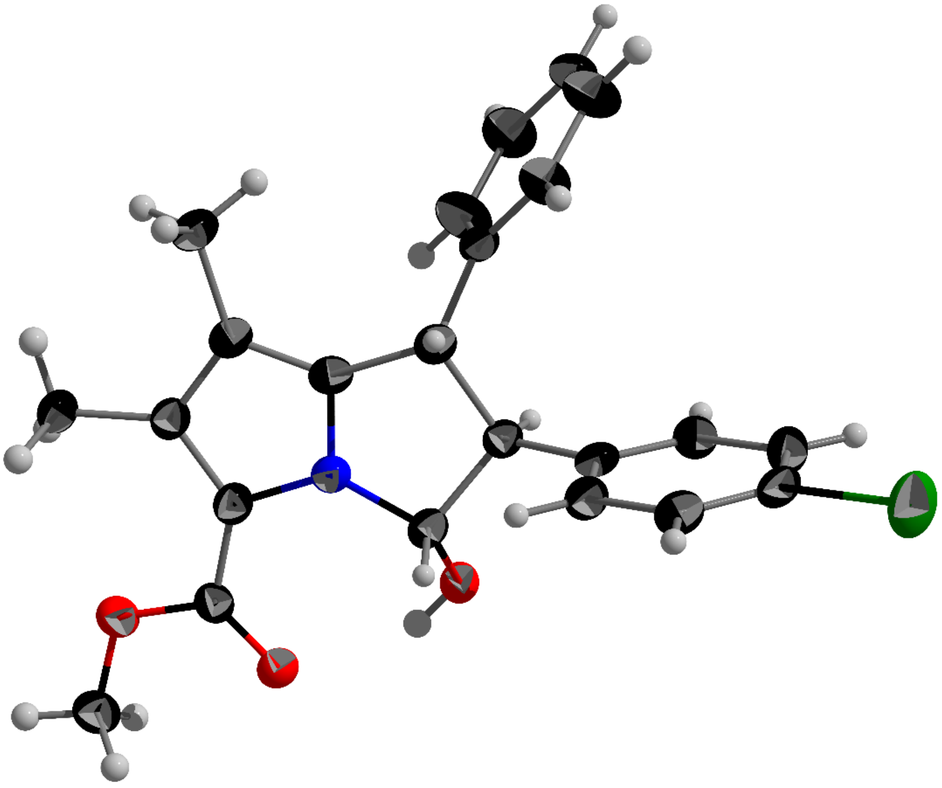

Figure S1: X-Ray crystal structure of **4f**. Thermal ellipsoids are set at 50 % probability.

The crystal was prepared by slow cooling of a saturated methanol solution of **4f** 60 °C. A single crystal of **4f** was mounted and the diffraction data was collected on a STOE STADIVARI diffractometer using Cu-*K*α radiation (λ = 1.54186 Å) and ω-scan rotation. The structure was solved with SHELXL-2014/7. Absorption correction was done by Gaussian integration using STOE X-Red32.[5] Afterwards scaling of reflection intensities was performed within STOE LANA.[6] Finally, a spherical absorption correction was done within STOE LANA. Reflections were merged by SHELXL according to the crystal class for the calculation of statistics and refinement. Anisotropic refinement of all non-hydrogen atoms with SHELXL-2014/7. Excluding H atoms bonded to the hydroxyl group, all hydrogen atoms were calculated on idealized positions. The absolute structure was established by anomalous dispersion effects. Structure was drawn using Diamond 4 and POV-Ray v3.7. The crystallographic refinement parameters are given below:

Table S7: Crystal data and structure refinement of **4f**.

**CCDC**	2376720
**Empirical formula**	$C_{23}H_{22}BrNO_3$
**Formula Weight**	440.32
**Temperature**	130(2) K
**Crystal system**	Monoclinic

**Space group**	$P2_1$
**a**	1359.54(11) pm
**b**	507.62(3) pm
**c**	1475.63(11) pm
**α**	90 °
**β**	90.290(6) °
**γ**	90 °
**Volume**	1.01836(13) nm^3
**Z**	2
**Radiation type**	Cu *K*α
**Density (calculated)**	1.436 gcm^{-3}
**F(000)**	452
**Absorption coefficient**	2.939 mm^{-1}
**Crystal size**	0.700 x 0.070 x 0.016 mm^3
**T_{min}, T_{max}**	0.117, 0.854
**No. of measured, independent and observed [$I > 2\sigma(I)$] reflections**	17325, 2512, 2272
**R_{int}**	0.058
**$(\sin \theta/\lambda)_{max}$ ($Å^{-1}$)**	0.609
**Theta range for data collection**	2.995 to 69.912
**Index ranges**	-16<=h<=16, -5<=k<=2, -17<=l<=17
**Completeness to theta = 69.912 °**	97.5 %
**$R[F^2 > 2\sigma(F^2)]$, $\omega R(F^2)$, S**	0.039, 0.103, 1.06
**No. of reflections**	2512
**No. of parameters**	260
**No. of restraints**	1
**H-atom treatment**	H atoms treated by a mixture of independent and constrained refinement
**Δ〉max, Δ〉min (e $Å^{-3}$)**	0.32, -0.63
**Absolute structure parameter**	-0.04 (3)

Table S8: Fractional atomic coordinates and isotropic or equivalent isotropic displacement parameters ($Å^2$) for **4f**. U_{eq} is defined as one third of the trace of the orthogonalized U^{ij} tensor. For hydrogen atoms, excluding bonded to OH fragments (H1), idealized positions are calculated and U_{iso} is given instead.

	x	y	z	U_{iso}*/U_{eq}
**C1**	0.8244 (4)	0.6330 (10)	0.2194 (3)	0.0298 (10)
**H1A**	0.8850	0.5230	0.2265	0.036*
**C2**	0.7525 (3)	0.5784 (9)	0.2969 (3)	0.0282 (9)
**H2**	0.7137	0.7436	0.3059	0.034*
**C3**	0.6785 (3)	0.3649 (9)	0.2612 (3)	0.0288 (9)

**H3**	0.7027	0.1868	0.2797	0.035*
**C4**	0.6894 (3)	0.3932 (9)	0.1607 (3)	0.0292 (9)
**C5**	0.6522 (3)	0.2870 (11)	0.0812 (3)	0.0293 (8)
**C6**	0.7107 (3)	0.3834 (10)	0.0104 (3)	0.0309 (9)
**C7**	0.7837 (3)	0.5443 (10)	0.0477 (3)	0.0315 (9)
**C8**	0.5672 (3)	0.0964 (10)	0.0741 (3)	0.0354 (10)
**H8A**	0.5289	0.1349	0.0193	0.053*
**H8B**	0.5251	0.1147	0.1274	0.053*
**H8C**	0.5926	-0.0841	0.0710	0.053*
**C9**	0.6953 (3)	0.3190 (11)	-0.0888 (3)	0.0412 (13)
**H9A**	0.7526	0.2229	-0.1116	0.062*
**H9B**	0.6871	0.4827	-0.1233	0.062*
**H9C**	0.6363	0.2098	-0.0958	0.062*
**C10**	0.8614 (3)	0.7010 (9)	0.0077 (3)	0.0311 (10)
**C11**	0.9417 (3)	0.8293 (10)	-0.1255 (3)	0.0411 (12)
**H11A**	0.9410	0.7945	-0.1909	0.062*
**H11B**	1.0058	0.7796	-0.1002	0.062*
**H11C**	0.9300	1.0172	-0.1148	0.062*
**C12**	0.7963 (3)	0.5085 (9)	0.3881 (3)	0.0281 (9)
**C13**	0.7690 (3)	0.6477 (10)	0.4641 (3)	0.0332 (9)
**H13**	0.7256	0.7933	0.4579	0.040*
**C14**	0.8041 (3)	0.5790 (10)	0.5502 (3)	0.0370 (11)
**H14**	0.7854	0.6765	0.6023	0.044*
**C15**	0.8667 (3)	0.3651 (11)	0.5573 (3)	0.0342 (10)
**C16**	0.8968 (3)	0.2250 (11)	0.4831 (3)	0.0347 (10)
**H16**	0.9411	0.0815	0.4897	0.042*
**C17**	0.8610 (3)	0.2968 (11)	0.3975 (3)	0.0330 (10)
**H17**	0.8810	0.2008	0.3455	0.040*
**C18**	0.5766 (3)	0.4062 (9)	0.2998 (3)	0.0286 (9)
**C19**	0.5138 (4)	0.5974 (12)	0.2659 (4)	0.0478 (14)
**H19**	0.5324	0.6950	0.2138	0.057*
**C20**	0.4244 (4)	0.6489 (12)	0.3066 (4)	0.0470 (13)
**H20**	0.3822	0.7802	0.2820	0.056*
**C21**	0.3961 (3)	0.5122 (11)	0.3821 (3)	0.0390 (11)
**H21**	0.3353	0.5502	0.4106	0.047*
**C22**	0.4574 (4)	0.3175 (11)	0.4166 (4)	0.0483 (14)
**H22**	0.4380	0.2189	0.4682	0.058*
**C23**	0.5471 (3)	0.2669 (14)	0.3757 (3)	0.0388 (9)
**H23**	0.5889	0.1346	0.4001	0.047*
**N1**	0.7676 (3)	0.5474 (7)	0.1403 (2)	0.0290 (8)
**O1**	0.8489 (3)	0.9005 (7)	0.2177 (2)	0.0338 (8)

**H1**	0.883 (5)	0.916 (16)	0.175 (4)	0.06 (2)*
**O2**	0.9190 (2)	0.8403 (6)	0.0500 (2)	0.0363 (8)
**O3**	0.8653 (2)	0.6769 (7)	-0.0824 (2)	0.0395 (8)
**Br1**	0.91305 (4)	0.26605 (14)	0.67455 (3)	0.0535 (2)

Table S9: Atomic displacement parameters ($Å^2$) for **4f**.

	U^{11}	U^{22}	U^{33}	U^{12}	U^{13}	U^{23}
**C1**	0.028 (2)	0.030 (2)	0.031 (3)	0.0009 (18)	-0.0043 (18)	-0.0013 (18)
**C2**	0.025 (2)	0.028 (2)	0.031 (2)	0.0016 (17)	-0.0018 (16)	-0.0020 (16)
**C3**	0.026 (2)	0.029 (2)	0.032 (2)	0.0026 (17)	-0.0036 (16)	0.0016 (16)
**C4**	0.025 (2)	0.027 (2)	0.036 (2)	0.0020 (18)	-0.0012 (17)	0.0017 (18)
**C5**	0.0272 (18)	0.026 (2)	0.0343 (18)	0.0024 (19)	-0.0056 (14)	0.002 (2)
**C6**	0.029 (2)	0.033 (2)	0.031 (2)	0.0032 (18)	-0.0036 (17)	-0.0028 (18)
**C7**	0.031 (2)	0.035 (2)	0.028 (2)	0.0012 (18)	-0.0041 (17)	0.0005 (17)
**C8**	0.033 (2)	0.035 (3)	0.038 (2)	-0.006 (2)	-0.0083 (19)	0.0030 (19)
**C9**	0.035 (2)	0.057 (4)	0.032 (2)	-0.006 (2)	-0.0046 (17)	-0.007 (2)
**C10**	0.028 (2)	0.037 (3)	0.0285 (19)	0.0048 (17)	0.0003 (16)	0.0007 (16)
**C11**	0.040 (2)	0.046 (4)	0.038 (2)	-0.005 (2)	0.0048 (19)	0.0072 (19)
**C12**	0.0213 (19)	0.029 (2)	0.035 (2)	-0.0030 (17)	-0.0039 (16)	0.0016 (17)
**C13**	0.029 (2)	0.035 (2)	0.035 (2)	0.0051 (19)	-0.0012 (17)	-0.0005 (18)
**C14**	0.036 (2)	0.046 (3)	0.029 (2)	0.003 (2)	-0.0034 (18)	-0.0032 (19)
**C15**	0.028 (2)	0.045 (3)	0.029 (2)	-0.0049 (19)	-0.0097 (17)	0.0058 (18)
**C16**	0.0290 (19)	0.036 (3)	0.039 (2)	0.003 (2)	-0.0061 (16)	0.002 (2)
**C17**	0.030 (2)	0.035 (3)	0.0341 (19)	0.001 (2)	-0.0026 (15)	-0.004 (2)
**C18**	0.0251 (19)	0.031 (2)	0.030 (2)	-0.0035 (17)	-0.0037 (16)	-0.0009 (17)
**C19**	0.039 (3)	0.058 (4)	0.046 (3)	0.011 (2)	0.005 (2)	0.022 (3)
**C20**	0.036 (2)	0.057 (3)	0.049 (3)	0.015 (2)	-0.002 (2)	0.012 (2)
**C21**	0.026 (2)	0.047 (3)	0.044 (3)	-0.001 (2)	0.0049 (19)	-0.001 (2)
**C22**	0.041 (3)	0.051 (4)	0.053 (3)	0.003 (2)	0.012 (2)	0.013 (2)
**C23**	0.037 (2)	0.037 (2)	0.042 (2)	0.006 (3)	0.0029 (17)	0.008 (3)
**N1**	0.0254 (18)	0.031 (2)	0.0305 (18)	-0.0010 (15)	0.0002 (14)	-0.0020 (14)
**O1**	0.0370 (18)	0.0328 (18)	0.0316 (19)	-0.0067 (15)	0.0025 (15)	-0.0047 (14)
**O2**	0.0335 (16)	0.042 (2)	0.0337 (15)	-0.0069 (14)	-0.0003 (13)	-0.0025 (13)
**O3**	0.0352 (16)	0.054 (2)	0.0293 (15)	-0.0059 (15)	0.0014 (13)	0.0039 (14)
**Br1**	0.0583 (3)	0.0661 (4)	0.0359 (3)	0.0116 (3)	-0.0168 (2)	0.0038 (3)

Table S10: Bond lengths [Å] for **4f**.

	Bond length [Å]		Bond length [Å]
**C1—O1**	1.398 (6)	**C11—H11A**	0.9800
**C1—N1**	1.463 (6)	**C11—H11B**	0.9800

**C1—C2**	1.533 (6)	**C11—H11C**	0.9800
**C1—H1A**	1.0000	**C12—C13**	1.378 (7)
**C2—C12**	1.510 (6)	**C12—C17**	1.396 (7)
**C2—C3**	1.569 (6)	**C13—C14**	1.399 (6)
**C2—H2**	1.0000	**C13—H13**	0.9500
**C3—C4**	1.498 (6)	**C14—C15**	1.384 (7)
**C3—C18**	1.515 (6)	**C14—H14**	0.9500
**C3—H3**	1.0000	**C15—C16**	1.369 (7)
**C4—N1**	1.355 (6)	**C15—Br1**	1.906 (4)
**C4—C5**	1.384 (6)	**C16—C17**	1.400 (6)
**C5—C6**	1.404 (6)	**C16—H16**	0.9500
**C5—C8**	1.510 (6)	**C17—H17**	0.9500
**C6—C7**	1.396 (6)	**C18—C19**	1.384 (7)
**C6—C9**	1.514 (6)	**C18—C23**	1.386 (6)
**C7—N1**	1.385 (6)	**C19—C20**	1.384 (7)
**C7—C10**	1.451 (6)	**C19—H19**	0.9500
**C8—H8A**	0.9800	**C20—C21**	1.369 (7)
**C8—H8B**	0.9800	**C20—H20**	0.9500
**C8—H8C**	0.9800	**C21—C22**	1.388 (7)
**C9—H9A**	0.9800	**C21—H21**	0.9500
**C9—H9B**	0.9800	**C22—C23**	1.387 (7)
**C9—H9C**	0.9800	**C22—H22**	0.9500
**C10—O2**	1.224 (5)	**C23—H23**	0.9500
**C10—O3**	1.337 (5)	**O1—H1**	0.80 (6)
**C11—O3**	1.445 (5)		

Table S11: Bond angles [°] for **4f**.

	**Bond angles [°]**		**Bond angles [°]**
**O1—C1—N1**	113.5 (4)	**O3—C11—H11B**	109.5
**O1—C1—C2**	110.0 (4)	**H11A—C11—H11B**	109.5
**N1—C1—C2**	101.9 (4)	**O3—C11—H11C**	109.5
**O1—C1—H1A**	110.4	**H11A—C11—H11C**	109.5
**N1—C1—H1A**	110.4	**H11B—C11—H11C**	109.5
**C2—C1—H1A**	110.4	**C13—C12—C17**	119.1 (4)
**C12—C2—C1**	117.2 (4)	**C13—C12—C2**	119.9 (4)
**C12—C2—C3**	112.7 (4)	**C17—C12—C2**	121.0 (4)
**C1—C2—C3**	106.5 (4)	**C12—C13—C14**	121.3 (4)
**C12—C2—H2**	106.6	**C12—C13—H13**	119.4
**C1—C2—H2**	106.6	**C14—C13—H13**	119.4
**C3—C2—H2**	106.6	**C15—C14—C13**	118.1 (4)
**C4—C3—C18**	117.0 (3)	**C15—C14—H14**	120.9

**C4—C3—C2**	101.6 (3)	**C13—C14—H14**	120.9
**C18—C3—C2**	111.4 (4)	**C16—C15—C14**	122.2 (4)
**C4—C3—H3**	108.8	**C16—C15—Br1**	119.3 (4)
**C18—C3—H3**	108.8	**C14—C15—Br1**	118.4 (4)
**C2—C3—H3**	108.8	**C15—C16—C17**	118.8 (5)
**N1—C4—C5**	108.7 (4)	**C15—C16—H16**	120.6
**N1—C4—C3**	110.9 (4)	**C17—C16—H16**	120.6
**C5—C4—C3**	139.8 (4)	**C12—C17—C16**	120.4 (4)
**C4—C5—C6**	106.8 (4)	**C12—C17—H17**	119.8
**C4—C5—C8**	125.8 (4)	**C16—C17—H17**	119.8
**C6—C5—C8**	127.4 (4)	**C19—C18—C23**	118.0 (4)
**C7—C6—C5**	108.4 (4)	**C19—C18—C3**	121.6 (4)
**C7—C6—C9**	127.0 (4)	**C23—C18—C3**	120.2 (4)
**C5—C6—C9**	124.6 (4)	**C20—C19—C18**	121.1 (5)
**N1—C7—C6**	106.2 (4)	**C20—C19—H19**	119.4
**N1—C7—C10**	121.0 (4)	**C18—C19—H19**	119.4
**C6—C7—C10**	132.7 (4)	**C21—C20—C19**	120.5 (5)
**C5—C8—H8A**	109.5	**C21—C20—H20**	119.7
**C5—C8—H8B**	109.5	**C19—C20—H20**	119.7
**H8A—C8—H8B**	109.5	**C20—C21—C22**	119.3 (4)
**C5—C8—H8C**	109.5	**C20—C21—H21**	120.4
**H8A—C8—H8C**	109.5	**C22—C21—H21**	120.4
**H8B—C8—H8C**	109.5	**C23—C22—C21**	120.0 (5)
**C6—C9—H9A**	109.5	**C23—C22—H22**	120.0
**C6—C9—H9B**	109.5	**C21—C22—H22**	120.0
**H9A—C9—H9B**	109.5	**C18—C23—C22**	121.0 (5)
**C6—C9—H9C**	109.5	**C18—C23—H23**	119.5
**H9A—C9—H9C**	109.5	**C22—C23—H23**	119.5
**H9B—C9—H9C**	109.5	**C4—N1—C7**	109.9 (4)
**O2—C10—O3**	122.2 (4)	**C4—N1—C1**	113.9 (4)
**O2—C10—C7**	125.0 (4)	**C7—N1—C1**	134.9 (4)
**O3—C10—C7**	112.8 (4)	**C1—O1—H1**	104 (6)
**O3—C11—H11A**	109.5	**C10—O3—C11**	114.9 (4)

Table S12: Bond torsion angles [°] for **4f**.

	**Bond angles [°]**		**Bond angles [°]**
**O1—C1—C2—C12**	-89.7 (5)	**C12—C13—C14—C15**	-0.4 (7)
**N1—C1—C2—C12**	149.7 (4)	**C13—C14—C15—C16**	1.6 (7)
**O1—C1—C2—C3**	143.0 (4)	**C13—C14—C15—Br1**	-178.9 (4)
**N1—C1—C2—C3**	22.4 (4)	**C14—C15—C16—C17**	-1.6 (7)
**C12—C2—C3—C4**	-150.5 (4)	**Br1—C15—C16—C17**	179.0 (4)

**C1—C2—C3—C4**	-20.6 (4)	**C13—C12—C17—C16**	0.8 (7)
**C12—C2—C3—C18**	84.2 (4)	**C2—C12—C17—C16**	-176.4 (4)
**C1—C2—C3—C18**	-145.9 (4)	**C15—C16—C17—C12**	0.3 (7)
**C18—C3—C4—N1**	132.4 (4)	**C4—C3—C18—C19**	-37.8 (7)
**C2—C3—C4—N1**	10.9 (5)	**C2—C3—C18—C19**	78.3 (5)
**C18—C3—C4—C5**	-57.9 (8)	**C4—C3—C18—C23**	147.8 (5)
**C2—C3—C4—C5**	-179.3 (6)	**C2—C3—C18—C23**	-96.1 (5)
**N1—C4—C5—C6**	-0.2 (5)	**C23—C18—C19—C20**	0.2 (8)
**C3—C4—C5—C6**	-170.1 (5)	**C3—C18—C19—C20**	-174.3 (5)
**N1—C4—C5—C8**	177.7 (4)	**C18—C19—C20—C21**	0.4 (9)
**C3—C4—C5—C8**	7.8 (9)	**C19—C20—C21—C22**	-1.2 (9)
**C4—C5—C6—C7**	0.8 (5)	**C20—C21—C22—C23**	1.3 (9)
**C8—C5—C6—C7**	-177.1 (4)	**C19—C18—C23—C22**	0.0 (8)
**C4—C5—C6—C9**	-179.4 (5)	**C3—C18—C23—C22**	174.6 (5)
**C8—C5—C6—C9**	2.7 (8)	**C21—C22—C23—C18**	-0.7 (9)
**C5—C6—C7—N1**	-1.0 (5)	**C5—C4—N1—C7**	-0.4 (5)
**C9—C6—C7—N1**	179.1 (4)	**C3—C4—N1—C7**	172.6 (4)
**C5—C6—C7—C10**	-177.6 (5)	**C5—C4—N1—C1**	-169.5 (4)
**C9—C6—C7—C10**	2.6 (9)	**C3—C4—N1—C1**	3.5 (5)
**N1—C7—C10—O2**	1.6 (7)	**C6—C7—N1—C4**	0.9 (5)
**C6—C7—C10—O2**	177.7 (5)	**C10—C7—N1—C4**	178.0 (4)
**N1—C7—C10—O3**	-179.1 (4)	**C6—C7—N1—C1**	166.7 (5)
**C6—C7—C10—O3**	-2.9 (7)	**C10—C7—N1—C1**	-16.2 (8)
**C1—C2—C12—C13**	126.8 (5)	**O1—C1—N1—C4**	-134.8 (4)
**C3—C2—C12—C13**	-109.1 (5)	**C2—C1—N1—C4**	-16.6 (5)
**C1—C2—C12—C17**	-56.0 (6)	**O1—C1—N1—C7**	59.8 (7)
**C3—C2—C12—C17**	68.2 (5)	**C2—C1—N1—C7**	178.0 (5)
**C17—C12—C13—C14**	-0.7 (7)	**O2—C10—O3—C11**	-0.6 (6)
**C2—C12—C13—C14**	176.5 (4)	**C7—C10—O3—C11**	-180.0 (4)

5. References

(1) Laue, M.; Schneider, M.; Gebauer, M.; Böhlmann, W.; Gläser, R.; Schneider, C. General, Modular Access toward Immobilized Chiral Phosphoric Acid Catalysts and Their Application in Flow Chemistry. *ACS Catal.* **2024**, *14*, 5550–5559.

(2) Kallweit, I.; Schneider, C. Brønsted Acid Catalyzed [6 + 2]-Cycloaddition of 2-Vinylindoles with in Situ Generated 2-Methide-2 H-pyrroles: Direct, Catalytic, and Enantioselective Synthesis of 2,3-Dihydro-1 *H*-pyrrolizines. *Org. Lett.* **2019**, *21*, 519–523.

(3) Spanka, M.; Schneider, C. Phosphoric Acid Catalyzed Aldehyde Addition to in Situ Generated o-Quinone Methides: An Enantio- and Diastereoselective Entry toward *cis*-3,4-Diaryl Dihydrocoumarins. *Org. Lett.* **2018**, *20*, 4769–4772.

(4) Yang, X.; Yang, S.; Xiang, L.; Pang, X.; Chen, B.; Huang, G.; Yan, R. Synthesis of 3-Arylpyridines via Palladium/Copper-Catalyzed Annulation of Allylamine/1,3-Propanediamine and Aldehydes. *Adv. Synth. Catal.* **2015**, *357*, 3732–3736.

(5) Coppens, P. *Crystallographic Computing*; Munksgaard, 1970.

(6) Koziskova, J.; Hahn, F.; Richter, J.; Kožíšek, J. Comparison of different absorption corrections on the model structure of tetrakis(µ 2 -acetato)-diaqua-di-copper(II). *Acta Chimica Slovaca* **2016**, *9*, 136–140.
